# Cobalt-based nanoparticles prepared from MOF–carbon templates as efficient hydrogenation catalysts†
†Electronic supplementary information (ESI) available. See DOI: 10.1039/c8sc02807a


**DOI:** 10.1039/c8sc02807a

**Published:** 2018-09-20

**Authors:** Kathiravan Murugesan, Thirusangumurugan Senthamarai, Manzar Sohail, Ahmad S. Alshammari, Marga-Martina Pohl, Matthias Beller, Rajenahally V. Jagadeesh

**Affiliations:** a Leibniz-Institut für Katalyse e. V. an der Universität Rostock , Albert-Einstein-Str. 29a , 18059 Rostock , Germany . Email: Matthias.Beller@catalysis.de ; Email: Jagadeesh.Rajenahally@catalysis.de; b The Center of Research Excellence in Nanotechnology (CENT) , King Fahd University of Petroleum and Minerals , Dhahran 31261 , Saudi Arabia; c King Abdulaziz City for Science and Technology , Riyadh 11442 , Saudi Arabia

## Abstract

The development of efficient and selective nanostructured catalysts for industrially relevant hydrogenation reactions continues to be an actual goal of chemical research. In particular, the hydrogenation of nitriles and nitroarenes is of importance for the production of primary amines, which constitute essential feedstocks and key intermediates for advanced chemicals, life science molecules and materials. Herein, we report the preparation of graphene shell encapsulated Co_3_O_4_- and Co-nanoparticles supported on carbon by the template synthesis of cobalt-terephthalic acid MOF on carbon and subsequent pyrolysis. The resulting nanoparticles create stable and reusable catalysts for selective hydrogenation of functionalized and structurally diverse aromatic, heterocyclic and aliphatic nitriles, and as well as nitro compounds to primary amines (>65 examples). The synthetic and practical utility of this novel non-noble metal-based hydrogenation protocol is demonstrated by upscaling several reactions to multigram-scale and recycling of the catalyst.

## Abstract 2

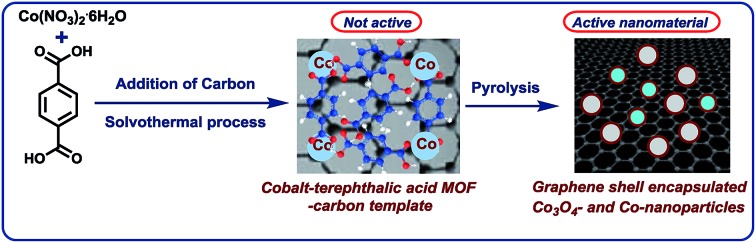
Pyrolysis of cobalt-terephthalic acid MOF template on carbon produces highly active and selective cobalt nanoparticles-based hydrogenation catalysts.

## Introduction

**Fig. 1:**
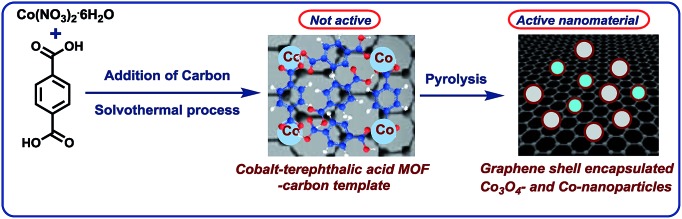
Preparation of graphene encapsulated cobalt oxide- and cobalt-nanoparticles supported on carbon by the pyrolysis of cobalt-terephthalic acid MOF–carbon template.

The development of base metal catalysts for sustainable and cost-effective processes is an actual and long standing goal of chemical research in academia and industry.^1^–^3^ Among the different synthetic technologies, catalytic hydrogenations represent a versatile tool box for the preparation of numerous fine and bulk chemicals, fuels, and life science molecules.^4^–^10^ Until today, on a practical scale these reactions mainly rely on precious metal-based catalysts,^4^–^9^ as well as RANEY® nickel.^4^–^10^ However, the availability and high price of precious metals, and selectivity problems and sensitivity of RANEY® Ni spurred interest towards alternative catalysts.^1^–^3^,^11^–^18^ In this respect, reusable supported nanoparticles are attractive due to easy separation, accessibility of active sites, control of size, and prospect of mild reaction conditions.^15^–^25^ Traditionally, supported metal nanoparticles are prepared by thermal (pyrolysis or calcination) or chemical reduction of the respective metal salts on heterogeneous supports.^19^–^25^ Despite the synthesis of literally thousands of such catalytic materials, the majority of them does not permit for the refinement of functionalized and complex molecules.^19^–^25^ However in recent years, more potent materials were prepared by using specific precursors such as metal–nitrogen complexes^15^–^17^,^26^ or structure-controlling templates.^27^–^35^ In this respect, metal organic frameworks (MOFs) built from metal ions and different organic linkers can be assembled in a highly flexible manner.^20^–^27^ So far, MOFs proved to be suitable for gas separation and storage, but also interesting catalytic applications have been realized,^27^–^30^ especially *via* subsequent pyrolysis processes.^31^–^34^ Complementary to these materials, most recently we described the use of cobalt-diamine-dicarboxylic acid-based MOFs as precursors for the preparation of supported nanoparticles and single Co atoms, which exhibit excellent catalytic activity for reductive aminations.^35^ In this case, both the nitrogen and carboxylic acid linker was necessary to produce an active material. In continuation of our efforts to develop cost-efficient materials^36^ for sustainable catalysis, herein we describe the expedient preparation of a cobalt-terephthalic acid MOF–carbon template, which forms after pyrolysis graphene shell encapsulated Co_3_O_4_/Co particles. The resulting nanoparticles are supported on carbon, which creates stable and reusable catalysts for selective hydrogenations of aliphatic and aromatic nitriles and nitro compounds (Fig. 1).

The resulting amines are of interest to chemistry, medicine, biology, biochemistry and material science.^37^–^43^ For example, amine motifs constitute an integral part of the majority of life science molecules and natural products.^37^–^43^ In fact, among 200 existing drugs, more than 170 contain at least one nitrogen atom regulating their activities.^37^ Among the different kinds of amines, primary amines attract special interest because they can be easily further transformed; thus constitute central intermediates for the production of specialty chemicals, pharmaceuticals, agrochemicals, dyes and polymers. In industry primary benzylic, aliphatic and aromatic amines are often prepared by the hydrogenation of the corresponding nitriles^44^–^46^ and nitroarenes,^15^,^16^,^47^–^53^ respectively. Apart from traditional noble metal-catalysts,^44^–^46^ Raney®-nickel^54^–^57^ or -cobalt,^57^ Fe,^58^,^59^ Co,^60^–^64^ and Mn^65^-based catalysts have recently been disclosed. Despite these achievements, still the development of active and selective nanocatalysts based on earth abundant metals is desired.

## Results and discussion

### Catalyst preparation and activity studies

At the start of our investigations, we prepared a cobalt-terephthalic acid MOF template on carbon by reacting cobalt nitrate and terephthalic acid (1 : 3) in DMF with carbon powder at 150 °C. After slow evaporation of solvent, the corresponding material was pyrolyzed at different temperatures (400, 600, 800, and 1000 °C) under inert atmosphere. The activity of the prepared potential catalysts was tested for the hydrogenation of benzonitrile to benzylamine as a bench mark reaction. Among these, cobalt-terephthalic acid MOF@C-800 was the most active material. However, a mixture of the desired benzylamine (<15%) and several side products such as *N*-benzylidenebenzylamine and dibenzylamine were observed (Table 1, entry 1). To suppress their formation, the reaction was performed in presence of hydrogen and ammonia. The latter is known to control the selective formation of primary amines from nitriles (Fig. S1†). Advantageously, under these conditions benzylamine was obtained in 97% yield (Table 1, entry 2). Other materials such as pyrolyzed cobalt nitrate on carbon (cobalt nitrate@C-800), unpyrolyzed MOF–carbon template (cobalt-terephthalic acid MOF@C) and isolated MOF (cobalt-terephthalic acid MOF) were also tested and none of these materials were active (Table 1, entries 3–5). As expected, cobalt nitrate and cobalt-terephthalic acid under homogeneous reaction conditions were also not active (Table 1, entries 6–7). Further, the cobalt-terephthalic acid MOF was pyrolyzed directly without carbon support and tested for its catalytic activity. Interestingly, this material was also found to be active and produced 96% of benzylamine (Table 1, entry 8). However, due to the high cobalt content (40% wt%), this catalyst (cobalt-terephthalic acid MOF-800) exhibits less stability compared to the cobalt-terephthalic acid MOF@C-800.

**Table 1:** Hydrogenation of benzonitrile to benzylamine: activity of cobalt catalysts^*a*^

Entry	Catalyst	Conv. (%)	Yield (%)
1^*b*^	Cobalt-terephthalic acid MOF@C-800	>99	<15
2^*c*^	Cobalt-terephthalic acid MOF@C-800	>99	97
3^*c*^	Cobalt nitrate@C-800	<5	<2
4^*c*^	Cobalt-terephthalic acid MOF@C	<2	<1
5^*c*^	Cobalt-terephthalic acid MOF	<2	<1
6^*d*^	Cobalt nitrate + terephthalic acid	<2	<1
7^*d*^	Co(NO_3_)_2_·6H_2_O	<2	<1
8^*e*^	Cobalt-terephthalic acid MOF-800	>99	96

^*a*^Materials are pyrolyzed at 800 °C for 2 h under argon. Cobalt content in the pyrolyzed sample supported on carbon = 4.5 wt%.

^*b*^Reaction conditions: Heterogeneous catalysis conditions = 0.5 mmol benzonitrile, 25 mg catalyst (3.8 mol% Co), 3 mL toluene, 25 bar H_2_, 120 °C, 15 h.

^*c*^Same as ‘a’ with 5 bar NH_3_.

^*d*^Homogeneous catalysis conditions = 0.5 mmol benzonitrile, 0.02 mmol Co(NO_3_)_2_·H_2_O, 0.06 mmol linker, 3 mL toluene, 25 bar H_2_, 5 bar NH_3_, 120 °C, 15 h.

^*e*^Material was prepared by the direct pyrolysis (800 °C, 2 h Ar) of MOF without carbon support.

To further optimize the benchmark reaction and to study the course of the formation of products, we varied different parameters such as ammonia concentration, reaction time, amount of catalyst, and hydrogen pressure in the presence of the optimal catalyst (cobalt-terephthalic acid MOF@C-800). As shown in Fig. S2,† complete conversion of benzonitrile (0.5 mmol-scale) and formation of benzylamine as the sole desired product was observed in presence of 20 mg of catalyst at 20 bar of H_2_.

### Characterization of the active nano-catalyst

**Fig. 2:**
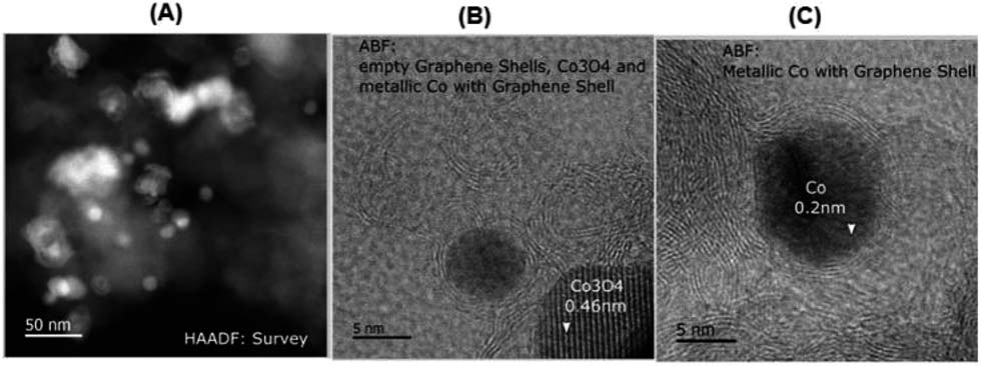
TEM images of cobalt-terephthalic acid MOF@C-800 catalyst showing Co_3_O_4_ and Co-nanoparticles (A) and metallic cobalt particles encapsulated in graphene shells (B and C).

In order to understand the structural features of the most active material, cobalt-terephthalic acid MOF@C-800 was characterized using Cs-corrected STEM (scanning transmission electron microscopy), XRD (X-ray diffraction), EPR (electron paramagnetic resonance), and XPS (X-ray photoelectron spectroscopy) spectral analysis. TEM analysis of the active material revealed the formation of cobalt oxide (Co_3_O_4_) particles with 5–30 nm size (Fig. 2). In addition, the material also contained metallic cobalt nanoparticles. Interestingly, both types of nanoparticles are encapsulated within graphene shells (Fig. 2). In contrast, the inactive material cobalt nitrate@C-800 contained hollow Co_3_O_4_ particles above 50 nm size (Fig. S4†). Notably, this material contained no graphene shells. Further, the formation of Co_3_O_4_-particles was confirmed by XRD data (Fig. S7†). Also, XRD analysis revealed the formation of cobalt-terephthalic acid MOF (Fig. S8†).

To understand the nature of the active metal species in more detail, EPR measurements were performed, too. Cobalt-terephthalic acid MOF@C-800 displayed a broad signal with a resonance peak observed at 3391 G with full width at half maximum (FWHM) 1640 G (Fig. S9†). The *g* value 2.572 observed was about 23% higher than the *g* value of free electron (2.00) in space. This indicates Co in higher oxidation states (Co^2+^/Co^3+^). The observation of only one peak indicates the presence of one paramagnetic species predominantly.^66^ Further, to confirm the presence of Co_3_O_4_, we performed XPS analysis of Co2p and O1s electrons for cobalt-terephthalic acid MOF@C-800 (Fig. S10†). The binding energy values of its most intense Co2p signals match very closely to the presence of Co_3_O_4_ at the surface of the catalyst. The Co2p^3/2^ and Co2p^1/2^ peaks appeared at 779.75 eV and 794.91 eV, respectively. The Co2p^3/2^–Co2p^1/2^ splitting energy of 15.16 eV is also in close agreement with the presence of Co_3_O_4_. Weak peaks, which are the characteristics of Co^2+^ in Co_3_O_4_, support the absence of other CoO species. It is generally known that in Co_3_O_4_, the Co2p^3/2^ spectrum of cobalt oxide is representative for high spin Co^2+^ and low spin Co^3+^ ions.^67^,^68^ The O1s surface spectra showed a significant broadening towards higher binding energy and was deconvoluted in three components. The first peak at 529.05 eV is representative of a cobalt oxide network, while the second peak present at 531.60 eV could be related to the presence of hydroxyl groups in the inner surface. The third broad peak at 535.23 eV is related to water adsorbed at the surface.^69^ The BET surface area of cobalt-terephthalic acid MOF@C-800 is 158.4 m^2^ g^–1^ and the corresponding average pore size is 7.96 nm (Fig. S9 and S10†). Notably, TEM analysis revealed the formation of both Co_3_O_4_ and metallic Co nanoparticles in the most active sample (cobalt-terephthalic acid MOF@C-800). However, XPS and XRD analyses detected only Co_3_O_4_-particles. This might be due to the small quantity or size of metallic cobalt particles, which are difficult to detect by XRD or XPS.

### Hydrogenation of functionalized (hetero)aromatic nitriles

**Fig. 3:**
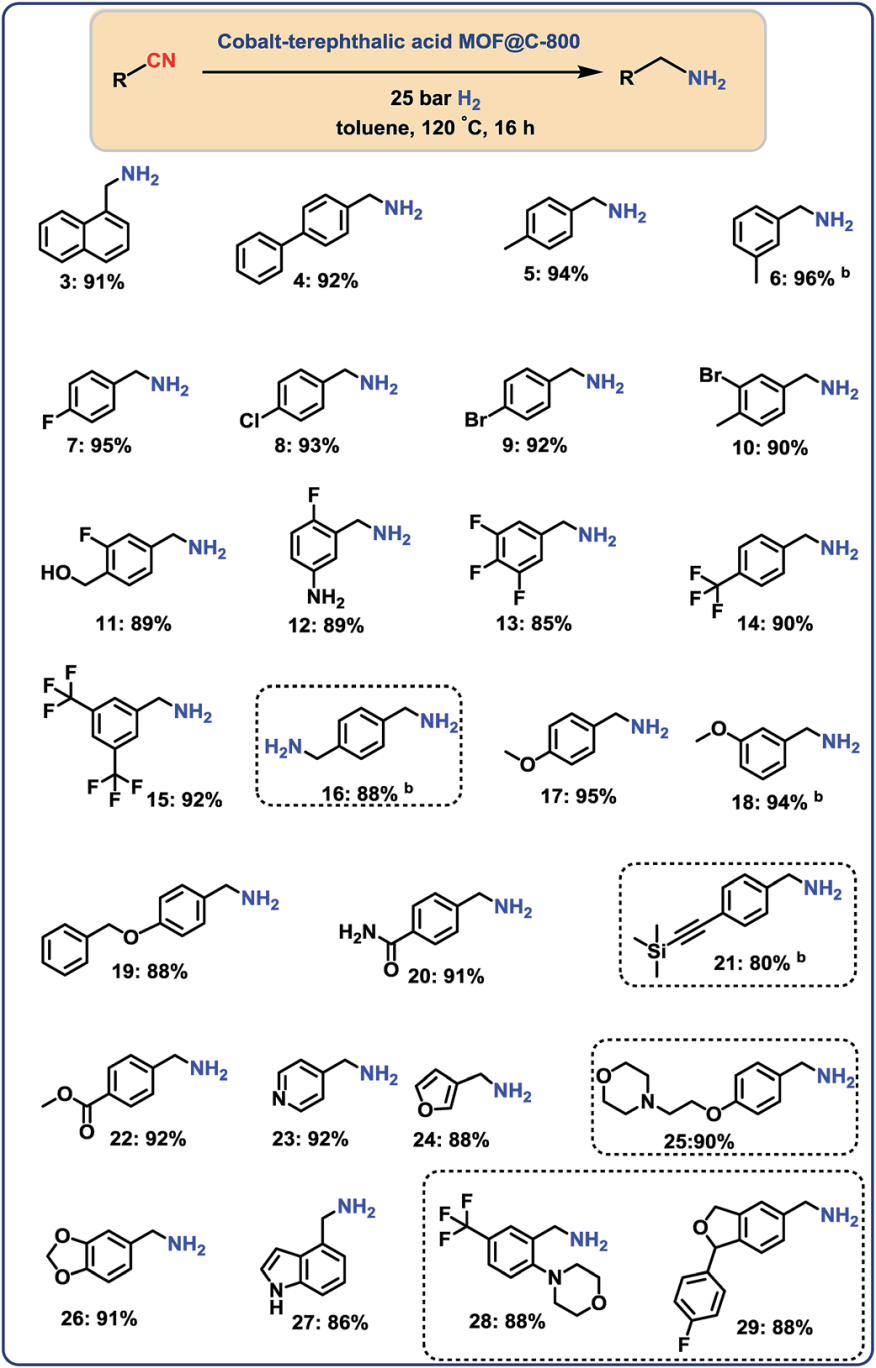
Cobalt-terephthalic acid MOF@C-800-catalyzed hydrogenation of benzonitriles and cyano-heterocycles to primary amines^a^. ^a^Reaction conditions: 0.5 mmol benzonitrile, 25 mg catalyst (3.8 mol% Co), 3 mL toluene, 25 bar H_2_, 5 bar NH_3_, 120 °C, 16 h, isolated yields. ^b^GC yields using 100 μL *n*-hexadecane. Isolated as respective hydrochloride salts.

**Fig. 4:**
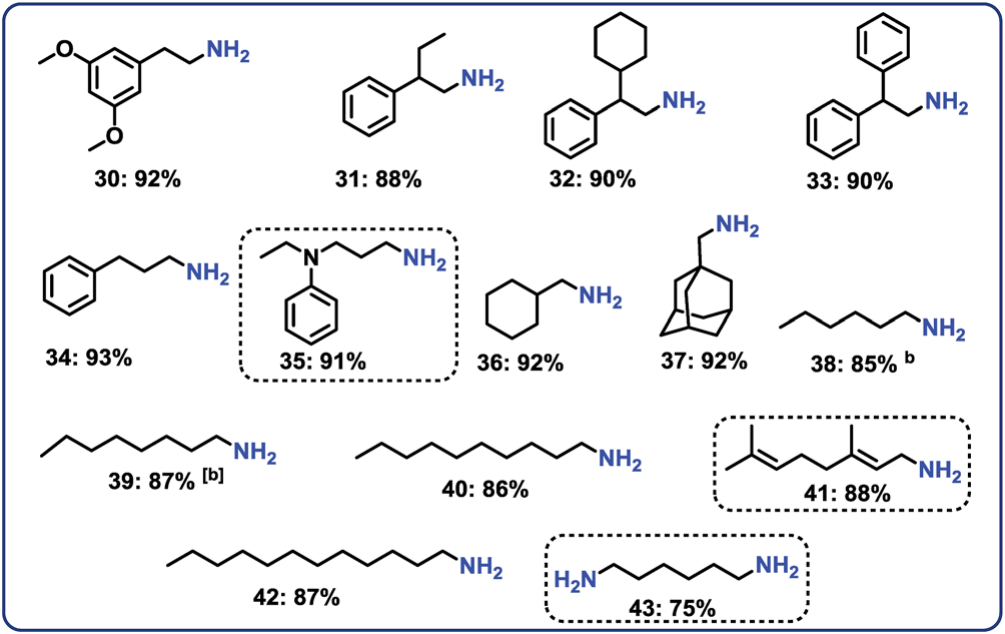
Hydrogenation of aliphatic nitriles catalyzed Co_3_O_4_/Co-nanoparticles. ^a^Reaction conditions: 0.5 mmol benzonitrile, 25 mg catalyst (3.8 mol% Co), 3 mL toluene, 25 bar H_2_, 5 bar NH_3_, 120 °C, 16 h, isolated yields. ^b^GC yields using 100 μL *n*-hexadecane. Isolated as respective hydrochloride salts.

With the optimal material (cobalt-terephthalic acid MOF@C-800) in hand, we investigated the scope and general applicability for the hydrogenation of nitriles. As shown in Fig. 3 and 4, various functionalized and structurally diverse benzonitriles, heterocyclic nitriles and challenging aliphatic ones smoothly underwent hydrogenation to produce the corresponding primary amines in good to excellent yields. For example, 8 different halogenated benzylic amines, which constitute common building blocks in the chemical industry, were obtained in 85–95% yield without significant dehalogenations (Fig. 3; products 7–14). In order to apply this protocol for advanced organic synthesis and drug discovery, achieving high chemo-selectivity is of crucial importance. In this regard, we were pleased to find excellent hydrogenation selectivity in the presence of amide, ester, and even C–C double and triple bonds (Fig. 3; products 17–21). Furthermore, diverse heterocyclic amines have been prepared from the corresponding nitriles (Fig. 3; products 23–29).

### Hydrogenation of aliphatic nitriles

Next, we performed the hydrogenation of aliphatic nitriles to the corresponding primary amines (Fig. 4). Compared to aromatic nitriles, such hydrogenations are in general more difficult and need harsher conditions. Fortunately, this catalyst is similar active and selective for the hydrogenation of aliphatic substrates, too. As a result, aliphatic amines including long chain, (bi)cyclic and as well as allylic ones were obtained in up to 93% yield (Fig. 4). The preparation of hexamethylenediamine in 75%—a central precursor for nylon 66 polymer (Fig. 4; product 43)—highlights the industrial relevance of this methodology.

### Chemoselective hydrogenation of nitroarenes to anilines

After having demonstrated the reduction of nitriles, we speculated on the utility of our cobalt-catalyst for other hydrogenations to give amines.

**Fig. 5:**
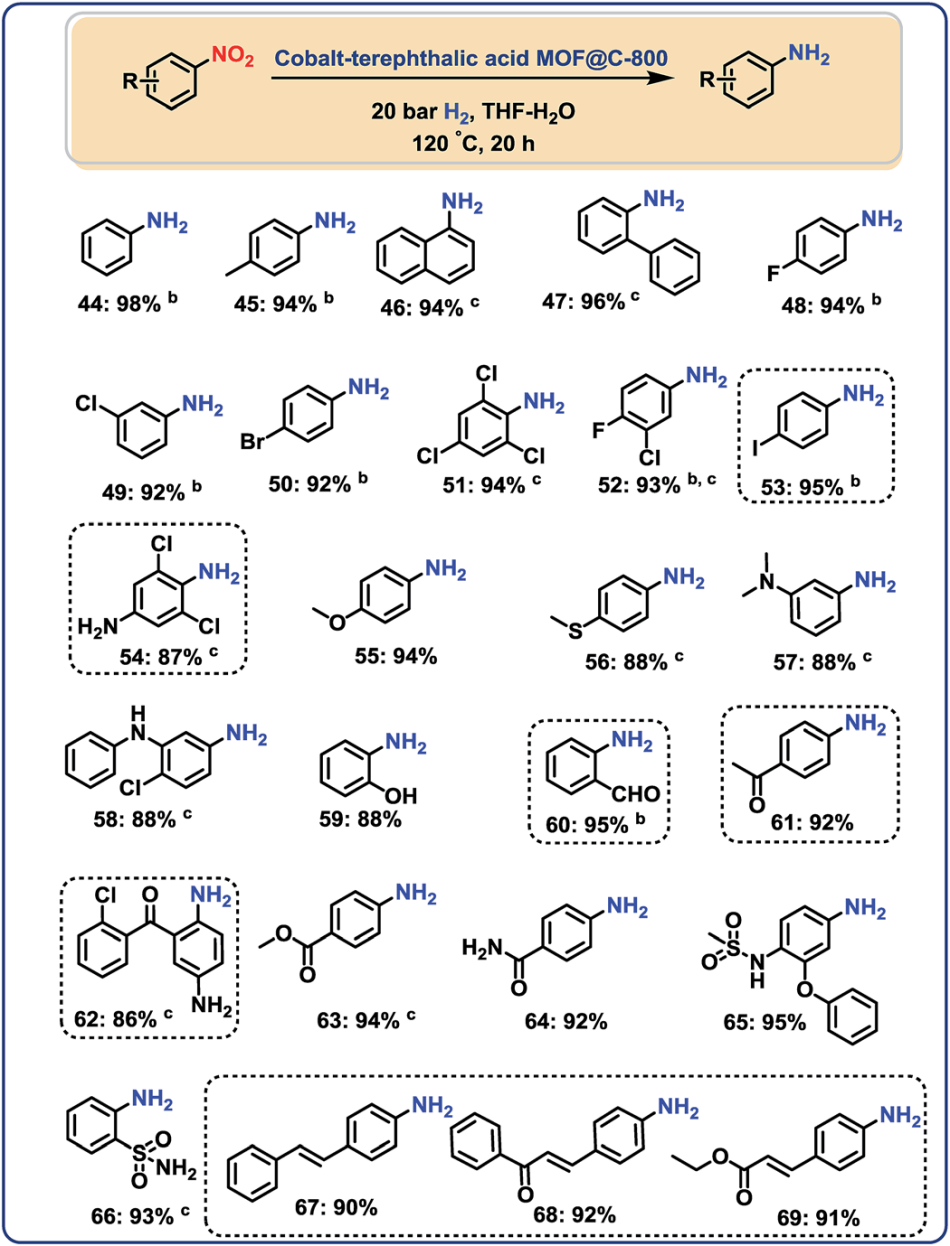
Cobalt-terephthalic acid MOF@C-800-catalyzed hydrogenation of functionalized nitroarenes to anilines^a^. ^a^Reaction conditions: 0.5 mmol nitroarene, 25 mg catalyst (3.8 mol% Co), 3 mL THF–H_2_O (1 : 1), 20 bar H_2_, 120 °C, 20 h, isolated yields. ^b^GC yields using 100 μL *n*-hexadecane. ^c^With 40 bar H_2_.

**Fig. 6:**
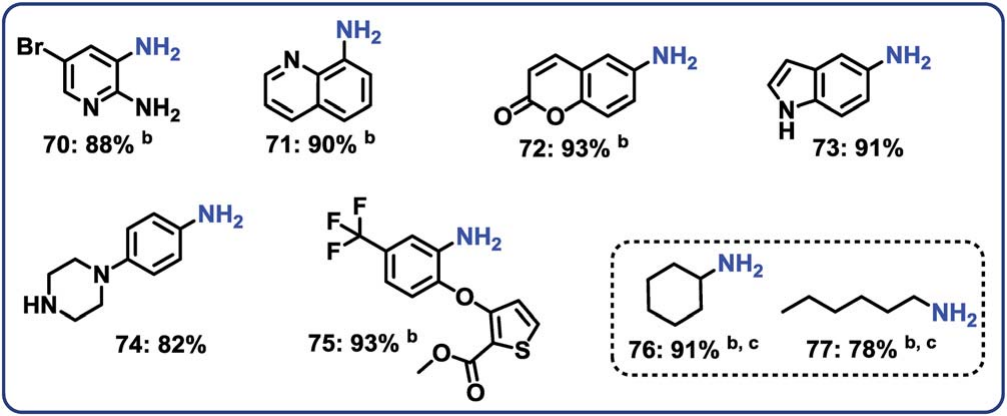
Hydrogenation of nitro-heterocycles and aliphatic nitro compounds catalyzed cobalt-terephthalic acid MOF@C-800-catalyst. ^a^Reaction conditions: 0.5 mmol nitro compound, 25 mg catalyst (3.8 mol% Co), 3 mL THF–H_2_O (1 : 1), 20 bar H_2_, 120 °C, 20 h, isolated yields. ^b^With 40 bar H_2_ in 3 mL *t*-butanol solvent. ^c^GC yields using 100 μL *n*-hexadecane.

In this context, several nitroarenes were hydrogenated to produce the corresponding anilines with excellent yields (Fig. 5–6). Similar to nitriles, aromatic, heterocyclic and aliphatic nitro compounds were selectively reduced. For example, halogenated anilines were obtained in up to 95% yield without dehalogenation (Fig. 5; products 48–54). Most interestingly, 4-iodonitrobenzene, which is a highly sensitive compound, led in 95% to the corresponding aniline (Fig. 5; product 53). In other structurally diverse and functionalized molecules the nitro group was also selectively reduced. As a result, hydroxyl groups, aldehyde, ketone, ester, amide, and C–C double bonds were untouched.

### Hydrogenation of nitro heterocycles and nitroalkanes

Next, 5 representative nitro-substituted heteroarenes were hydrogenated to produce amino-substituted N-heterocycles (Fig. 6; products 70–75). For example, the resulting 8-aminoquinoline (product 71) represents a key intermediate for preparation of primaquine, tafenoquine and pamaquine drugs, which are used in the treatment of malaria. Finally, nitrocyclohexane and 1-nitrohexane were tested. It should be noted that most of the known nitro reduction catalysts either show poor reactivity or do not work at all for aliphatic nitro compounds. Favorably, using this novel cobalt catalyst both compounds were successfully hydrogenated and produced primary amines in 78 and 91% yield, respectively (Fig. 6; products 76 and 77).

### Demonstrating scale-up and catalyst recycling

**Fig. 7:**
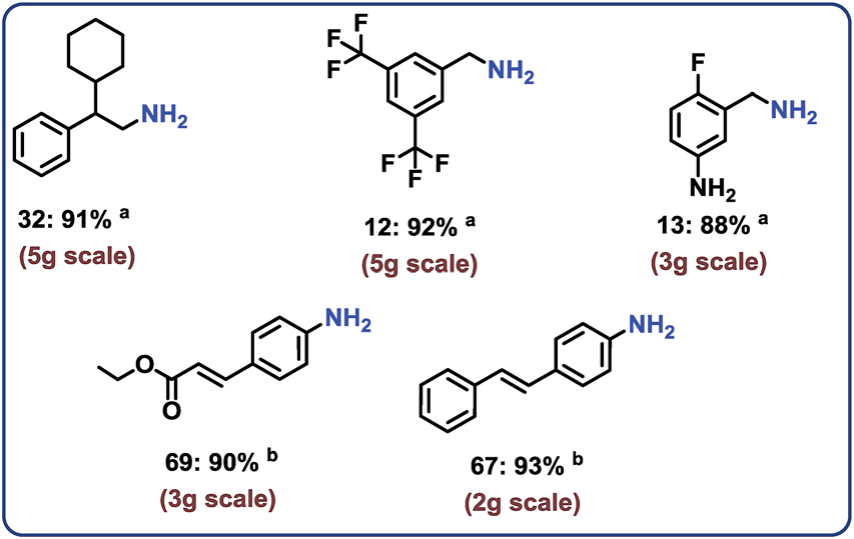
Representing the practical utility of the cobalt-catalyzed hydrogenation protocol for gram scale reactions. Reaction conditions: ^a^3–5 g nitrile, 25 mg catalyst (3.8 mol% Co) for each for each 0.5 mmol substrate, 25 bar H_2_, 5 bar NH_3_, 120 °C, 20–60 mL toluene 16 h, Isolated respective hydrochloride salts. ^b^2–3 g nitroarene 25 mg catalyst (3.8 mol% Co) for each 0.5 mmol substrate, 40 bar H_2_, 120 °C, 20–40 mL THF–H_2_O (1 : 1), 20 h, isolated yields.

To complement the synthetic viability of the catalyst system and the developed protocols, upscaling of selected reactions and recycling of catalyst are presented. The hydrogenation of three nitriles two nitroarenes on gram-scale (2–5 g) proceeded easily and in all cases similar yields to mg-scale reactions were obtained (Fig. 7).

**Fig. 8:**
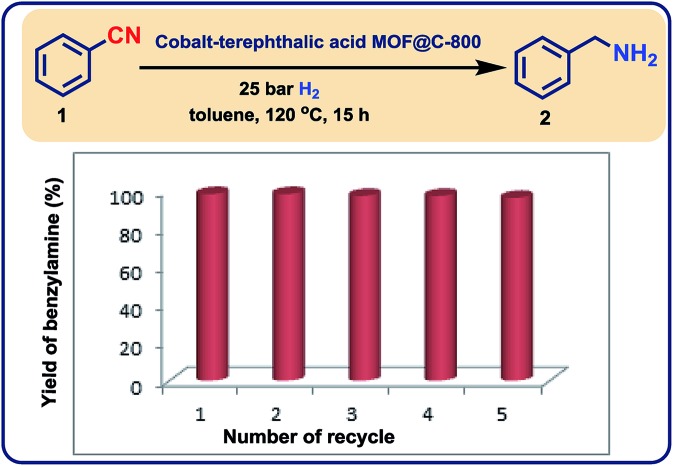
Demonstrating the recycling of cobalt-terephthalic acid MOF@C-800-catalyst for the hydrogenation of benzonitrile to benzylamine. Reaction condition: 5 mmol benzonitrile, 250 mg catalyst, (3.8 mol% Co), 20 mL toluene, 25 bar H_2_, 5 bar NH_3_, 120 °C, 16 h, GC yields using *n*-hexadecane standard.

In addition, these cobalt oxide nanoparticles proved to be highly stable in the benchmark hydrogenation and were recycled and reused for 5 times without significant loss of catalytic activity (Fig. 8). TEM analysis of the reused catalyst showed that there is no change in the structure of metallic Co particles (Fig. S5 and S6†). However, a change in the structure of the cobalt oxide particles was observed. Some of these particles migrate out from graphene shells and form new structures on carbon support (Fig. S5 and S6†).

## Experimental

### General considerations

All nitriles and nitroarenes were obtained commercially from various chemical companies. Cobalt(ii) nitrate hexahydrate, terephthalic acid were obtained from Sigma-Aldrich. Dry toluene, 99.8% was obtained from across chemicals. Carbon powder, VULCAN® XC72R with Code XVC72R and CAS no. 1333-86-4 was obtained from Cabot Corporation Prod. The pyrolysis experiments were carried out in Nytech-Qex oven. Before using, the purity of nitriles and nitroarenes has been checked.

The TEM measurements were performed at 200 kV with an aberration-corrected JEM-ARM200F (JEOL, Corrector: CEOS). The microscope is equipped with a JED-2300 (JEOL) energy-dispersive X-ray-spectrometer (EDXS). The aberration corrected STEM imaging (High-Angle Annular Dark Field (HAADF) and Annular Bright Field (ABF) were performed under the following conditions. HAADF and ABF both were done with a spot size of approximately 0.1 nm, a convergence angle of 30–36° and collection semi-angles for HAADF and ABF of 90–170 mrad and 11–22 mrad respectively. The solid samples were deposed without any pretreatment on a holey carbon supported Cu-grid (mesh 300) and transferred to the microscope.

XRD powder pattern were recorded either on a Panalytical X'Pert diffractometer equipped with a Xcelerator detector or on a Panalytical Empyrean diffractometer equipped with a PIXcel 3D detector system. Both were used with automatic divergence slits and Cu kα1/α2 radiation (40 kV, 40 mA; *λ* = 0.015406 nm, 0.0154443 nm). Cu β-radiation was excluded by using nickel filter foil. Peak positions and profile were fitted with pseudo-Voigt function using the HighScore Plus software package (Panalytical). Phase identification was done by using the PDF-2 database of the International Center of Diffraction Data (ICDD).

EPR spectra were recorded in X-band at 273 K on a Adani Spinscan X-band electron paramagnetic resonance (EPR) spectrometer equipped with cavity *Q* factor and MW power measurement with a magnetic field modulation capability of 10–250 kHz. The data were measured at microwave frequency = 9.48 GHz; modulation amplitude = 8 G; modulation frequency = 100 kHz as reported in the reference.

XPS data was obtained with a VG ESCALAB220iXL (ThermoScientific) with monochromatic Al Kα (1486.6 eV) radiation. The electron binding energies EB were obtained without charge compensation. For quantitative analysis the peaks were deconvoluted with Gaussian–Lorentzian curves, the peak area was divided by a sensitivity factor obtained from the element specific Scofield factor and the transmission function of the spectrometer.

All catalytic experiments were carried out in 300 mL and 100 mL autoclaves (PARR Instrument Company). In order to avoid unspecific reactions, all catalytic reactions were carried out either in glass vials, which were placed inside the autoclave, or glass/Teflon vessel fitted autoclaves.

GC and GC-MS were recorded on Agilent 6890N instrument. GC conversion and yields were determined by GC-FID, HP6890 with FID detector, column HP530 m × 250 mm × 0.25 μm.

^1^H, ^13^C NMR data were recorded on a Bruker ARX 300 and Bruker ARX 400 spectrometers using DMSO-d6, CD_3_OD and CDCl_3_ solvents.

### Procedure for the preparation of cobalt-terephthalic acid MOFs–carbon template and graphene shell encapsulated cobalt-based nanoparticles

A 50 mL dried round bottomed flask was charged with magnetic stirring bar and 1.52 mmol of cobalt(ii) nitrate hexahydrate and 4.58 mmol of terephthalic acid. Then 25–30 mL of DMF was added and stirred at 140–150 °C for 20–30 min. To this mixture, 1.5 g of carbon powder (VULCAN® XC72R) was added followed by the addition of 15 mL DMF and the reaction mixture again was stirred at 150 °C for 4–5 h by fixing reflux condenser. Then, the reflux condenser and magnetic stirring bar were removed and the round bottomed flask containing reaction products were. Then, the round bottomed flask containing reaction mixture was placed into an aluminium block preheated at 150 °C and allowed to stand without stirring and closing for 20–22 h at 150 °C in order to slow evaporation of DMF and to form Co–MOF–carbon template *via* solvothermal process. After complete drying, the material was cooled to room temperature and grinded to fine powder. The powdered material was pyrolyzed at 800 °C for 2 hours under an argon atmosphere and then cooled to room temperature after pyrolysis. (Elemental analysis (wt%): cobalt-terephthalic acid MOF@C-800: Co = 4.5, C = 90.70%, H = 0.25%.

### Procedure for the preparation and isolation of cobalt-terephthalic acid MOFs

A 50 mL dried round bottomed flask was charged with magnetic stirring bar, 0.52 mmol of cobalt(ii) nitrate hexahydrate and 4.58 mmol of terephthalic acid. Then 25–30 mL of DMF was added and stirred at 140–150 °C for 20–30 min. Then, the round bottomed flask containing reaction mixture was placed into an aluminium block preheated at 150 °C and stirred for 20–30 minutes by fixing reflux condenser. Then, the reflux condenser and magnetic stirring bar were removed and the round bottomed flask containing reaction products were allowed to stand without stirring and closing for 20–24 h at 150 °C in order to slow evaporation of DMF and to form Co–MOFs *via* solvothermal process. Afterward the product obtained was cooled down to room temperature and washed with hot DMF and then dried at 150 °C and at high vacuum.

### General procedure for the hydrogenation of nitriles

The magnetic stirring bar and 0.5 mmol corresponding nitrile compound was transferred to 8 mL glass vial and then 3 mL solvent (toluene*)* was added. Then, 25 mg of catalyst was added and the vial was fitted with septum, cap and needle. The reaction vials (8 vials with different substrates at a time) were placed into a 300 mL autoclave. The autoclave was flushed with hydrogen twice at 25 bar pressure and then it was pressurized with 5 bar ammonia gas and 25 bar hydrogen. The autoclave was placed into an aluminium block preheated at 130 °C (placed 30 minutes before counting the reaction time in ordered to attain reaction temperature) and the reactions were stirred for required time. During the reaction the inside temperature of the autoclave was measured to be 120 °C and this temperature was used as the reaction temperature. After the completion of the reactions, the autoclave was cooled to room temperature. The remaining ammonia and hydrogen were discharged and the vials containing reaction products were removed from the autoclave. The catalyst was filtered off and washed with ethyl acetate. The filtrate containing product was subjected to evaporation to remove solvent and ammonia. The crude product was diluted with ether and 1–2 mL methanol HCl or dioxane HCl (1.5 M HCl in methanol or 4 N HCl in dioxane) was added and stirred at room temperature for 4–5 h to obtain hydrochloride salt of amine. Then, the solvent was removed and the resulted hydrochloride salt of amine is dried under high vacuum. For determining the yields using GC analysis, after completing the reaction *n*-hexadecane (100 μL) as standard was added to the reaction vials and the reaction products were diluted with ethyl acetate followed by filtration using plug of silica and then analyzed by GC.

### General procedure for the hydrogenation of nitro compounds

The magnetic stirring bar and corresponding nitro compound (0.5 mmol) were transferred to the glass vial and then 3 mL solvent (water–THF or tertiary butanol) was added. Then, 25 mg of catalyst was added and the vial was fitted with septum, cap and needle. The reaction vials (8 reaction vials at a time in case of 0.5 mmol scale) were placed into a 300 mL autoclave. The autoclave was flushed with hydrogen twice at 30 bar pressure and then it was pressurized to 20 bar or 40 bar hydrogen pressure. The autoclave was placed into an aluminium block (placed 30 minutes before counting the reaction time in ordered to attain reaction temperature) preheated at 130 °C and the reactions were stirred for required time. During the reaction the inside temperature of the autoclave was measured to be 120 °C and this temperature was used as the reaction temperature. After the completion of the reaction, the autoclave was cooled to room temperature. The remaining hydrogen was discharged and the samples were removed from the autoclave. Catalyst from the reaction mixture was filtered off and washed with THF and then ethyl acetate. The solvent form filtrate containing reaction product was removed *in vacuo*. The corresponding aniline was purified by column chromatography (silica; *n*-hexane-ethyl acetate mixture). Then, dried over anhydrous Na_2_SO_4_ and the solvent was removed *in vacuo*. For determining the yields using GC analysis, after completing the reaction *n*-hexadecane (100 μL) as standard was added to the reaction vials and the reaction products were diluted with THF followed by filtration using plug of silica and then analyzed by GC.

### Procedure for up-scaling the reactions

#### For the hydrogenation of nitriles

To a Teflon or glass fitted 300 mL or 1.0 L autoclave, the magnetic stirring bar and corresponding nitriles were transferred and then 120–150 mL of dry toluene was added. Then after, required amount of catalyst (25 mg for each 0.5 mmol substrate) was added. The autoclave was flushed with hydrogen twice at 30 bar pressure and then it was pressurized with 5 bar ammonia gas and 25 bar hydrogen. The autoclave was placed into an aluminium block preheated at 130 °C (placed 30 minutes before counting the reaction time in ordered to attain reaction temperature) and the reaction was stirred for required time. During the reaction the inside temperature of the autoclave was measured to be 120 °C and this temperature was used as the reaction temperature. After the completion of the reaction, the autoclave was cooled to room temperature. The remaining ammonia and hydrogen were discharged and the reaction products were removed from the autoclave. The solid catalyst was filtered off and washed thoroughly with ethyl acetate. The reaction products were analyzed by GC-MS and the corresponding primary amines were converted to their respective hydrochloride salt and characterized.

#### For the hydrogenation of nitroarenes

To a Teflon or glass fitted 300 mL or 1.0 L autoclave, the magnetic stirring bar and corresponding nitrocompounds were transferred and then 120–150 mL of THF–H_2_O was added. Then after, required amount of catalyst (25 mg for each 0.5 mmol substrate) was added. The autoclave was flushed with hydrogen twice at 30 bar pressure and 40 bar hydrogen. The autoclave was placed into an aluminium block preheated at 130 °C (placed 30 minutes before counting the reaction time in ordered to attain reaction temperature) and the reaction was stirred for required time. During the reaction the inside temperature of the autoclave was measured to be 120 °C and this temperature was used as the reaction temperature. After the completion of the reaction, the autoclave was cooled to room temperature. The remaining hydrogen was discharged and the reaction products were removed from the autoclave. The solid catalyst was filtered off and washed thoroughly with ethyl acetate. The reaction products were analyzed by GC-MS and the corresponding anilines were isolated.

### Procedure for catalyst recycling

The magnetic stirring bar and 5 mmol benzonitrile were transferred to glass fitted 100 mL autoclave and then 20 mL dry toluene was added. Then after, 250 mg of catalyst was added. The autoclave was flushed with 30 bar hydrogen and then it was pressurized with 5 bar ammonia gas and 25 bar hydrogen. The autoclave was placed into heating system and reactions were allowed to progress at 120 °C (temperature inside the autoclave) by stirring for required time. After the completion of the reaction, the autoclave was cooled and the remaining ammonia and hydrogen were discharged. To the reaction products, 250 μL *n*-hexadecane as standard was added. The catalyst was then separated by centrifugation and the centrifugate containing reaction products was subjected to GC analysis for determining the yield of benzyl amine. The separated catalyst by centrifugation was then washed with ethyl acetate, dried under vacuum and used without further purification or reactivation for the next run.

## Conclusions

In conclusion, we prepared cobalt nanoparticles encapsulated in graphene shells supported on carbon by pyrolysis of simple cobalt salts and terephthalic acid (TPA). Compared to other recently developed nano-catalysts, this Co–TPA–carbon catalyst does not require nitrogen-doping for activity, which opens new avenues for catalyst design. The presented material constitutes a general base-metal hydrogenation catalyst, which allows for selective reduction of a series of functionalized and structurally diverse aromatic, heterocyclic and aliphatic nitriles, as well as nitro compounds. The synthetic utility and practicability of this cobalt-based hydrogenation protocol was further established by performing gram-scale synthesis and recycling of the catalyst.

## Conflicts of interest

There are no conflicts to declare.

## Supplementary Material

Supplementary information
