# Supplementary material for: Cobalt-based nanoparticles prepared from MOF–carbon templates as efficient hydrogenation catalysts
Source: Chem Sci. 2018 Sep 20;9(45):8553–60. doi: 10.1039/c8sc02807a (PMC6251336; doi:10.1039/c8sc02807a)
Supplement: Supplementary file 1 [file SC-009-C8SC02807A-s001.pdf]

Electronic Supplementary Material (ESI) for Chemical Science.
This journal is © The Royal Society of Chemistry 2018

Supporting Information

Cobalt-based nanoparticles prepared from MOF-carbon templates as efficient hydrogenation catalysts

Kathiravan Murugesan, Thirusangumurugan Senthamarai, Manzar Sohial, Ahmad S. Alshammari, Marga-Martina Pohl, Matthias Beller* and Rajenahally V. Jagadeesh*

Leibniz-Institut für Katalyse e. V. an der Universität Rostock, Albert-Einstein-Str. 29a, 18059 Rostock, Germany.

*Corresponding authors: Matthias.Beller@catalysis.de and Jagadeesh.Rajenahally@catalysis.de

Supplementary figures

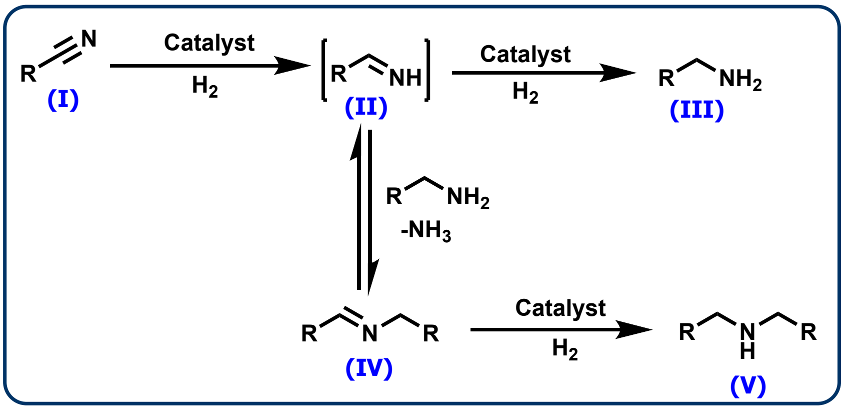

Fig. S1. Reaction pathway for the catalytic hydrogenation of nitriles: Formation of desired and un-desired products.

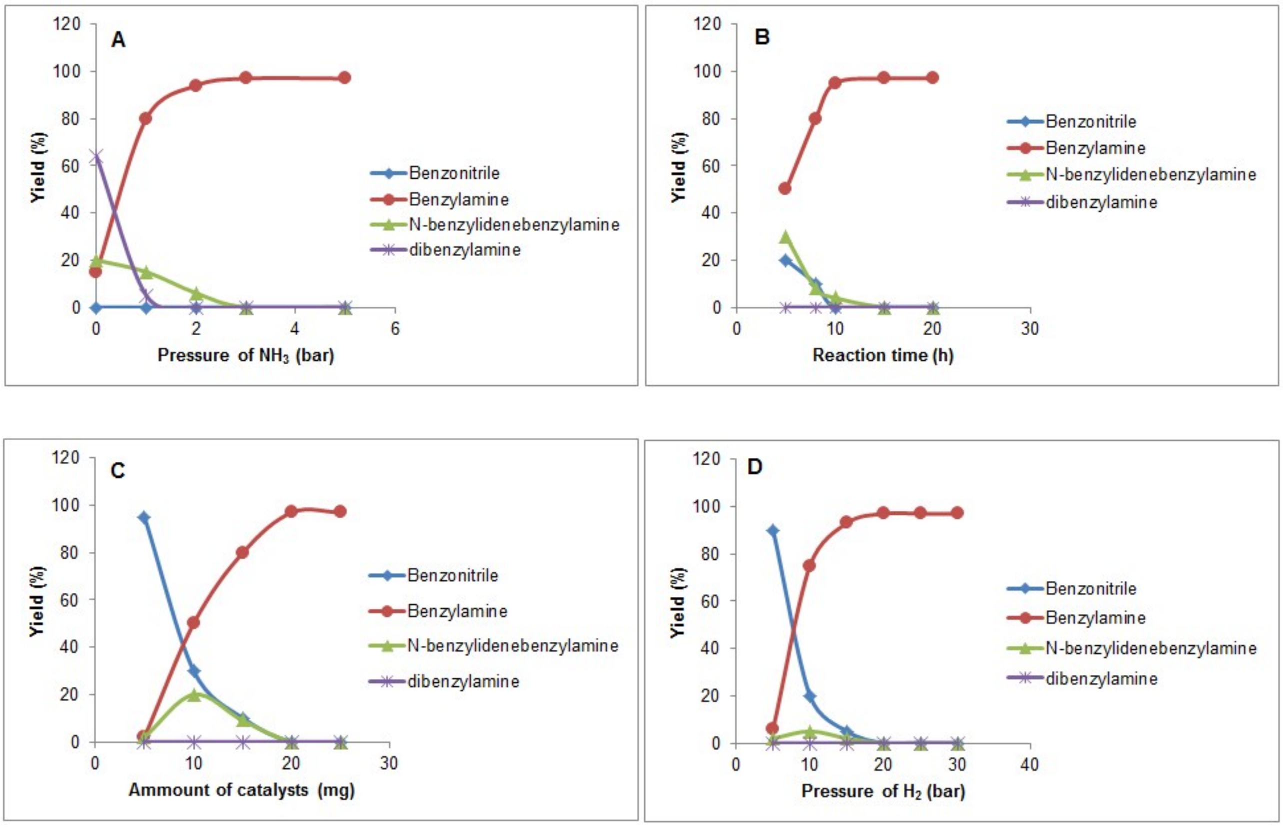

Fig. 2. Studies on the effect of ammonia (A), reaction time (B), amount of catalyst (C) and pressure of hydrogen (D) on the hydrogenation of benzonitrile. Reaction conditions: A: 0.5 mmol benzonitrile, 25 mg catalyst (3.8 mol% Co), 3 mL toluene, 25 bar H_2, 0-5 bar NH_3, 120 ^{0}C, 16 h. B: same as A with 5 bar NH_3, 120 ^{o}C, 0-20 h. C: Same as A with 5-25 mg catalyst and 5 bar NH_3. D: Same as A with 5-30 bar H_2 and 5 bar NH_3.

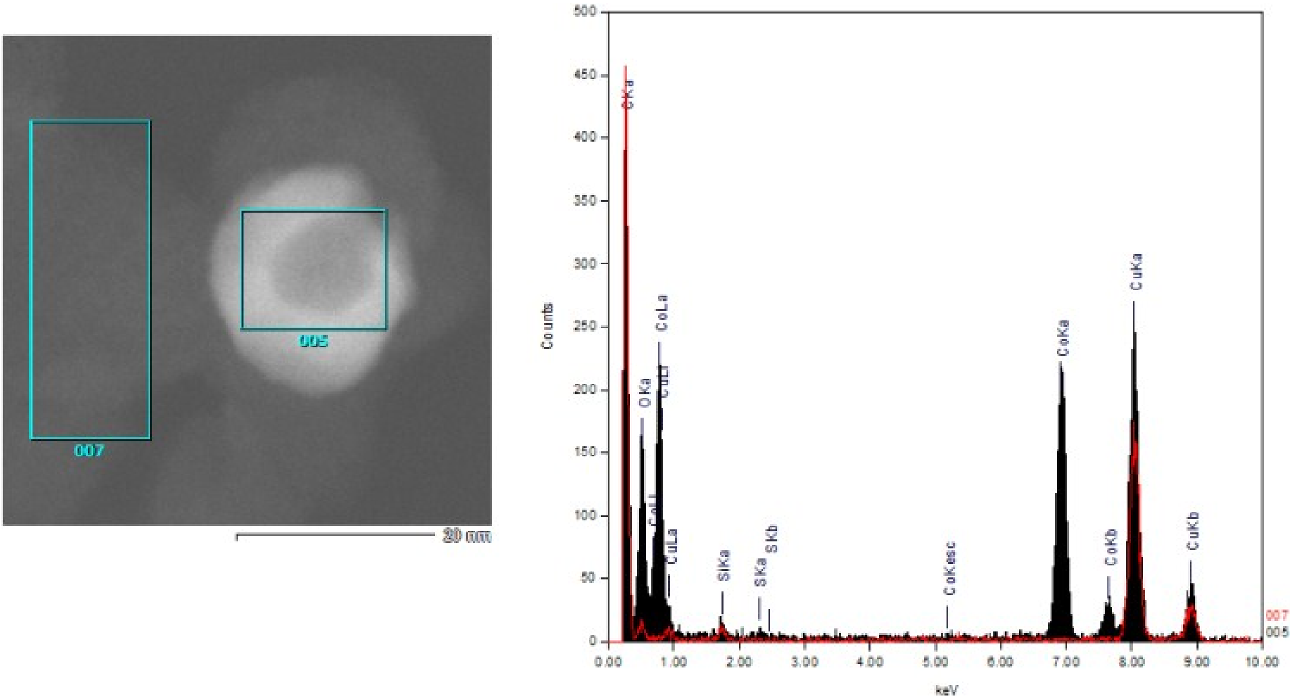

Fig. S3. HAADF-STEM/EDX images of cobalt-terephthalic acid MOF@C-800- catalyst.

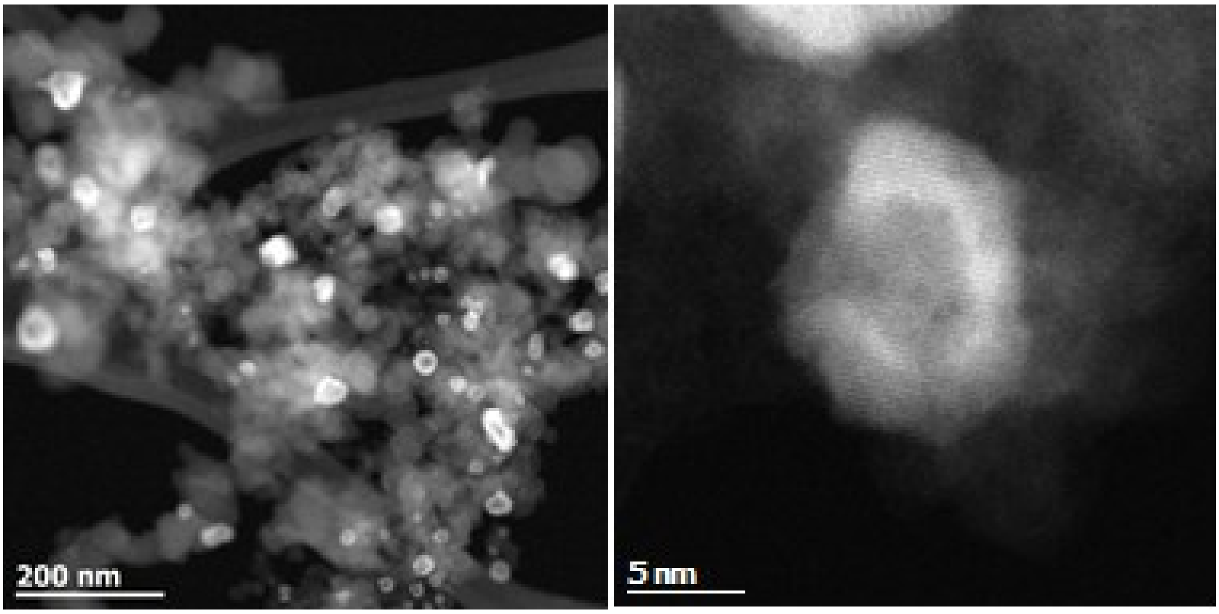

Fig. S4. TEM images cobalt nitrate@C-800.

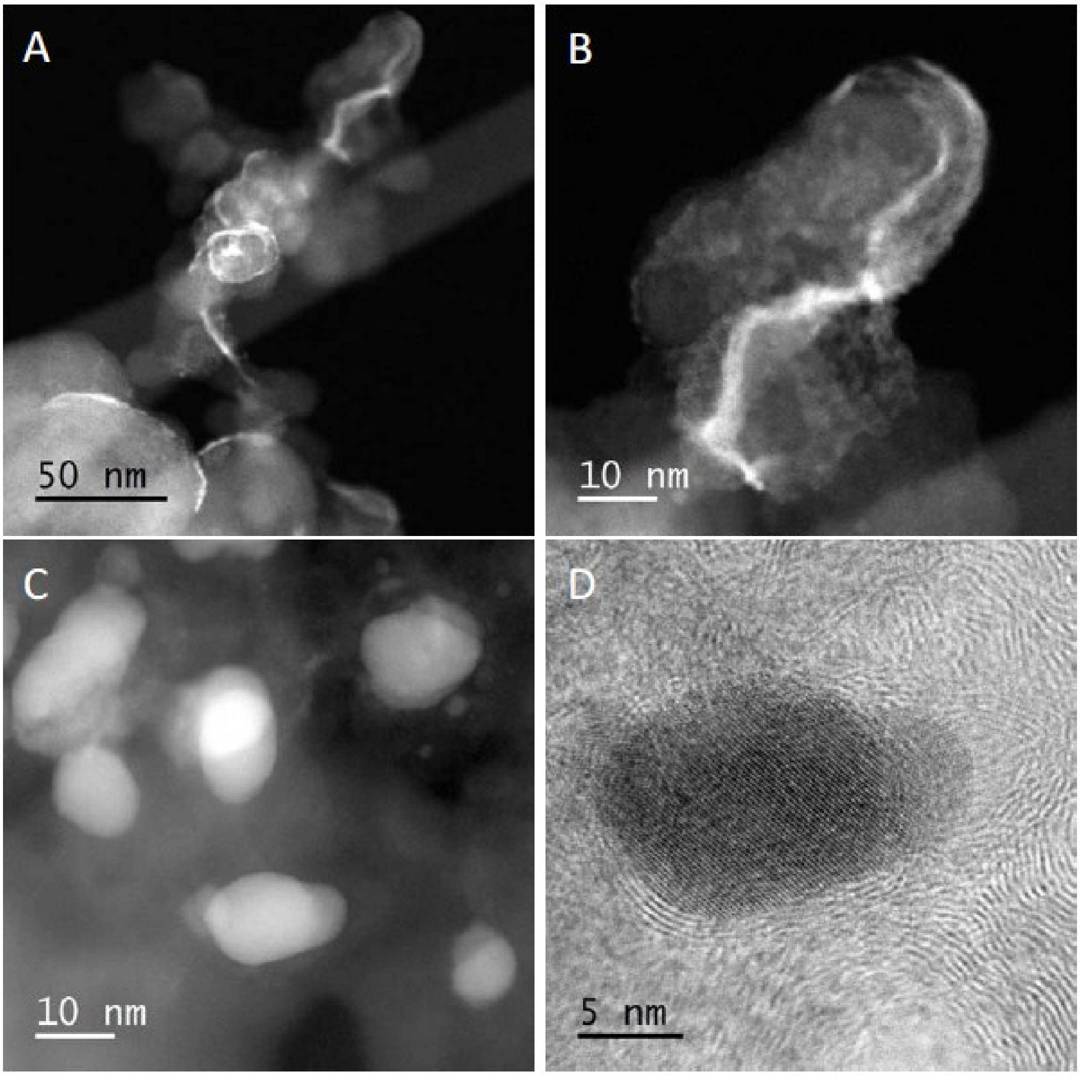

Fig. S5: HAADF-STEM images (A-C) and ABF-STEM image (D) of used catalyst. (A) and (B) show the new Co_3O_4 structures grown during reaction on the surface of the carbon support. (C) shows Co metal and Co_3O_4 particles. (D) shows a high resolution ABF image of a metal Co particle (see EDXS from S6) highlighting the encapsulation with several layers of graphenes.

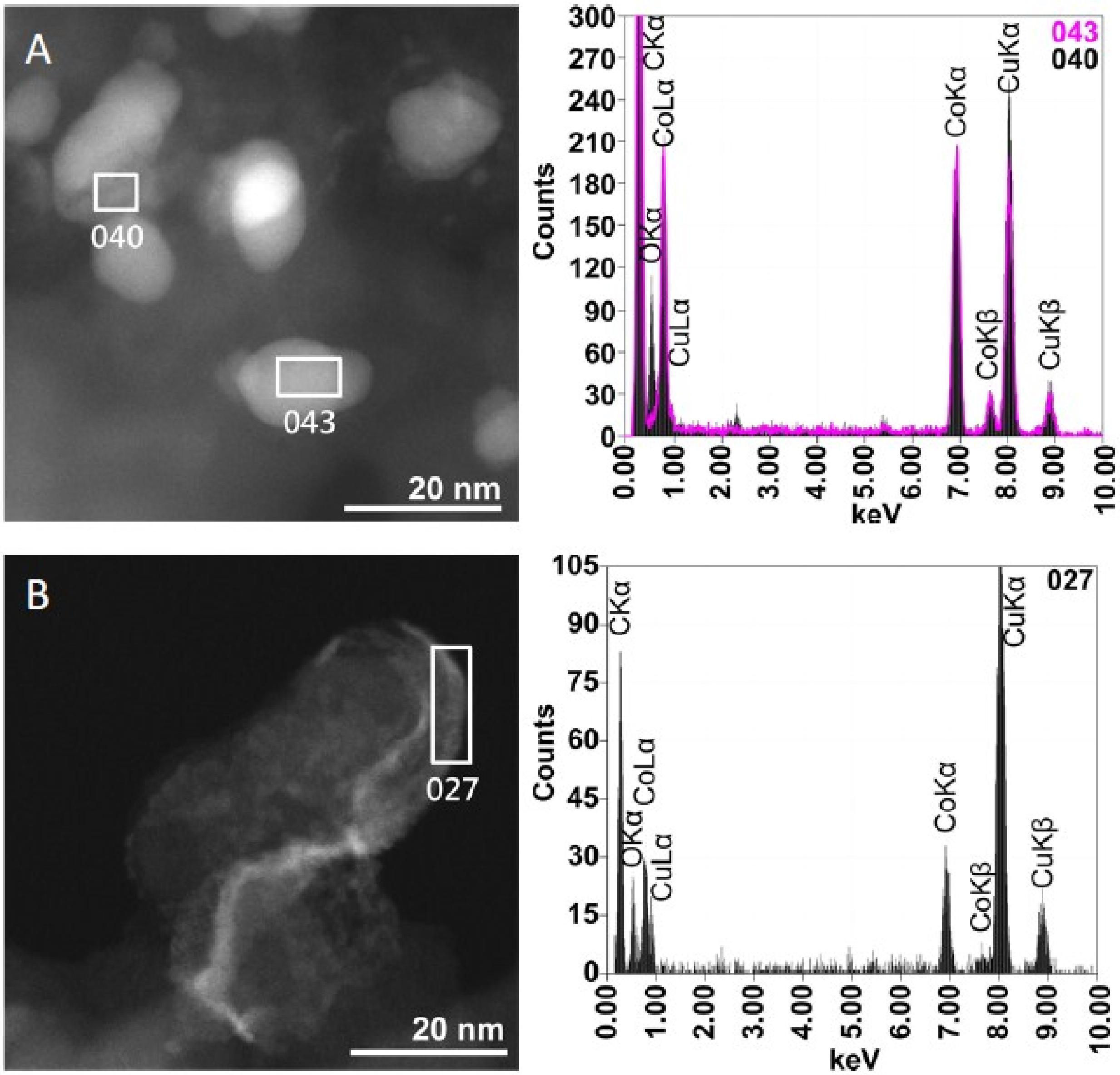

Fig. S6: HAADF-STEM images with EDX spectra from marked areas showing morphology comparable to the fresh specimen in the case of metallic Co particles (A, area 043) and changes in the case of cobalt oxide structures (A, area 040). Additionally a cobalt oxide phase has grown on the surface of carbon support (B, area 027).

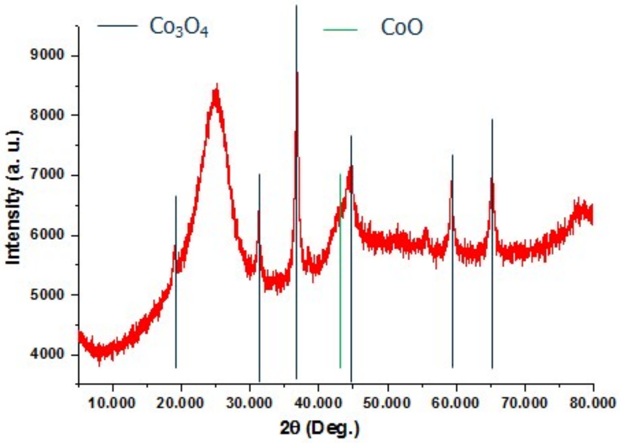

Fig. S7. XRD spectra of cobalt-terephthalic acid MOF@C-800 catalyst.

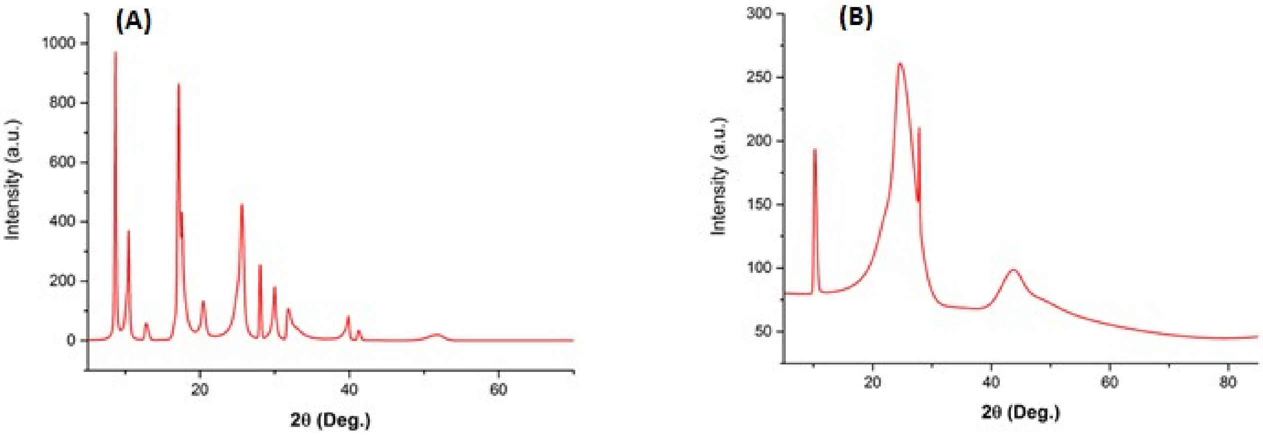

Fig. S8. XRD spectra of (A) Cobalt-terephthalic acid MOF and cobalt-terephthalic acid MOF-carbon template.

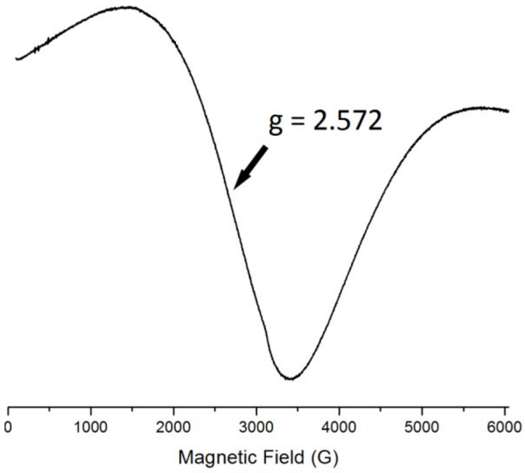

Fig. S9. EPR spectra of cobalt-terephthalic acid MOF@C-800 catalyst.

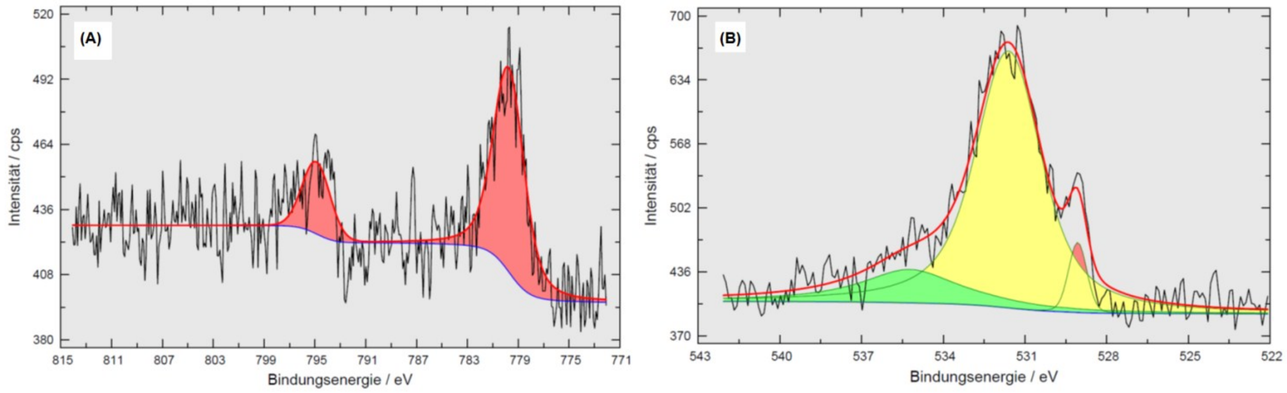

Fig. S10. XPS of Co2p (A) and O1s (B) electrons for cobalt-terephthalic acid MOF@C-800.

**Analysis**				**Report**			
**Operator:**	Struve	**Date:**2017/11/14		**Operator:** Struve		**Date:**2017/11/16	
**Sample ID:**	MK-34B	**Filename:**		MK-34B.qps			
**Sample Desc:**	Co3O4/Carbon, pressed, fresh, black	**Comment:**		120°C/0,5h, 220°C/2h, Thiru			
**Sample weight:**	0.0894 g	**Sample Volume:**		1 cc			
**Outgas Time:**	2.0 hrs	**OutgasTemp:**		220.0 C			
**Analysis gas:**	Nitrogen	**Bath Temp:**		273.0 K			
**Press. Tolerance:**	0.100/0.100 (ads/des)	**Equil time:**		20/20 sec (ads/des)	**Equil timeout:**	240/240 sec (ads/des)	
**Analysis Time:**	240.7 min	**End of run:**		2017/11/14 22:34:19	**Instrument:**	Nova Station A	
**Cell ID:**	0						

Multi-Point BET Plot

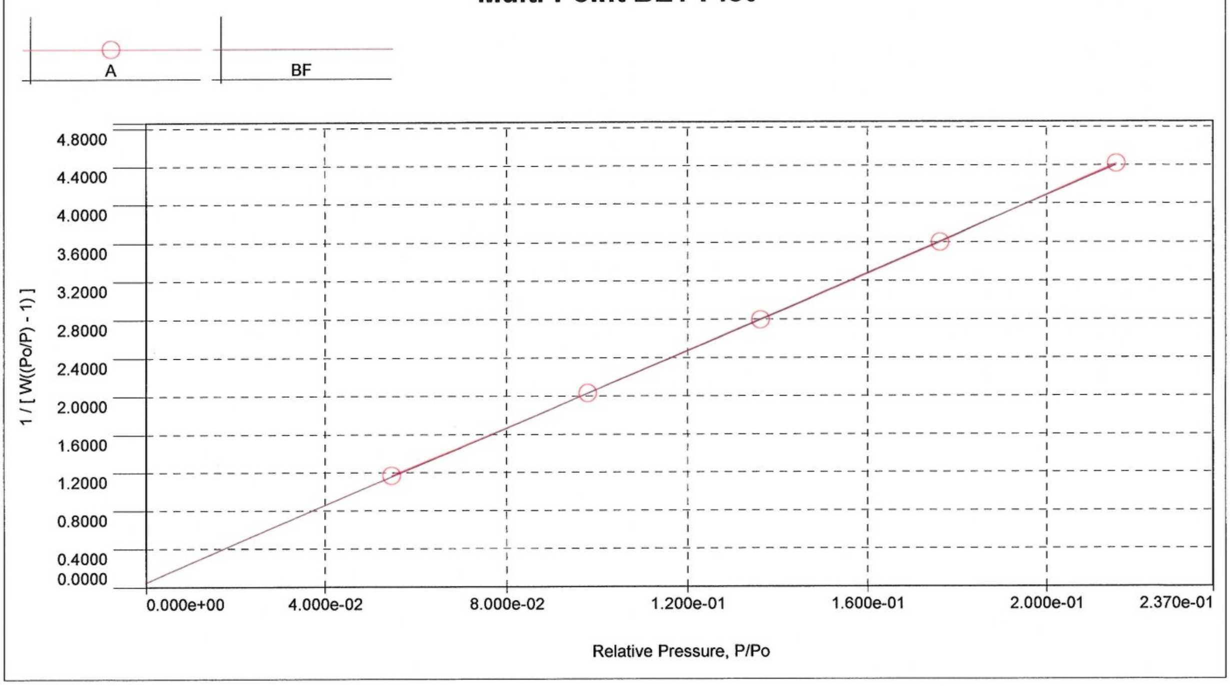

Multi-Point BET

Relative Pressure [P/Po]	Volume @ STP [cc/g]	1 / [W((Po/P) - 1)]	Relative Pressure [P/Po]	Volume @ STP [cc/g]	1 / [W((Po/P) - 1)]
5.46290e-02	39.7848	1.1621e+00	1.76050e-01	47.5872	3.5925e+00
9.79640e-02	42.9322	2.0240e+00	2.15359e-01	49.7683	4.4125e+00
1.36020e-01	45.1916	2.7874e+00			

MBET summary

**Slope =**	20.194
**Intercept =**	4.918e-02
**Correlation coefficient, r =**	0.999959
**C constant=**	411.620
**Surface Area =**	172.031 m²/g

Single Point Surface Area

Relative Pressure [P/Po]	Volume @ STP [cc/g]	1 / [W((P/Po) - 1)]	Slope	Surf. Area [m²/g]
2.98052e-01	54.6741	6.2138e+00	20.8480	167.0434

Fig. S9. Sorption isotherm of cobalt-terephthalic acid MOF@C-800 catalyst.

Analysis				Report			
Operator:	Struve	Date:	2017/11/14	Operator:	Struve	Date:	2017/11/16
Sample ID:	MK-34B	Filename:	MK-34B.qps				
Sample Desc:	Co3O4/Carbon, pressed, fresh, black	Comment:	120°C/0,5h, 220°C/2h, Thiru				
Sample weight:	0.0894 g	Sample Volume:	1 cc				
Outgas Time:	2.0 hrs	OutgasTemp:	220.0 C				
Analysis gas:	Nitrogen	Bath Temp:	273.0 K				
Press. Tolerance:	0.100/0.100 (ads/des)	Equil time:	20/20 sec (ads/des)	Equil timeout:	240/240 sec (ads/des)		
Analysis Time:	240.7 min	End of run:	2017/11/14 22:34:19	Instrument:	Nova Station A		
Cell ID:	0						

BJH method Desorption dV()

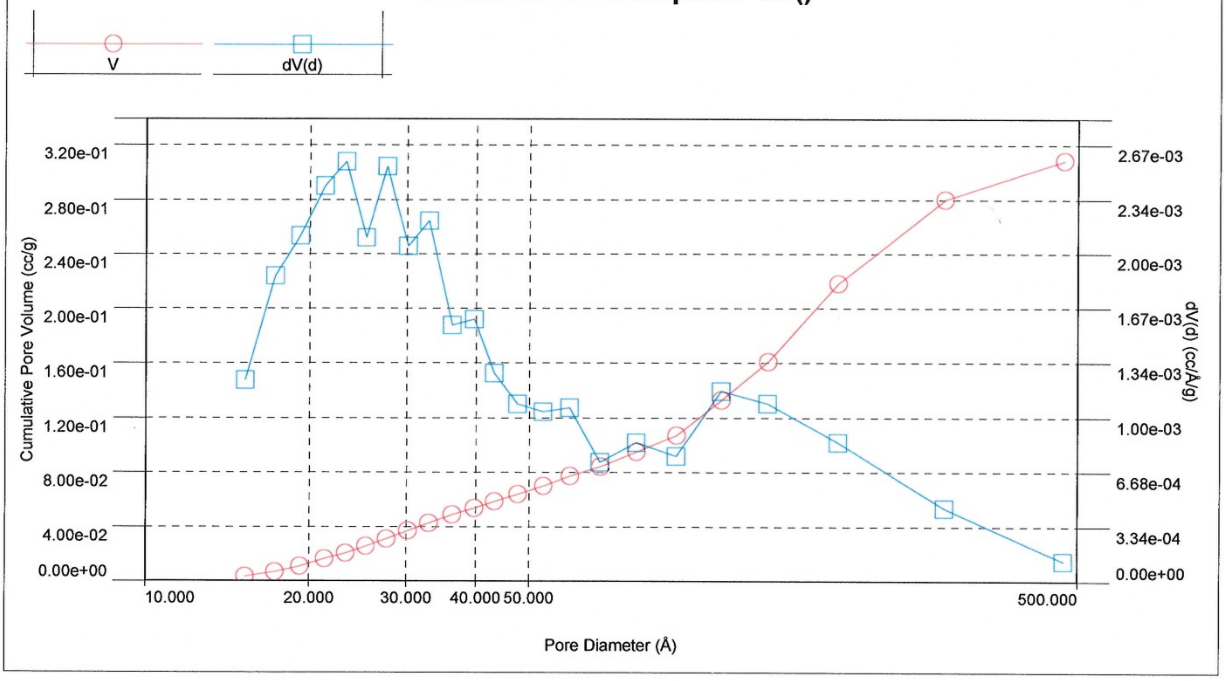

Data Reduction Parameters

t-Method	Calc. method: de Boer				
BJH/DH method	Moving pt. avg.: off				
Adsorbate	Nitrogen	Temperature	77.350K		
	Molec. Wt.: 28.013	Cross Section:	16.200 Å²	Liquid Density:	0.808 g/cc

BJH desorption summary

Surface Area = 158.418 m²/g
Pore Volume = 0.308 cc/g
Pore Diameter Dv(d) = 23.270 Å

Total Pore Volume data

Total Pore Volume

Total pore volume = 3.426e-01 cc/g for
pores smaller than 2627.1 Å (Diameter)
at P/Po = 0.99265

Average PoreSize data

Average pore Diameter = 7.96658e+01 Å

Fig. S10. Pore-size distribution calculated from the desorption isotherm for Cobalt-terephthalic acid MOF@C-800 catalyst.

NMR Data

KM10-42

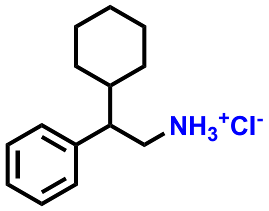

^{1}H NMR (300 MHz, DMSO-*d*$_6$) δ 8.10 (br s, 3H), 7.44 – 7.07 (m, 5H), 3.24 (dd, J = 12.8, 5.6 Hz, 1H), 3.05 (dd, J = 12.7, 9.4 Hz, 1H), 2.89 – 2.72 (m, 1H), 1.87 – 1.47 (m, 5H), 1.44 – 1.30 (m, 1H), 1.26 – 0.58 (m, 5H). **^{13}C NMR (75 MHz, DMSO-*d*$_6$)** δ 139.96 , 129.21 , 128.76 , 127.30 , 49.24 , 41.35 , 40.29 , 31.26 , 29.72 , 26.35 , 26.26 , 26.04 .**Off white solid.**

KM10-47

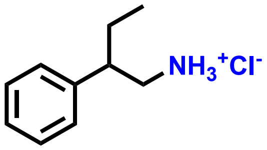

^{1}H NMR (300 MHz, DMSO-*d*$_6$) δ 8.24 (br s, 3H), 7.51 – 7.08 (m, 5H), 3.14 – 2.96 (m, 1H), 2.98 – 2.80 (m, 2H), 1.91 – 1.71 (m, 1H), 1.62 – 1.41 (m, 1H), 0.67 (t, *J* = 7.3 Hz, 3H). **^{13}C NMR (75 MHz, DMSO-*d*$_6$)** δ 141.49 , 129.06 , 128.36 , 127.37 , 45.26 , 44.17 , 26.28 , 11.88 . **Brown solid.**

KM10-48

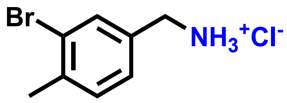

^{1}H NMR (300 MHz, DMSO-*d*$_6$) δ 8.67 (br s, 3H), 7.79 (d, *J* = 1.9 Hz, 1H), 7.49 – 7.32 (m, 2H), 3.99 (s, 2H), 2.35 (s, 3H). **^{13}C NMR (75 MHz, DMSO-*d*$_6$)** δ 137.79 , 134.39 , 133.02 , 131.50 , 128.92 , 124.43 , 41.53 , 22.58 . **White solid.**

KM10-52

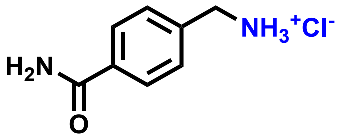

^{1}H NMR (300 MHz, DMSO-*d*$_6$) δ 8.68 (br s, 3H), 8.08 (br s, 1H), 7.92 (d, *J* = 8.3 Hz, 2H), 7.59 (d, *J* = 8.3 Hz, 2H), 7.43 (br s, 1H), 4.07 (s, 2H). **^{13}C NMR (75 MHz, DMSO-*d*$_6$)** δ 167.85 , 137.65 , 134.57 , 129.17 , 128.10 , 42.20 . **White solid.**

KM10-72

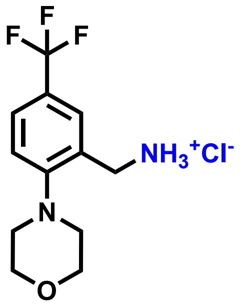

^{1}H NMR (300 MHz, DMSO-*d*$_6$) δ 8.80 (br s, 3H), 8.05 (d, *J* = 1.8 Hz, 1H), 7.70 (dd, *J* = 8.5, 1.7 Hz, 1H), 7.39 (d, *J* = 8.3 Hz, 1H), 4.17 (s, 2H), 3.86 – 3.68 (m, 4H), 2.94 – 2.82 (m, 4H). **^{13}C NMR (75 MHz, DMSO-*d*$_6$)** δ 155.02 , 130.46 , 127.02 (q, J = 3.5 Hz), 126.67 (q, J = 3.6 Hz), 124.73 (q, *J* = 271.7 Hz), 124.62 (q, *J* = 32.1 Hz), 121.37 , 66.76 , 52.82 , 37.77 . **Off white solid.**

KM10-73

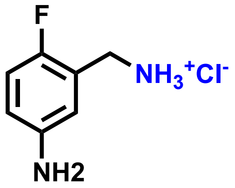

^{1}H NMR (300 MHz, DMSO-*d*$_6$) δ 10.50 (br s, 2H), 8.73 (br s, 3H), 7.61 (dd, *J* = 6.3, 2.5 Hz, 1H), 7.54 – 7.32 (m, 2H), 4.05 (s, 2H). **^{13}C NMR (75 MHz, DMSO-*d*$_6$)** δ 159.65 (d, *J* = 247.1 Hz), 129.36 , 126.28 (d, *J* = 3.6 Hz), 126.11 (d, *J* = 8.9 Hz), 122.89 (d, *J* = 16.5 Hz), 117.24 (d, *J* = 23.4 Hz), 35.90. **Off white solid.**

KM10-74

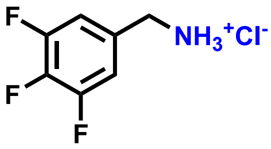

^{1}H NMR (300 MHz, DMSO-*d*$_6$) δ 8.80 (br s, 3H), 7.76 – 7.41 (m, 2H), 4.05 (s, 2H). **^{13}C NMR (75 MHz, DMSO-*d*$_6$)** δ 150.32 (ddd, *J* = 247.1, 9.7, 3.9 Hz), 139.00 (dt, *J* = 249.7, 15.5 Hz), 131.91 (td, *J* = 8.4, 4.8 Hz), 114.77 – 114.38 (m), 41.36. **Off white solid.**

KM10-77

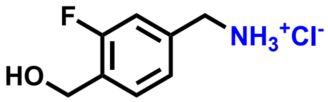

^{1}H NMR (300 MHz, DMSO-*d*$_6$) δ 8.68 (br s, 3H), 7.55 – 7.43 (m, 1H), 7.42 – 7.28 (m, 2H), 5.35 (br s, 1H), 4.54 (s, 2H), 4.01 (s, 2H). **^{13}C NMR (75 MHz, DMSO-*d*$_6$)** δ 159.68 (d, *J* = 244.1 Hz), 135.37 (d, *J* = 8.0 Hz), 129.74 (d, *J* = 15.0 Hz), 129.54 (d, *J* = 5.4 Hz), 125.28 (d, *J* = 3.1 Hz), 115.89 (d, *J* = 22.5 Hz), 55.49, 41.86. **Off white solid.**

KM10-24

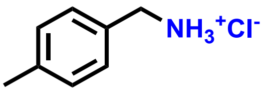

^{1}H NMR (300 MHz, DMSO-d_6) δ 8.62 (br s, 3H), 7.41 (d, J = 8.0 Hz, 2H), 7.20 (d, J = 7.9 Hz, 2H), 3.94 (s, 2H), 2.30 (s, 3H). **^{13}C NMR (75 MHz, DMSO-d_6)** δ 138.11 , 131.55 , 129.47 , 129.44 , 42.31 , 21.23 . **White solid.**

KM10-45

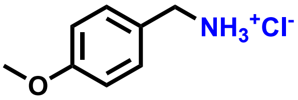

^{1}H NMR (300 MHz, DMSO-d_6) δ 8.66 (br s, 3H), 7.47 (d, J = 9.0 Hz, 2H), 6.94 (d, J = 8.7 Hz, 2H), 3.92 (s, 2H), 3.74 (s, 3H). **^{13}C NMR (75 MHz, DMSO-d_6)** δ 159.73 , 131.07 , 126.46 , 114.30 , 55.67 , 42.05 . **White solid.**

KM10-56

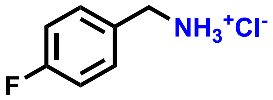

^{1}H NMR (300 MHz, DMSO-d_6) δ 8.72 (br s, 3H), 7.65 – 7.56 (m, 2H), 7.30 – 7.17 (m, 2H), 4.00 (s, 2H). **^{13}C NMR (75 MHz, DMSO-d_6)** δ 162.49 (d, J = 244.5 Hz), 131.89 (d, J = 8.4 Hz), 130.87 (d, J = 3.1 Hz), 115.73 (d, J = 21.5 Hz), 41.79. **White solid.**

KM10-57

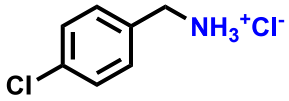

^{1}H NMR (300 MHz, DMSO-d_6) δ 8.72 (br s, 3H), 7.57 (d, J = 8.5 Hz, 2H), 7.48 (d, J = 8.6 Hz, 2H), 4.01 (s, 2H). **^{13}C NMR (75 MHz, DMSO-d_6)** δ 133.61 , 133.54 , 131.48 , 128.92 , 41.83 . **White solid.**

KM10-58

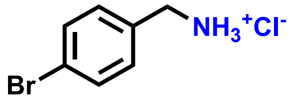

^{1}H NMR (300 MHz, DMSO-d_6) δ 8.73 (br s, 3H), 7.60 (d, J = 8.6 Hz, 2H), 7.51 (d, J = 8.7 Hz, 2H), 4.00 (s, 2H). **^{13}C NMR (75 MHz, DMSO-d_6)** δ 133.99 , 131.82 , 131.82 , 122.12 , 41.88 . **White solid.**

KM10-60

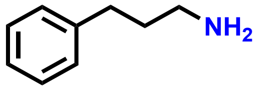

^{1}H NMR (300 MHz, DMSO-d_6) δ 7.47 – 6.93 (m, 5H), 3.44 (br s, 2H), 2.78 – 2.39 (m, 4H), 1.70 (t, *J* = 7.5 Hz, 2H). **^{13}C NMR (75 MHz, DMSO-d_6)** δ 142.49 , 128.72 , 128.68 , 126.07 , 41.05 , 34.34 , 32.97 . **Brown oil.**

KM10-61

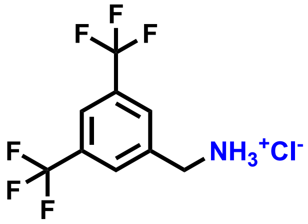

^{1}H NMR (300 MHz, DMSO-d_6) δ 8.92 (br s, 3H), 8.51 – 8.28 (m, 2H), 8.22 – 7.90 (m, 1H), 4.26 (s, 2H). **^{19}F NMR (282 MHz, DMSO-d_6)** δ -61.38 . **White solid.**

KM10-63

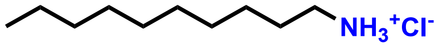

^{1}H NMR (300 MHz, DMSO-d_6) δ 5.66 (br s, 3H), 2.62 (t, *J* = 7.3 Hz, 2H), 1.45-1.42 (m, 2H), 1.25-1.22 (m, 14H), 0.86 (t, *J* = 6.6 Hz, 3H). **^{13}C NMR (75 MHz, DMSO-d_6)** δ 40.44, 31.80 , 30.25 , 29.48 , 29.47 , 29.28 , 29.22 , 26.67 , 22.58 , 14.36 . **Off white solid.**

KM10-83

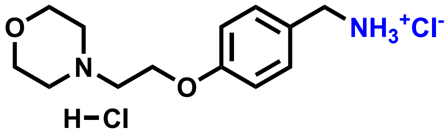

^{1}H NMR (300 MHz, DMSO-d_6) δ 11.99 (br s, 1H), 8.65 (br s, 3H), 7.49 (d, *J* = 8.7 Hz, 2H), 7.02 (d, *J* = 8.7 Hz, 2H), 4.37 (s, 2H), 4.01 – 3.78 (m, 6H), 3.62 – 3.36 (m, 4H), 3.34 – 3.14 (m, 2H). **^{13}C NMR (75 MHz, DMSO-d_6)** δ 157.98 , 131.12 , 127.38 , 115.15 , 63.55 , 62.94 , 55.12 , 52.08 , 41.98 . **Yellow solid.**

KM10-89

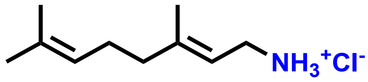

^{1}H NMR (300 MHz, DMSO-d_6) δ 5.40 (br s, 3H), 5.24 (t, *J* = 6.9 Hz, 1H), 5.06 (t, *J* = 6.0 Hz, 1H), 3.32 (d, *J* = 6.9 Hz, 2H), 2.14 – 1.83 (m, 4H), 1.66 – 1.57 (m, 6H), 1.54 (s, 3H). **^{13}C NMR (75 MHz, DMSO-d_6)** δ 138.69 , 131.21 , 124.21 , 122.15 , 48.84 , 39.55 , 26.43 , 25.76 , 17.79 , 16.36 . **Brown gum.**

KM10-95

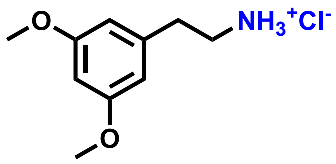

^{1}H NMR (300 MHz, DMSO-d_6) δ 7.96 (br s, 3H), 6.92 – 6.82 (m, 2H), 6.75 (dd, *J* = 8.2, 2.0 Hz, 1H), 3.74 (s, 3H), 3.71 (s, 3H), 3.11 – 2.94 (m, 2H), 2.93 – 2.78 (m, 2H). **13C NMR (75 MHz, DMSO-d6)** δ 149.22 , 148.03 , 130.41 , 120.97 , 112.97 , 112.44 , 56.00 , 55.93 , 40.62 , 33.19 .**Yellow solid.**

KM10-97

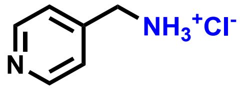

^{1}H NMR (300 MHz, DMSO-d_6) δ 8.82 (m, 5H), 7.78 – 7.43 (m, 2H), 4.07 (s, 2H). **^{13}C NMR (75 MHz, DMSO-d_6)** δ 150.14 , 143.40 , 124.51 , 41.41 . **Brown solid.**

KM10-98

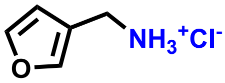

^{1}H NMR (300 MHz, DMSO-d_6) δ 8.28 (br s, 3H), 7.79 - 7368 (m, 2H), 6.73 (s, 1H), 3.91 (s, 2H). **^{13}C NMR (75 MHz, DMSO-d_6)** δ 144.15 , 142.37 , 119.32 , 111.59 , 33.83 . **Brown solid.**

KM10-101

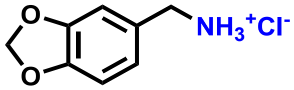

^{1}H NMR (300 MHz, DMSO-d_6) δ 8.65 (br s, 3H), 7.18 (s, 1H), 6.99 (d, *J* = 7.9 Hz, 1H), 6.92 (d, *J* = 7.7 Hz, 1H), 6.03 (s, 2H), 3.90 (s, 2H). **^{13}C NMR (75 MHz, DMSO-d_6)** δ 147.67 , 147.66 , 128.15 , 123.37 , 110.03 , 108.62 , 101.64 , 42.38 . **White solid.**

KM10-103

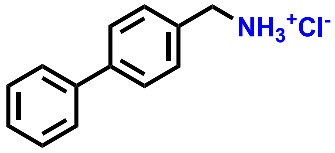

^{1}H NMR (300 MHz, DMSO-d_6) δ 8.78 (br s, 3H), 7.87 – 7.58 (m, 6H), 7.58 – 7.28 (m, 3H), 4.06 (s, 2H). **^{13}C NMR (75 MHz, DMSO-d_6)** δ 140.59 , 140.02 , 133.78 , 130.13 , 129.45 , 128.12 , 127.18 , 127.15 , 42.25 .

KM10-120

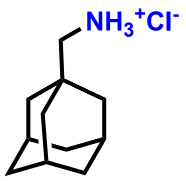

^{1}H NMR (300 MHz, DMSO-d_6) δ 5.33 (br s, 3H), 2.31 (s, 2H), 1.99 – 1.87 (m, 3H), 1.75 – 1.52 (m, 6H), 1.52 – 1.42 (m, 6H). **^{13}C NMR (75 MHz, DMSO-d_6)** δ 52.58 , 39.75 , 36.94 , 33.05 , 28.12 . **White solid.**

KM10-54

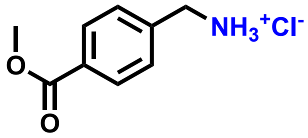

^{1}H NMR (300 MHz, DMSO-d_6) δ 8.82 (br s, 3H), 7.96 (d, J = 8.5 Hz, 2H), 7.68 (d, J = 8.5 Hz, 2H), 4.11 (s, 2H), 3.85 (s, 3H). **^{13}C NMR (75 MHz, DMSO-d_6)** δ 166.35 , 139.92 , 129.85 , 129.70 , 129.65 , 52.71 , 42.12 . **White solid.**

KM10-109

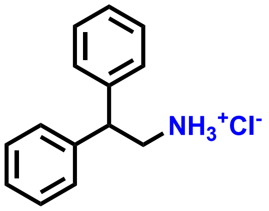

^{1}H NMR (300 MHz, DMSO-d_6) δ 8.20 (br s, 3H), 7.59 – 7.05 (m, 10H), 4.46 (t, J = 7.8 Hz, 1H), 3.52 (d, J = 7.8 Hz, 2H). **^{13}C NMR (75 MHz, DMSO-d_6)** δ 141.69 , 129.19 , 128.32 , 127.39 , 49.03 , 42.94 . **Off white solid.**

KM10-110

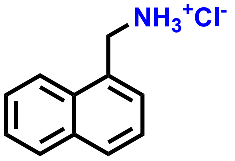

^{1}H NMR (300 MHz, DMSO-d_6) δ 8.41 (br s, 3H), 8.23 – 8.10 (m, 1H), 8.05 – 7.91 (m, 2H), 7.70 (dd, J = 7.1, 1.2 Hz, 1H), 7.67 – 7.44 (m, 3H), 4.50 (s, 2H). **^{13}C NMR (75 MHz, DMSO-d_6)** δ 133.64 , 131.12 , 130.95 , 129.29 , 129.07 , 127.55 , 127.14 , 126.62 , 125.82 , 123.95 , 39.75 . **Off white solid.**

KM10-124

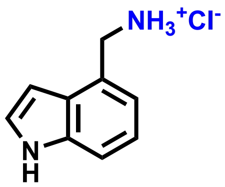

^{1}H NMR (300 MHz, DMSO-*d*$_6$) δ 11.50 (s, 1H), 8.66 (br s, 3H), 7.48 – 7.37 (m, 2H), 7.19 – 7.08 (m, 2H), 6.72 – 6.63 (m, 1H), 4.24 (s, 2H). **^{13}C NMR (75 MHz, DMSO-*d*$_6$)** δ 136.28 , 127.03 , 126.16 , 125.42 , 121.23 , 119.37 , 112.27 , 99.74 , 40.44 . **Off white solid.**

KM10-127

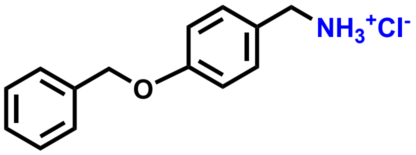

^{1}H NMR (300 MHz, DMSO-*d*$_6$) δ 8.59 (br s, 3H), 7.65 – 7.23 (m, 7H), 7.03 (d, *J* = 8.7 Hz, 2H), 5.13 (s, 2H), 3.92 (s, 2H). **^{13}C NMR (75 MHz, DMSO-*d*$_6$)** δ 158.75 , 137.42 , 131.05 , 128.90 , 128.29 , 128.08 , 126.72 , 115.24 , 69.62 , 42.04 . **White solid.**

KM10-128

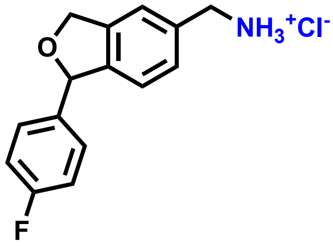

^{1}H NMR (300 MHz, DMSO-*d*$_6$) δ 8.67 (br s, 3H), 7.53 (s, 1H), 7.44 – 7.34 (m, 3H), 7.23 – 7.14 (m, 2H), 7.08 (d, *J* = 7.8 Hz, 1H), 6.19 (s, 1H), 5.30 (dd, *J* = 12.8, 2.6 Hz, 1H), 5.12 (d, *J* = 12.2 Hz, 1H), 4.02 (s, 2H). **^{13}C NMR (75 MHz, DMSO-*d*$_6$)** δ 162.16 (d, *J* = 243.4 Hz), 142.54 , 139.49 , 139.25 (d, *J* = 2.8 Hz), 134.29 , 129.05 , 128.90 (d, *J* = 8.3 Hz), 122.60 , 122.46 , 115.72 (d, *J* = 21.4 Hz), 84.47 , 72.85 , 42.38 . **White solid.**

KM10-138

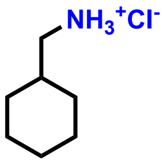

^{1}H NMR (300 MHz, DMSO-*d*$_6$) δ 8.20 (br s, 3H), 2.72 – 2.49 (m, 2H), 1.93 – 1.46 (m, 6H), 1.23 – 0.67 (m, 5H). **^{13}C NMR (75 MHz, DMSO-*d*$_6$)** δ 44.80 , 35.76 , 30.30 , 26.13 , 25.54 . **White solid.**

KM10-144

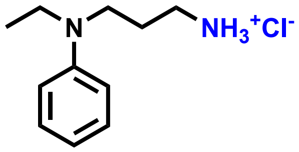

^{1}H NMR (300 MHz, DMSO-*d*$_6$) δ 7.67 (br s, 3H), 7.32 – 6.98 (m, 2H), 6.68 (d, *J* = 8.2 Hz, 2H), 6.66 – 6.41 (m, 1H), 3.45 – 3.14 (m, 4H), 2.94 – 2.67 (m, 2H), 1.99 – 1.68 (m, 2H), 1.04 (t, *J* = 6.9 Hz, 3H). **^{13}C NMR (75 MHz, DMSO-*d*$_6$)** δ 147.87 , 129.56 , 115.68 , 112.26 , 47.17 , 44.56 , 37.30 , 26.00 , 12.49 . **Off white solid.**

KM10-121

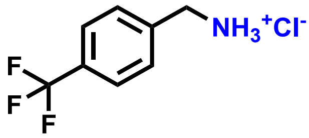

^{1}H NMR (300 MHz, DMSO-*d*$_6$) δ 8.85 (br s, 3H), 7.93 – 7.56 (m, 4H), 4.12 (s, 2H). **^{13}C NMR (75 MHz, DMSO-*d*$_6$)** δ 139.30 , 130.27 , 129.26 (q, *J* = 31.8 Hz), 125.76 , 124.59 (d, *J* = 272.1 Hz), 41.99 . **White solid.**

KM10-182

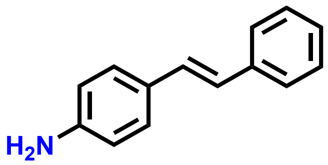

^{1}H NMR (300 MHz, Chloroform-*d*) δ 7.45 – 7.05 (m, 7H), 7.00 – 6.74 (m, 2H), 6.54 (d, *J* = 8.0 Hz, 2H), 3.42 (br s, 2H). **^{13}C NMR (75 MHz, Chloroform-*d*)** δ 146.22 , 138.02 , 128.77 , 128.67 , 128.06 , 127.81 , 126.96 , 126.18 , 125.16 , 115.28 . **Yellow solid.**

KM10-186

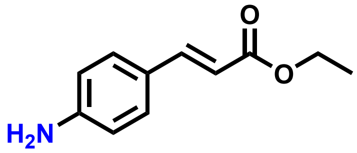

^{1}H NMR (300 MHz, Chloroform-*d*) δ 7.51 (d, *J* = 15.9 Hz, 1H), 7.24 (d, *J* = 8.2 Hz, 2H), 6.54 (d, *J* = 8.6 Hz, 2H), 6.14 (d, *J* = 15.9 Hz, 1H), 4.15 (q, *J* = 7.1 Hz, 2H), 3.82 (br s, 2H), 1.23 (t, *J* = 7.1 Hz, 3H). **^{13}C NMR (75 MHz, Chloroform-*d*)** δ 167.76 , 148.82 , 144.93 , 129.86 , 124.62 , 114.83 , 113.61 , 60.18 , 14.39 . **Yellow solid.**

KM10-200

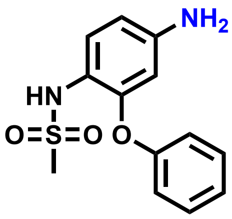

^{1}H NMR (300 MHz, Chloroform-*d*) δ 7.34 – 7.21 (m, 3H), 7.13 – 7.02 (m, 1H), 6.95 – 6.88 (m, 2H), 6.32 (dd, *J* = 8.6, 2.5 Hz, 1H), 6.08 (d, *J* = 2.5 Hz, 1H), 3.55 (br s, 2H), 2.82 (s, 3H). **^{13}C NMR (75 MHz, Chloroform-*d*)** δ 155.81 , 150.57 , 146.26 , 130.12 , 127.28 , 124.28 , 118.87 , 117.76 , 110.61 , 104.53 , 39.00 . **Brown solid.**

KM10-252

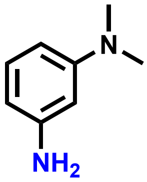

^{1}H NMR (300 MHz, Chloroform-*d*) δ 7.26 – 6.90 (m, 1H), 6.41 – 5.89 (m, 3H), 3.40 (br s, 2H), 2.96 (s, 6H). **^{13}C NMR (75 MHz, Chloroform-*d*)** δ 151.88 , 147.35 , 129.88 , 104.38 , 103.86 , 99.71 , 40.64 . **Brown gum.**

KM10-244

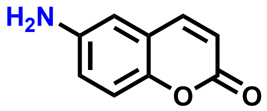

^{1}H NMR (300 MHz, DMSO-d_6) δ 7.85 (d, *J* = 9.5 Hz, 1H), 7.10 (d, *J* = 8.8 Hz, 1H), 6.86 (dd, *J* = 8.8, 2.7 Hz, 1H), 6.74 (d, *J* = 2.7 Hz, 1H), 6.35 (d, *J* = 9.5 Hz, 1H), 5.16 (br s, 2H). **^{13}C NMR (75 MHz, DMSO-d_6)** δ 160.98 , 145.91 , 145.74 , 144.77 , 119.51 , 119.30 , 116.98 , 116.29 , 110.81 . **Yellow solid.**

KM10-255

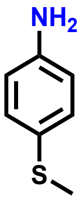

^{1}H NMR (300 MHz, Chloroform-*d*) δ 7.09 (d, *J* = 8.5 Hz, 2H), 6.53 (d, *J* = 8.5 Hz, 2H), 3.42 (br s, 2H), 2.32 (s, 3H). **^{13}C NMR (75 MHz, Chloroform-*d*)** δ 145.16 , 131.07 , 125.73 , 115.78 , 18.81 . **Brown gum.**

KM10-256

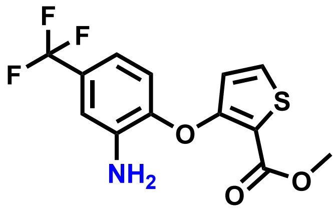

^{1}H NMR (300 MHz, Chloroform-*d*) δ 7.35 (d, *J* = 5.5 Hz, 1H), 6.99 – 6.92 (m, 1H), 6.87 – 6.79 (m, 2H), 6.66 (d, *J* = 5.5 Hz, 1H), 3.86 (br s, 2H), 3.77 (s, 3H). **^{13}C NMR (75 MHz, Chloroform-*d*)** δ 161.50 , 156.96 , 146.67 , 138.27 , 130.48 , 127.03 (q, *J* = 32.4 Hz), 124.06 (q, *J* = 271.8 Hz) , 120.89 , 117.96 , 117.50 , 115.18 (q, *J* = 3.9 Hz), 112.94 (q, *J* = 3.7 Hz), 51.98 . **Yellow gum.**

KM10-269

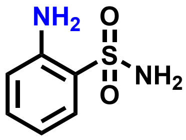

^{1}H NMR (300 MHz, DMSO-d_6) δ 7.55 (d, *J* = 7.8 Hz, 1H), 7.33 – 7.14 (m, 3H), 6.80 (dd, *J* = 8.2, 1.4 Hz, 1H), 6.67 – 6.54 (m, 1H), 5.84 (br s, 2H). **^{13}C NMR (75 MHz, DMSO-d_6)** δ 146.00 , 133.32 , 128.33 , 124.68 , 117.15 , 115.43 . **Off white solid.**

KM10-267

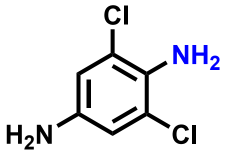

^{1}H NMR (300 MHz, Chloroform-*d*) δ 6.52 (s, 2H), 3.60 (br s, 4H). **^{13}C NMR (75 MHz, Chloroform-*d*)** δ 138.37 , 132.53 , 120.81 , 115.43 . **Brown solid.**

KM10-242

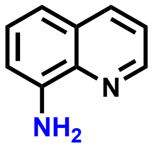

^{1}H NMR (300 MHz, Chloroform-*d*) δ 8.67 (dd, *J* = 4.2, 1.7 Hz, 1H), 7.96 (dd, *J* = 8.3, 1.7 Hz, 1H), 7.33 – 7.19 (m, 2H), 7.05 (dd, *J* = 8.2, 1.3 Hz, 1H), 6.83 (dd, *J* = 7.5, 1.3 Hz, 1H), 4.84 (br s, 2H). **^{13}C NMR (75 MHz, Chloroform-*d*)** δ 147.42 , 143.98 , 138.43 , 136.01 , 128.87 , 127.40 , 121.34 , 116.03 , 110.05 . **Brown solid.**

KM10-251

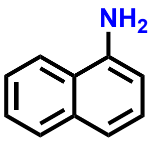

^{1}H NMR (300 MHz, Chloroform-*d*) δ 7.94 – 7.78 (m, 2H), 7.62 – 7.44 (m, 2H), 7.43 – 7.27 (m, 2H), 6.83 (dd, *J* = 6.6, 1.8 Hz, 1H), 4.16 (br s, 2H). **^{13}C NMR (75 MHz, Chloroform-*d*)** δ 142.15 , 134.46 , 128.61 , 126.40 , 125.90 , 124.90 , 123.72 , 120.86 , 119.02 , 109.74 . **Brown solid.**

KM10-277

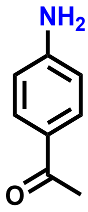

^{1}H NMR (300 MHz, Chloroform-*d*) δ 7.72 (d, *J* = 8.7 Hz, 2H), 6.56 (d, *J* = 8.7 Hz, 2H), 4.13 (br s, 2H), 2.42 (s, 3H). **^{13}C NMR (75 MHz, Chloroform-*d*)** δ 196.52 , 151.28 , 130.80 , 113.71 , 26.08 . **Off white solid.**

KM10-265

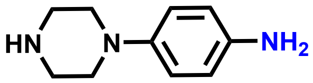

^{1}H NMR (300 MHz, Chloroform-*d*) δ 6.75 (d, *J* = 8.8 Hz, 2H), 6.57 (d, *J* = 8.7 Hz, 2H), 3.01 - 2.93 (m, 11H). **^{13}C NMR (75 MHz, Chloroform-*d*)** δ 144.87 , 140.33 , 118.61 , 116.14 , 52.17 , 46.18 . **Brown gum.**

KM10-268

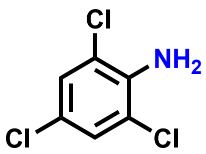

^{1}H NMR (300 MHz, Chloroform-*d*) δ 7.07 (s, 2H), 4.32 (br s, 2H). **^{13}C NMR (75 MHz, Chloroform-*d*)** δ 139.04, 127.59 , 121.85, 119.70 . **Brown solid.**

KM10-272

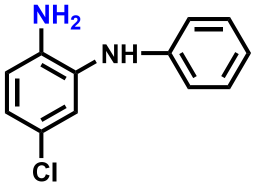

^{1}H NMR (300 MHz, Chloroform-*d*) δ 7.38 – 7.24 (m, 2H), 7.16 (d, *J* = 2.3 Hz, 1H), 7.06 – 6.90 (m, 2H), 6.83 (dt, *J* = 7.7, 1.1 Hz, 2H), 6.74 (d, *J* = 8.4 Hz, 1H), 5.34 (br s, 1H), 3.76 (br s, 2H). **^{13}C NMR (75 MHz,**

Chloroform-*d*) δ 144.33 , 139.69 , 130.42 , 129.53 , 124.77 , 123.45 , 123.23 , 120.27 , 117.15 , 116.15 . **Brown solid.**

KM10-270

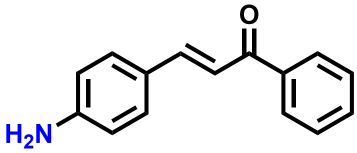

^{1}H NMR (300 MHz, Chloroform-*d*) δ 7.92 (d, *J* = 6.8 Hz, 2H), 7.67 (d, *J* = 15.6 Hz, 1H), 7.52 – 7.35 (m, 5H), 7.26 (d, *J* = 15.6 Hz, 1H), 6.59 (d, *J* = 8.5 Hz, 2H), 3.90 (br s, 2H). **^{13}C NMR (75 MHz, Chloroform-*d*)** δ 190.80 , 149.23 , 145.57 , 138.84 , 132.34 , 130.53 , 128.51 , 128.36 , 125.07 , 117.94 , 114.87 . **Yellow solid.**

KM10-270

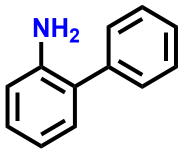

^{1}H NMR (300 MHz, Chloroform-*d*) δ 7.66 – 7.42 (m, 5H), 7.31 – 7.23 (m, 2H), 7.02 – 6.92 (m, 1H), 6.86 (dt, *J* = 7.3, 1.1 Hz, 1H), 3.78 (br s, 2H). **^{13}C NMR (75 MHz, Chloroform-*d*)** δ 143.67 , 139.71 , 130.59 , 129.23 , 128.95 , 128.64 , 127.73 , 127.29 , 118.75 , 115.75 . **Colorless gum**.

KM10-273

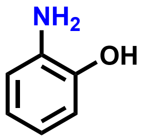

^{1}H NMR (300 MHz, DMSO-*d*$_6$) δ 8.97 (br s, 1H), 6.68 (ddd, *J* = 7.7, 1.4, 0.4 Hz, 1H), 6.65 – 6.54 (m, 2H), 6.43 (ddd, *J* = 7.7, 7.1, 2.0 Hz, 1H), 4.47 (br s, 2H). **^{13}C NMR (75 MHz, DMSO-*d*$_6$)** δ 144.47 , 136.94 , 120.02 , 117.00 , 114.97 , 114.88 . **Brown solid.**

KM10-281

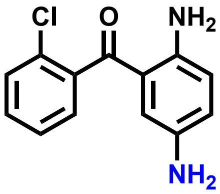

^{1}H NMR (300 MHz, DMSO-*d*$_6$) δ 7.60 – 7.31 (m, 5H), 6.83 (dd, *J* = 8.8, 2.6 Hz, 2H), 6.73 (d, *J* = 8.7 Hz, 1H), 6.32 (d, *J* = 2.6 Hz, 1H), 4.37 (br s, 2H). **^{13}C NMR (75 MHz, DMSO-*d*$_6$)** δ 196.22 , 145.44 , 140.72 , 137.77 , 130.83 , 129.99 , 129.73 , 128.68 , 127.60 , 126.05 , 118.46 , 116.61 , 116.25 . **Pale brown solid.**

KM10-284

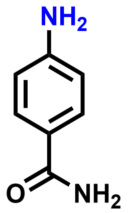

^{1}H NMR (300 MHz, DMSO-d_6) δ 7.63 (m, 3H), 6.95 (br s, 1H), 6.56 (d, *J* = 8.6 Hz, 2H), 5.63 (br s, 2H). **^{13}C NMR (75 MHz, DMSO-d_6)** δ 168.75 , 152.15 , 129.64 , 121.34 , 113.00 . **Off white solid.**

KM10-285

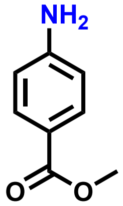

^{1}H NMR (300 MHz, DMSO-d_6) δ 7.67 (d, *J* = 8.7 Hz, 2H), 6.60 (d, *J* = 8.7 Hz, 2H), 5.97 (br s, 2H), 3.74 (s, 3H). **^{13}C NMR (75 MHz, DMSO-d_6)** δ 166.85 , 153.92 , 131.55 , 116.27 , 113.14 , 51.56 . **Off white solid.**

KM10-266

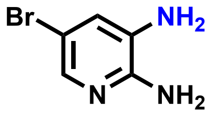

^{1}H NMR (300 MHz, DMSO-d_6) δ 7.29 (d, *J* = 2.2 Hz, 1H), 6.82 (d, *J* = 2.2 Hz, 1H), 5.61 (br s, 2H), 5.01 (br s, 2H). **^{13}C NMR (75 MHz, DMSO-d_6)** δ 147.85 , 134.47 , 132.34 , 119.55 , 106.88 . **Brown solid.**

NMR spectra

170428.f341.10.fid
Kathir KMID-42
PROTON DMSO {C:\Bruker\TopSpin3.5pl6} 1704 41

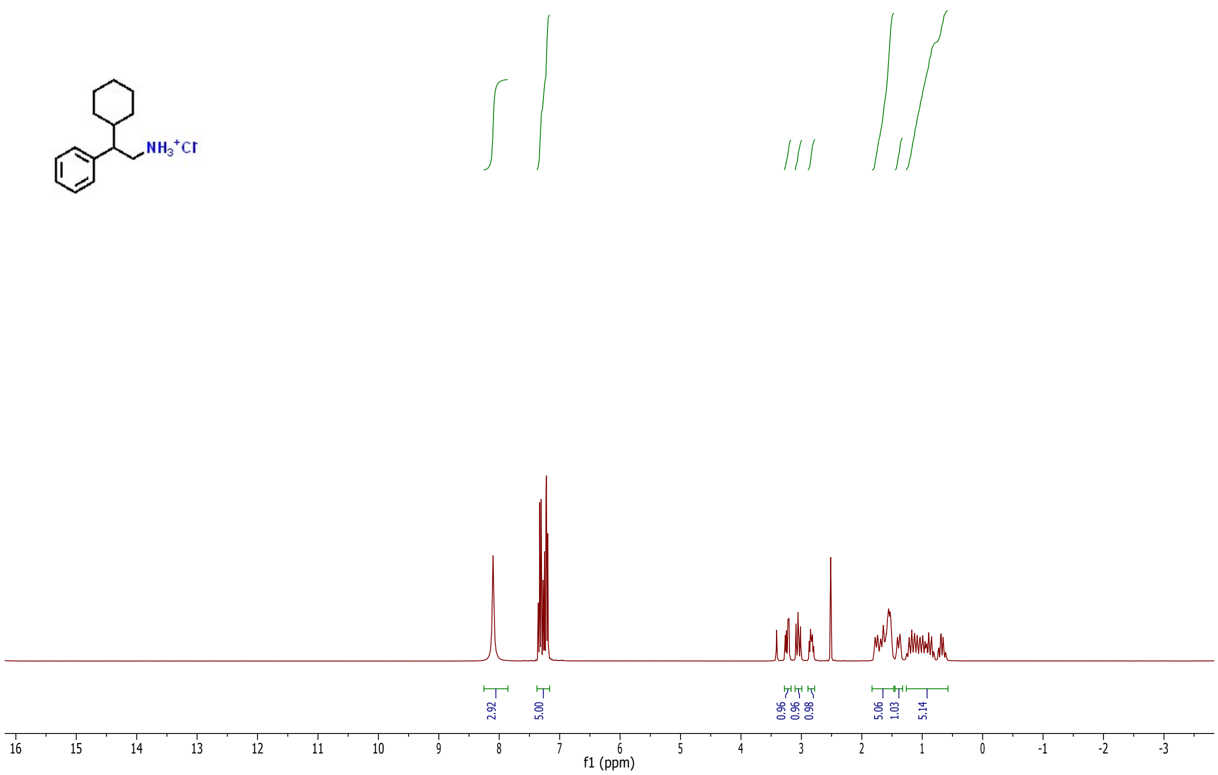

170428.f341.11.fid
Kathir KMID-42
C13CPD DMSO {C:\Bruker\TopSpin3.5pl6} 1704 41

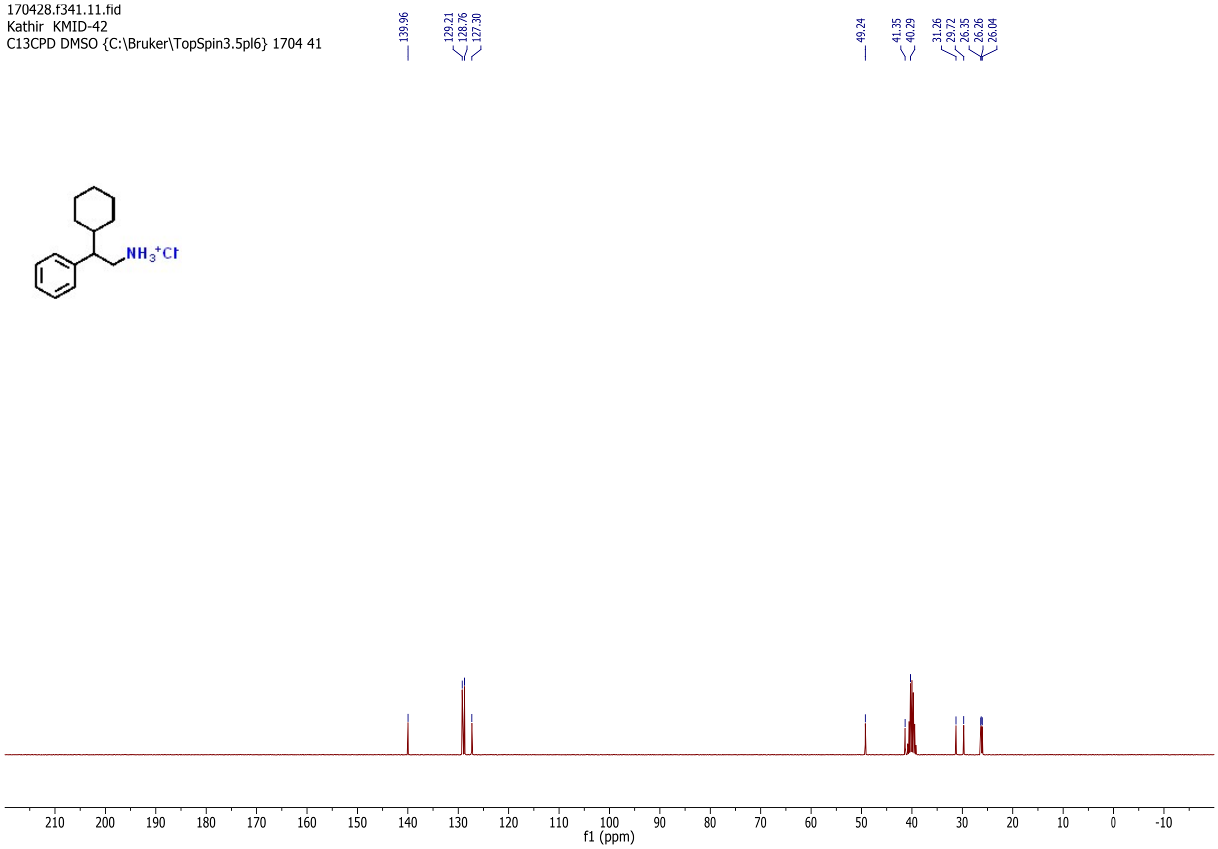

170428.f342.10.fid
Kathir KMID-47
PROTON DMSO {C:\Bruker\TopSpin3.5pl6} 1704 42

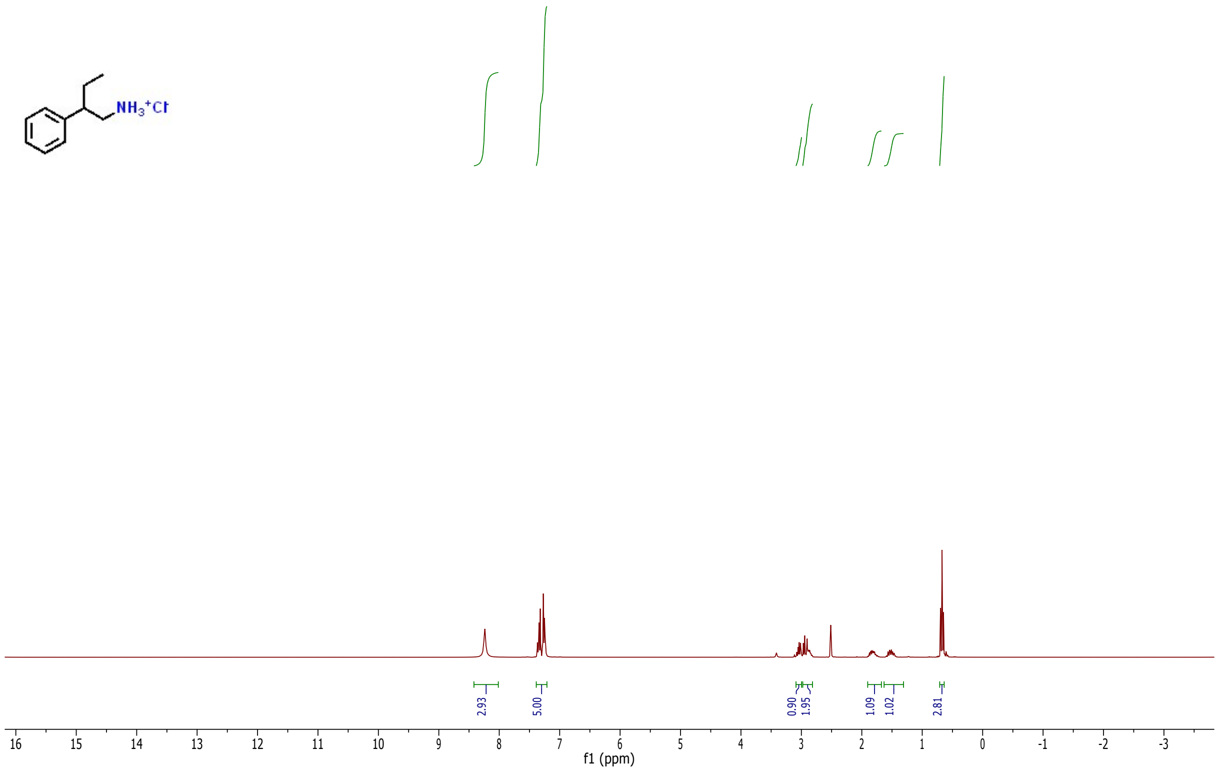

170428.f342.11.fid
Kathir KMID-47
C13CPD DMSO {C:\Bruker\TopSpin3.5pl6} 1704 42

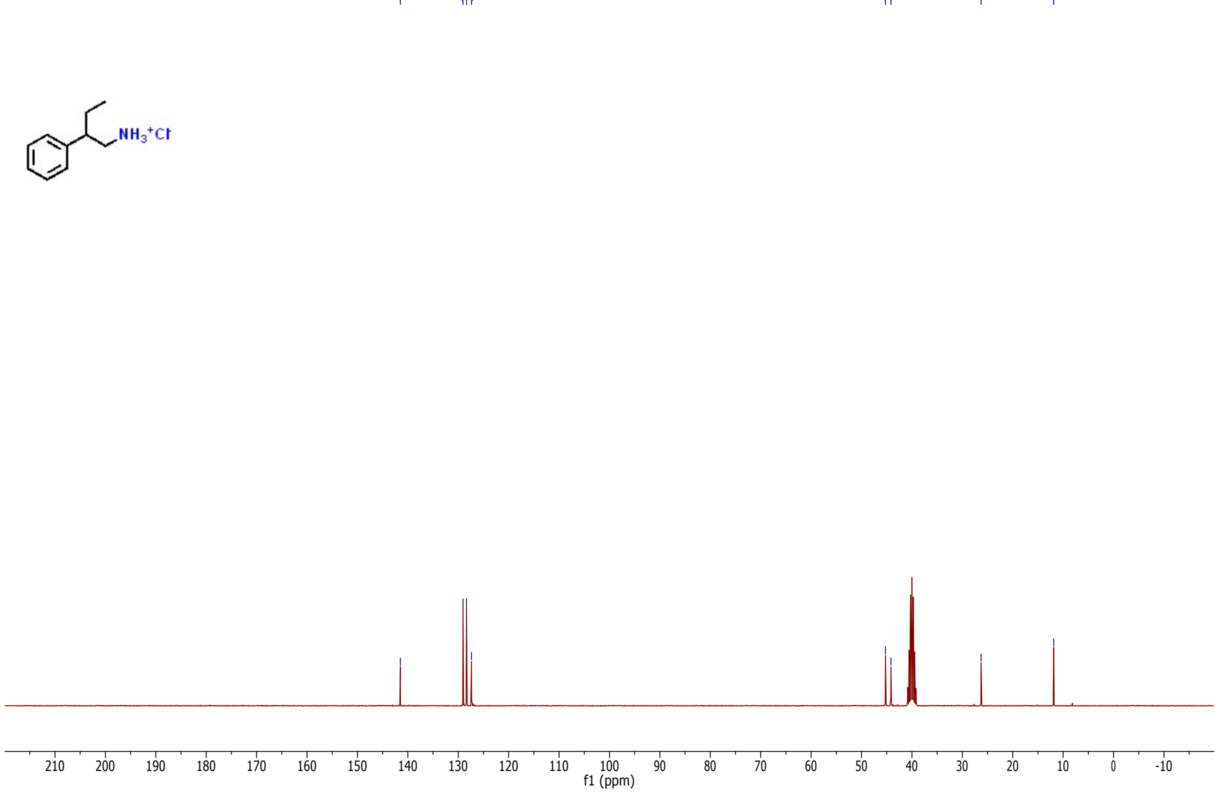

170428.f343.10.fid
Kathir KMID-48
PROTON DMSO {C:\Bruker\TopSpin3.5pl6} 1704 43

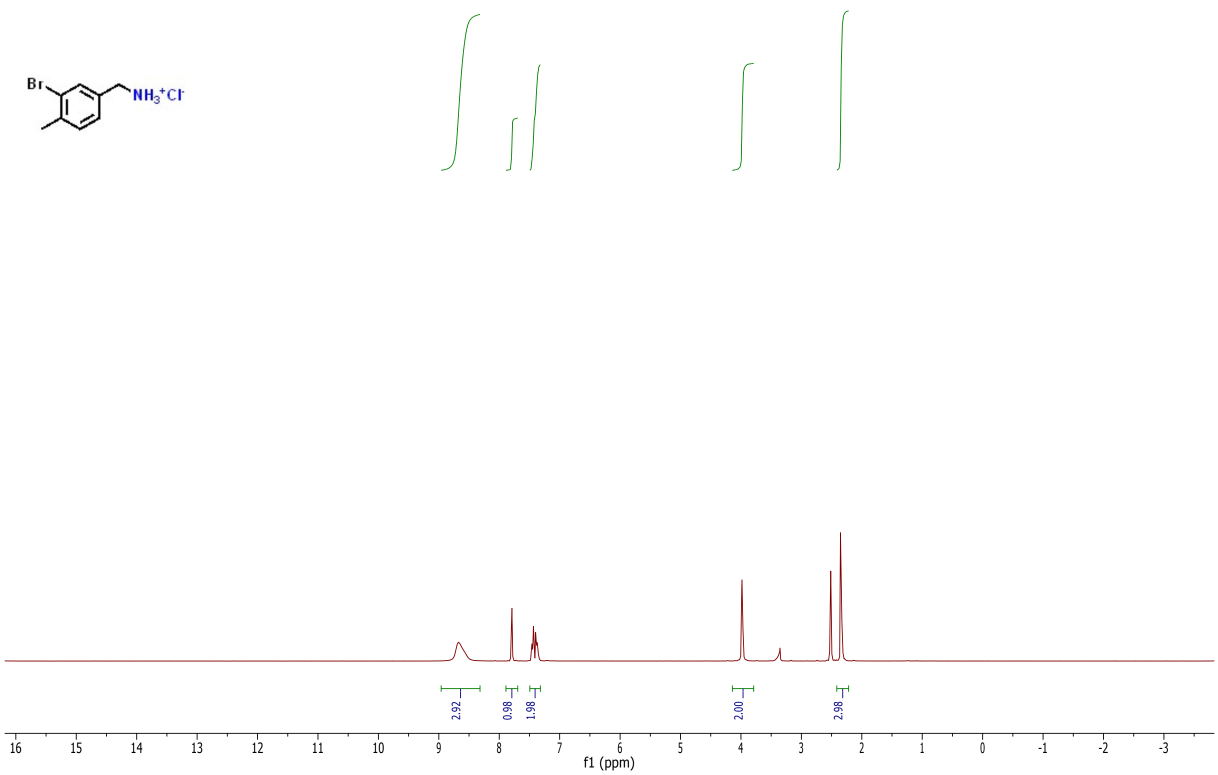

170428.f343.11.fid
Kathir KMID-48
C13CPD DMSO {C:\Bruker\TopSpin3.5pl6} 1704 43

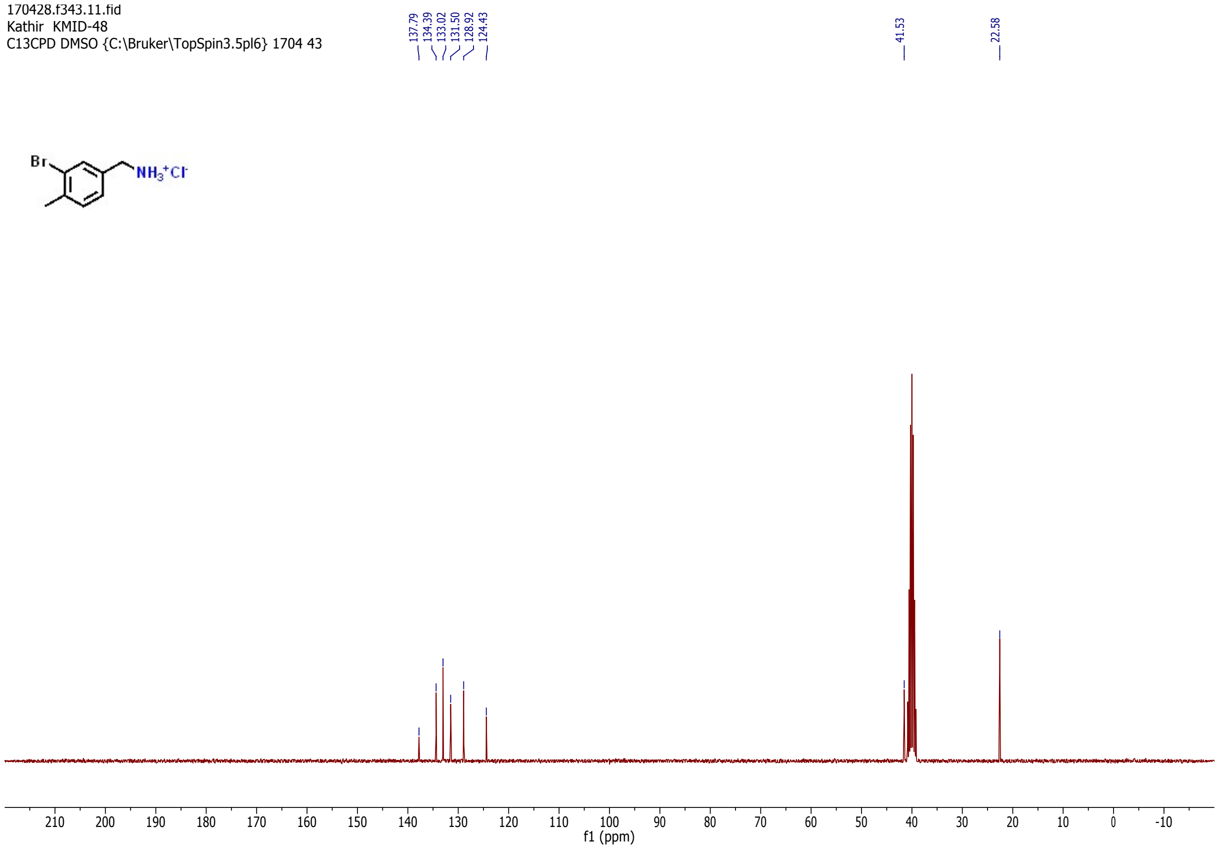

170428.f344.10.fid
Kathir KMID-52
PROTON DMSO {C:\Bruker\TopSpin3.5pl6} 1704 44

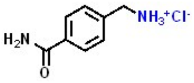

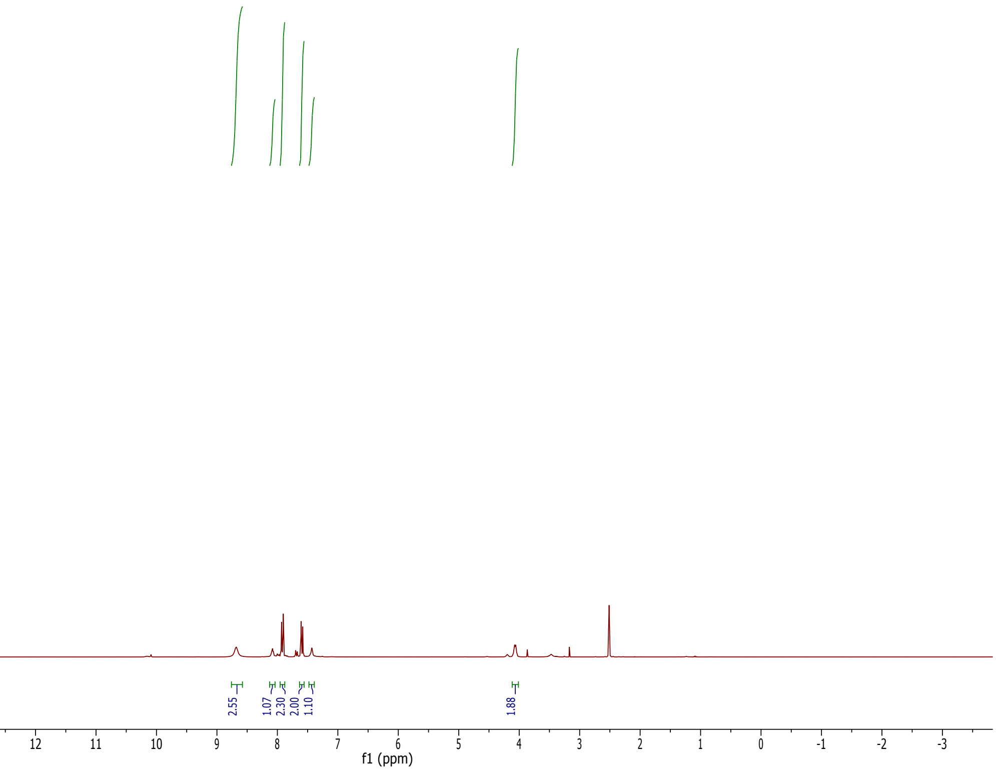

170428.f344.11.fid
Kathir KMID-52
C13CPD DMSO {C:\Bruker\TopSpin3.5pl6} 1704 44

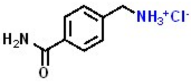

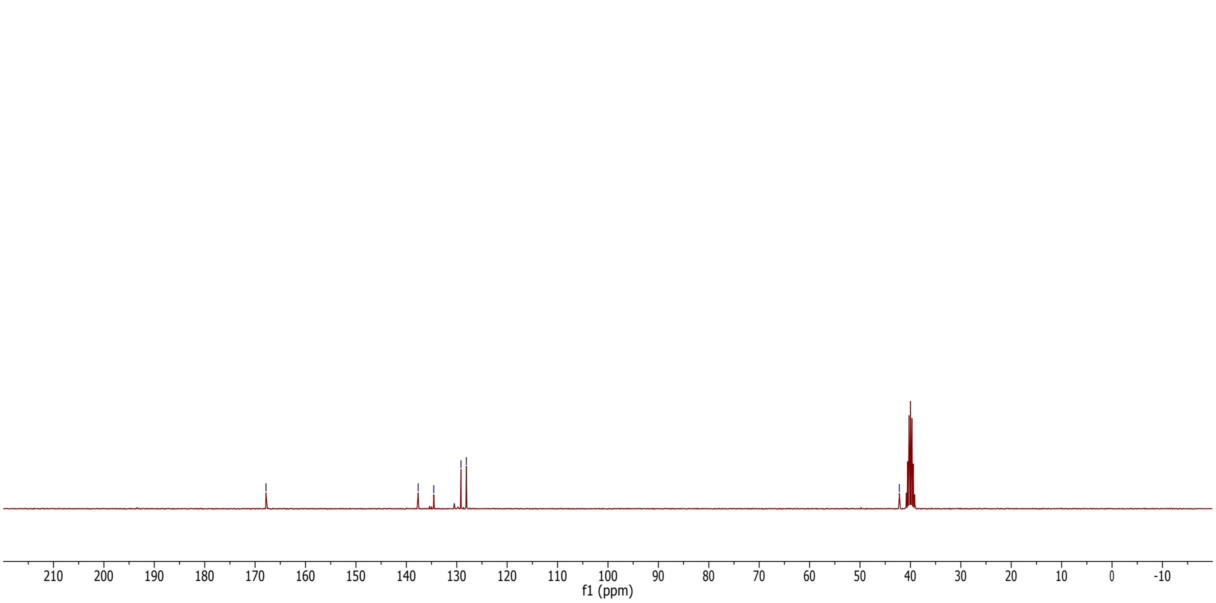

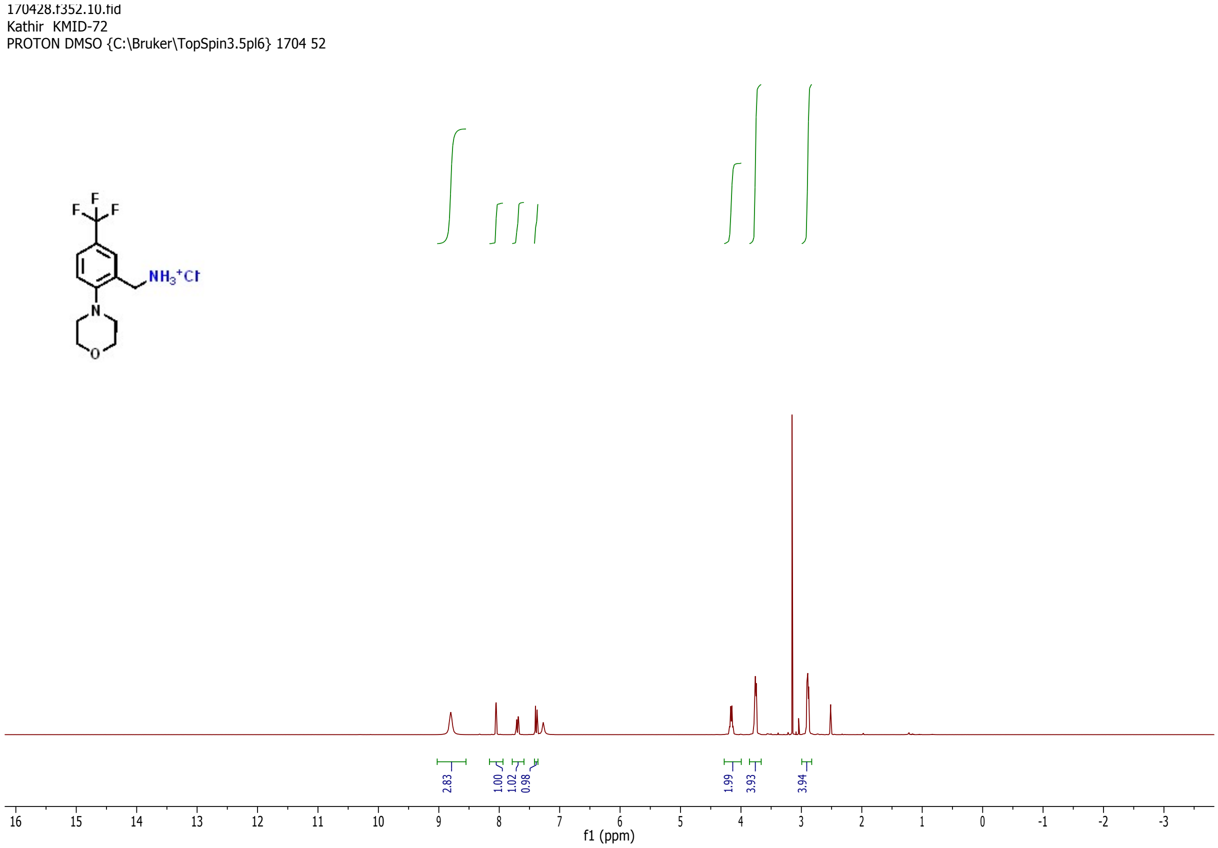
170428.f352.10.fid
Kathir KMID-72
PROTON DMSO {C:\Bruker\TopSpin3.5pl6} 1704 52
F
F
F
NH3+Cl-
N
O
2.83
1.00
1.02
0.98
1.99
3.93
3.94
16 15 14 13 12 11 10 9 8 7 6 5 4 3 2 1 0 -1 -2 -3
f1 (ppm)

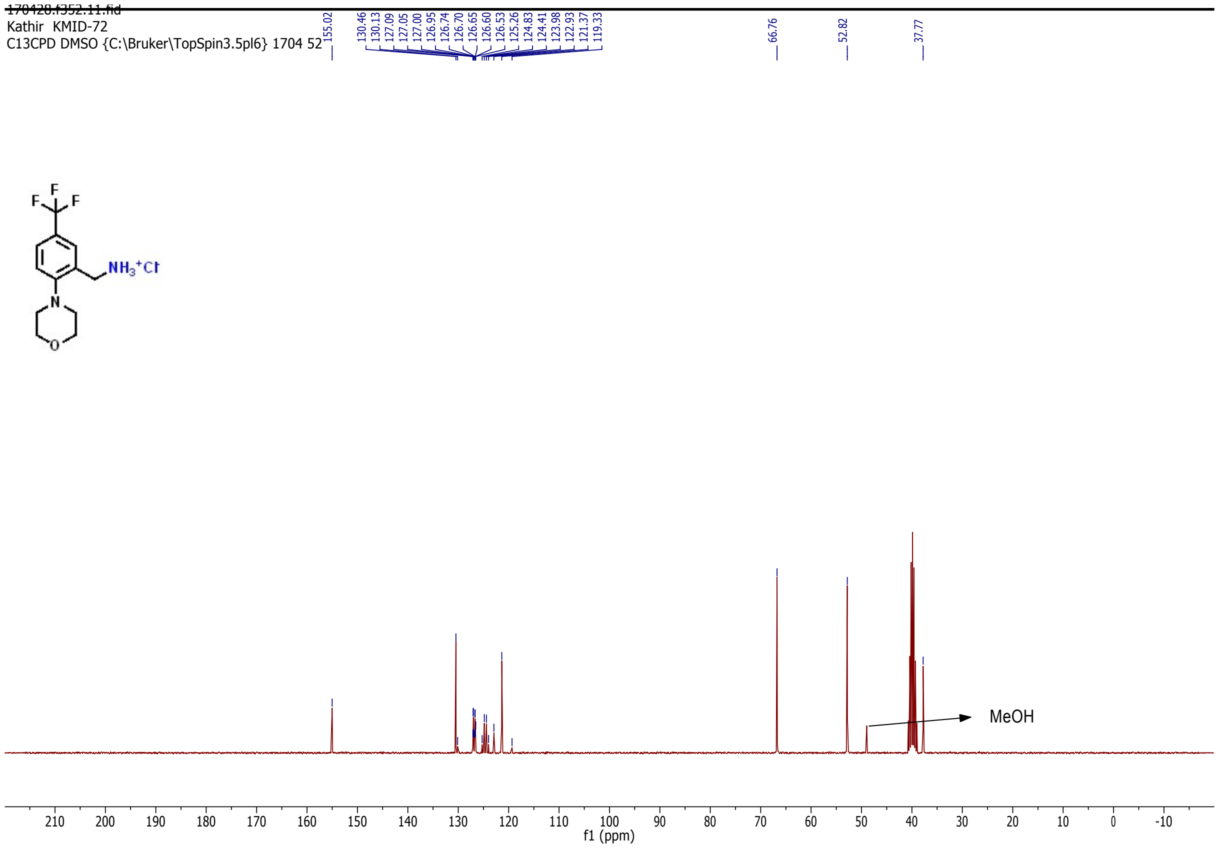
170428.f352.11.fid
Kathir KMID-72
C13CPD DMSO {C:\Bruker\TopSpin3.5pl6} 1704 52
155.02
130.46
130.13
127.09
127.05
127.00
126.95
126.74
126.70
126.65
126.60
126.53
125.26
124.83
124.41
123.98
122.93
121.37
119.33
66.76
52.82
37.77
F
F
F
NH3+Cl-
N
O
MeOH
210 200 190 180 170 160 150 140 130 120 110 100 90 80 70 60 50 40 30 20 10 0 -10
f1 (ppm)

170428.f353.10.fid
Kathir KMID-73
PROTON DMSO {C:\Bruker\TopSpin3.5pl6} 1704 53

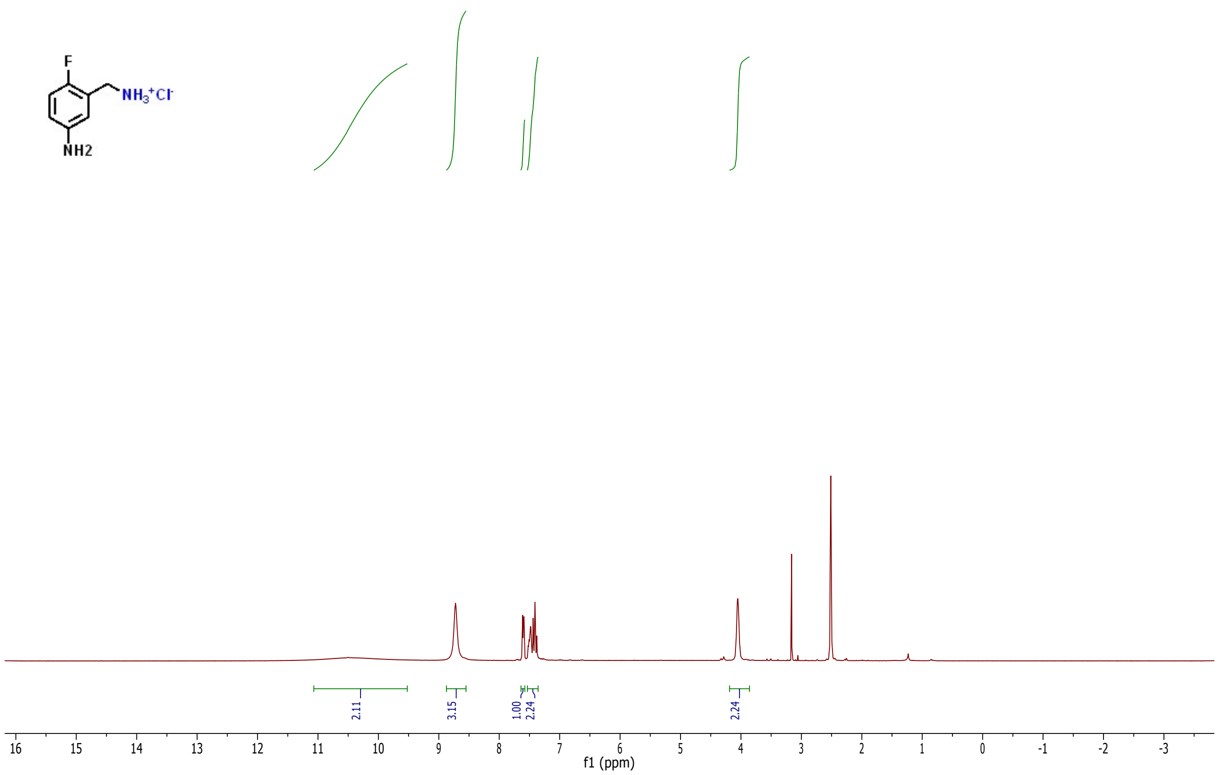

170428.f353.11.fid
Kathir KMID-73
C13CPD DMSO {C:\Bruker\TopSpin3.5pl6} 1704 53

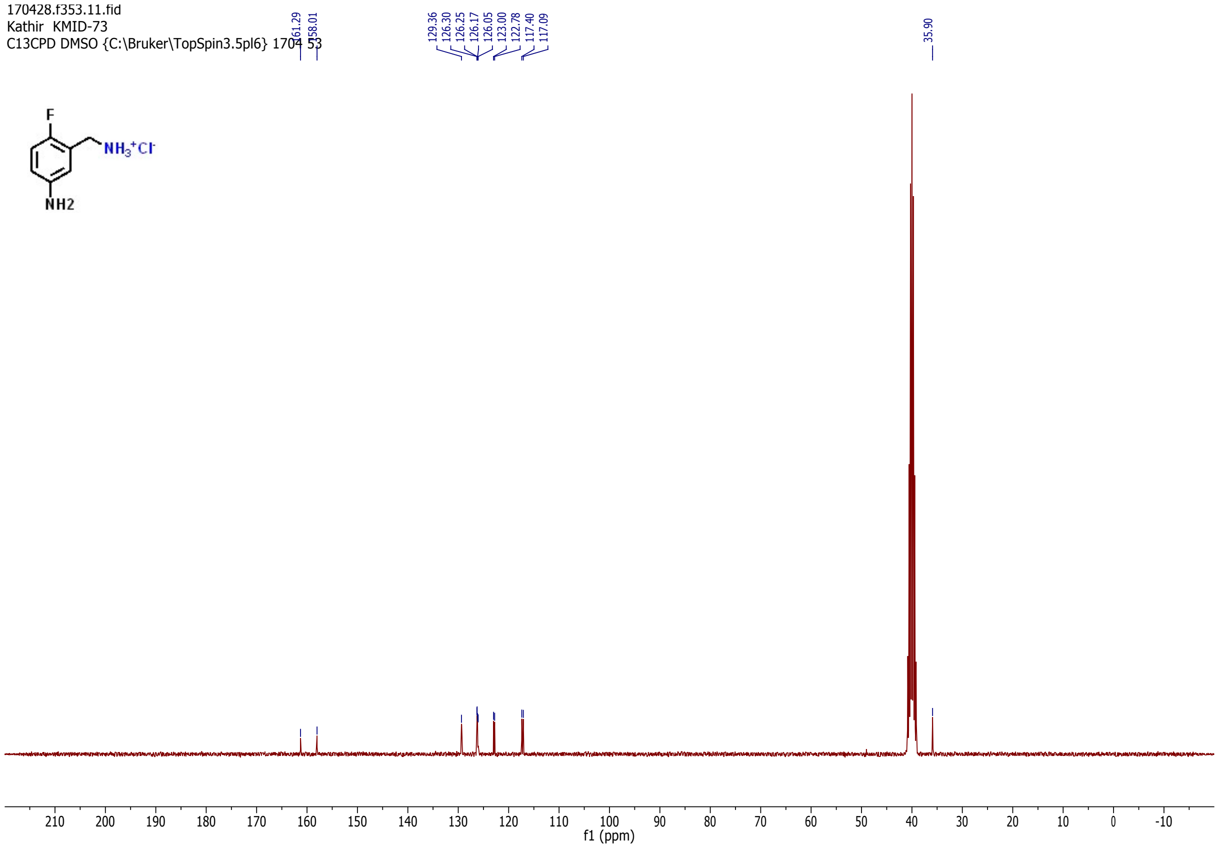

170428.f354.10.fid
Kathir KMID-74
PROTON DMSO {C:\Bruker\TopSpin3.5pl6} 1704 54

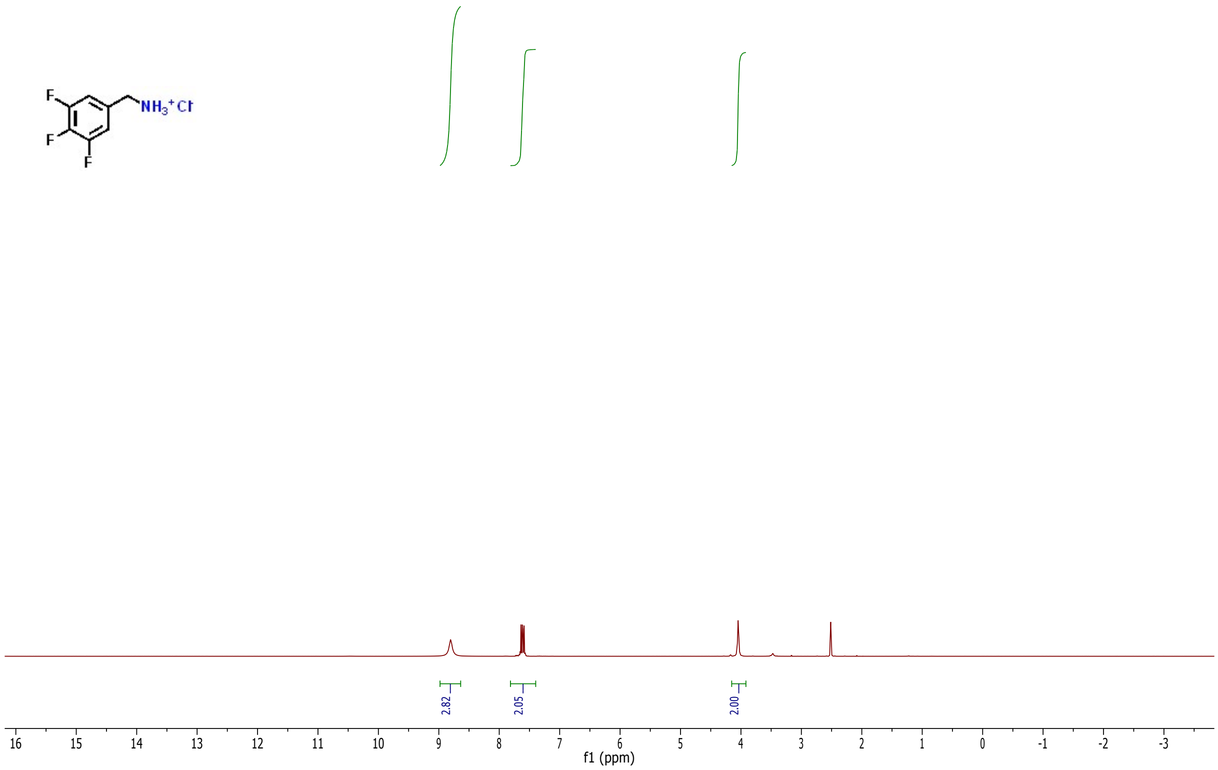

170428.f354.11.fid
Kathir KMID-74
C13CPD DMSO {C:\Bruker\TopSpin3.5pl6} 1704 54

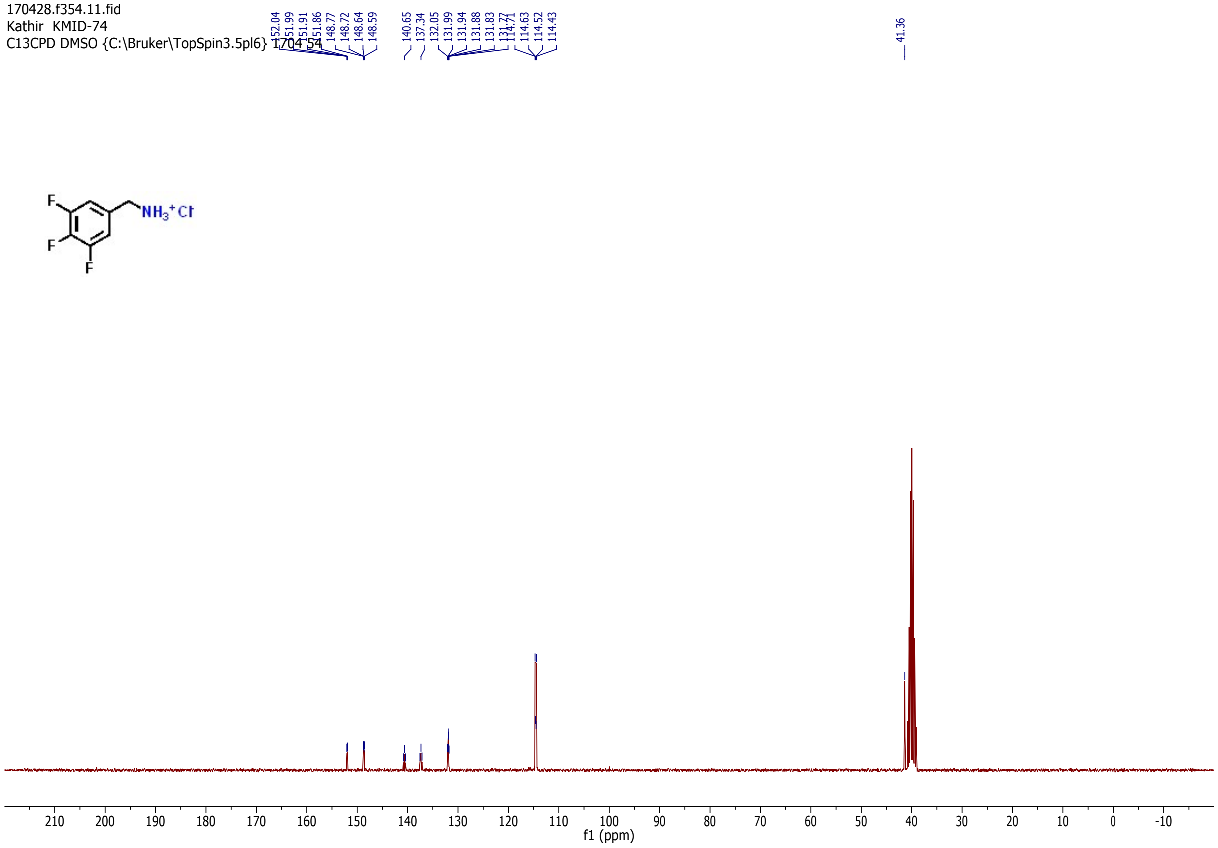

170428.f355.10.fid
Kathir KMID-77
PROTON DMSO {C:\Bruker\TopSpin3.5pl6} 1704 55

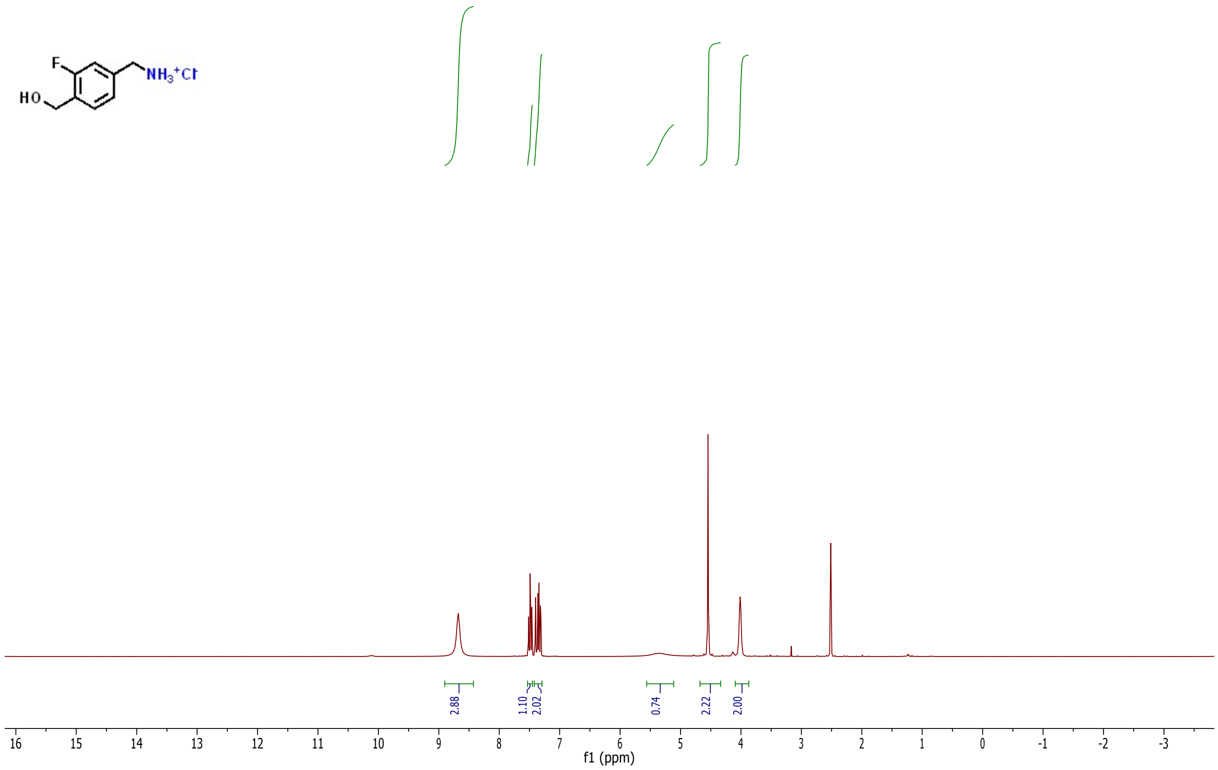

170428.f355.11.fid
Kathir KMID-77
C13CPD DMSO {C:\Bruker\TopSpin3.5pl6} 1704 55

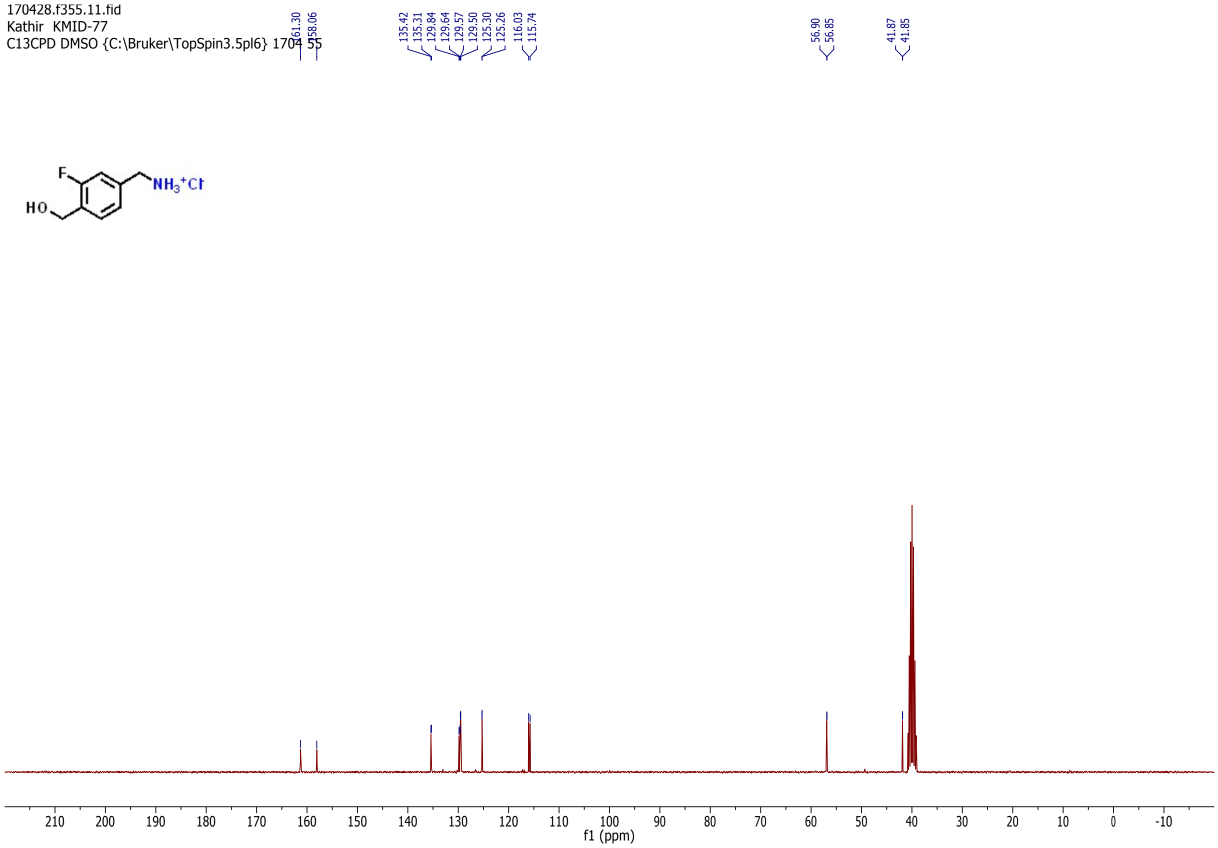

170503.f301.10.fid
Kathir/ KM10-24
PROTON DMSO {C:\Bruker\TopSpin3.5pl6} 1705 1

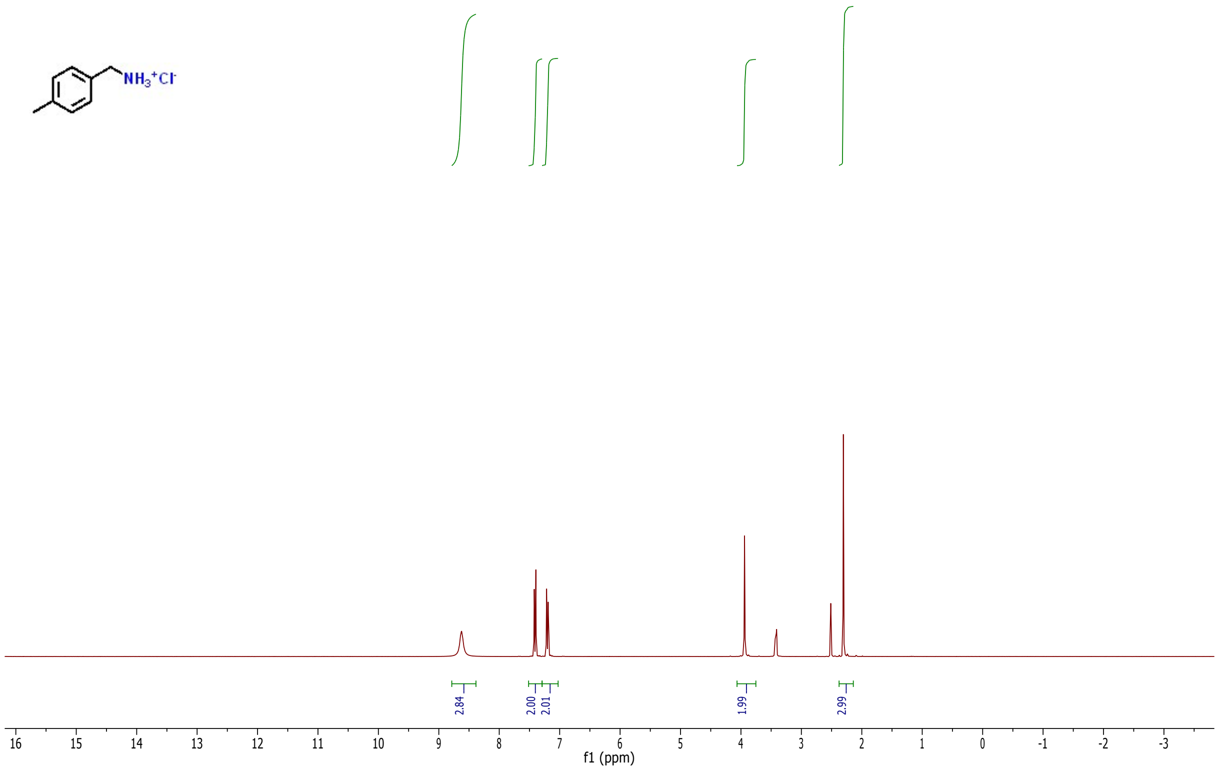

170503.f301.11.fid
Kathir/ KM10-24
C13CPD DMSO {C:\Bruker\TopSpin3.5pl6} 1705 1

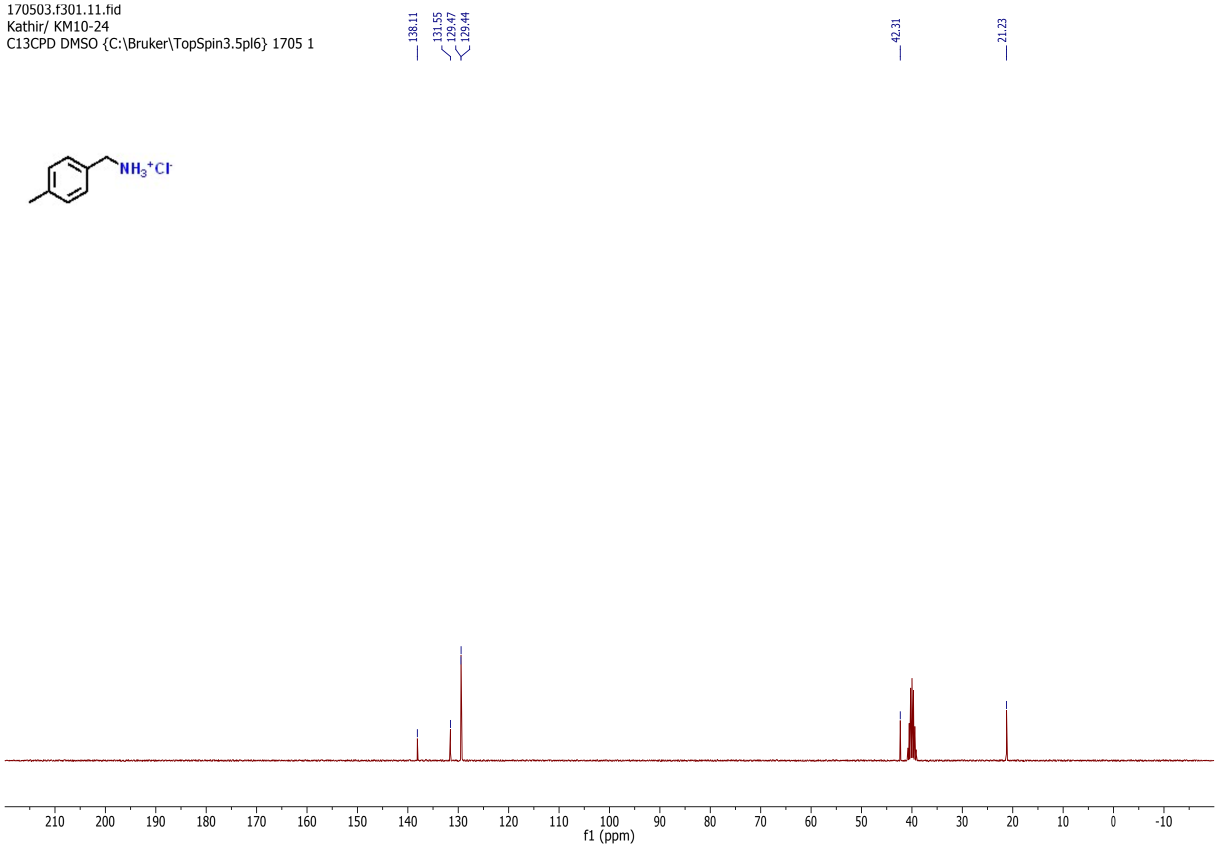

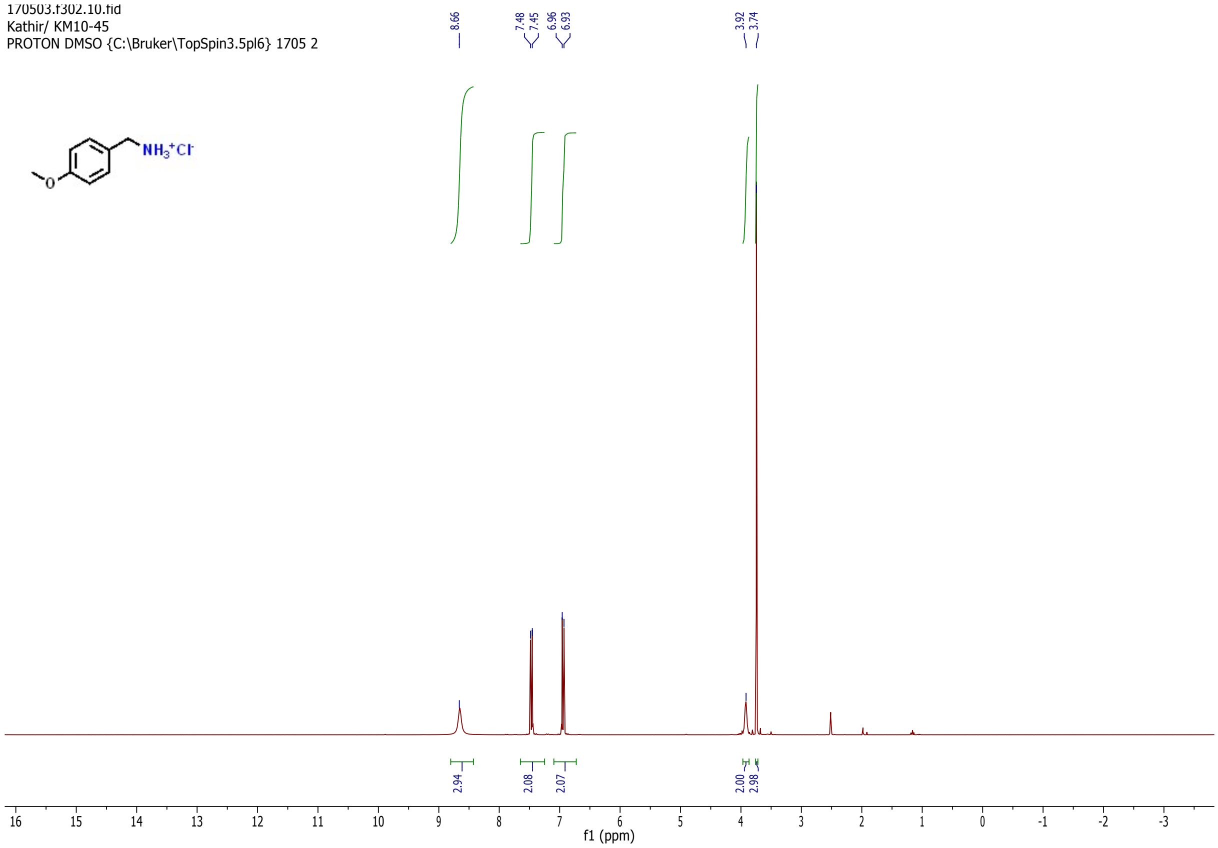
170503.f302.10.fid
Kathir/ KM10-45
PROTON DMSO {C:\Bruker\TopSpin3.5pl6} 1705 2
8.66
7.48
7.45
6.96
6.93
3.92
3.74
NH3+Cl-
2.94
2.08
2.07
2.00
2.98
f1 (ppm)

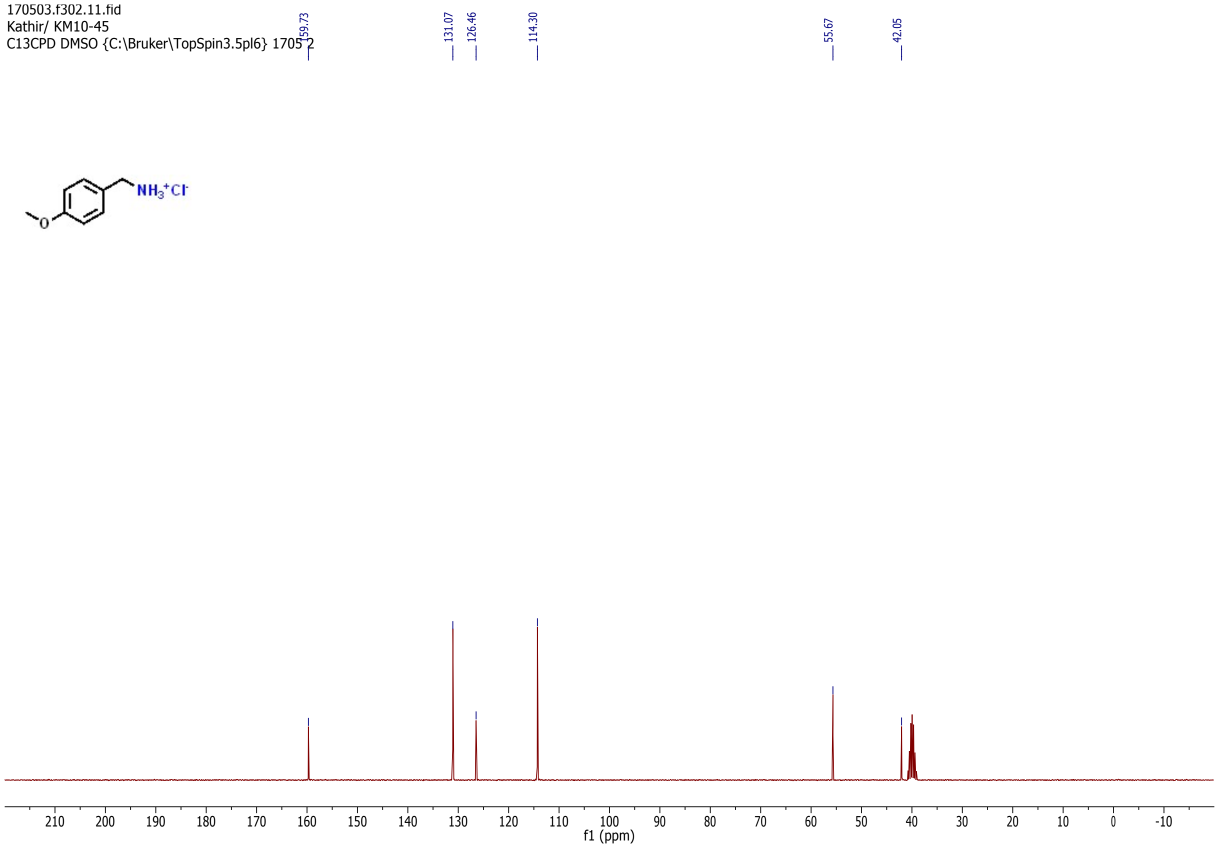
170503.f302.11.fid
Kathir/ KM10-45
C13CPD DMSO {C:\Bruker\TopSpin3.5pl6} 1705 2
159.73
131.07
126.46
114.30
55.67
42.05
NH3+Cl-
f1 (ppm)

170503.f303.10.fid
Kathir/ KM10-56
PROTON DMSO {C:\Bruker\TopSpin3.5pl6} 1705 3

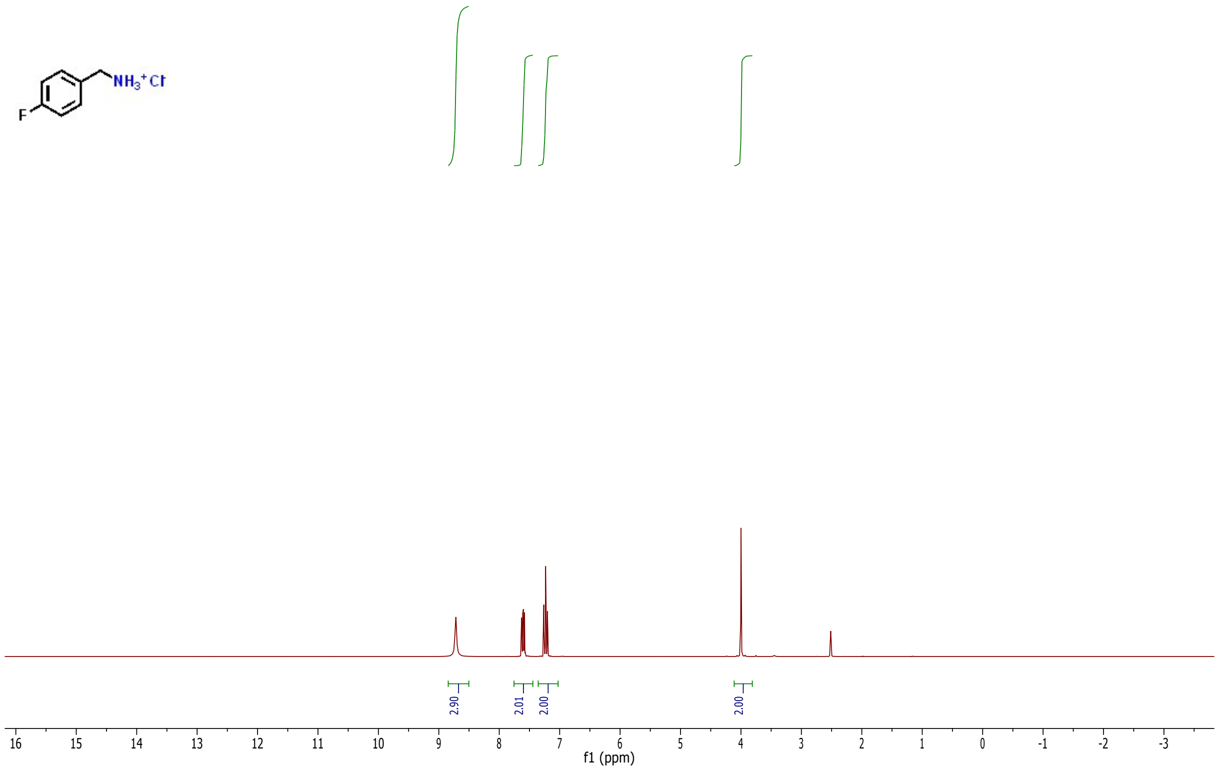

170503.f303.11.fid
Kathir/ KM10-56
C13CPD DMSO {C:\Bruker\TopSpin3.5pl6} 1705 3

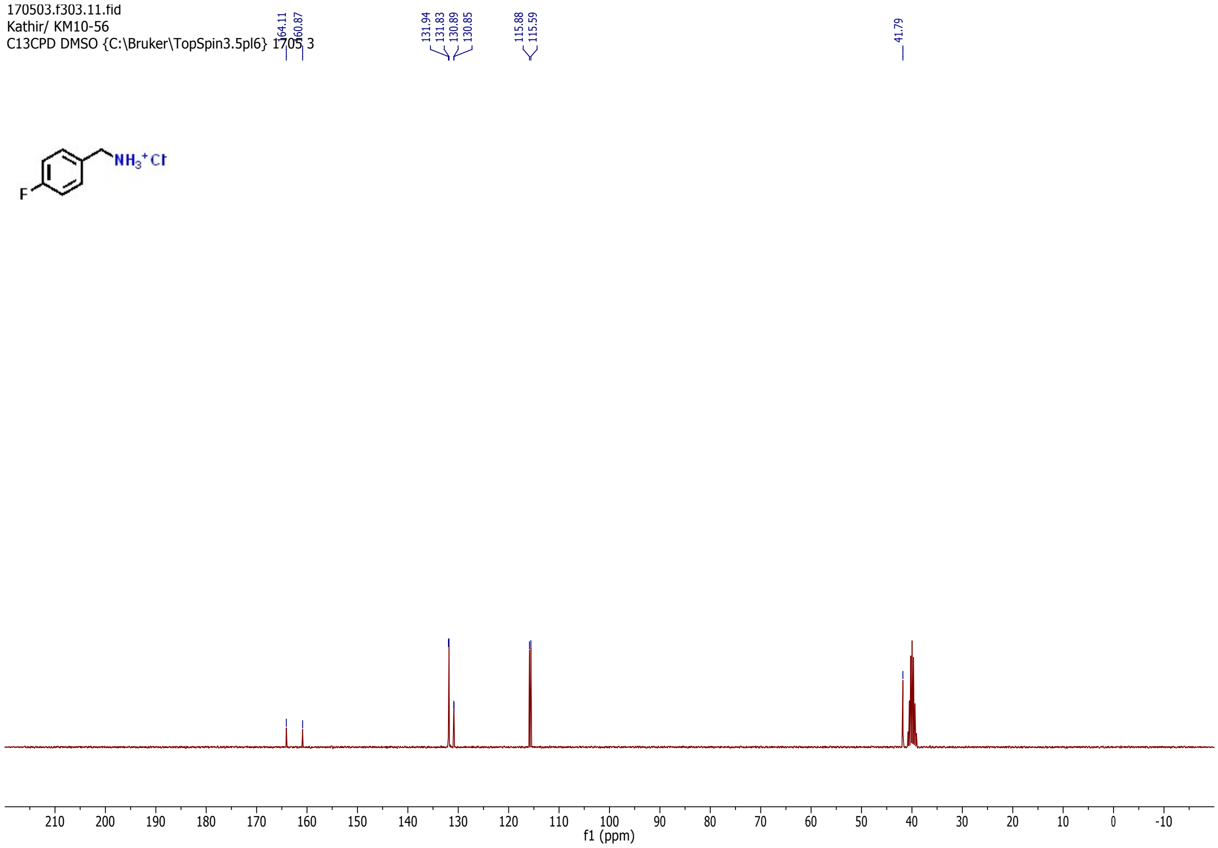

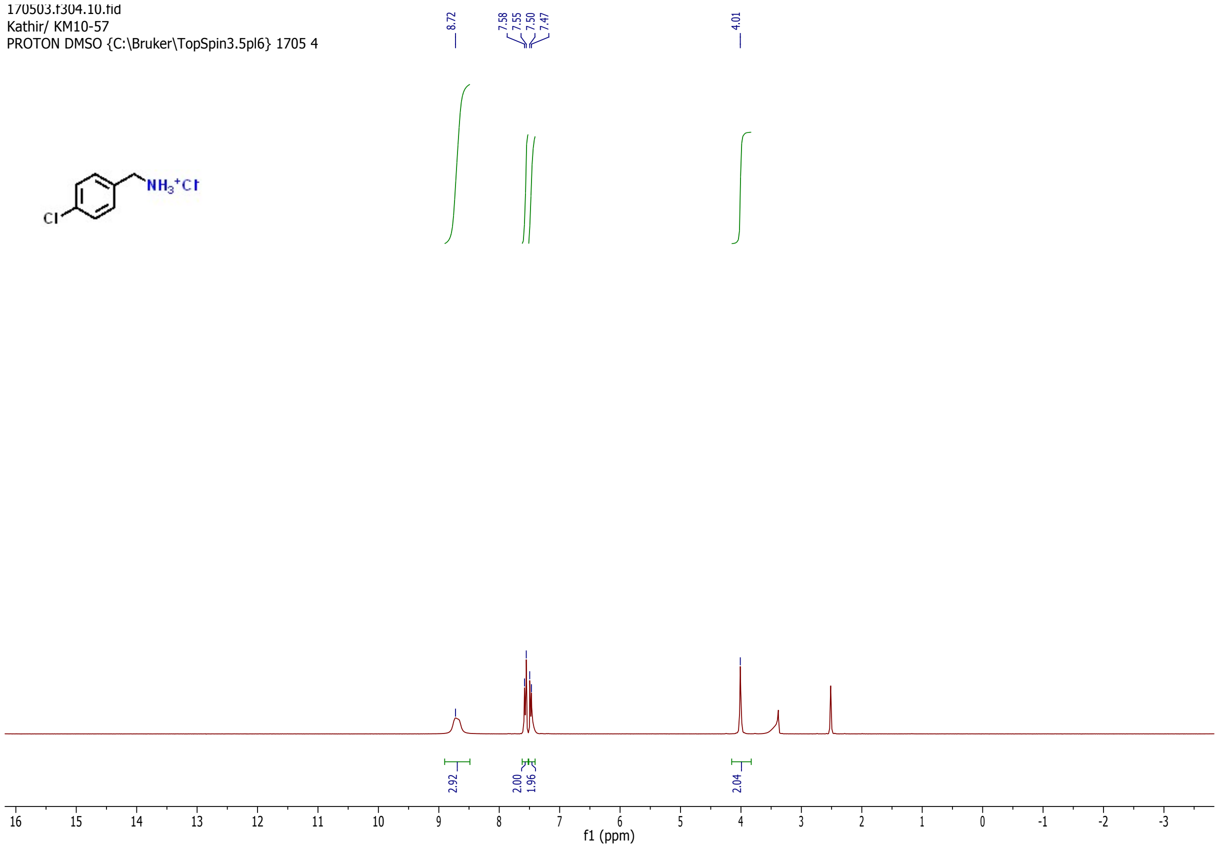
170503.f304.10.fid
Kathir/ KM10-57
PROTON DMSO {C:\Bruker\TopSpin3.5pl6} 1705 4
8.72
7.58
7.55
7.50
7.47
4.01
NH3+Cl-
Cl
2.92
2.00
1.96
2.04
16 15 14 13 12 11 10 9 8 7 6 5 4 3 2 1 0 -1 -2 -3
f1 (ppm)

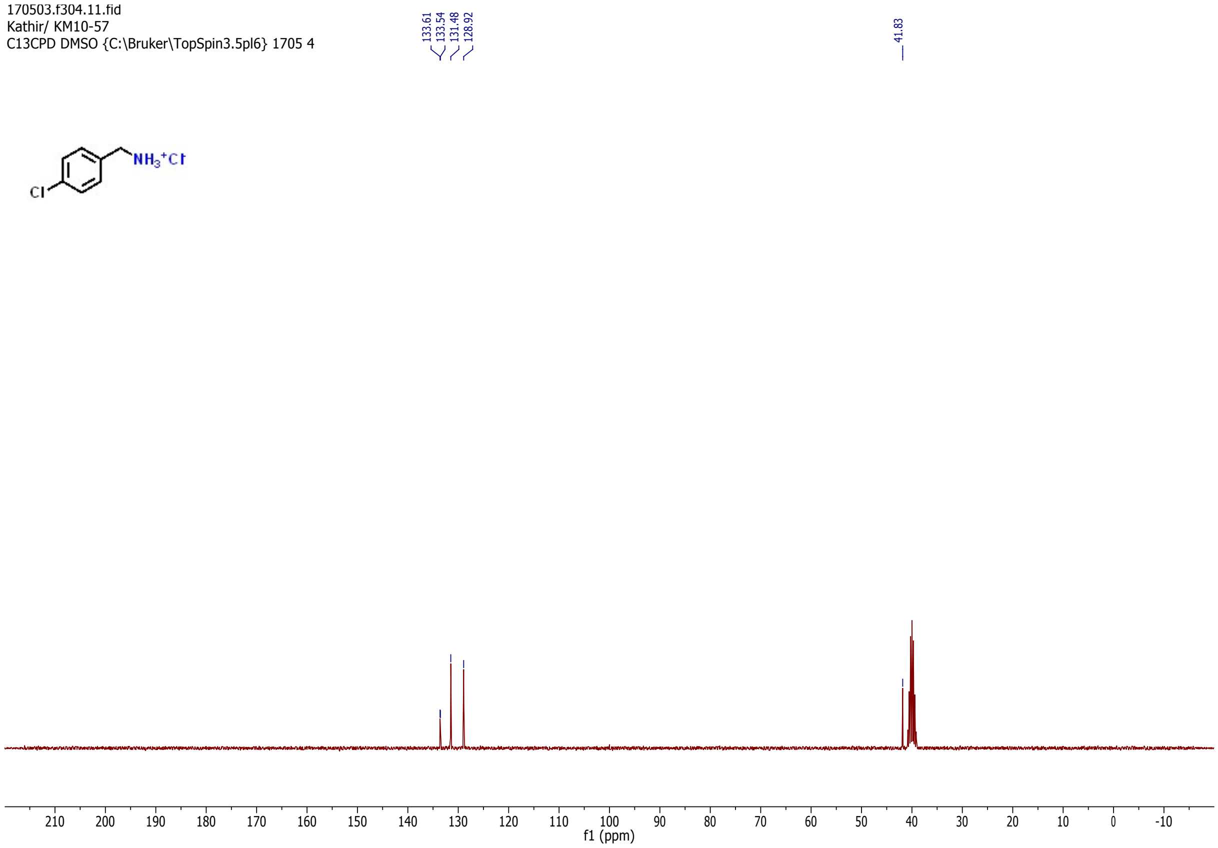
170503.f304.11.fid
Kathir/ KM10-57
C13CPD DMSO {C:\Bruker\TopSpin3.5pl6} 1705 4
133.61
133.54
131.48
128.92
41.83
NH3+Cl-
Cl
210 200 190 180 170 160 150 140 130 120 110 100 90 80 70 60 50 40 30 20 10 0 -10
f1 (ppm)

170503.f305.10.fid
Kathir/ KM10-58
PROTON DMSO {C:\Bruker\TopSpin3.5pl6} 1705 5

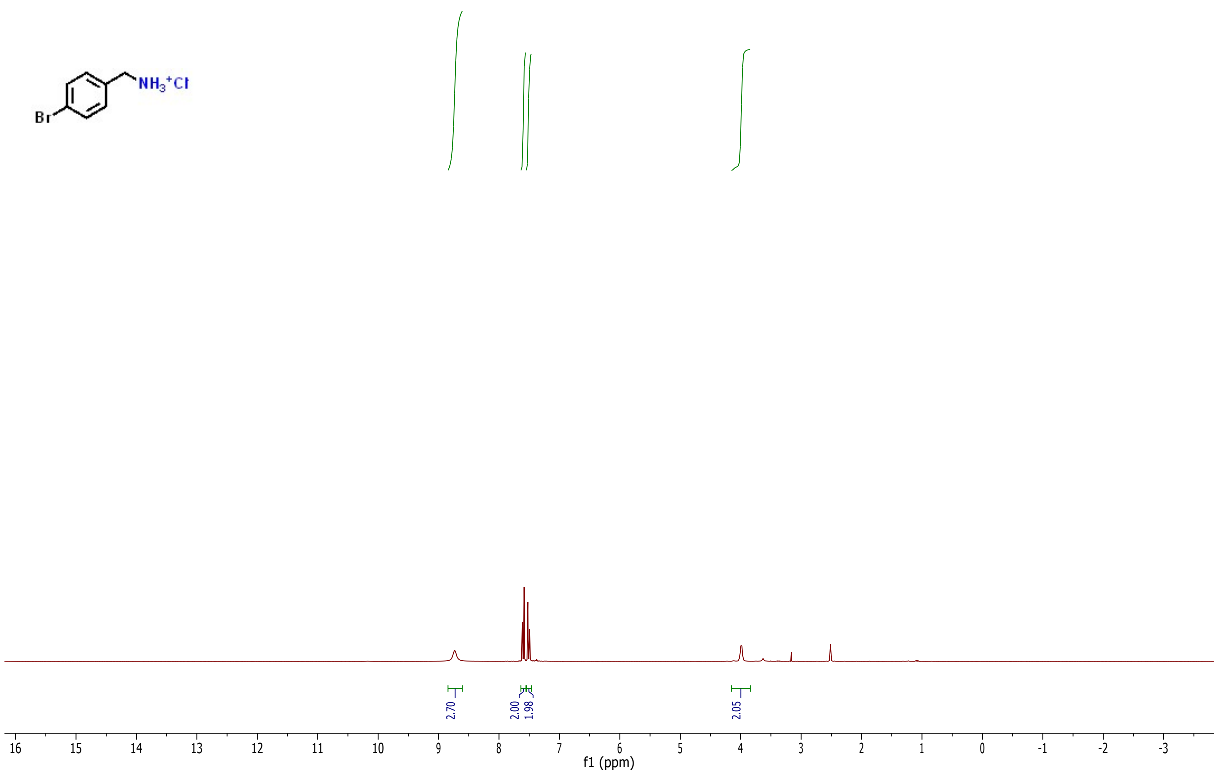

170503.f305.11.fid
Kathir/ KM10-58
C13CPD DMSO {C:\Bruker\TopSpin3.5pl6} 1705 5

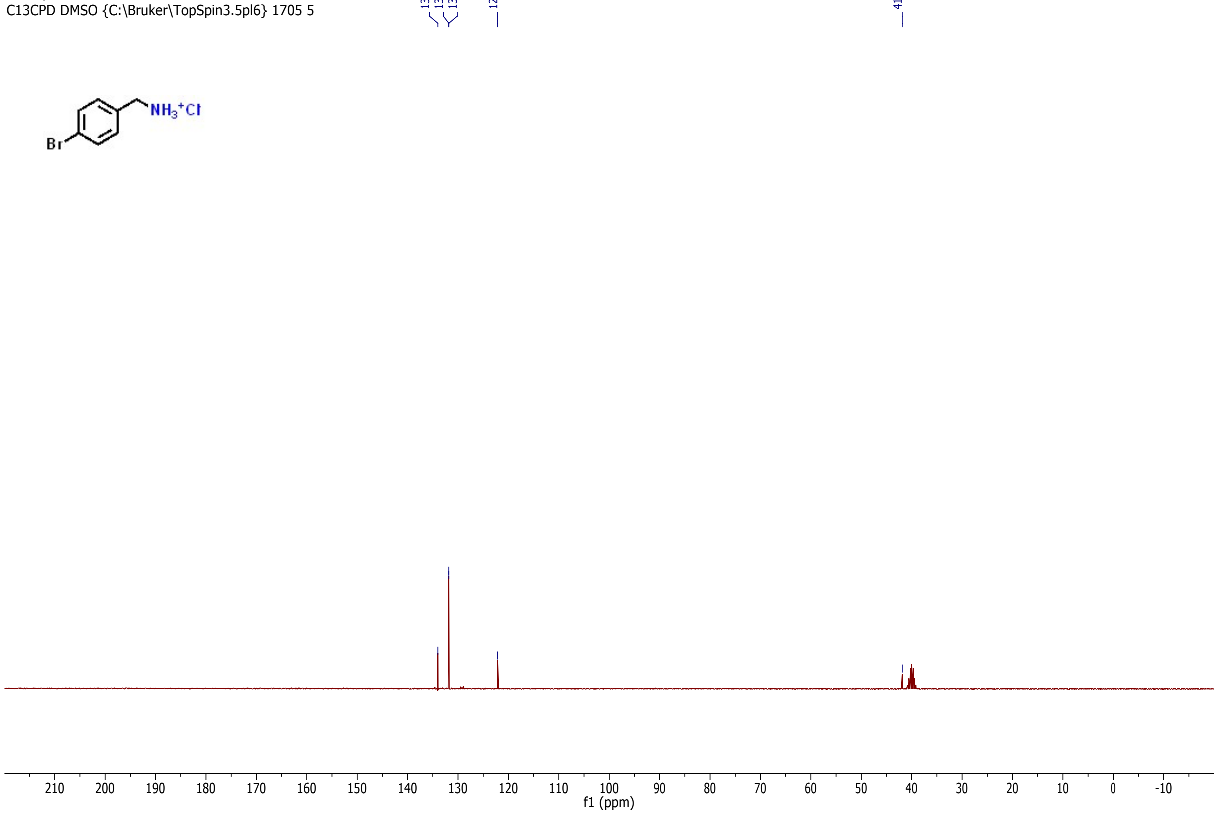

170503.f306.10.fid
Kathir/ KM10-60
PROTON DMSO {C:\Bruker\TopSpin3.5pl6} 1705 6

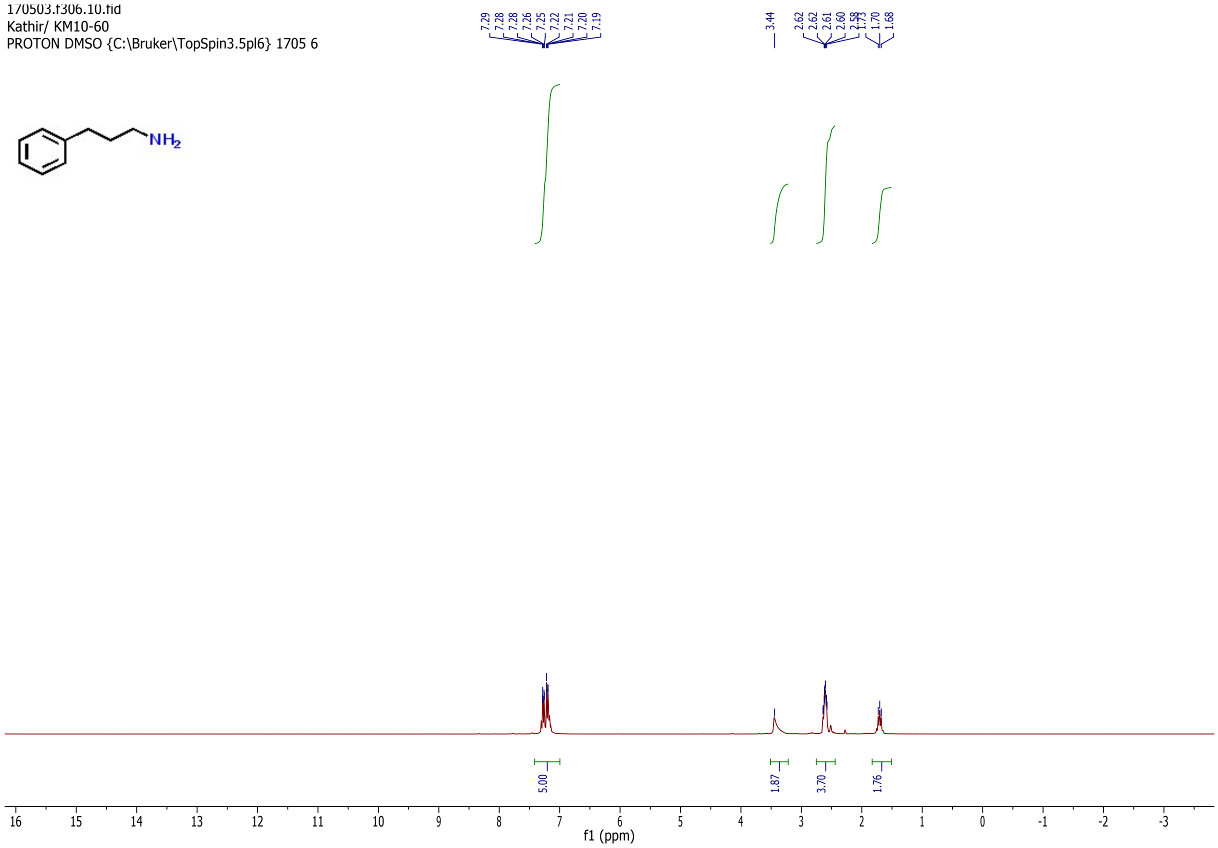

170503.f306.11.fid
Kathir/ KM10-60
C13CPD DMSO {C:\Bruker\TopSpin3.5pl6} 1705 6

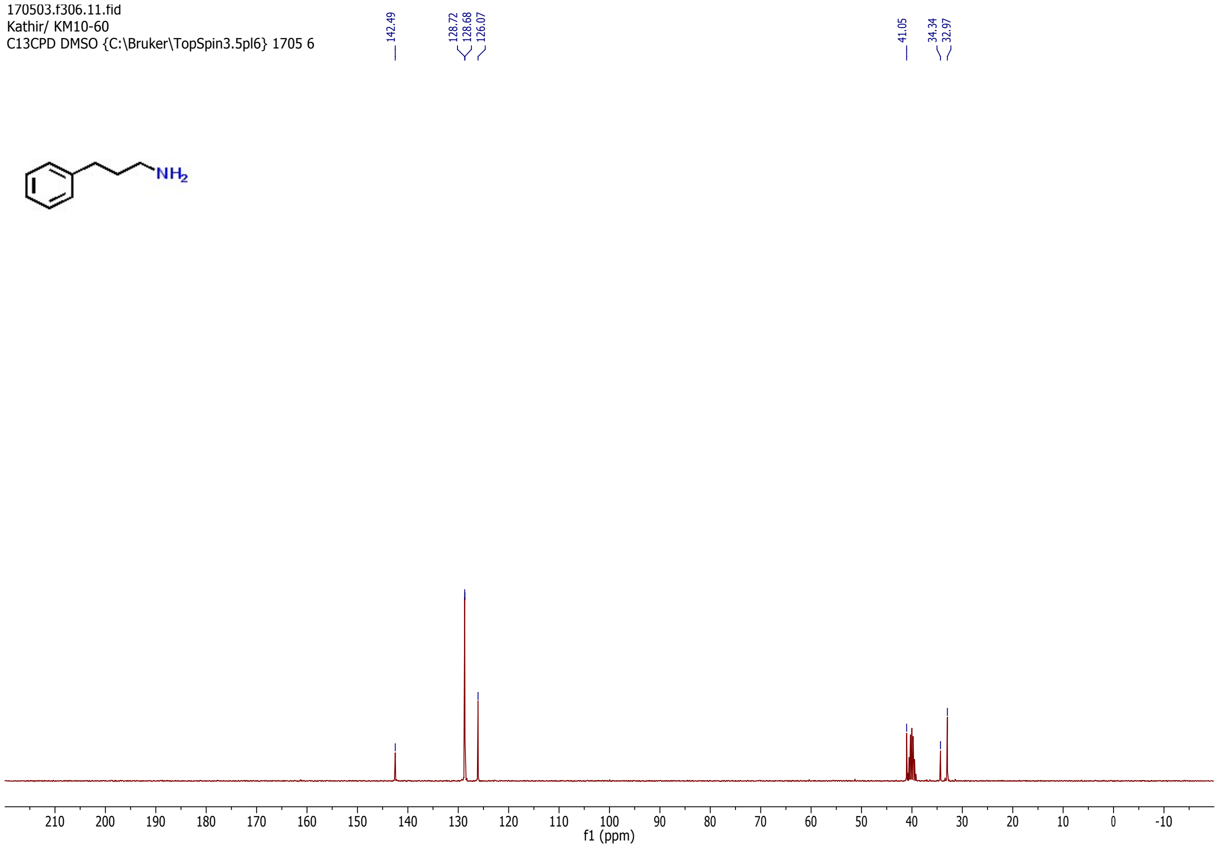

170503.f307.10.fid
Kathir/ KM10-61
PROTON DMSO {C:\Bruker\TopSpin3.5pl6} 1705 7

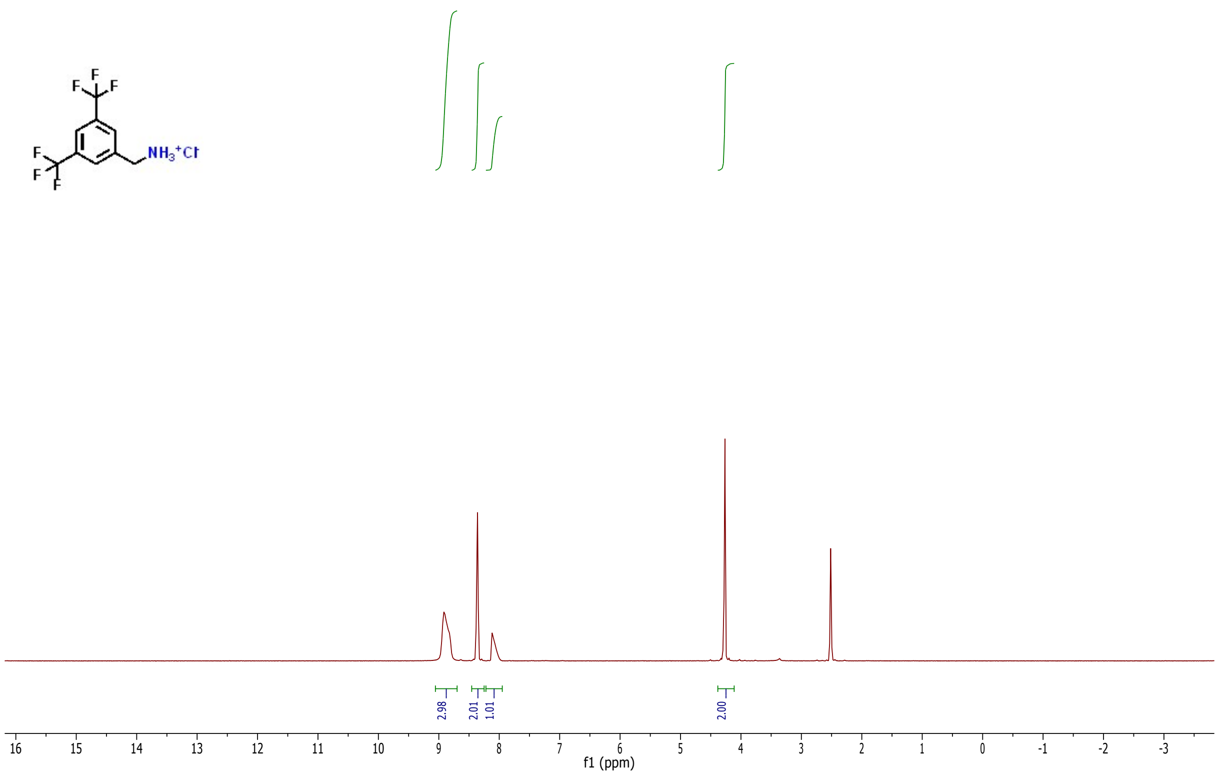

170503.f307.11.fid
Kathir/ KM10-61
C13CPD DMSO {C:\Bruker\TopSpin3.5pl6} 1705 7

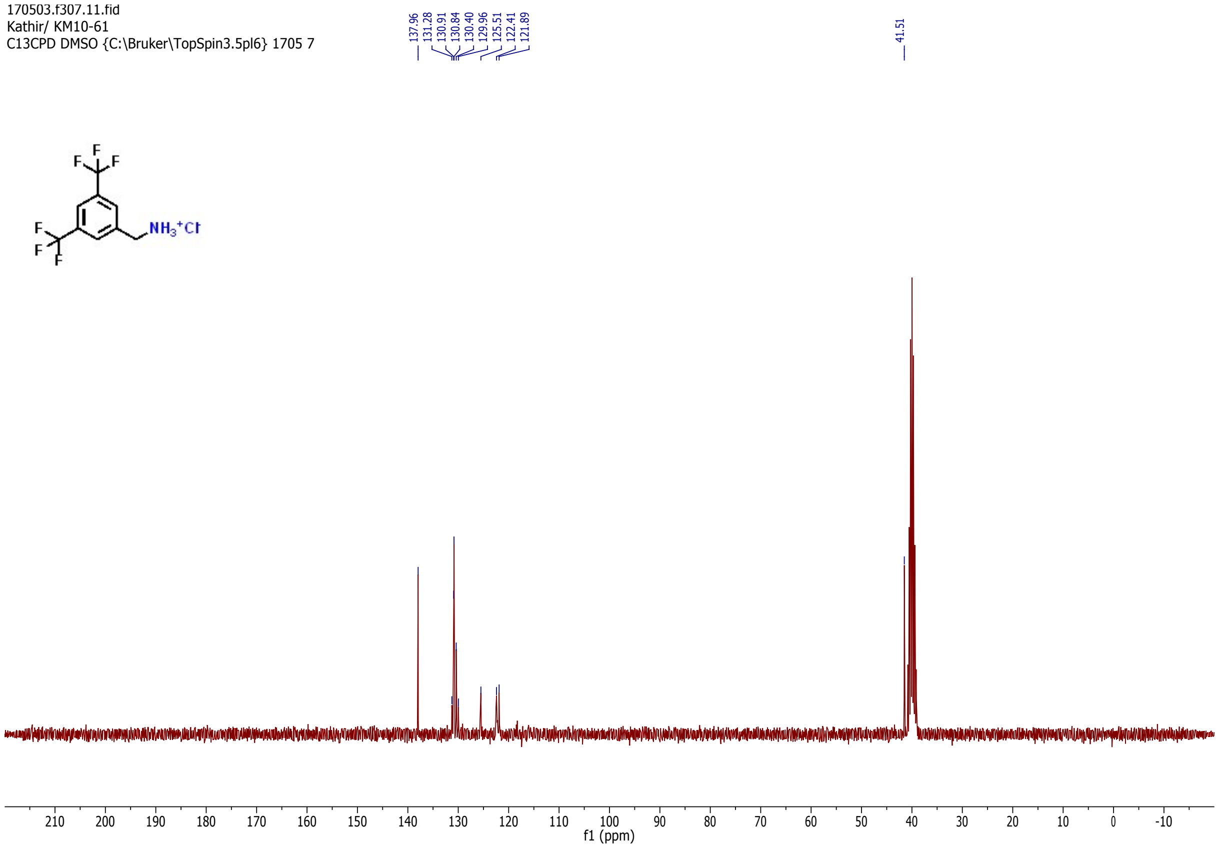

170928.319.3.fid
Kathir KM10-61
Au19F DMSO {C:\Bruker\TopSpin3.5pl6} 1709 19

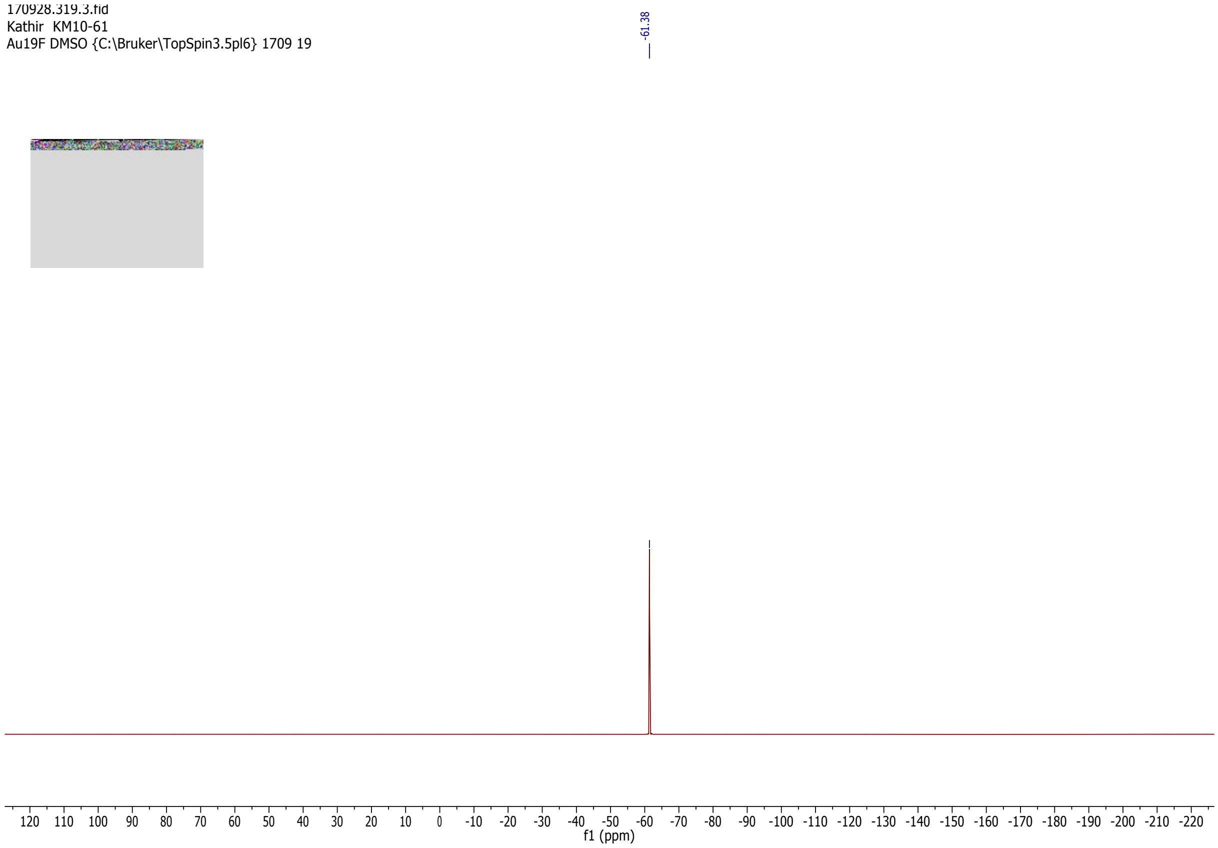

170503.f308.10.fid
Kathir/ KM10-63
PROTON DMSO {C:\Bruker\TopSpin3.5pl6} 1705 8

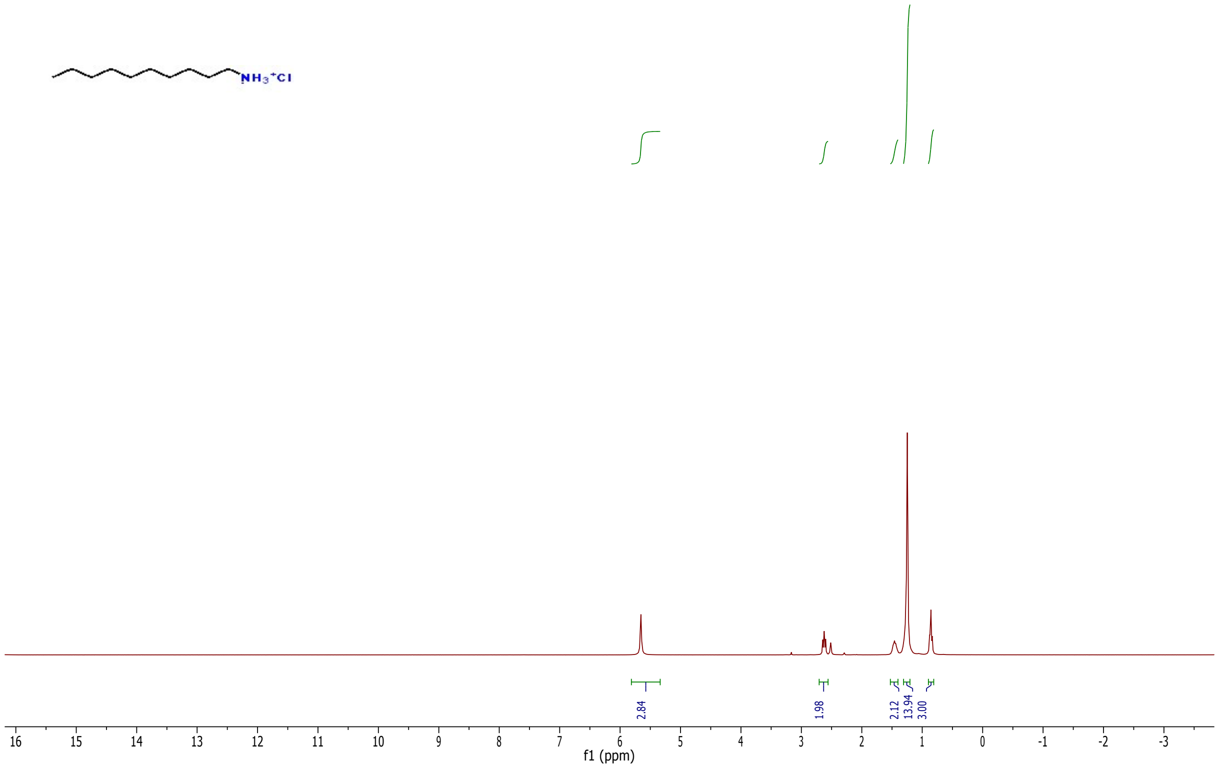

170503.f308.11.fid
Kathir/ KM10-63
C13CPD DMSO {C:\Bruker\TopSpin3.5pl6} 1705 8

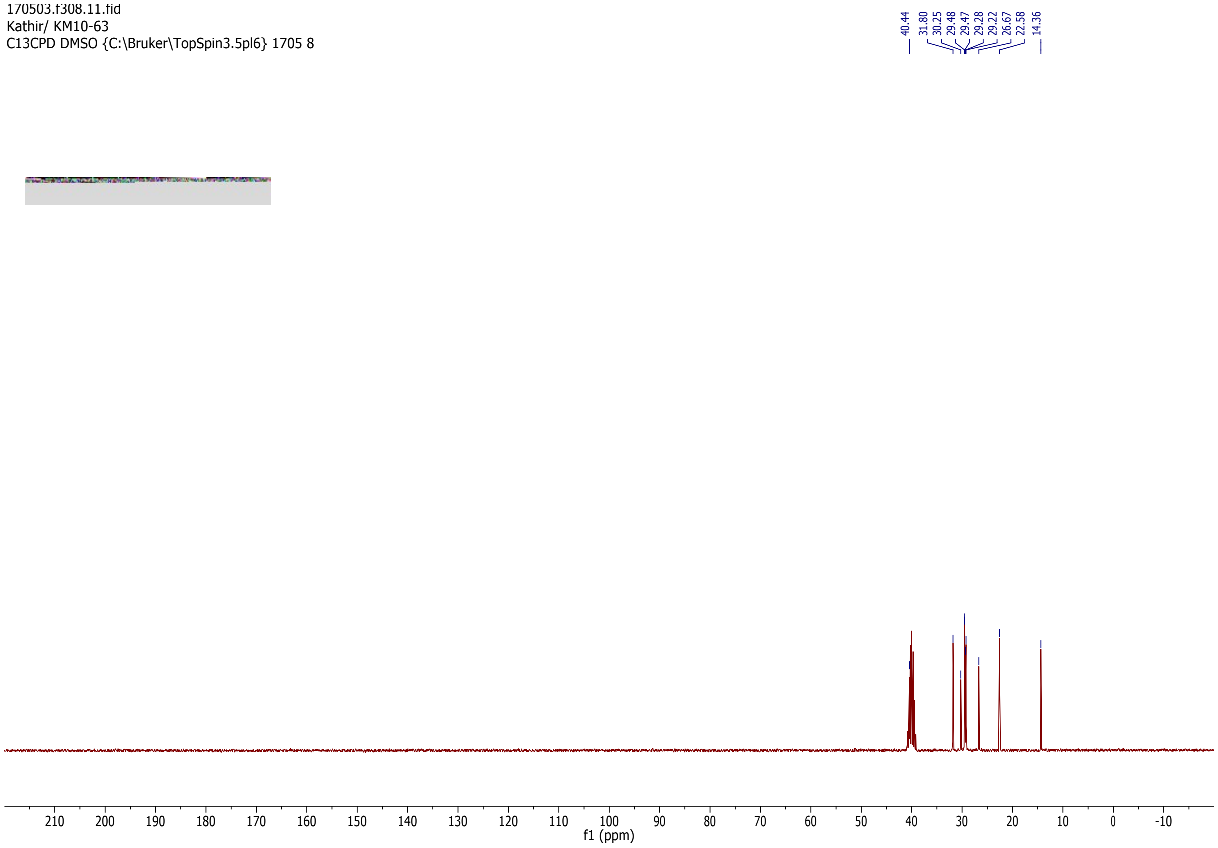

170503.f309.10.fid
Kathir/ KM10-83
PROTON DMSO {C:\Bruker\TopSpin3.5pl6} 1705 9

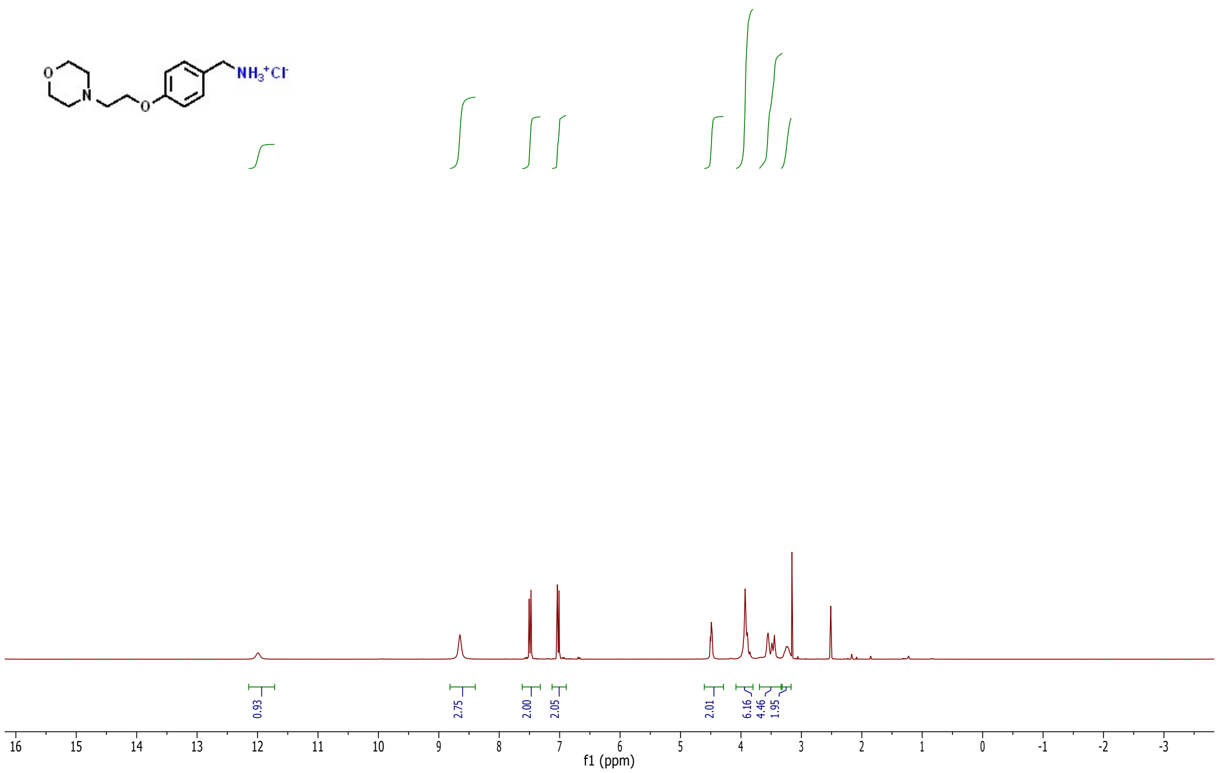

170503.f309.11.fid
Kathir/ KM10-83
C13CPD DMSO {C:\Bruker\TopSpin3.5pl6} 1705 9

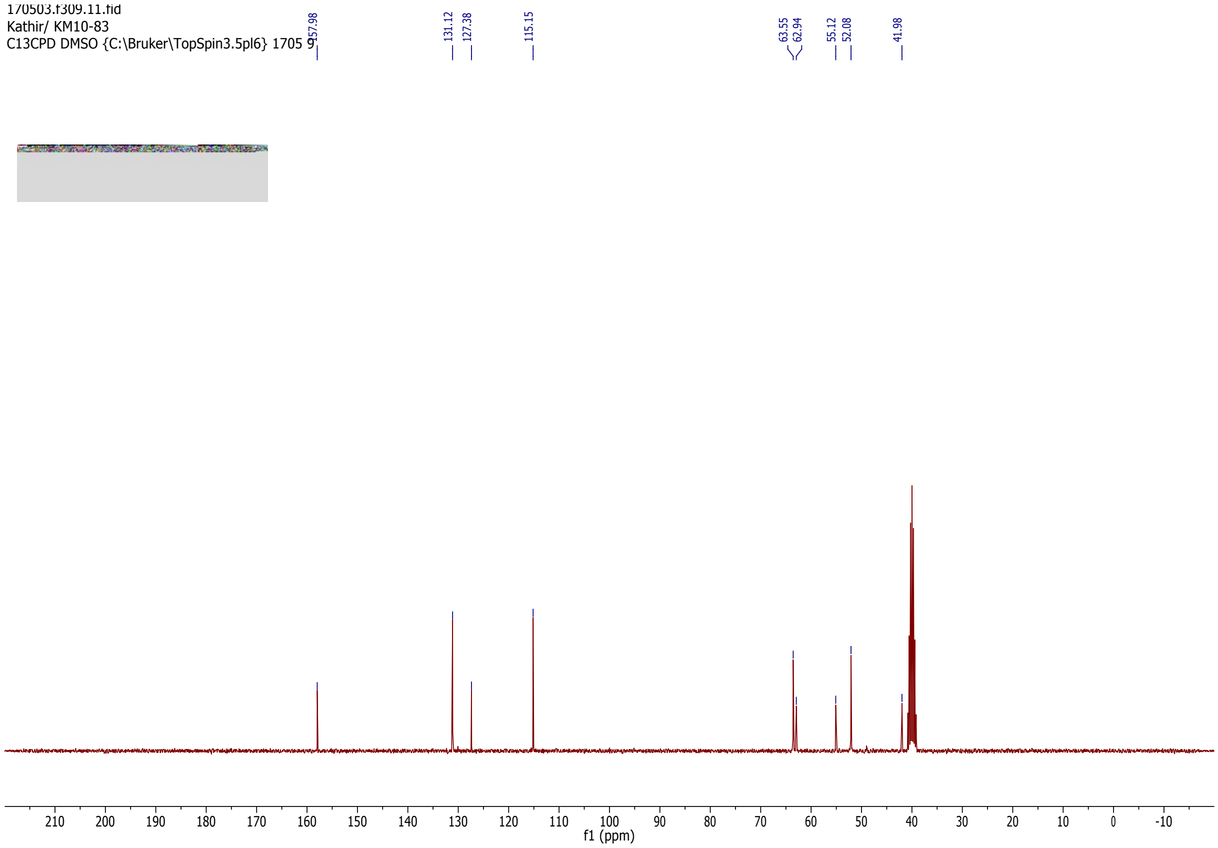

170503.f310.10.fid
Kathir/ KM10-89
PROTON DMSO {C:\Bruker\TopSpin3.5pl6} 1705 10

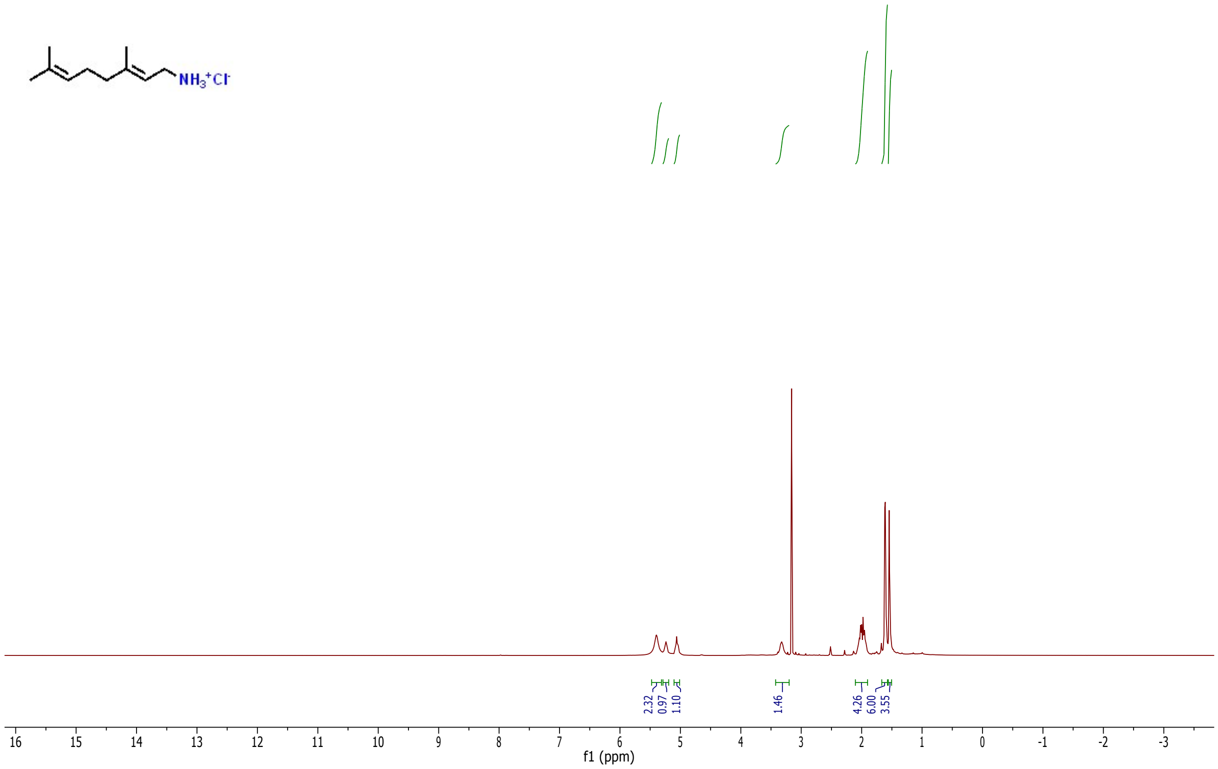

170503.f310.11.fid
Kathir/ KM10-89
C13CPD DMSO {C:\Bruker\TopSpin3.5pl6} 1705 10

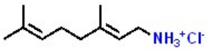

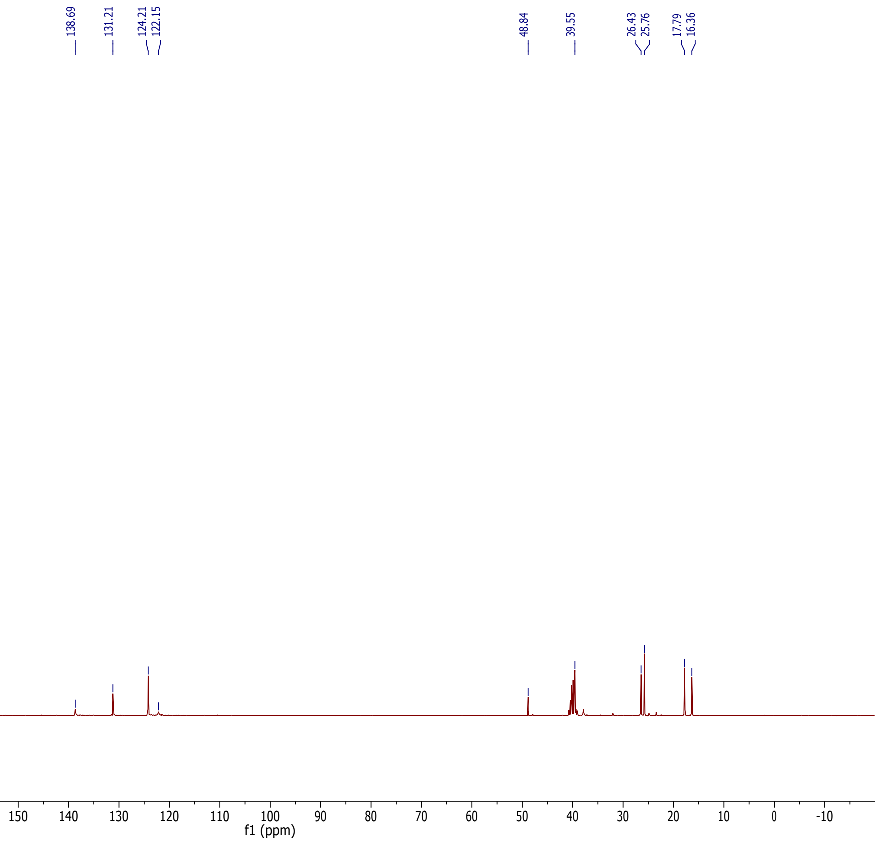

170503.f311.10.fid
Kathir/ KM10-95
PROTON DMSO {C:\Bruker\TopSpin3.5pl6} 1705 11

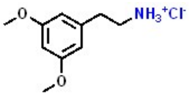

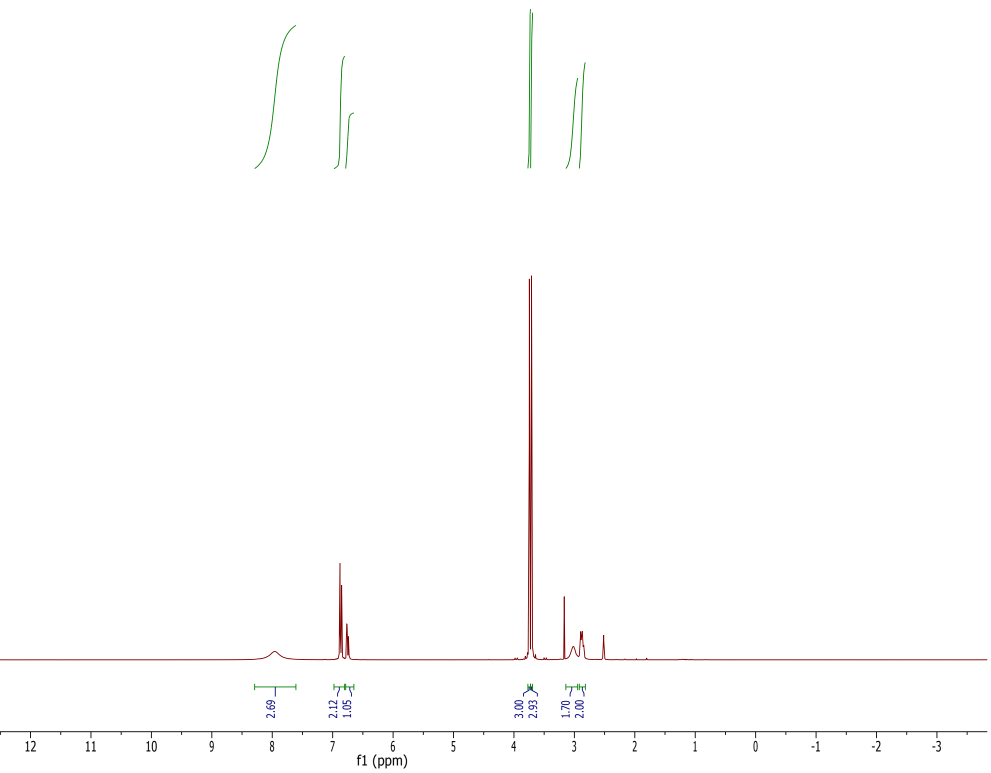

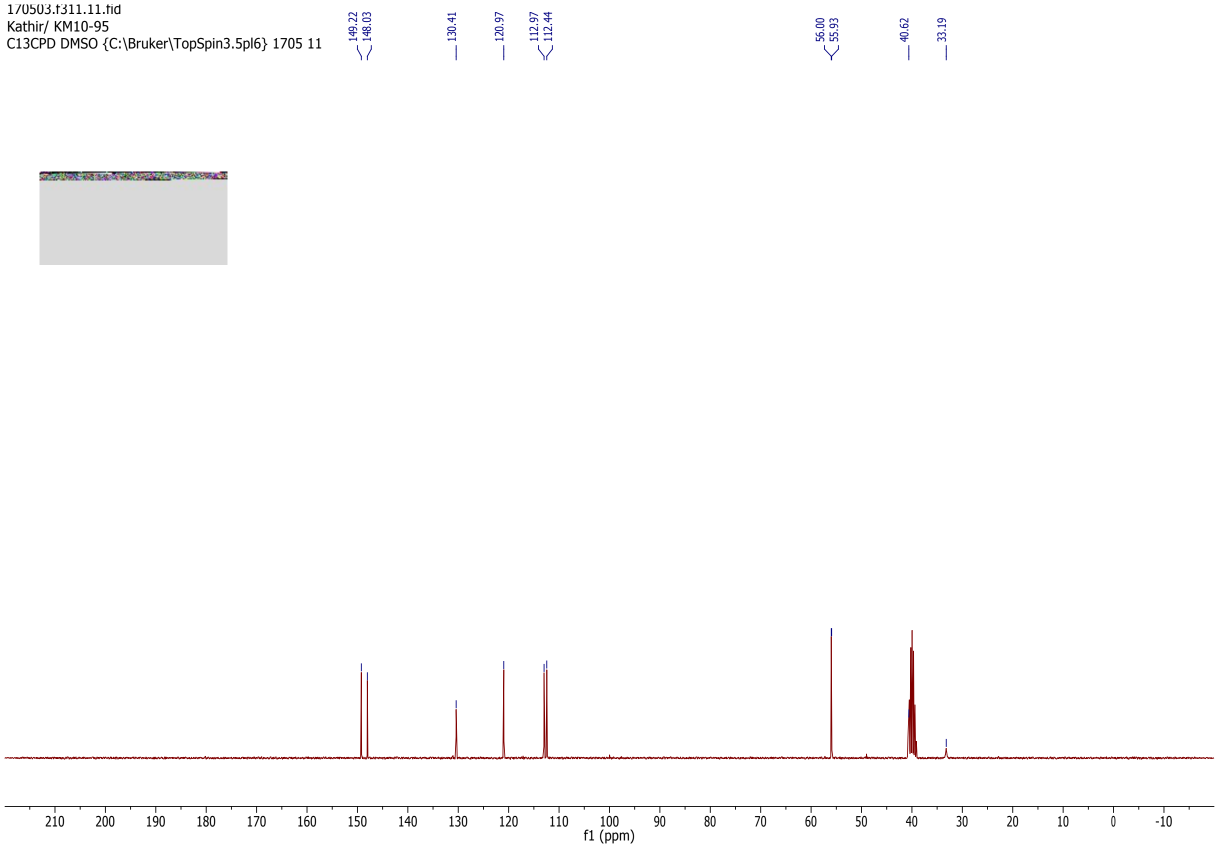
170503.f311.11.fid
Kathir/ KM10-95
C13CPD DMSO {C:\Bruker\TopSpin3.5pl6} 1705 11
149.22
148.03
130.41
120.97
112.97
112.44
56.00
55.93
40.62
33.19
f1 (ppm)

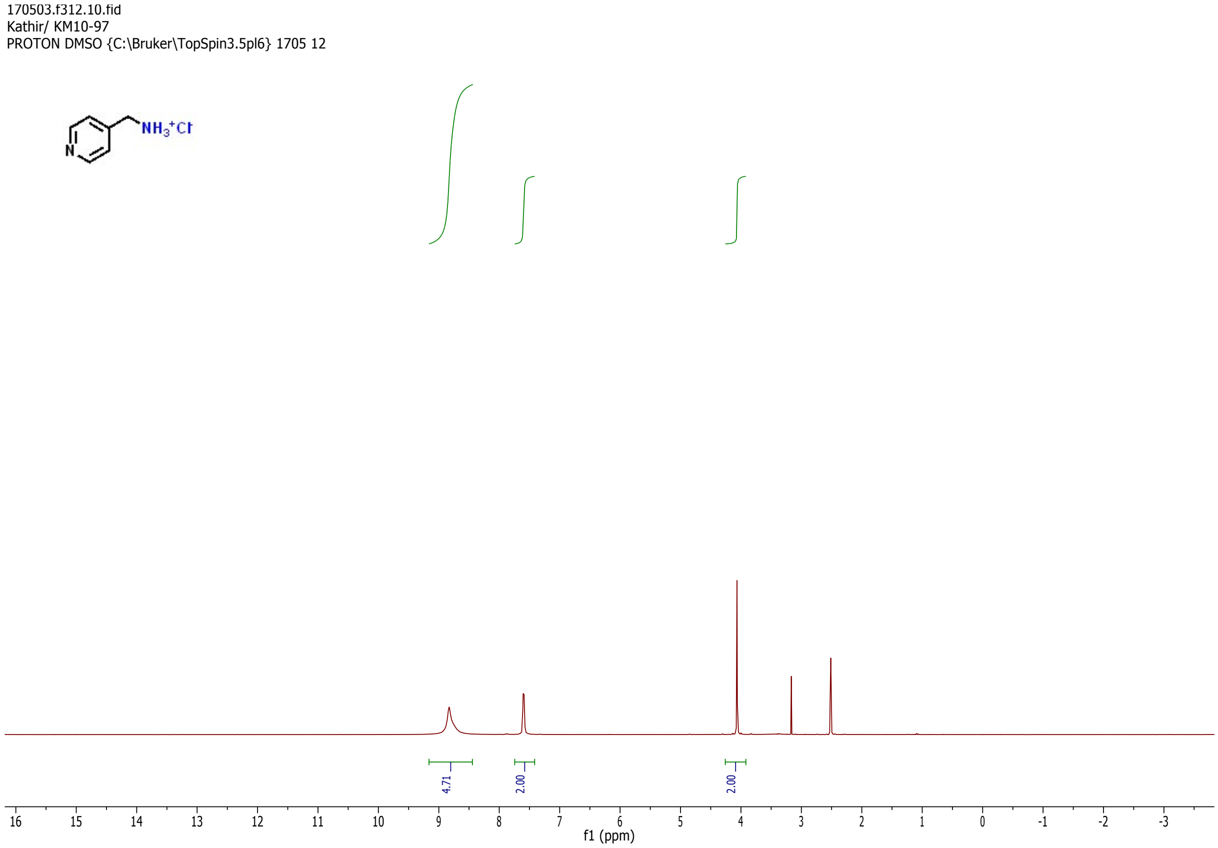
170503.f312.10.fid
Kathir/ KM10-97
PROTON DMSO {C:\Bruker\TopSpin3.5pl6} 1705 12
N
NH3+Cl-
4.71
2.00
2.00
f1 (ppm)

170503.f312.11.fid
Kathir/ KM10-97
C13CPD DMSO {C:\Bruker\TopSpin3.5pl6} 1705 12

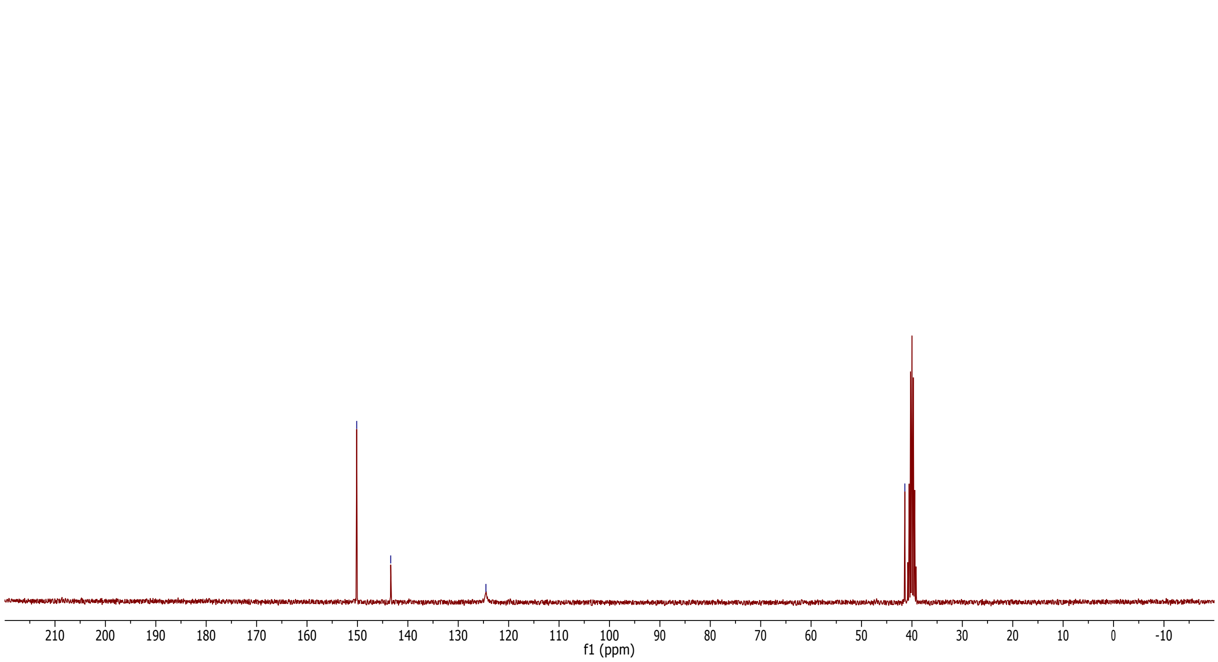

170503.f313.10.fid
Kathir/ KM10-98
PROTON DMSO {C:\Bruker\TopSpin3.5pl6} 1705 13

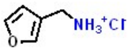

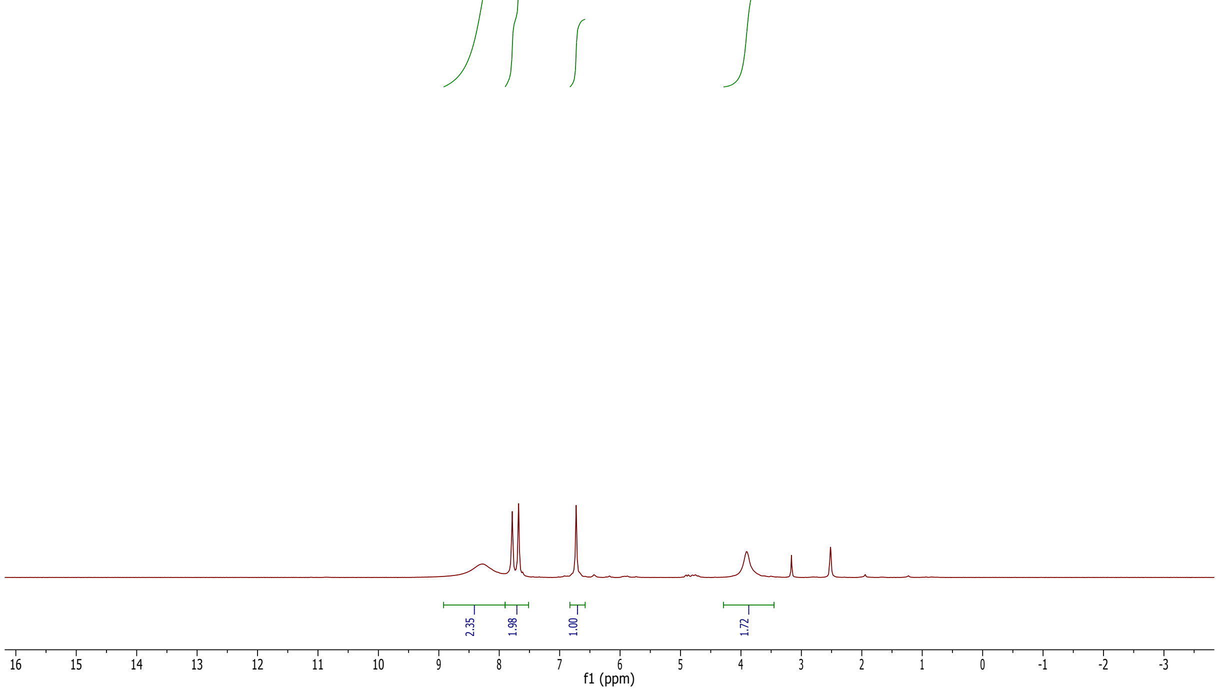

170503.f313.11.fid
Kathir/ KM10-98
C13CPD DMSO {C:\Bruker\TopSpin3.5pl6} 1705 13

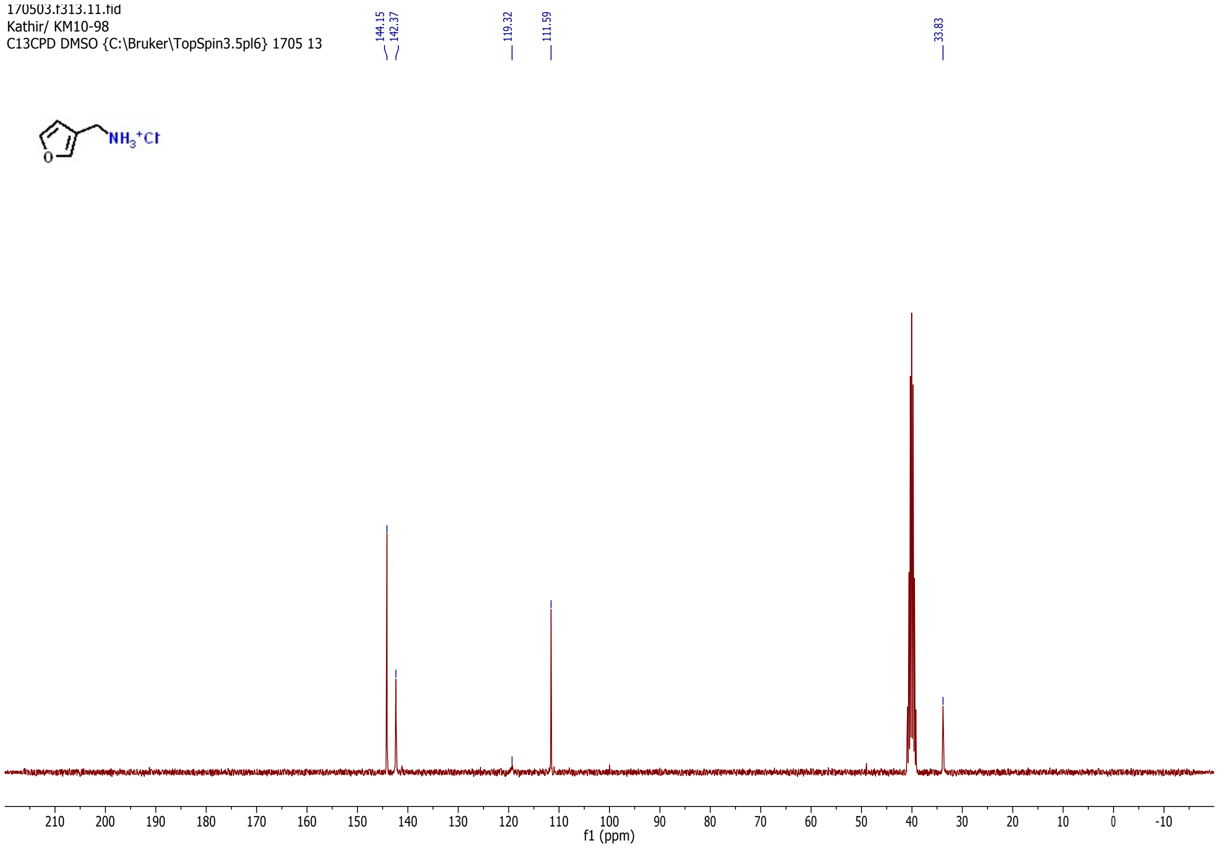

170503.f314.10.fid
Kathir/ KM10-101
PROTON DMSO {C:\Bruker\TopSpin3.5pl6} 1705 14

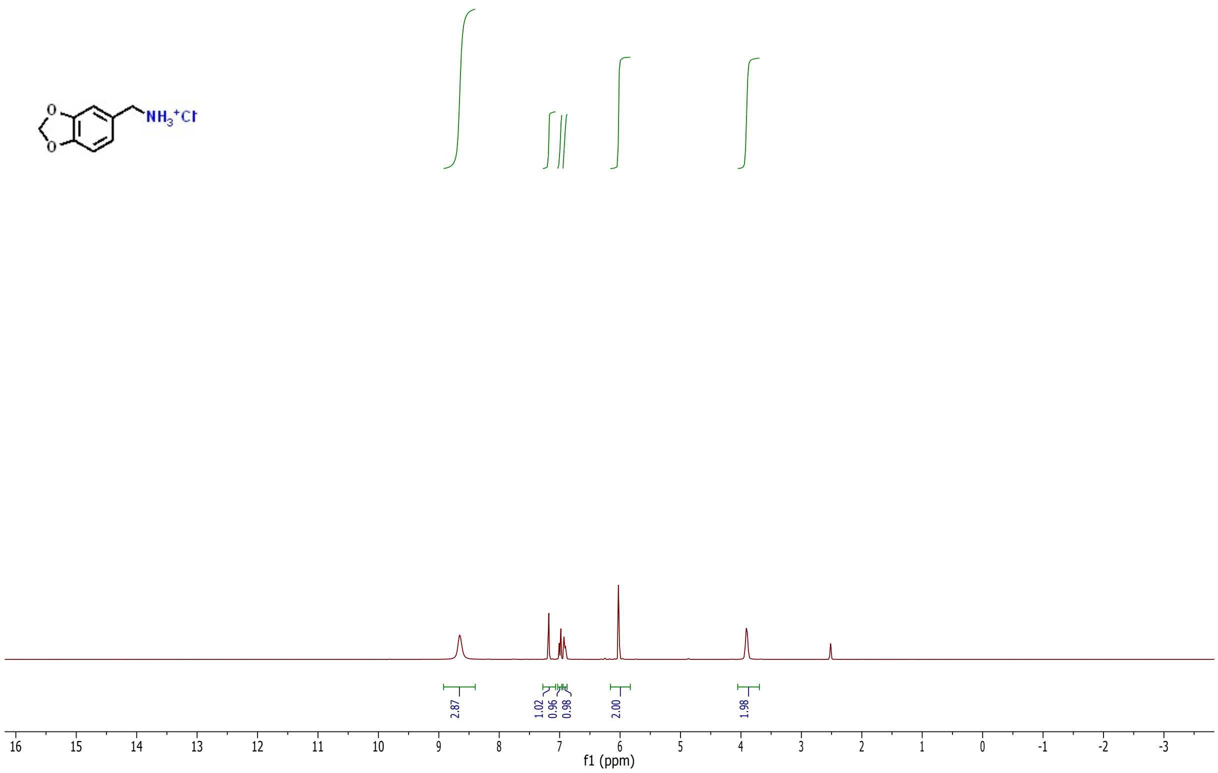

170503.f314.11.fid
Kathir/ KM10-101
C13CPD DMSO {C:\Bruker\TopSpin3.5pl6} 1705 14

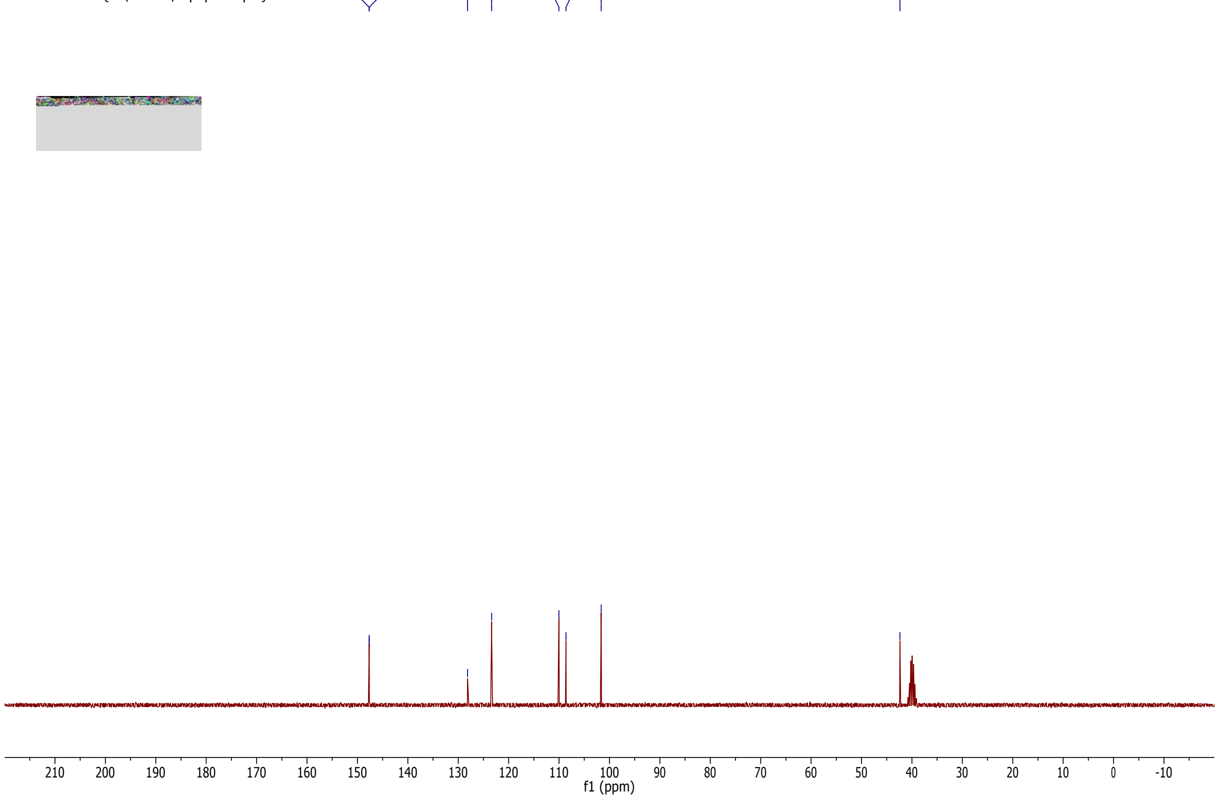

170503.f315.10.fid
Kathir/ KM10-103
PROTON DMSO {C:\Bruker\TopSpin3.5pl6} 1705 15

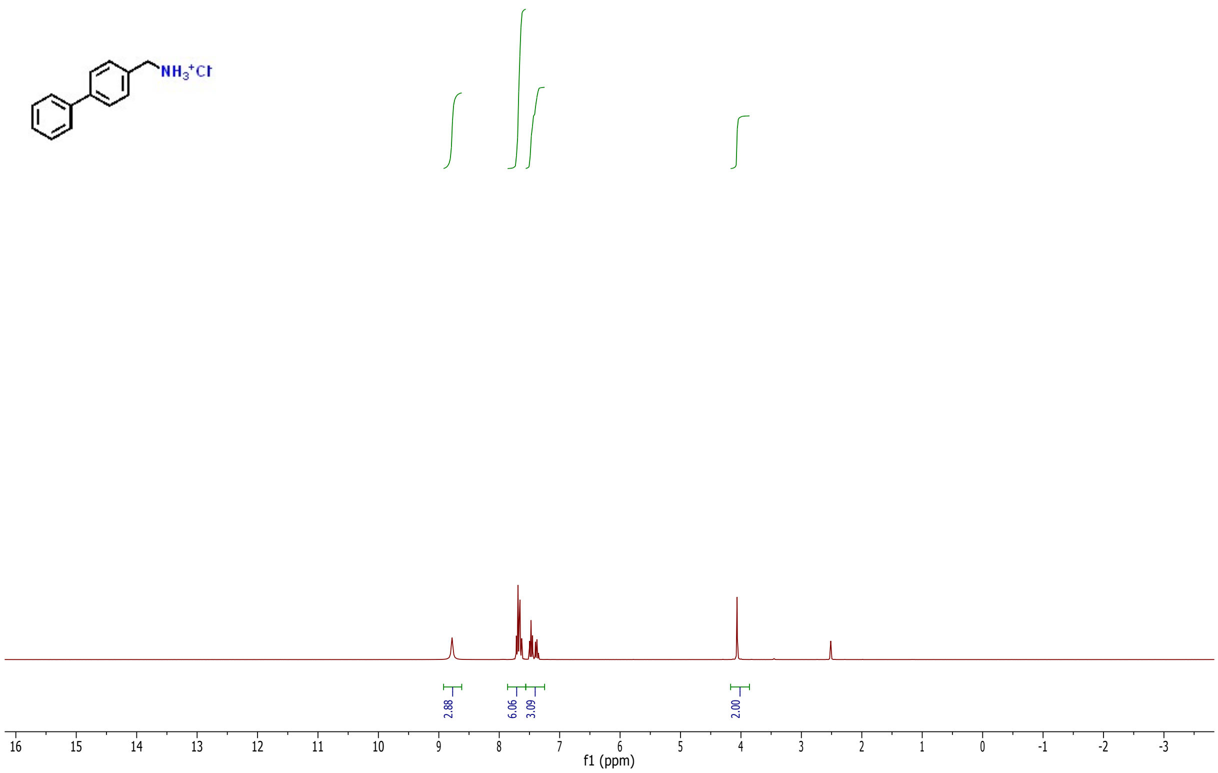

170503.f315.11.fid
Kathir/ KM10-103
C13CPD DMSO {C:\Bruker\TopSpin3.5pl6} 1705 15

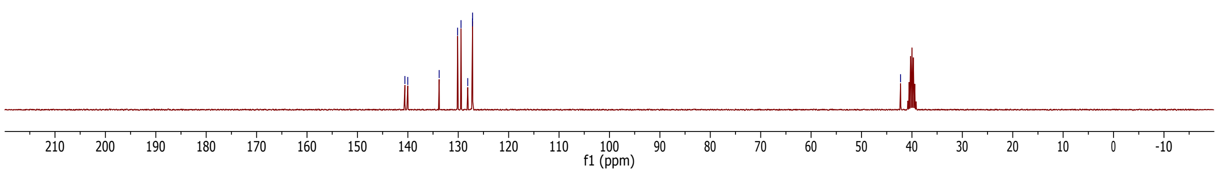

170503.f316.10.fid
Kathir/ KM10-120
PROTON DMSO {C:\Bruker\TopSpin3.5pl6} 1705 16

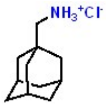

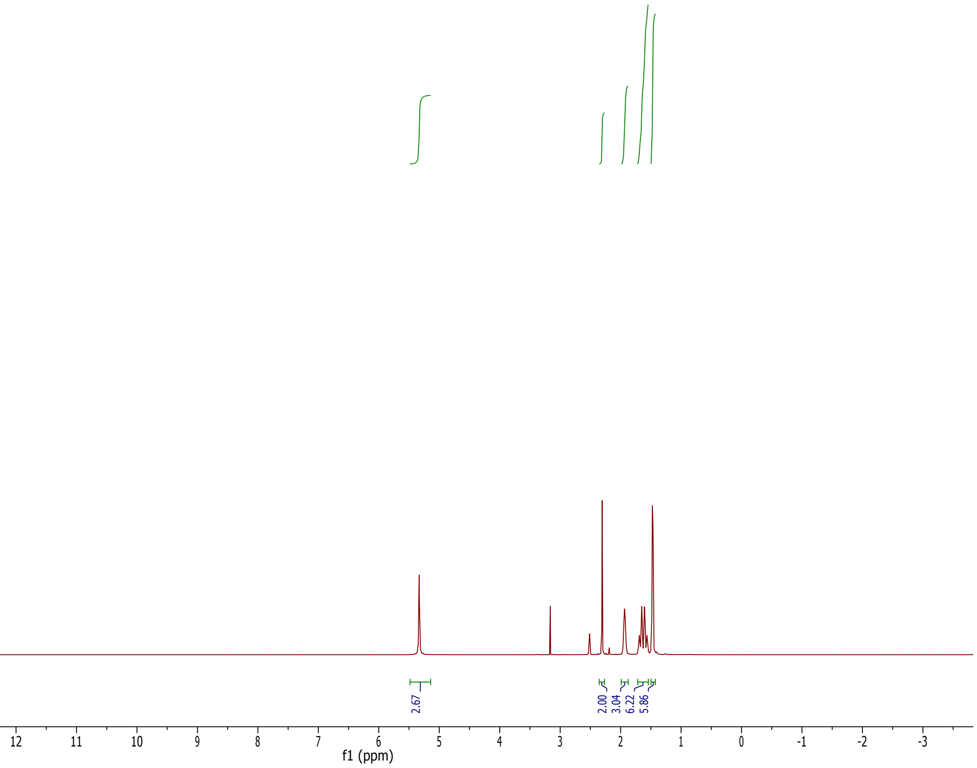

170503.f316.11.fid
Kathir/ KM10-120
C13CPD DMSO {C:\Bruker\TopSpin3.5pl6} 1705 16

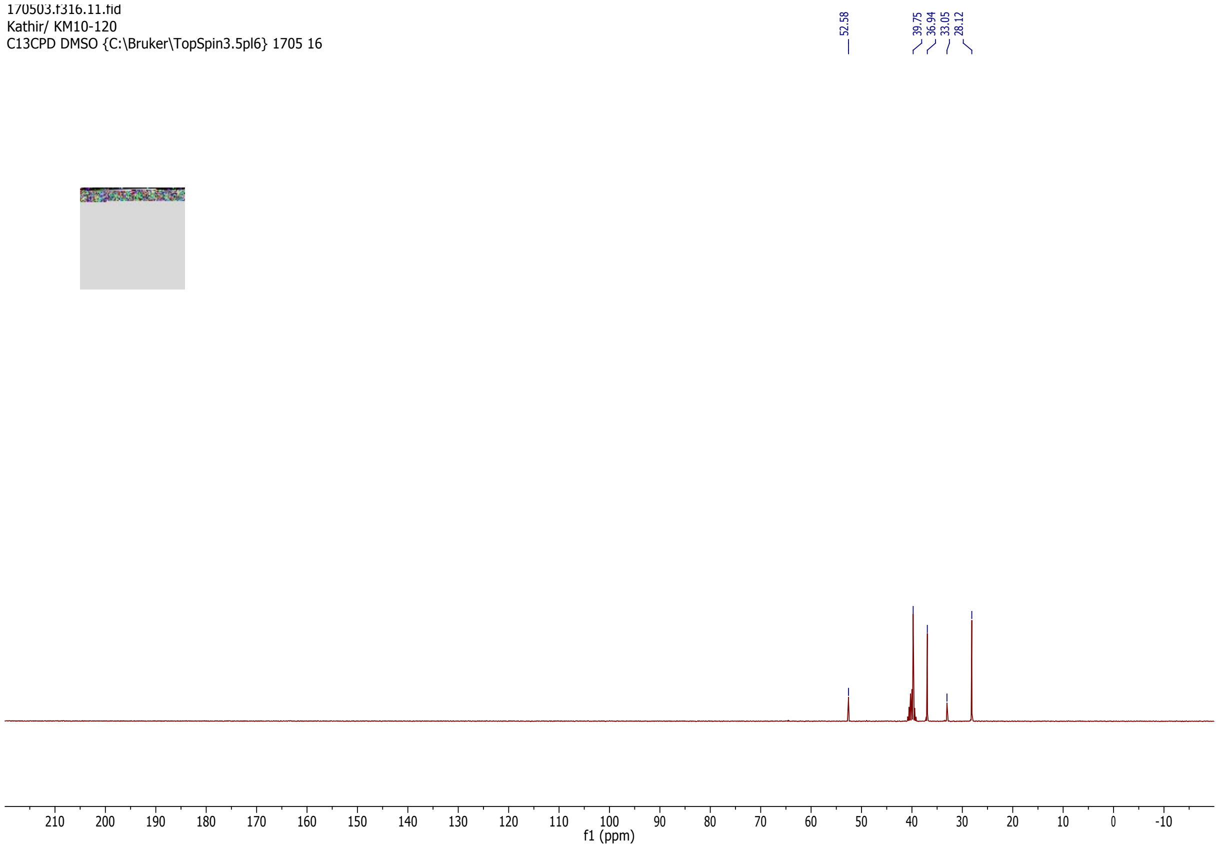

170504.305.1.fid
Kathir KMIO-54
Au1H DMSO {C:\Bruker\TopSpin3.5pl6} 1705 5

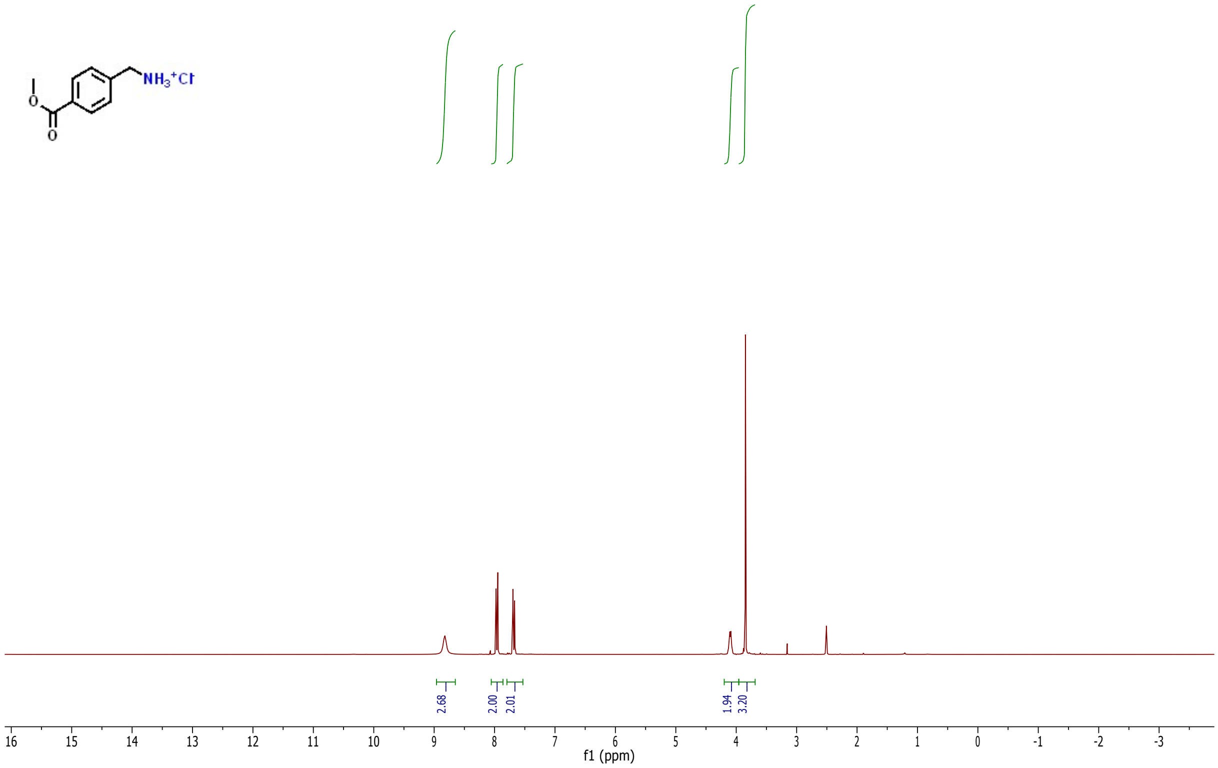

170504.305.2.fid
Kathir KMIO-54
Au13C DMSO {C:\Bruker\TopSpin3.5pl6} 1705 5

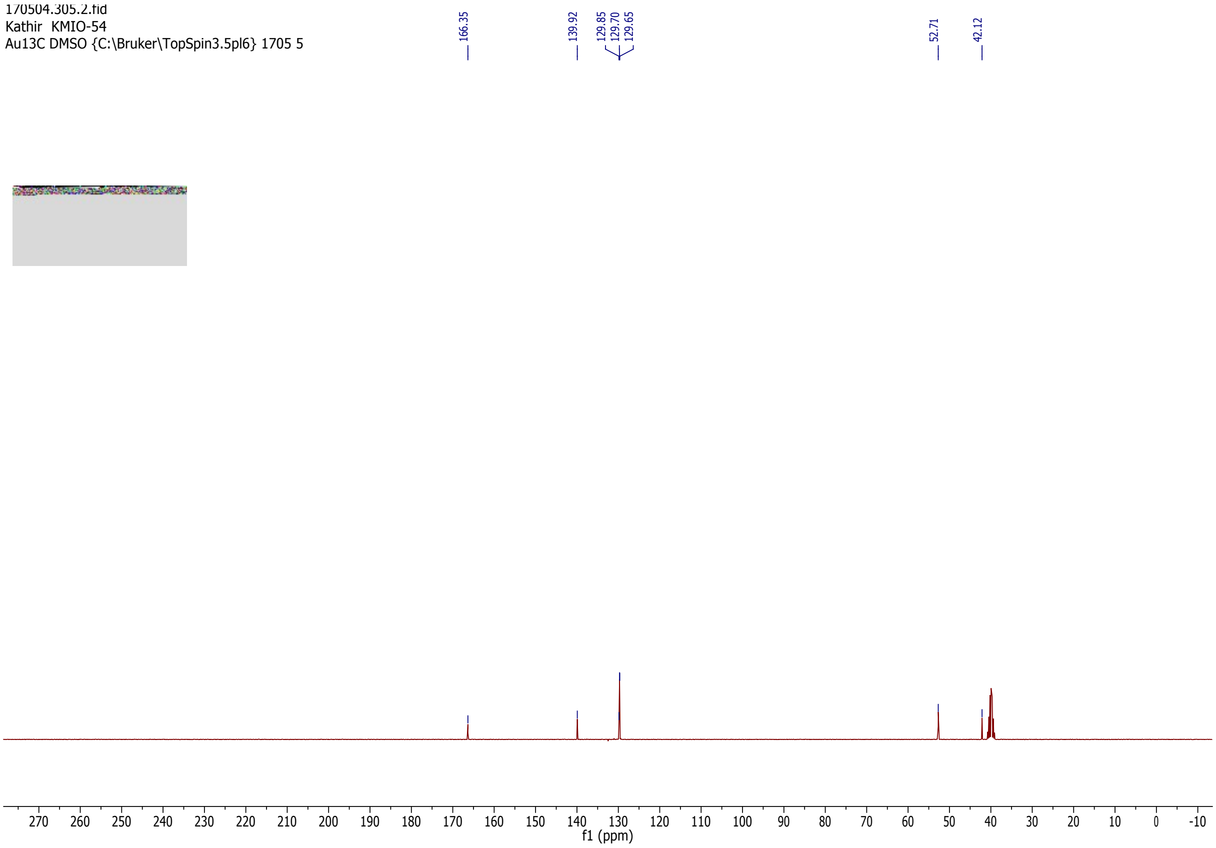

170504.307.1.fid
Kathir KMIO-109
Au1H DMSO {C:\Bruker\TopSpin3.5pl6} 1705 7

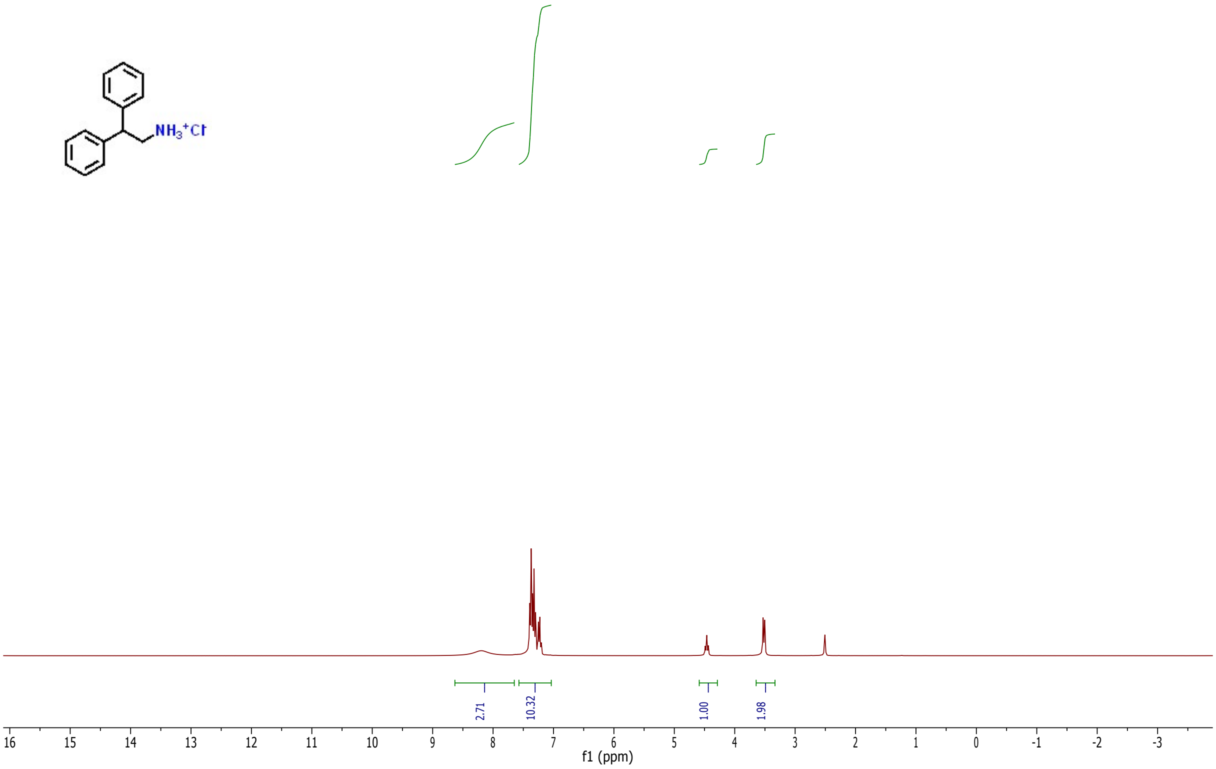

170504.307.2.fid
Kathir KMIO-109
Au13C DMSO {C:\Bruker\TopSpin3.5pl6} 1705 7

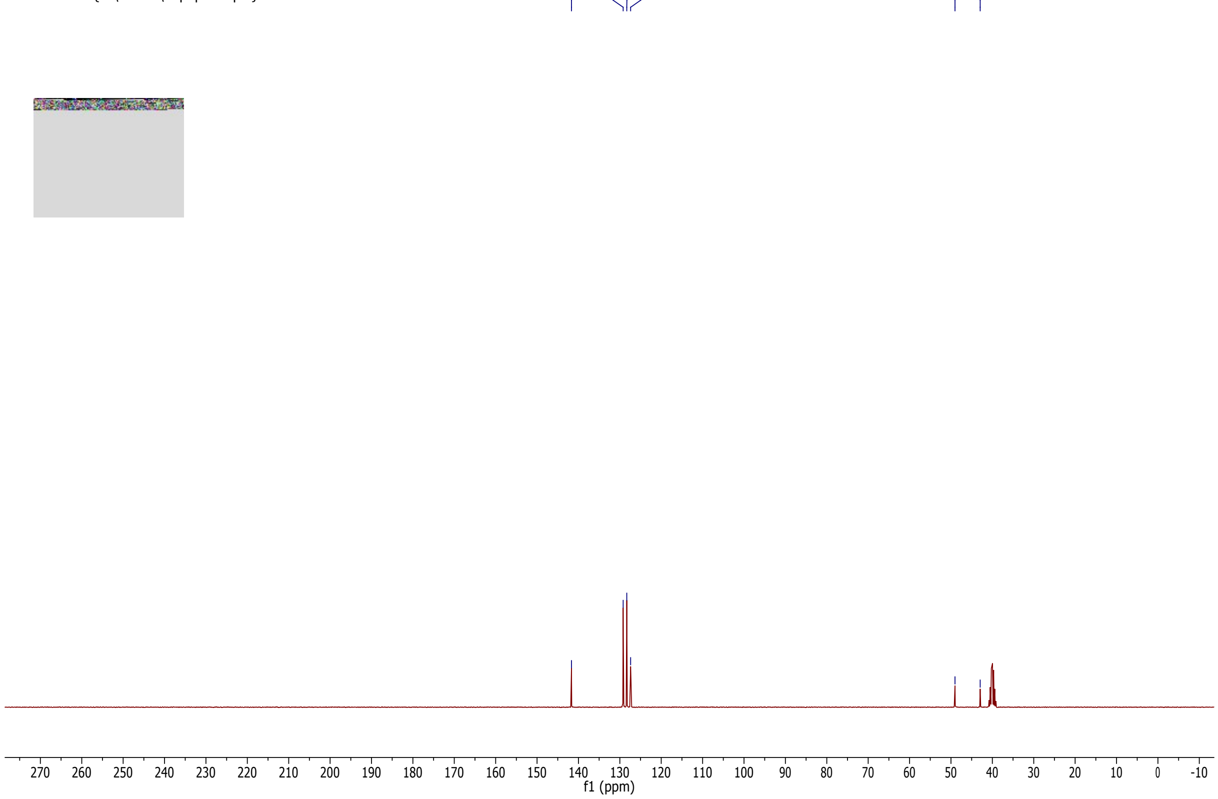

170504.308.1.fid
Kathir KMIO-110
Au1H DMSO {C:\Bruker\TopSpin3.5pl6} 1705 8

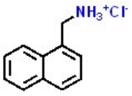

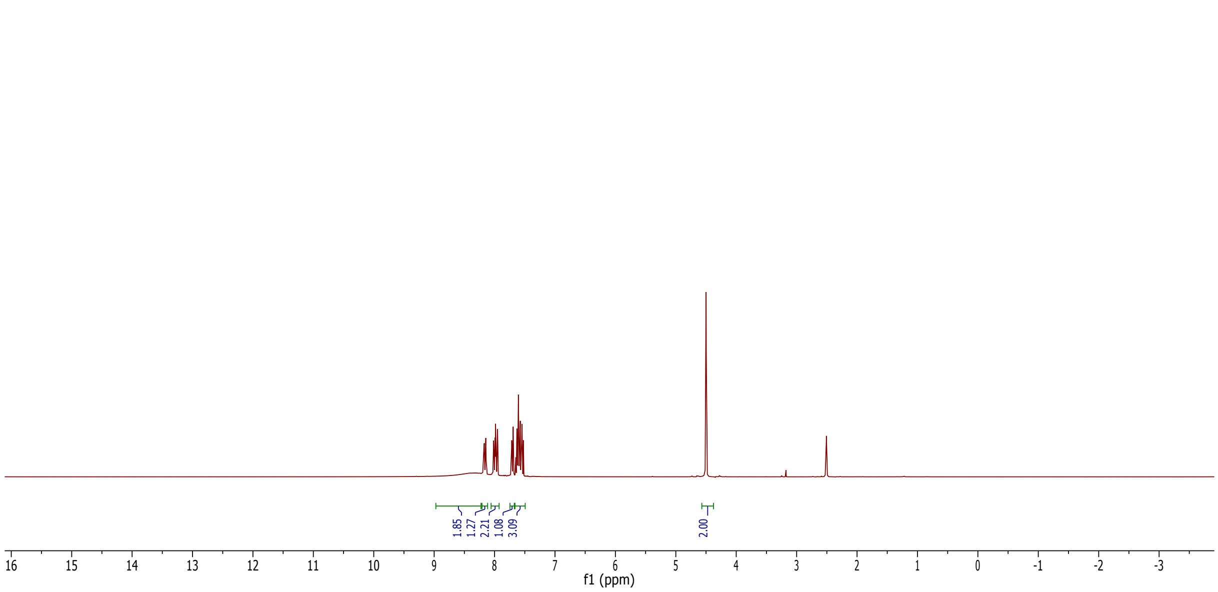

170504.308.2.fid
Kathir KMIO-110
Au13C DMSO {C:\Bruker\TopSpin3.5pl6} 1705 8

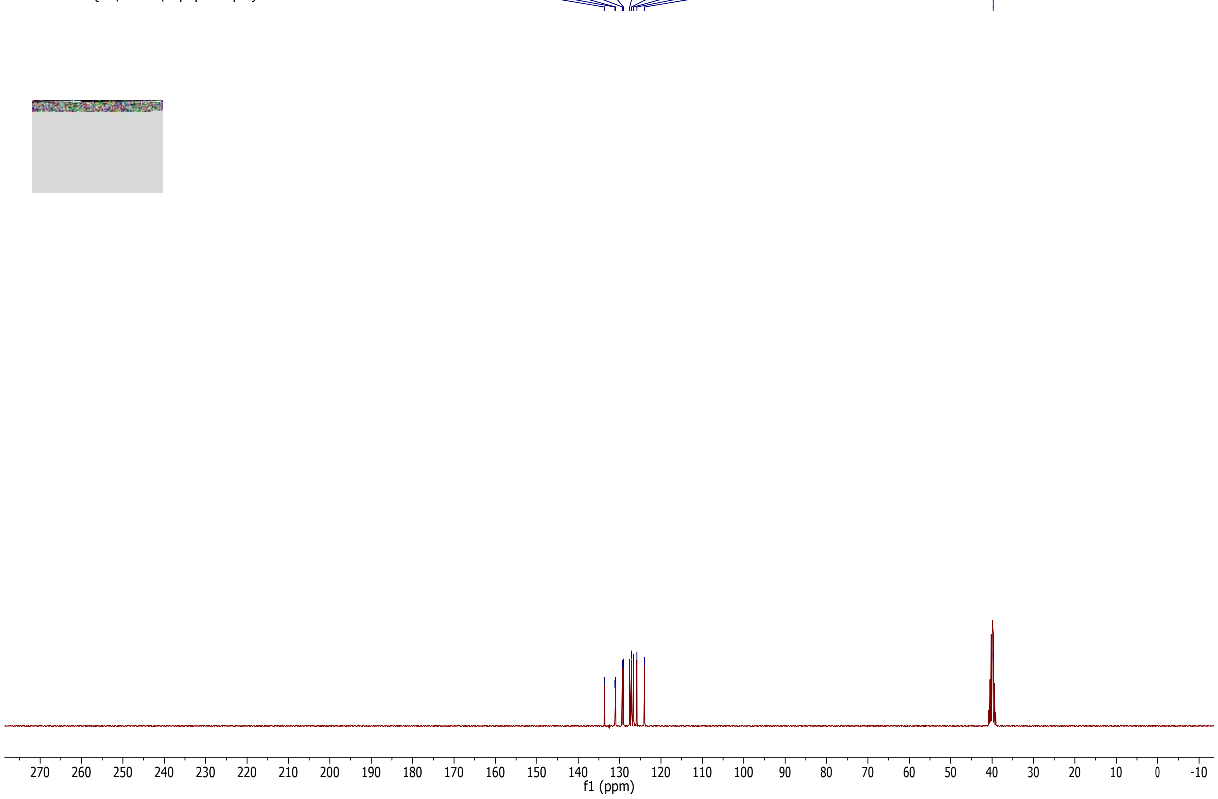

170508.302.1.fid
Kathir KM10-124
Au1H DMSO {C:\Bruker\TopSpin3.5pl6} 1705 2

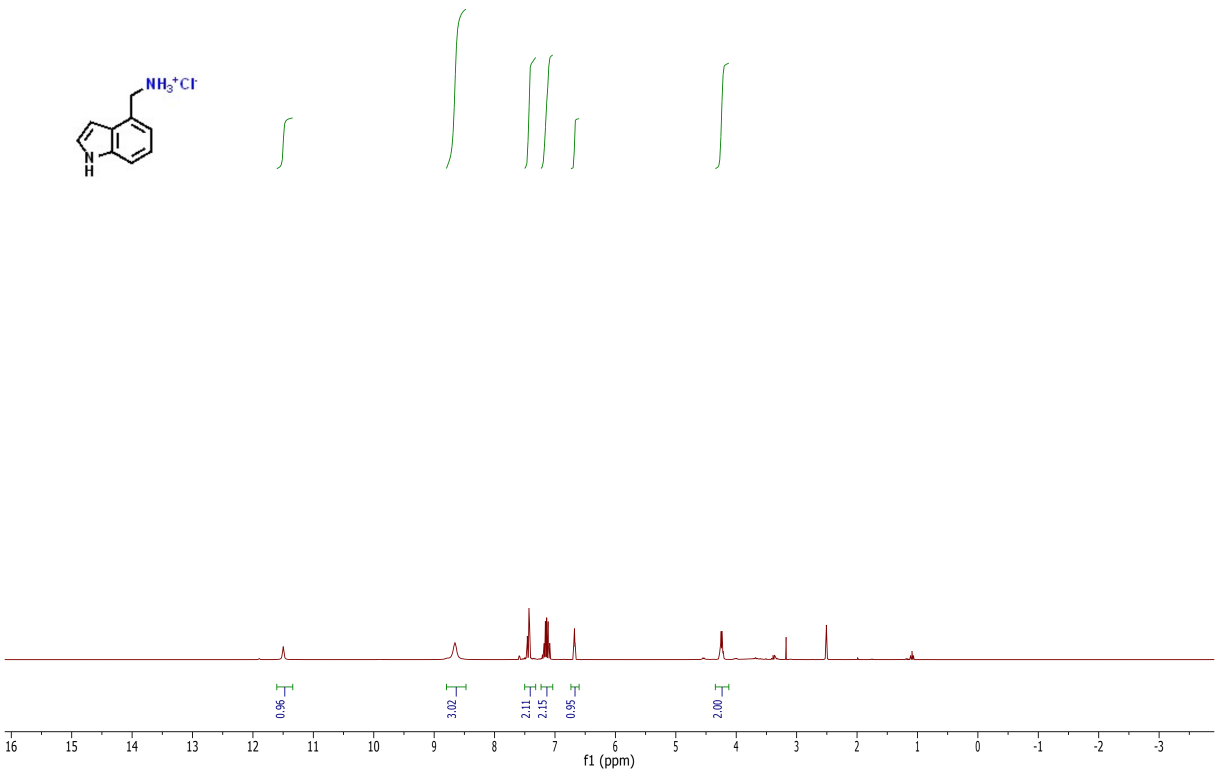

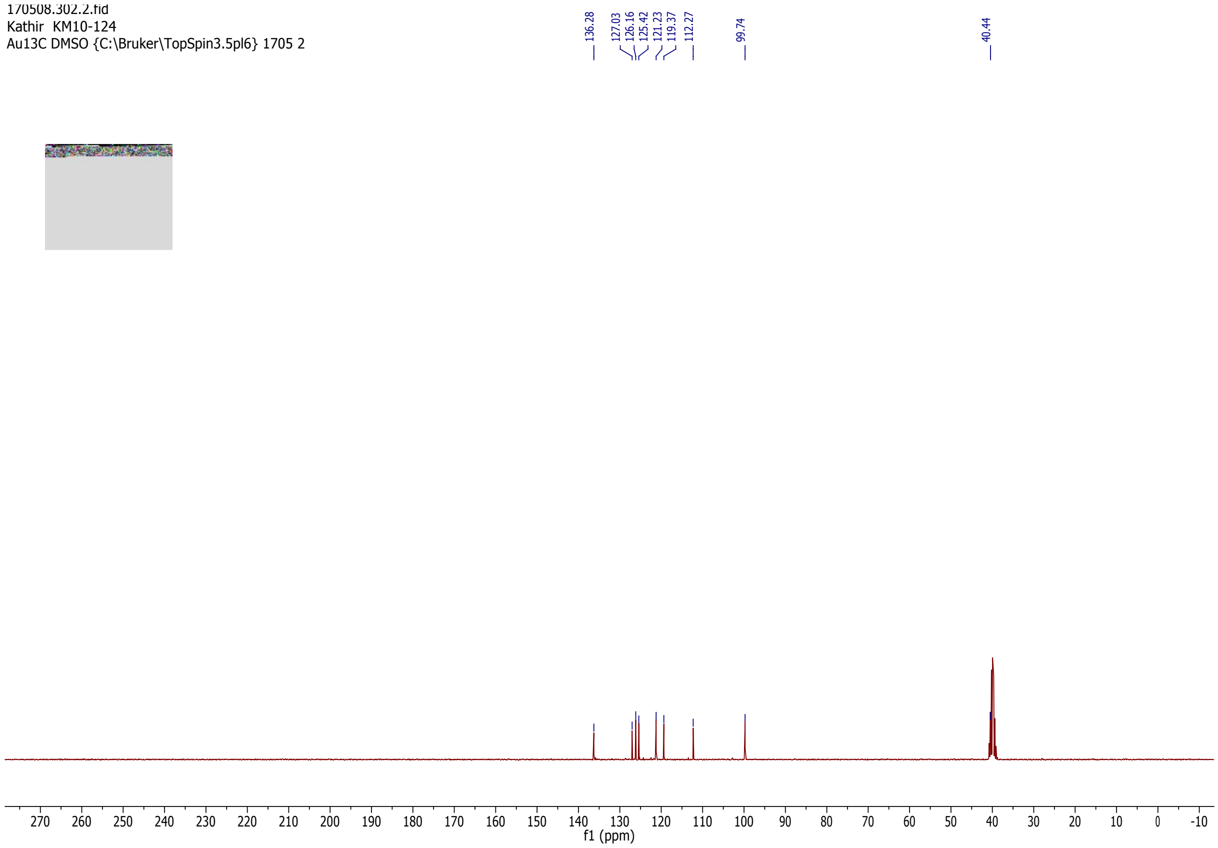
170508.302.2.fid
Kathir KM10-124
Au13C DMSO {C:\Bruker\TopSpin3.5pl6} 1705 2
136.28
127.03
126.16
125.42
121.23
119.37
112.27
99.74
40.44
270 260 250 240 230 220 210 200 190 180 170 160 150 140 130 120 110 100 90 80 70 60 50 40 30 20 10 0 -10
f1 (ppm)

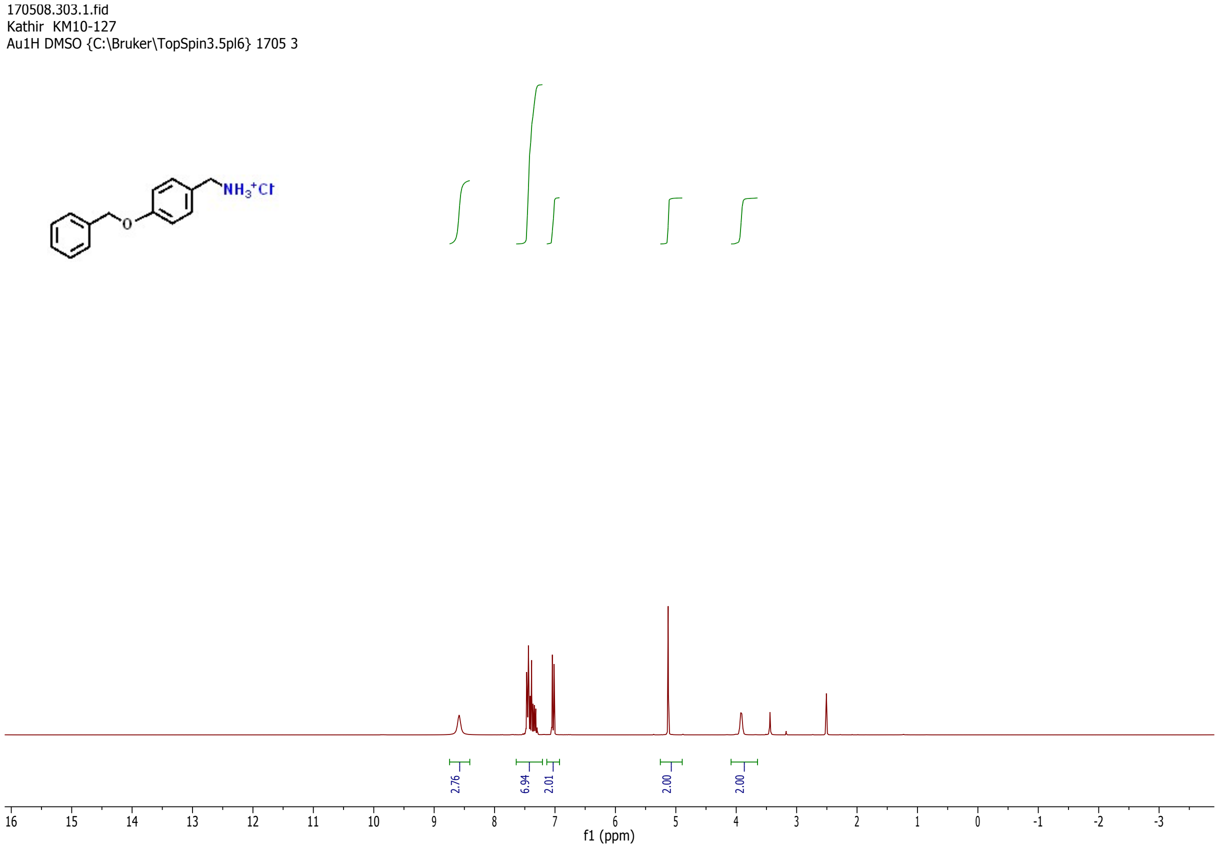
170508.303.1.fid
Kathir KM10-127
Au1H DMSO {C:\Bruker\TopSpin3.5pl6} 1705 3
O
NH3+Cl-
2.76
6.94
2.01
2.00
2.00
16 15 14 13 12 11 10 9 8 7 6 5 4 3 2 1 0 -1 -2 -3
f1 (ppm)

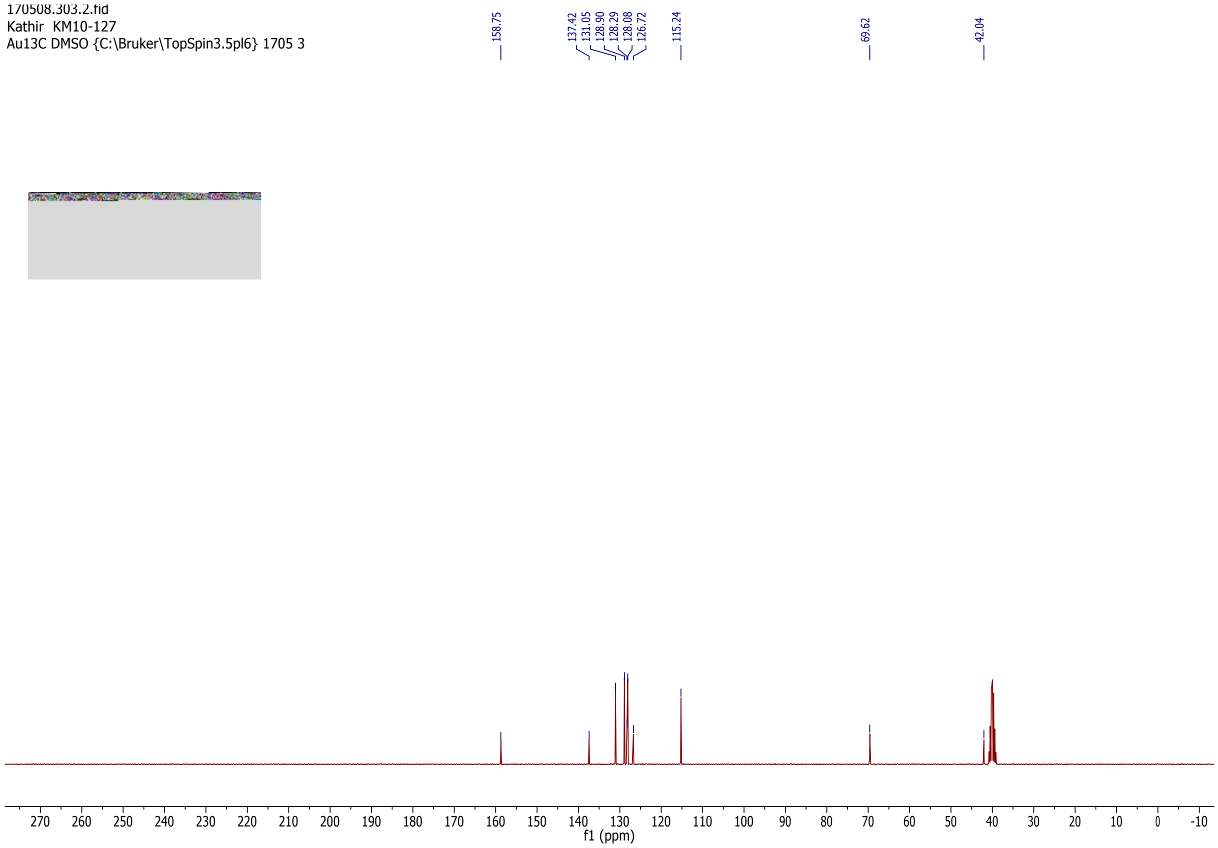
170508.303.2.fid
Kathir KM10-127
Au13C DMSO {C:\Bruker\TopSpin3.5pl6} 1705 3
158.75
137.42
131.05
128.90
128.29
128.08
126.72
115.24
69.62
42.04
270 260 250 240 230 220 210 200 190 180 170 160 150 140 130 120 110 100 90 80 70 60 50 40 30 20 10 0 -10
f1 (ppm)

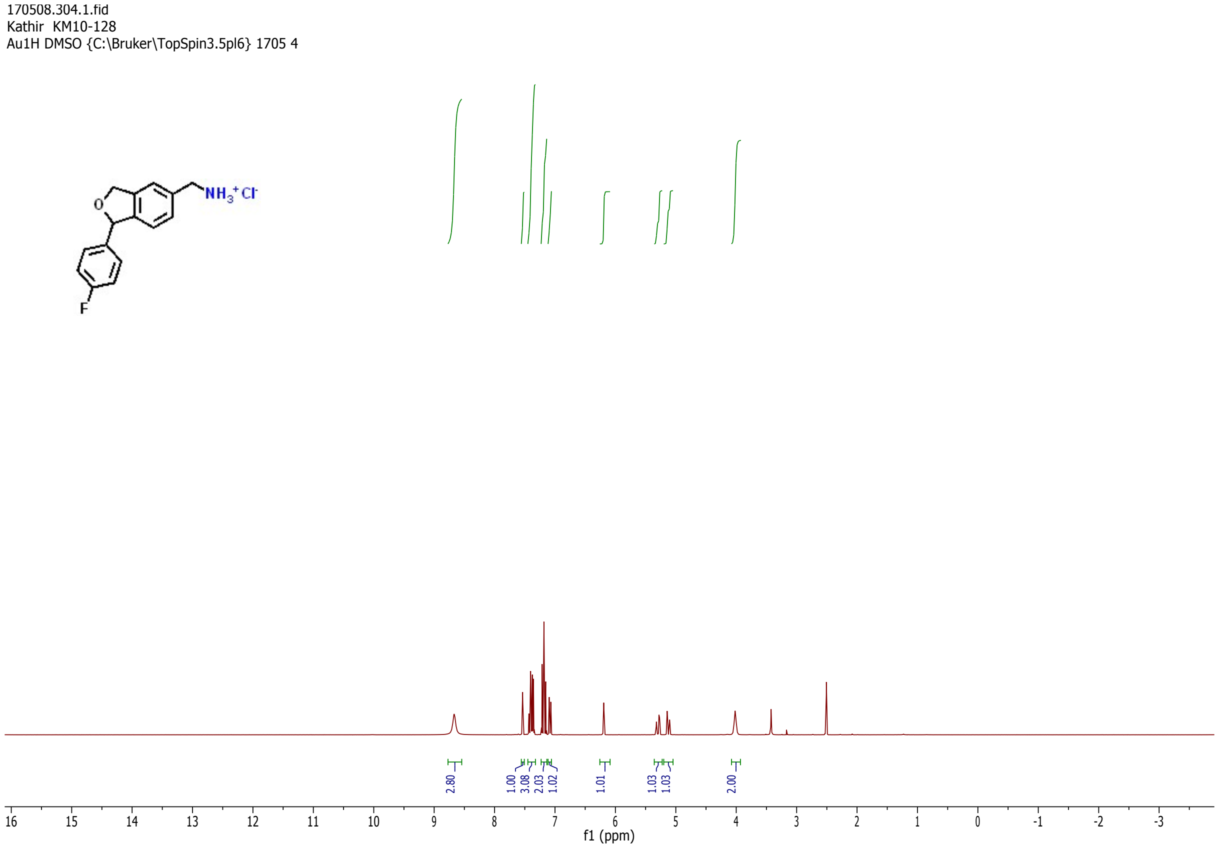
170508.304.1.fid
Kathir KM10-128
Au1H DMSO {C:\Bruker\TopSpin3.5pl6} 1705 4
O
NH3+ Cl-
F
2.80
1.00
3.08
2.03
1.02
1.01
1.03
1.03
2.00
16 15 14 13 12 11 10 9 8 7 6 5 4 3 2 1 0 -1 -2 -3
f1 (ppm)

170508.304.2.fid
Kathir KM10-128
Au13C DMSO {C:\Bruker\TopSpin3.5pl6} 1705 4

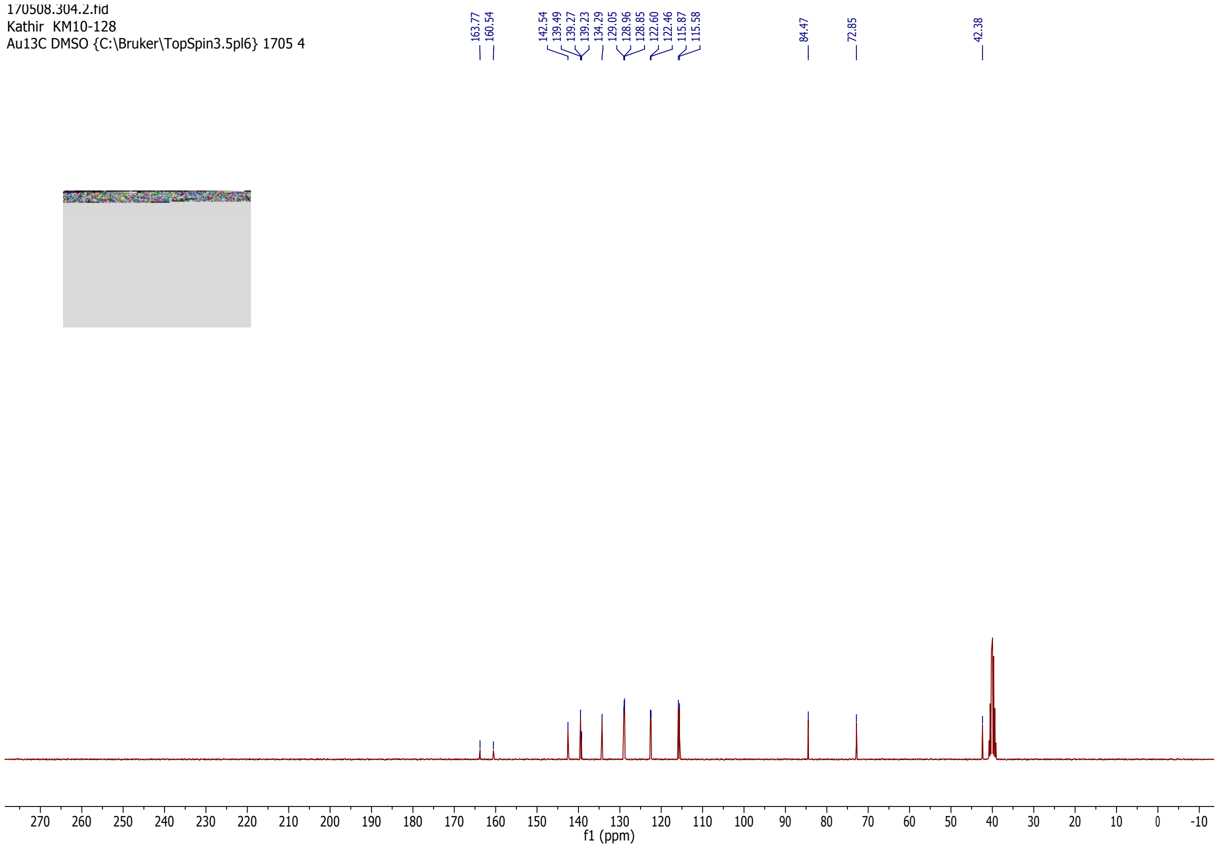

170509.302.1.fid
Kathir KM10-138
Au1H DMSO {C:\Bruker\TopSpin3.5pl6} 1705 2

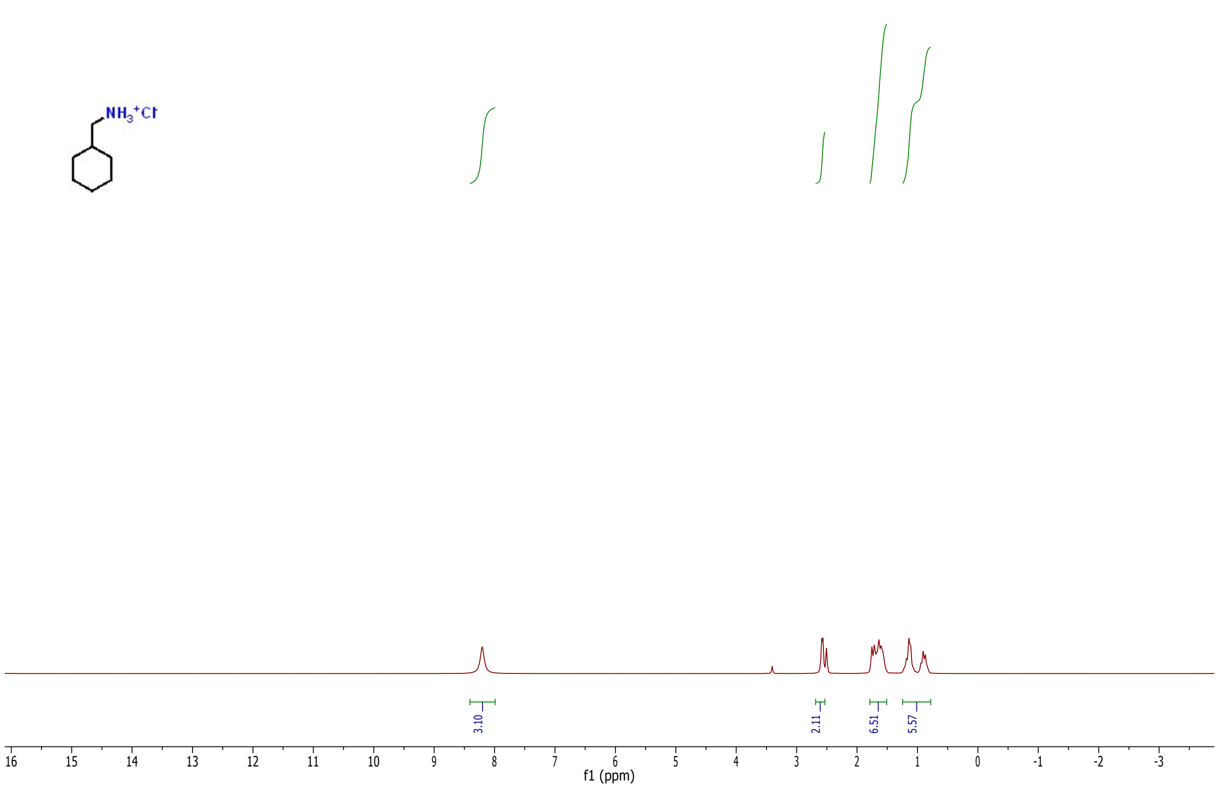

170509.302.2.fid
Kathir KM10-138
Au13C DMSO {C:\Bruker\TopSpin3.5pl6} 1705 2

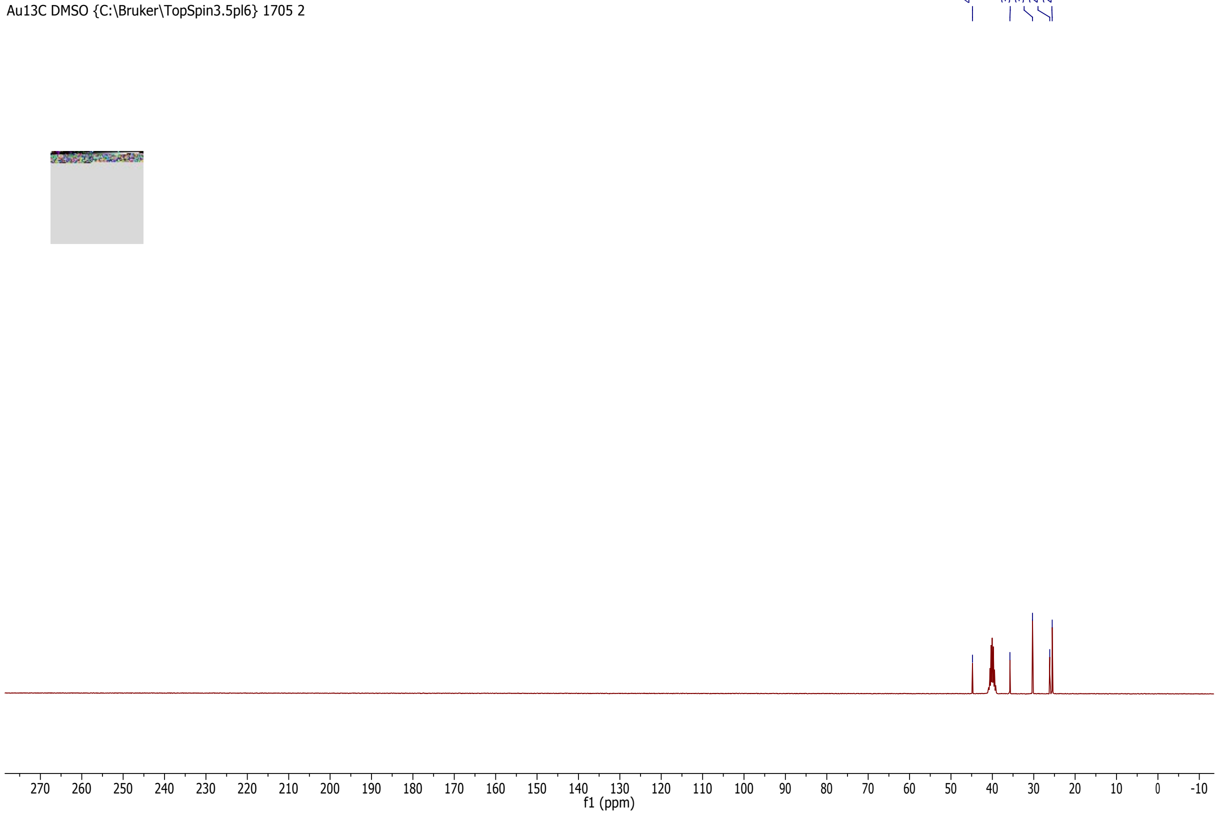

170509.304.1.fid
Kathir KM10-144
Au1H DMSO {C:\Bruker\TopSpin3.5pl6} 1705 4

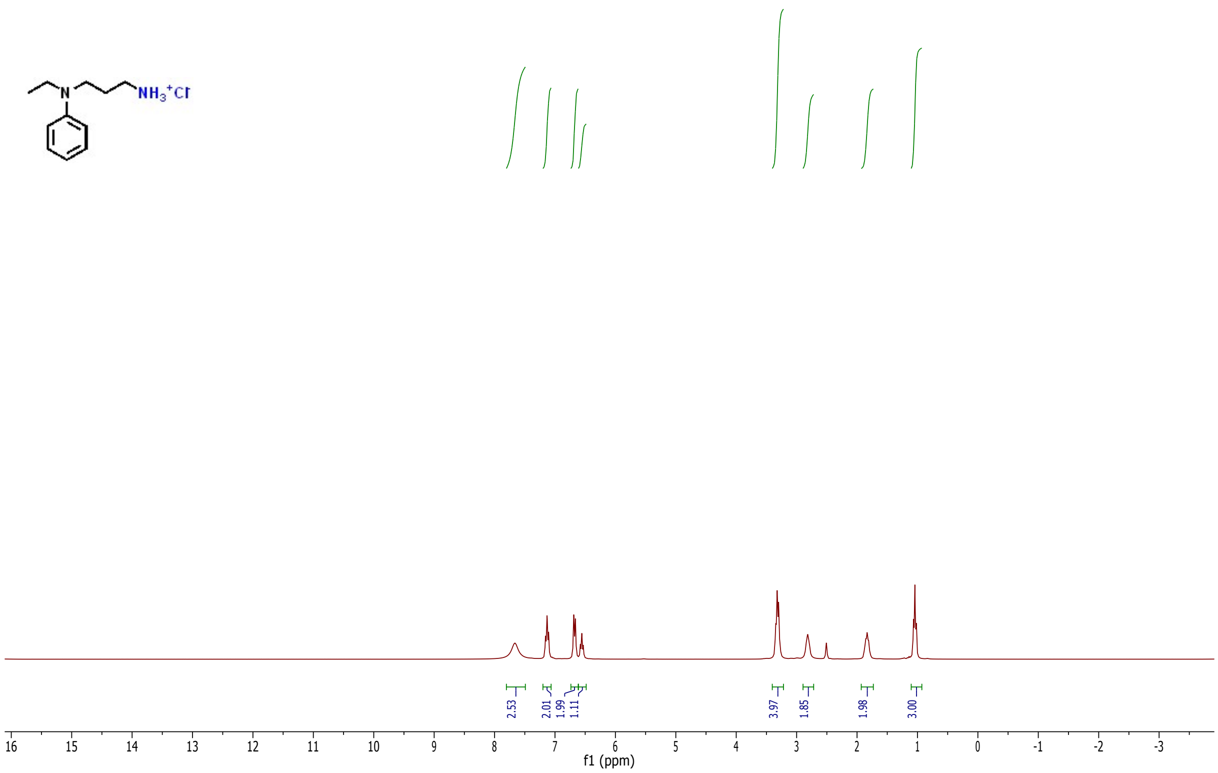

170509.304.2.fid
Kathir KM10-144
Au13C DMSO {C:\Bruker\TopSpin3.5pl6} 1705 4

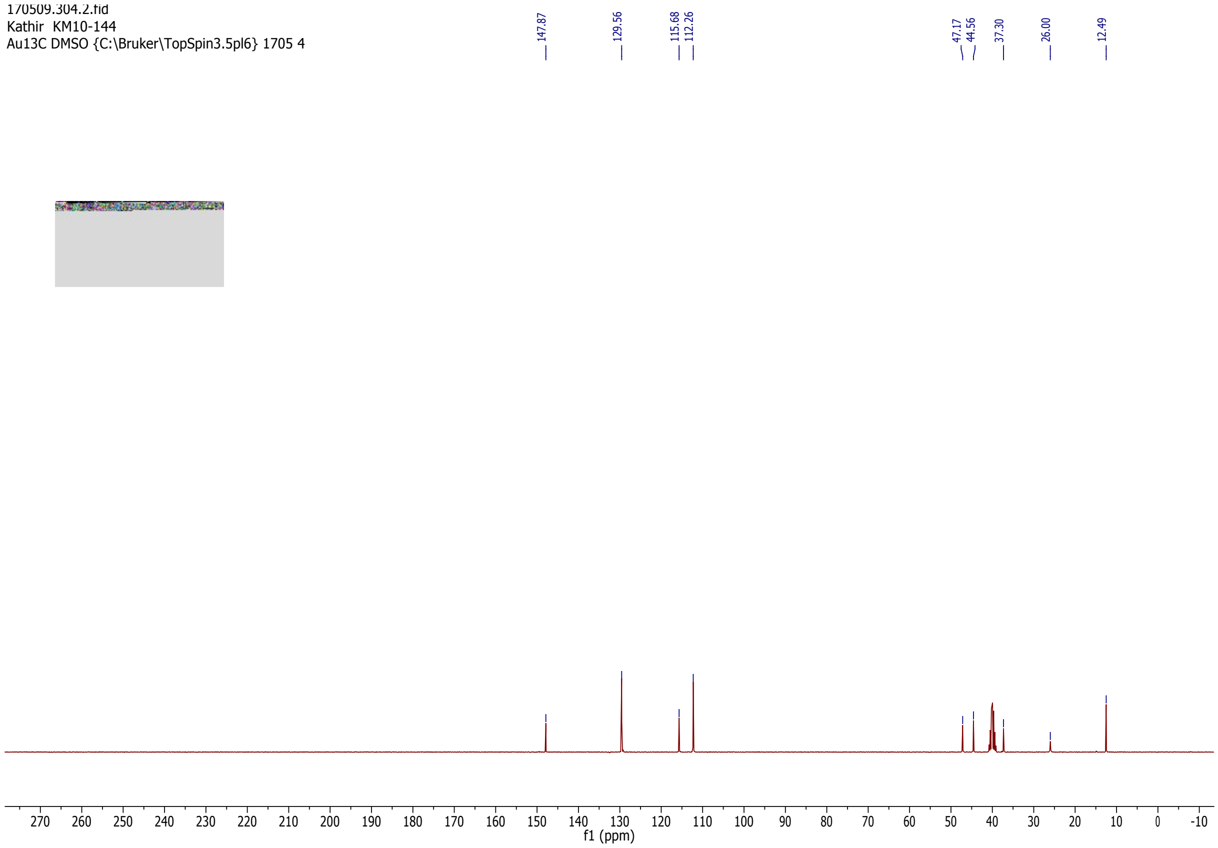

170925.301.1.fid
Kathir KM10-121
Au1H DMSO {C:\Bruker\TopSpin3.5pl6} 1709 1

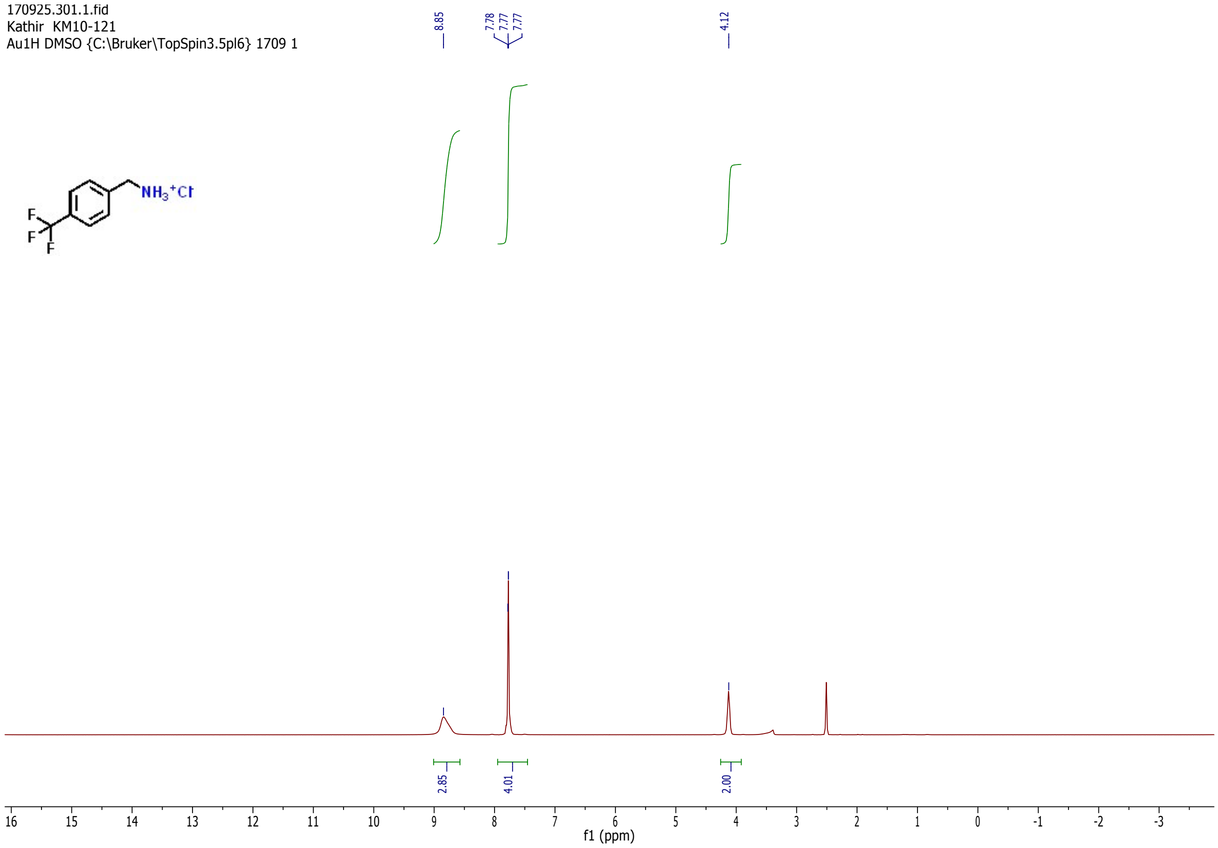

170925.301.2.fid
Kathir KM10-121
Au13C DMSO {C:\Bruker\TopSpin3.5pl6} 1709 1

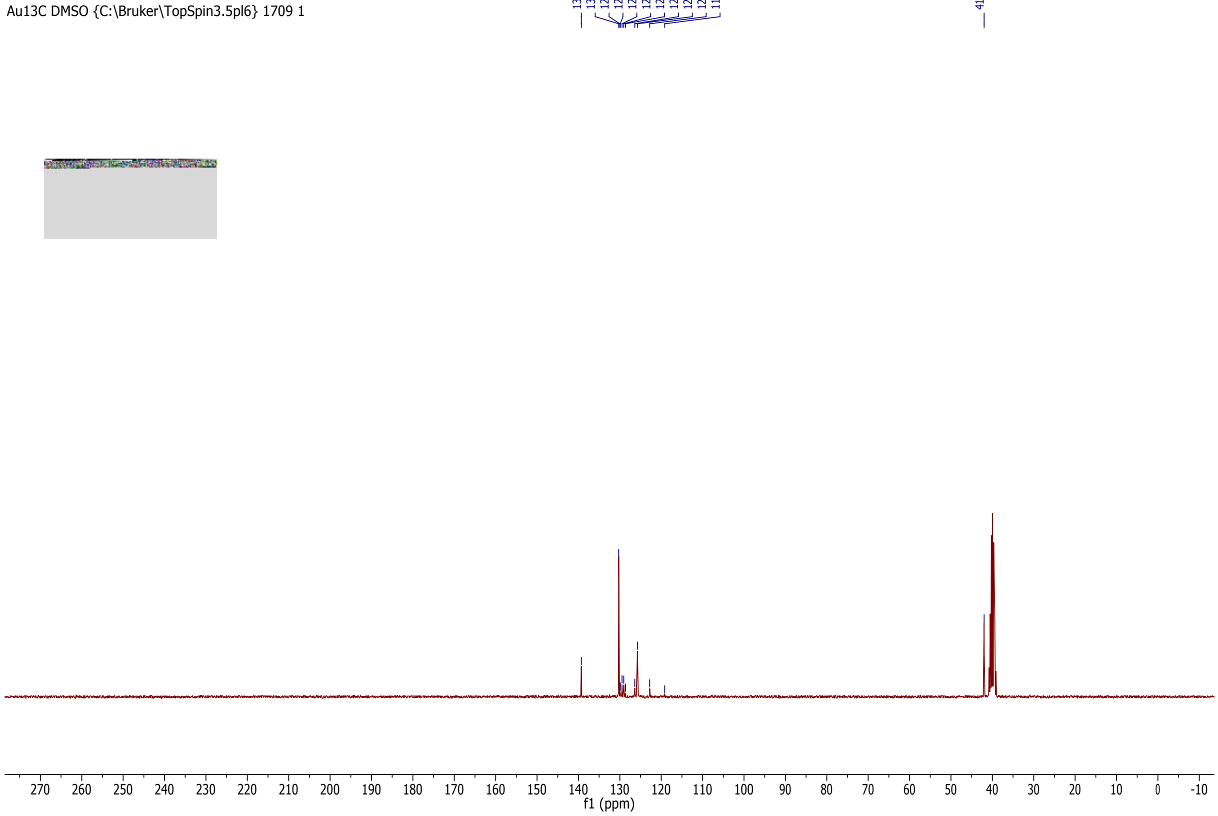

170817.f313.10.fid
Kathir/ KM10-182
PROTON CDCl3 {C:\Bruker\TopSpin3.5pl6} 1708 13

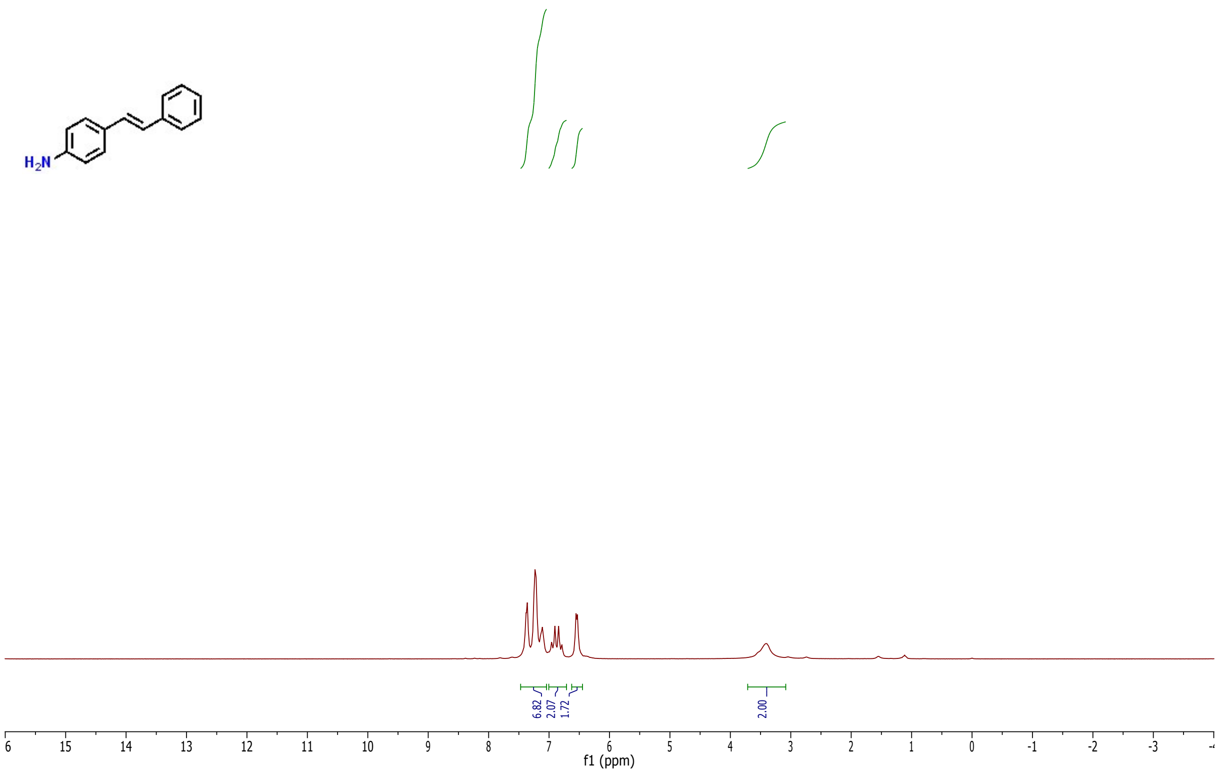

170817.f313.11.fid
Kathir/ KM10-182
C13CPD CDCl3 {C:\Bruker\TopSpin3.5pl6} 1708 13

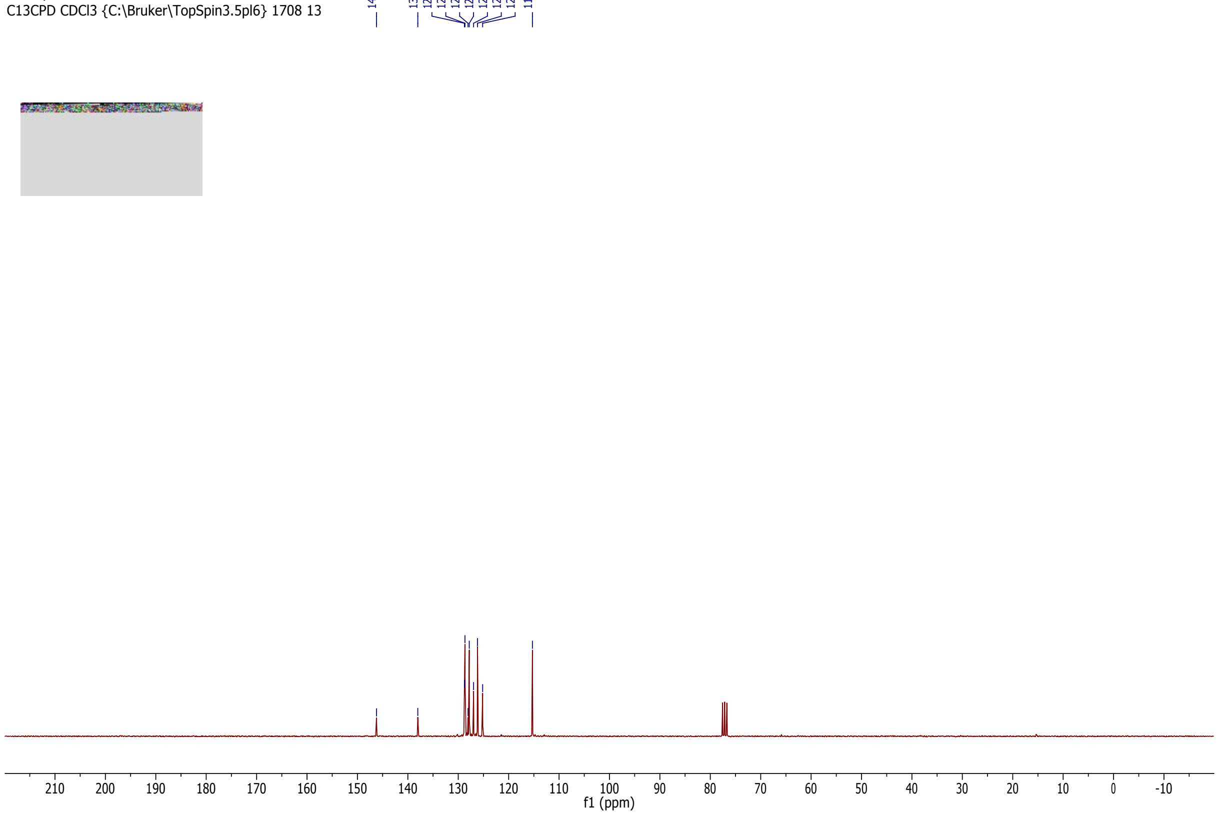

170817.f314.10.fid
Kathir/ KM10-186
PROTON CDCl3 {C:\Bruker\TopSpin3.5pl6} 1708 14

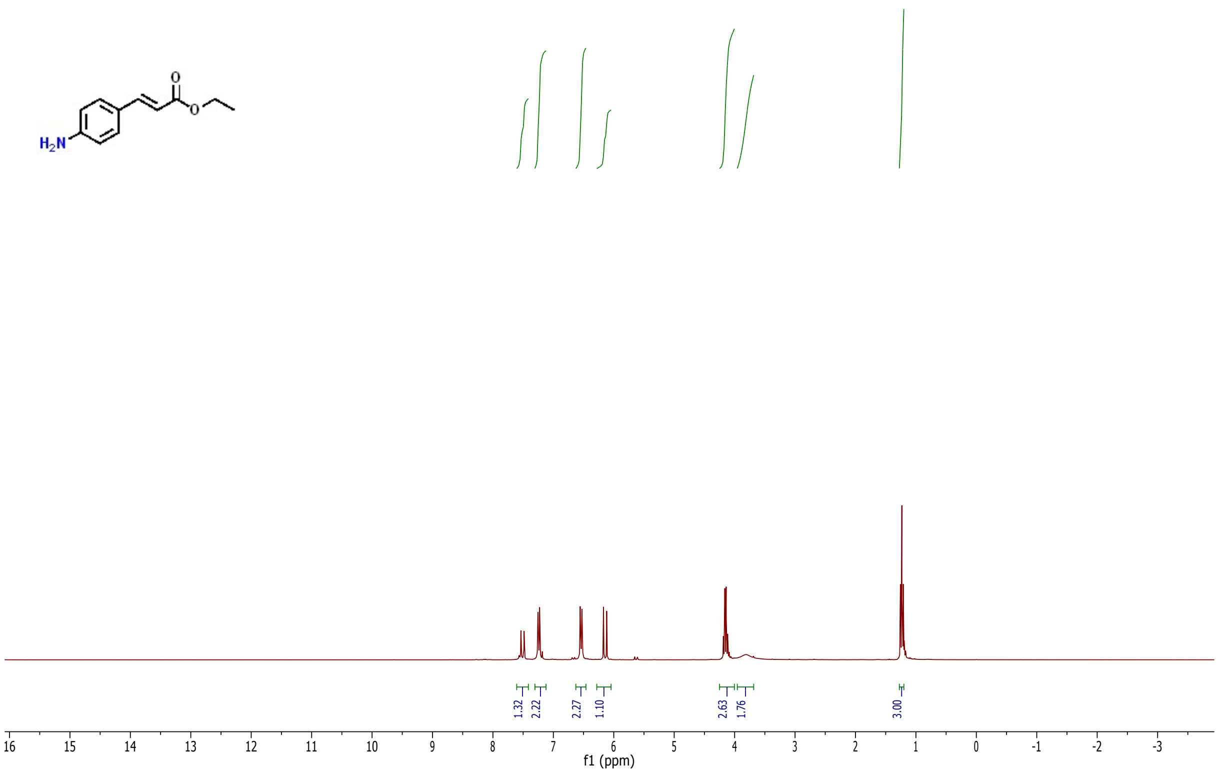

170817.f314.11.fid
Kathir/ KM10-186
C13CPD CDCl3 {C:\Bruker\TopSpin3.5pl6} 1708 14

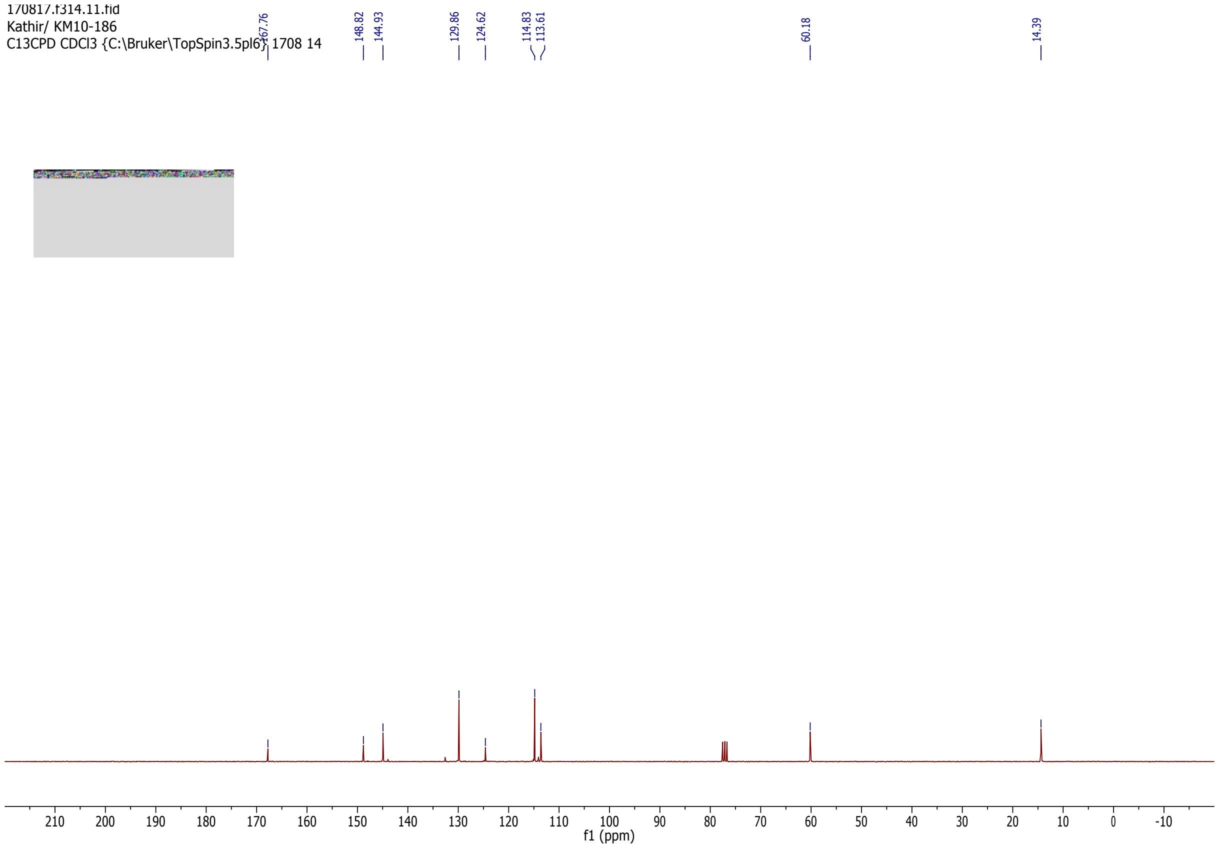

170817.f316.10.fid
Kathir/ KM10-200
PROTON CDCl3 {C:\Bruker\TopSpin3.5pl6} 1708 16

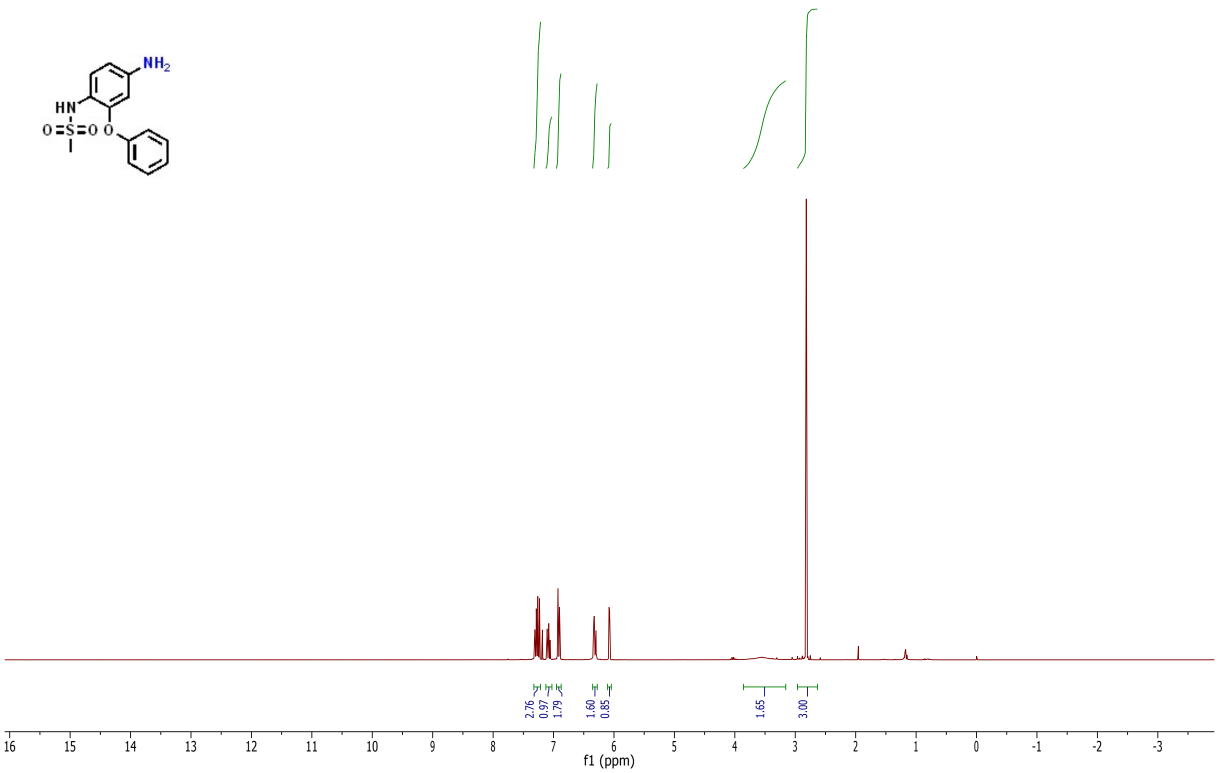

170817.f316.11.fid
Kathir/ KM10-200
C13CPD CDCl3 {C:\Bruker\TopSpin3.5pl6} 1708 16

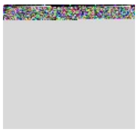

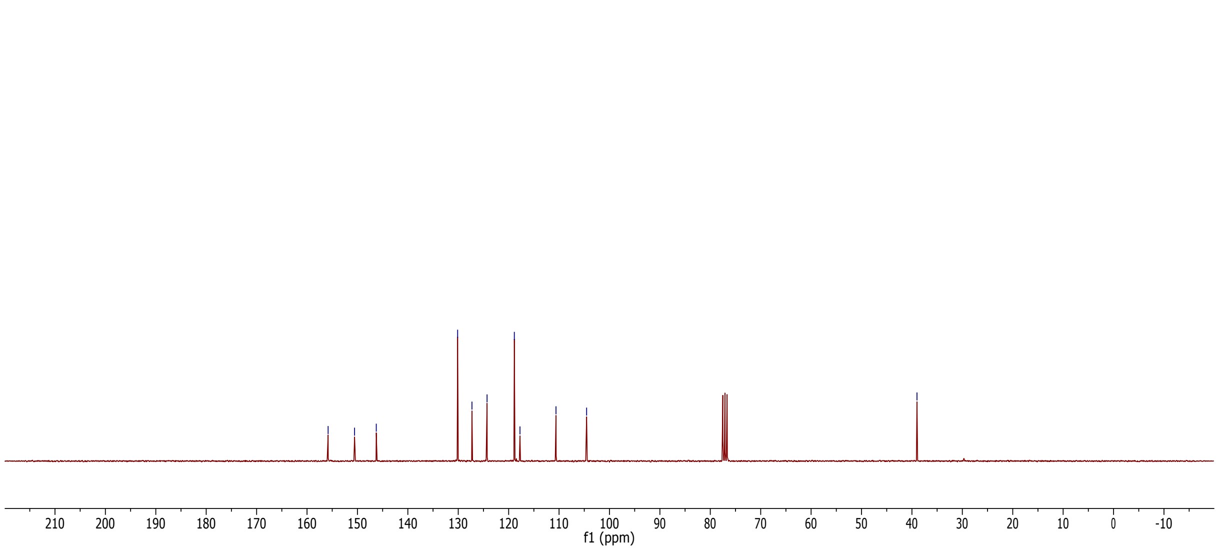

170818.f302.10.fid
Kathir/ KM10-252
PROTON CDCl3 {C:\Bruker\TopSpin3.5pl6} 1708 2

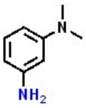

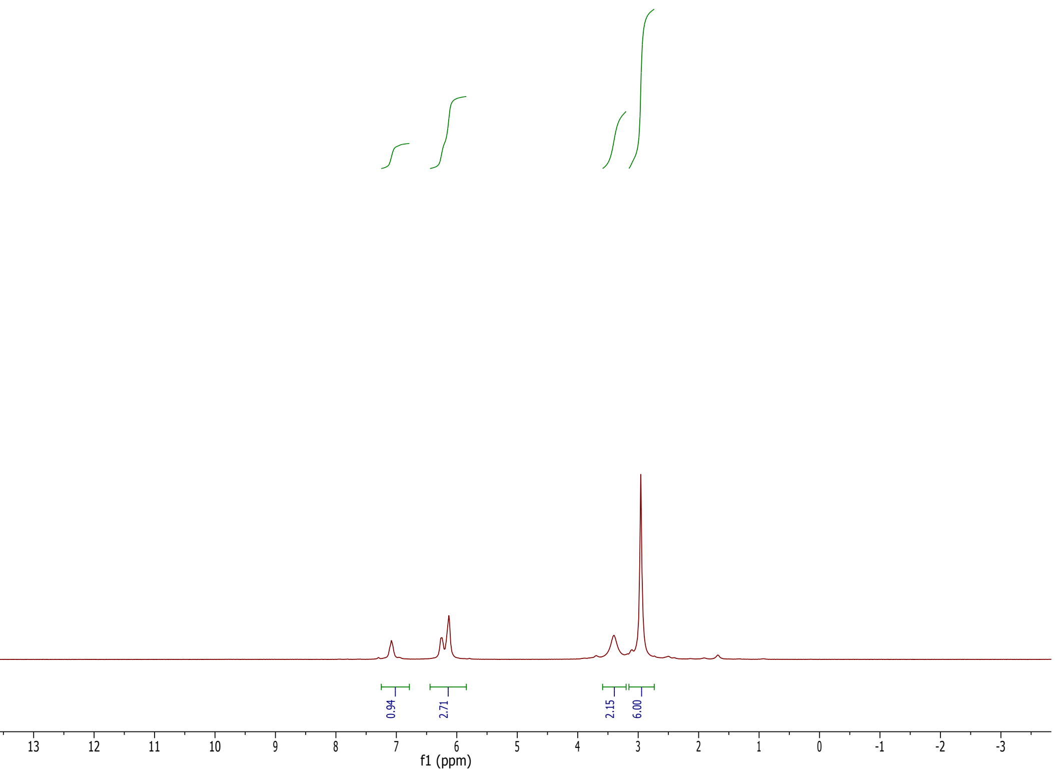

170818.f302.11.fid
Kathir/ KM10-252
C13CPD CDCl3 {C:\Bruker\TopSpin3.5pl6} 1708 2

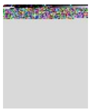

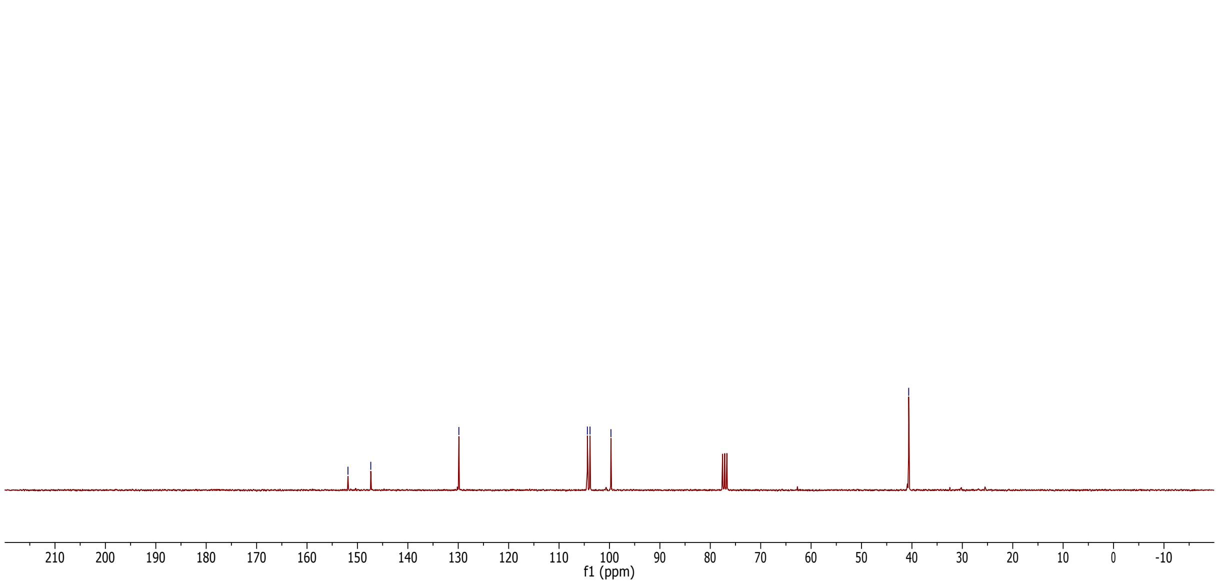

170818.f301.10.fid
Kathir/ KM10-244
PROTON DMSO {C:\Bruker\TopSpin3.5pl6} 1708 1

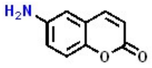

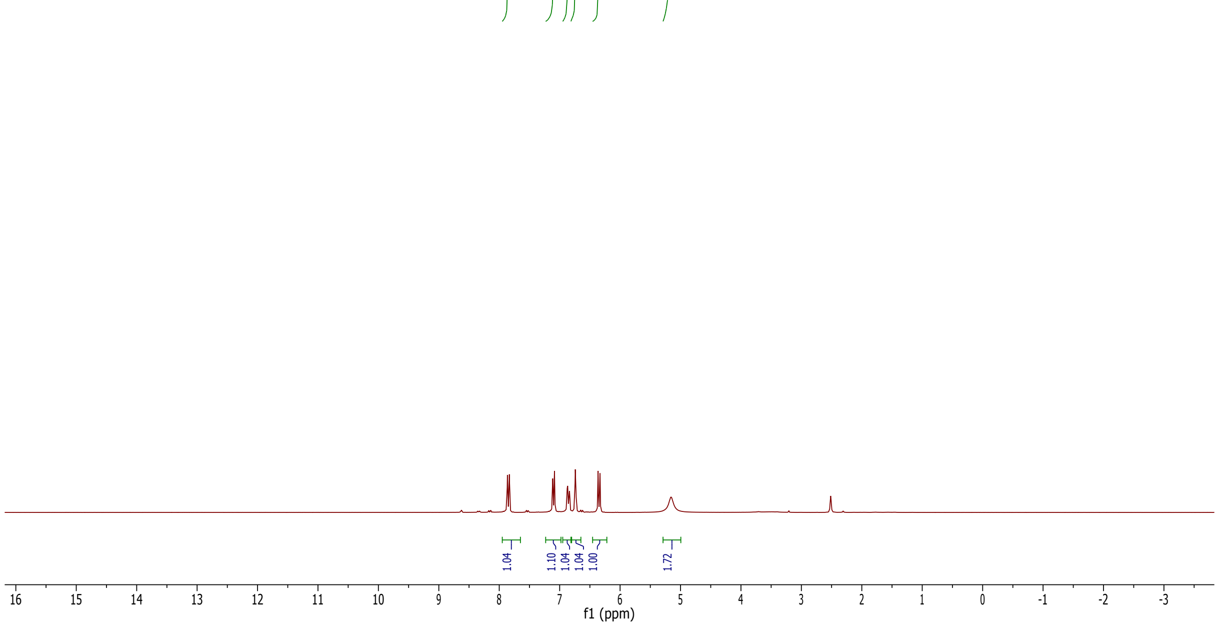

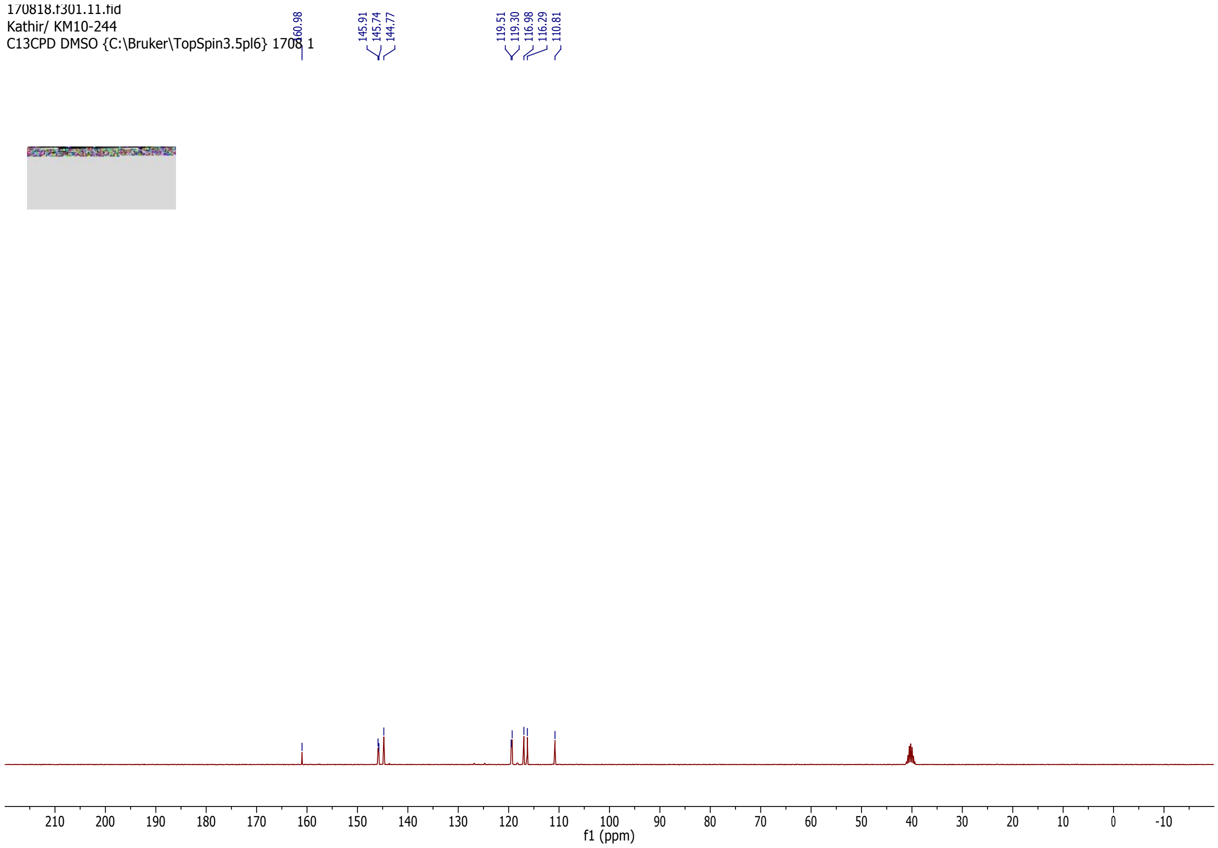
170818.f301.11.fid
Kathir/ KM10-244
C13CPD DMSO {C:\Bruker\TopSpin3.5pl6} 1708 1
160.98
145.91
145.74
144.77
119.51
119.30
116.98
116.29
110.81
210 200 190 180 170 160 150 140 130 120 110 100 90 80 70 60 50 40 30 20 10 0 -10
f1 (ppm)

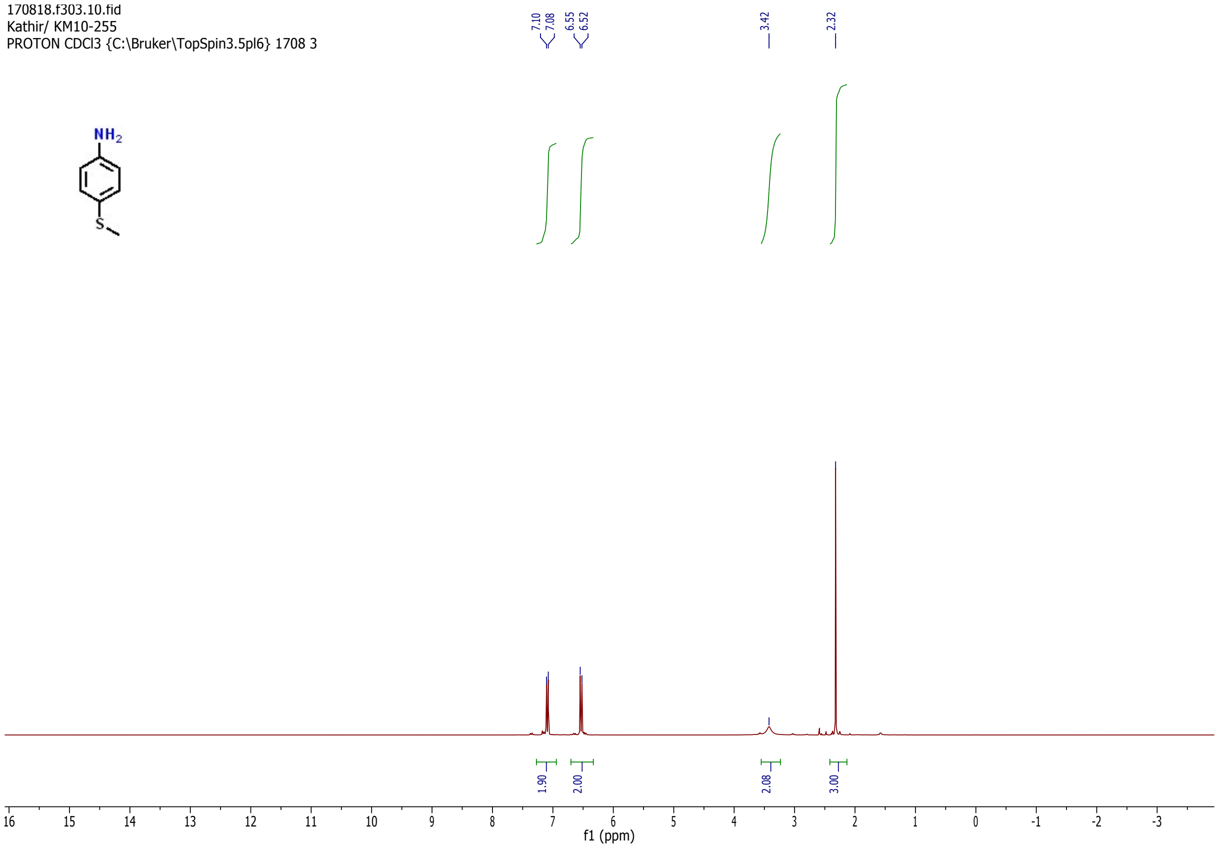
170818.f303.10.fid
Kathir/ KM10-255
PROTON CDCl3 {C:\Bruker\TopSpin3.5pl6} 1708 3
7.10
7.08
6.55
6.52
3.42
2.32
NH2
S
1.90
2.00
2.08
3.00
16 15 14 13 12 11 10 9 8 7 6 5 4 3 2 1 0 -1 -2 -3
f1 (ppm)

170818.f303.11.fid
Kathir/ KM10-255
C13CPD CDCl3 {C:\Bruker\TopSpin3.5pl6} 1708 3

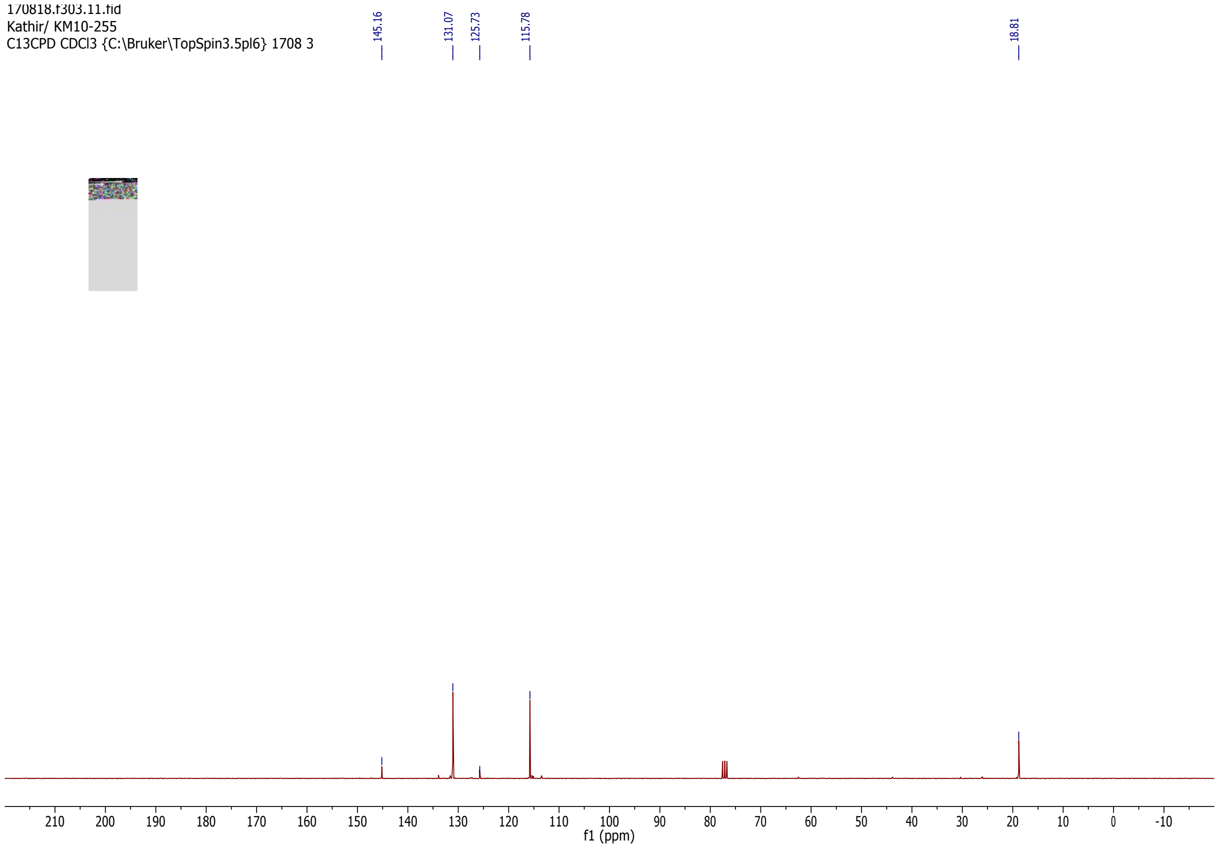

170818.f304.10.fid
Kathir/ KM10-256
PROTON CDCl3 {C:\Bruker\TopSpin3.5pl6} 1708 4

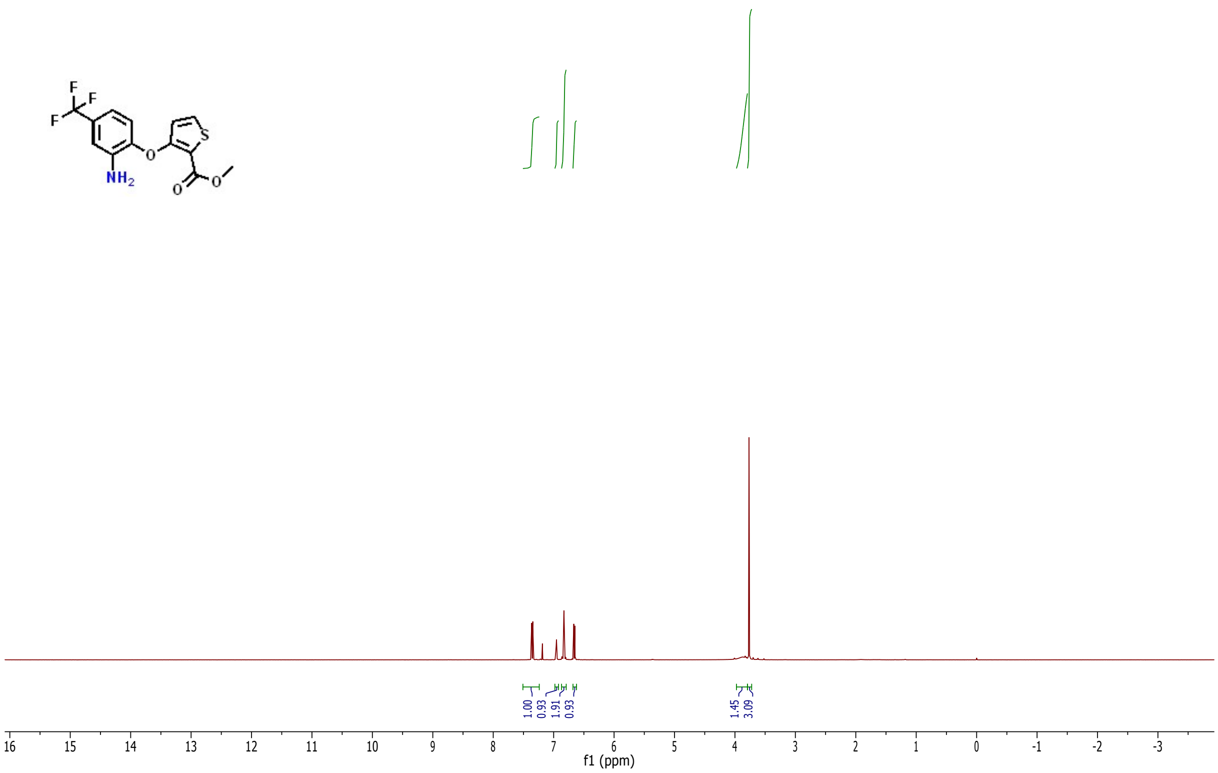

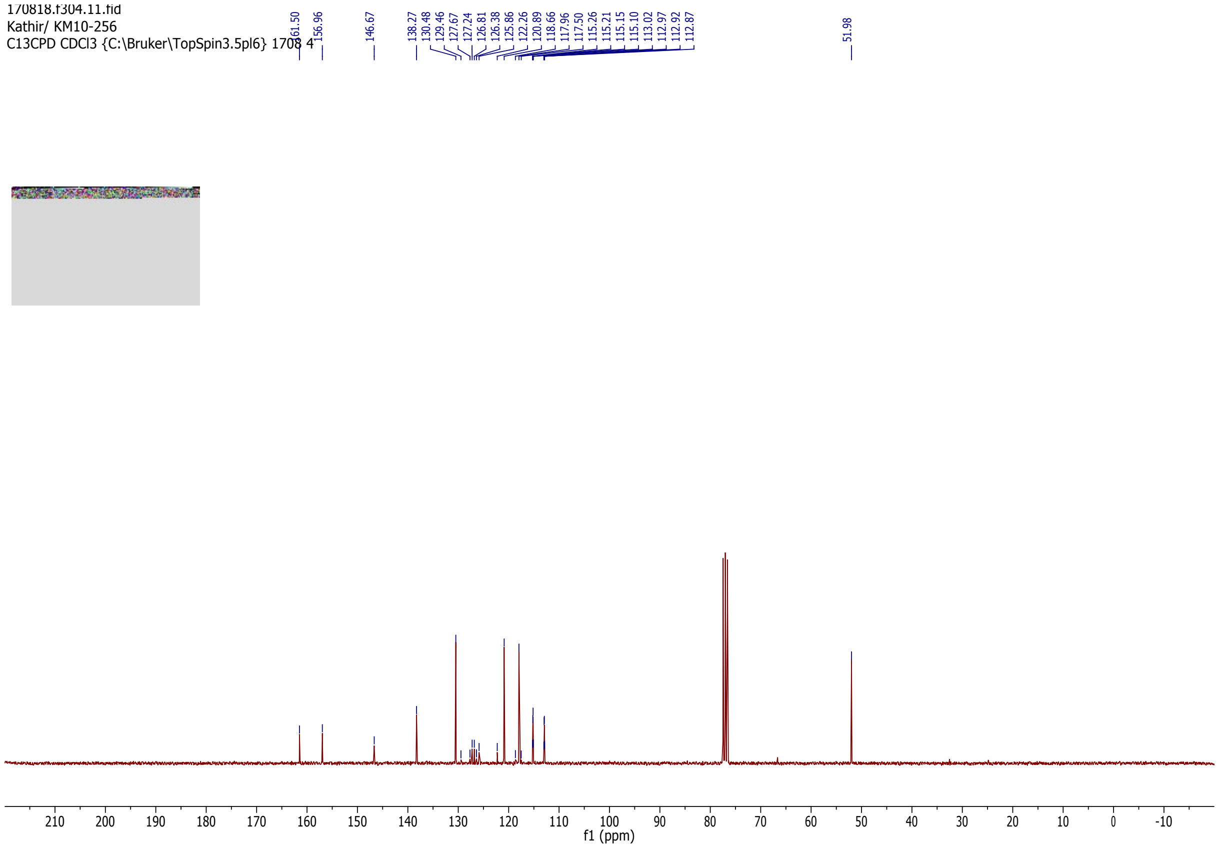
170818.f304.11.fid
Kathir/ KM10-256
C13CPD CDCl3 {C:\Bruker\TopSpin3.5pl6} 1708 4
161.50
156.96
146.67
138.27
130.48
129.46
127.67
127.24
126.81
126.38
125.86
122.26
120.89
118.66
117.96
117.50
115.26
115.21
115.15
115.10
113.02
112.97
112.92
112.87
51.98
f1 (ppm)

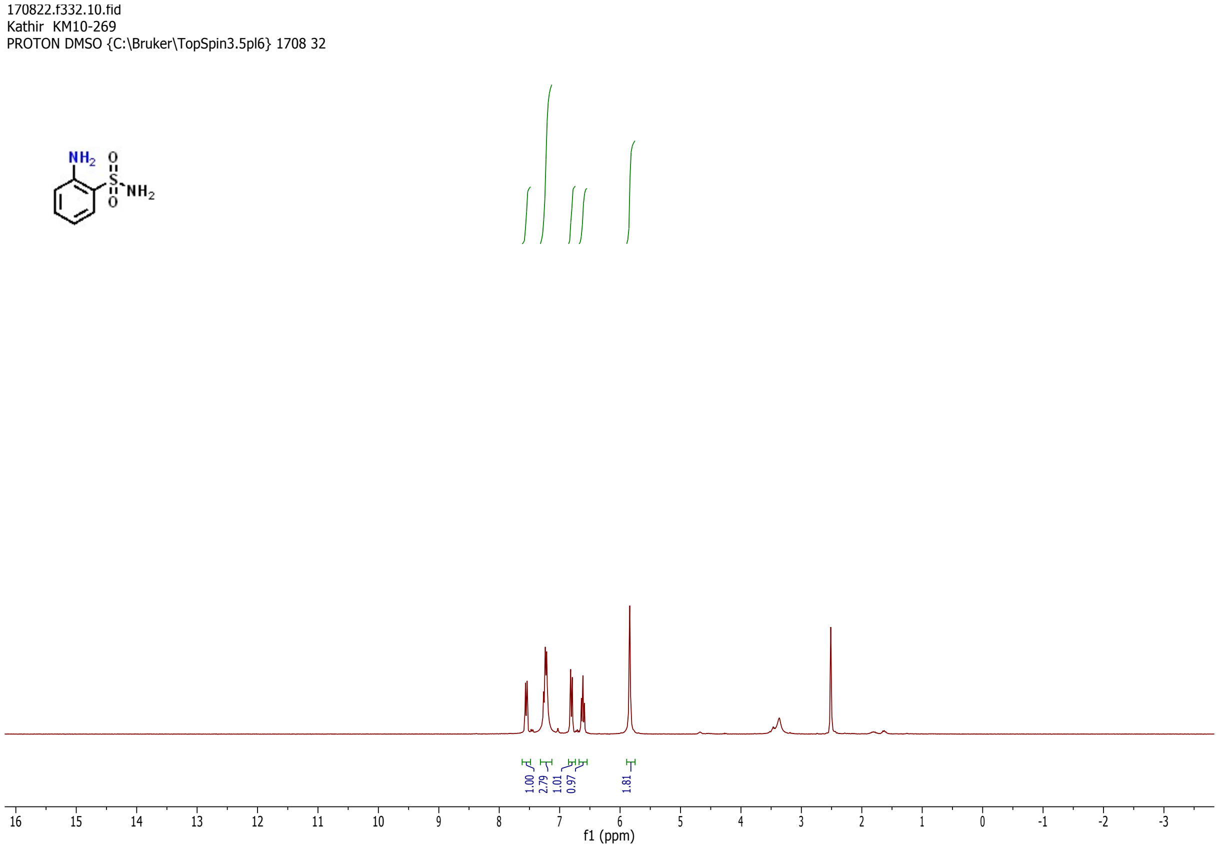
170822.f332.10.fid
Kathir KM10-269
PROTON DMSO {C:\Bruker\TopSpin3.5pl6} 1708 32
NH2
O
S
O
NH2
1.00
2.79
1.01
0.97
1.81
f1 (ppm)

170822.f332.11.fid
Kathir KM10-269
C13CPD DMSO {C:\Bruker\TopSpin3.5pl6} 1708 32

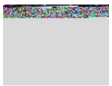

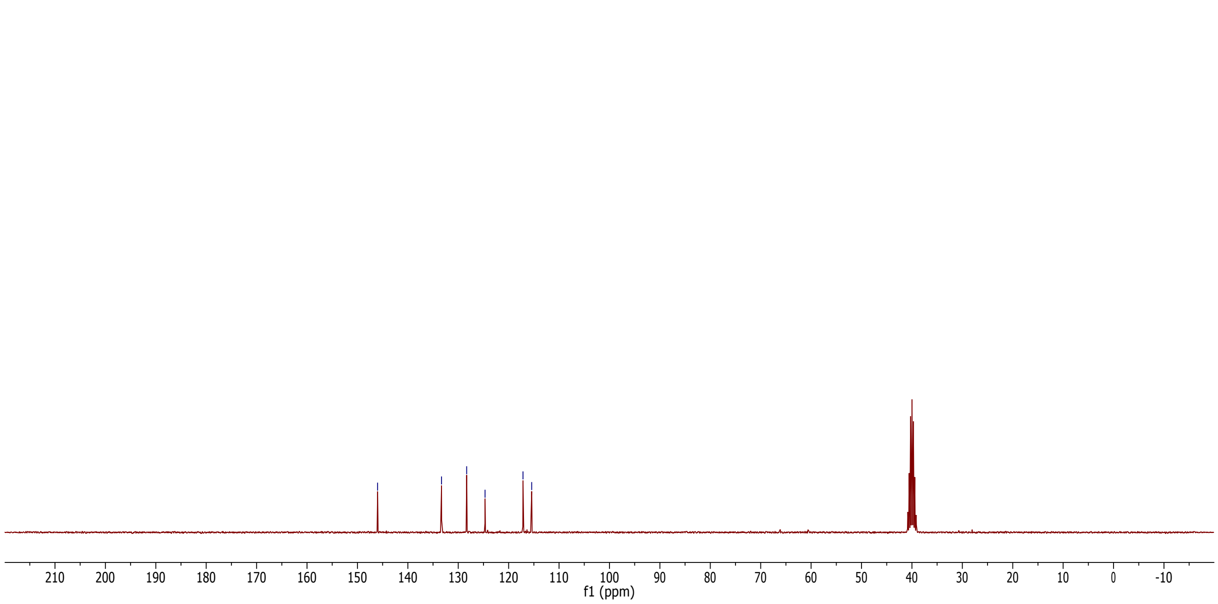

170822.f334.10.fid
Kathir KM10-267
PROTON CDCl3 {C:\Bruker\TopSpin3.5pl6} 1708 34

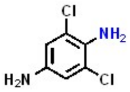

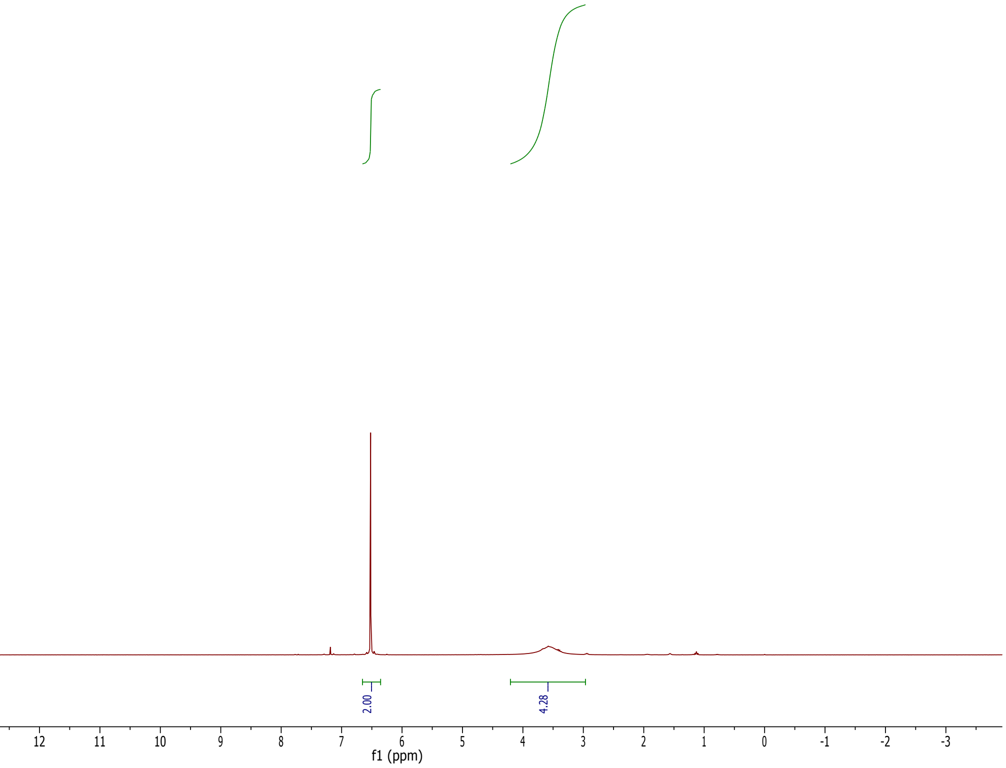

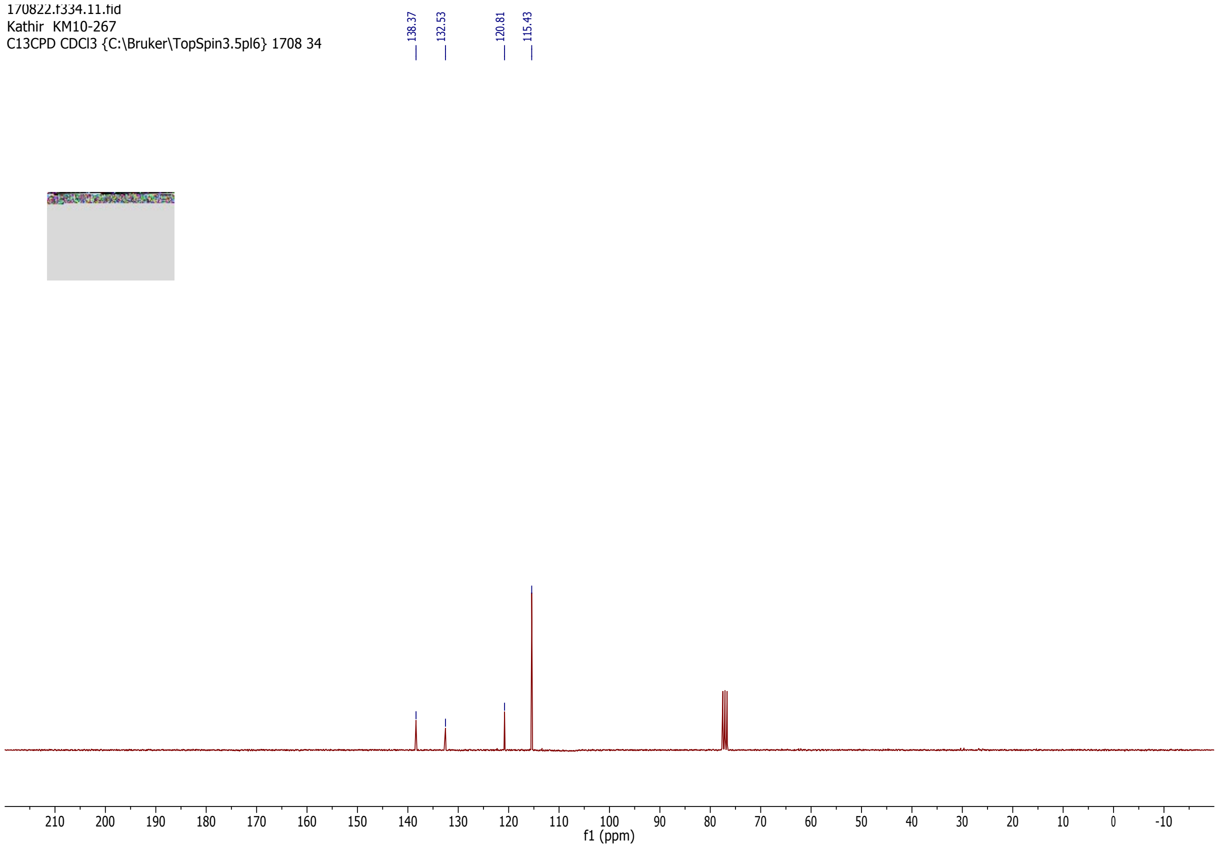
170822.f334.11.fid
Kathir KM10-267
C13CPD CDCl3 {C:\Bruker\TopSpin3.5pl6} 1708 34
138.37
132.53
120.81
115.43
f1 (ppm)

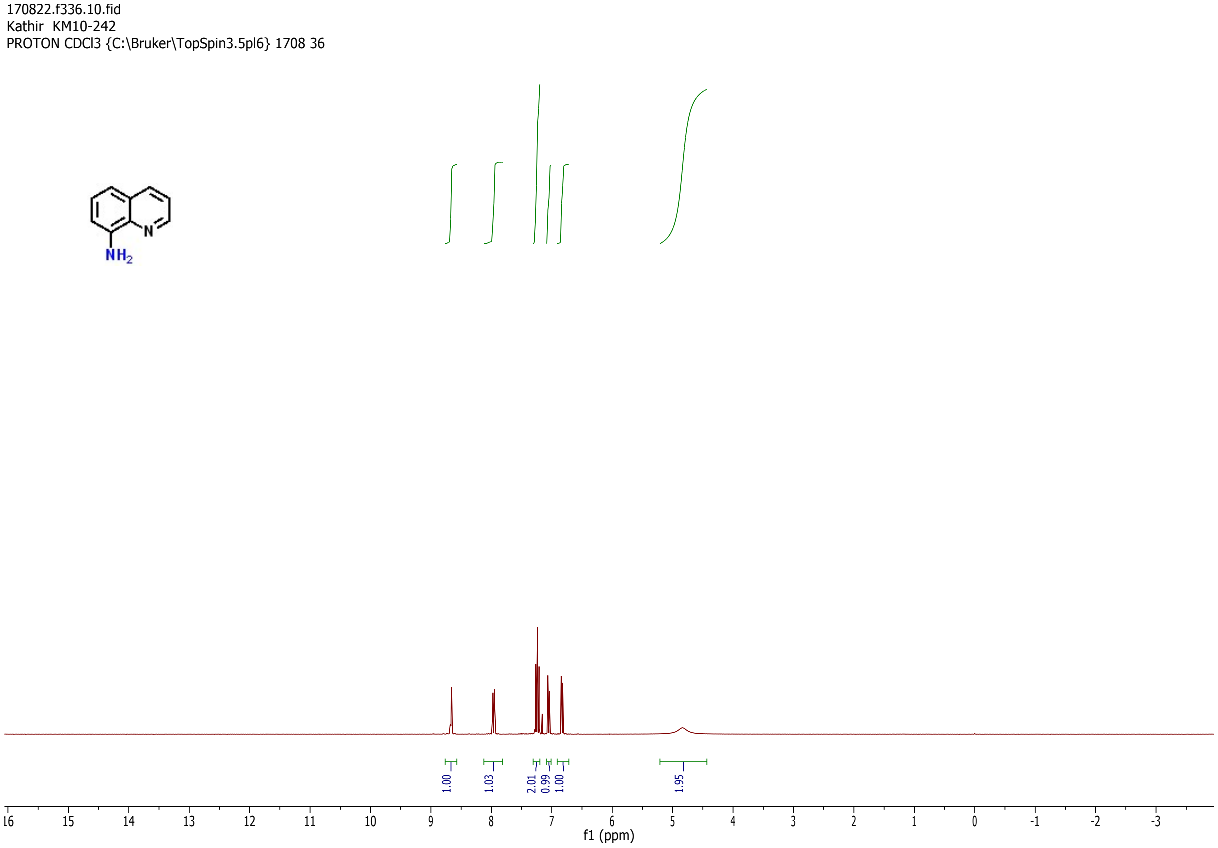
170822.f336.10.fid
Kathir KM10-242
PROTON CDCl3 {C:\Bruker\TopSpin3.5pl6} 1708 36
N
NH2
1.00
1.03
2.01
0.99
1.00
1.95
f1 (ppm)

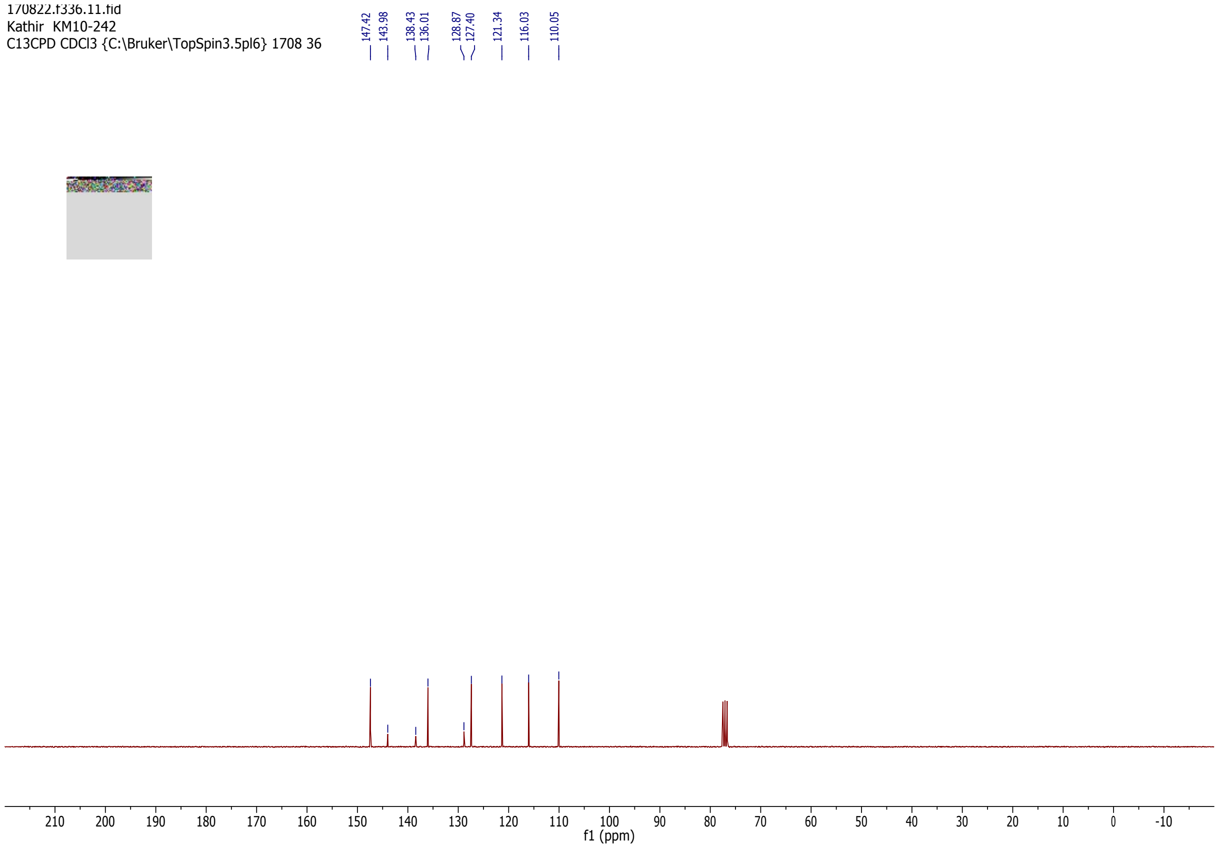
170822.f336.11.fid
Kathir KM10-242
C13CPD CDCl3 {C:\Bruker\TopSpin3.5pl6} 1708 36
147.42
143.98
138.43
136.01
128.87
127.40
121.34
116.03
110.05
f1 (ppm)

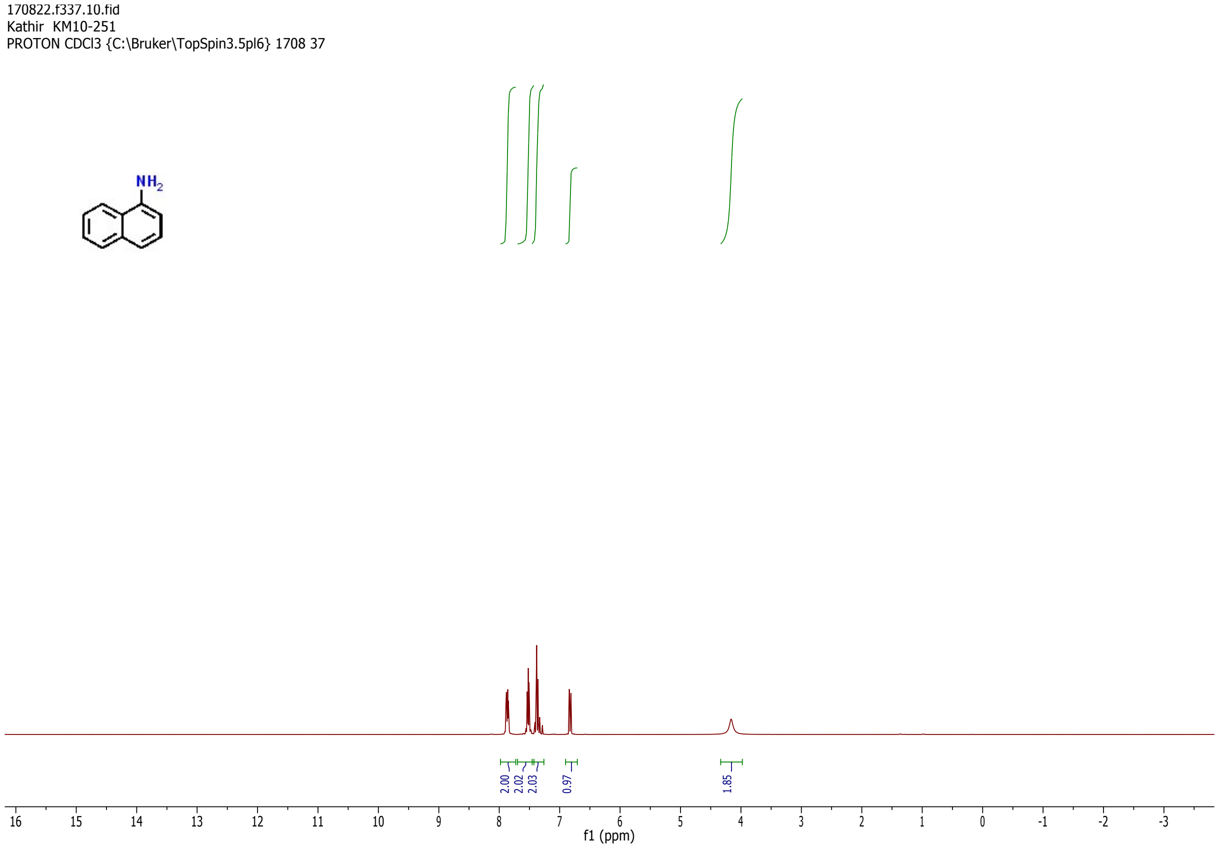
170822.f337.10.fid
Kathir KM10-251
PROTON CDCl3 {C:\Bruker\TopSpin3.5pl6} 1708 37
NH2
2.00
2.02
2.03
0.97
1.85
f1 (ppm)

170822.f337.11.fid
Kathir KM10-251
C13CPD CDCl3 {C:\Bruker\TopSpin3.5pl6} 1708 37

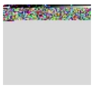

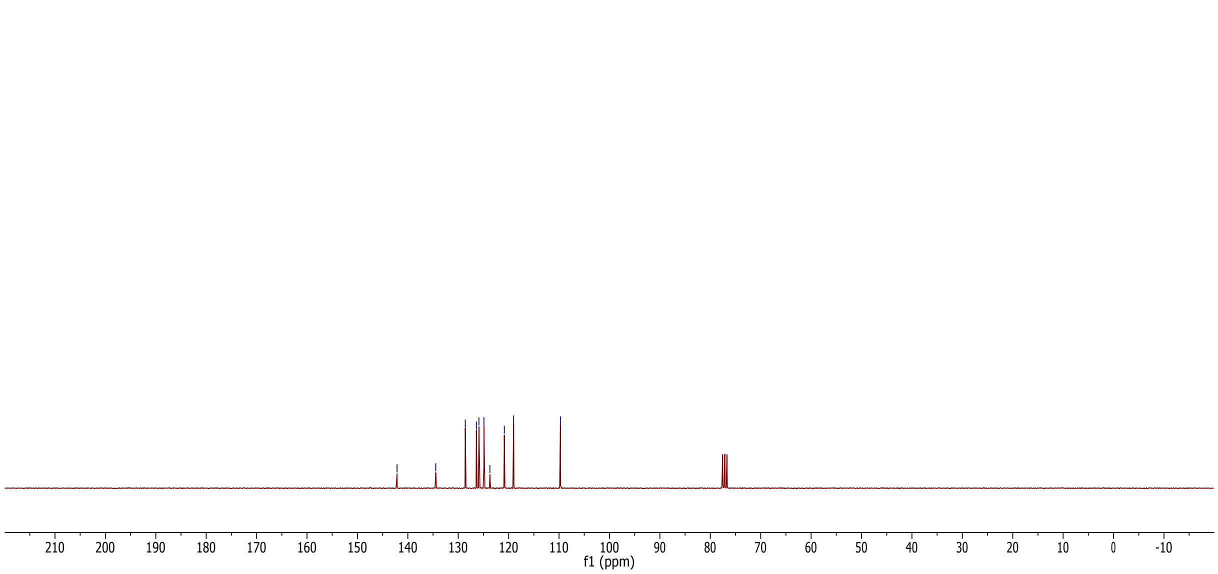

170822.f338.10.fid
Kathir KM10-277
PROTON CDCl3 {C:\Bruker\TopSpin3.5pl6} 1708 38

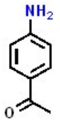

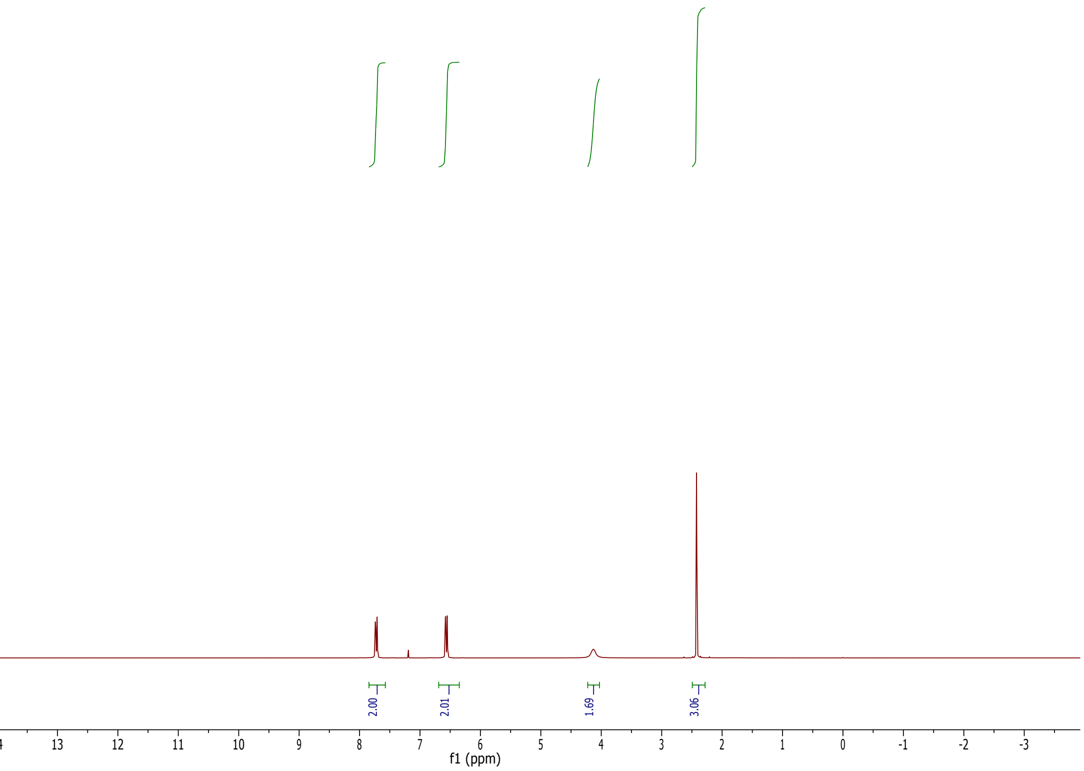

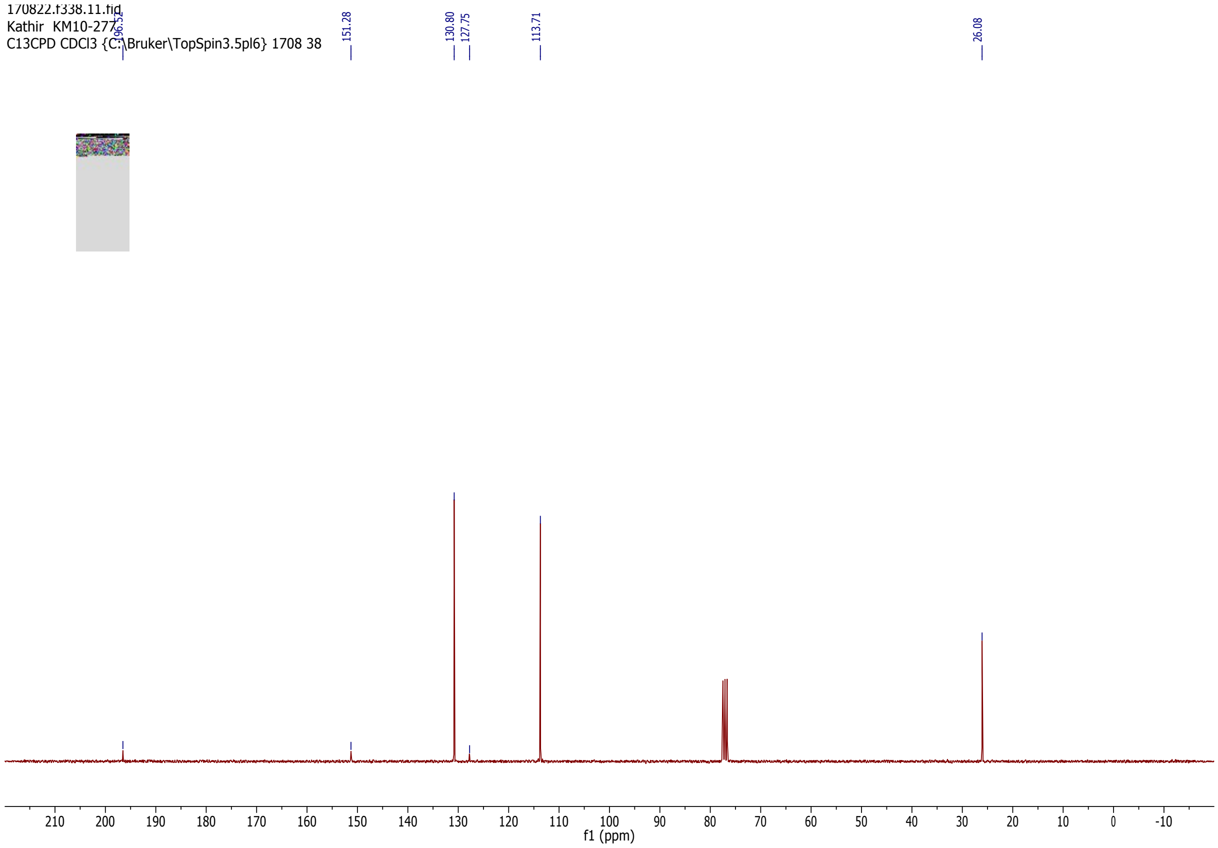
170822.f338.11.fid
Kathir KM10-277
C13CPD CDCl3 {C:\Bruker\TopSpin3.5pl6} 1708 38
196.52
151.28
130.80
127.75
113.71
26.08
210 200 190 180 170 160 150 140 130 120 110 100 90 80 70 60 50 40 30 20 10 0 -10
f1 (ppm)

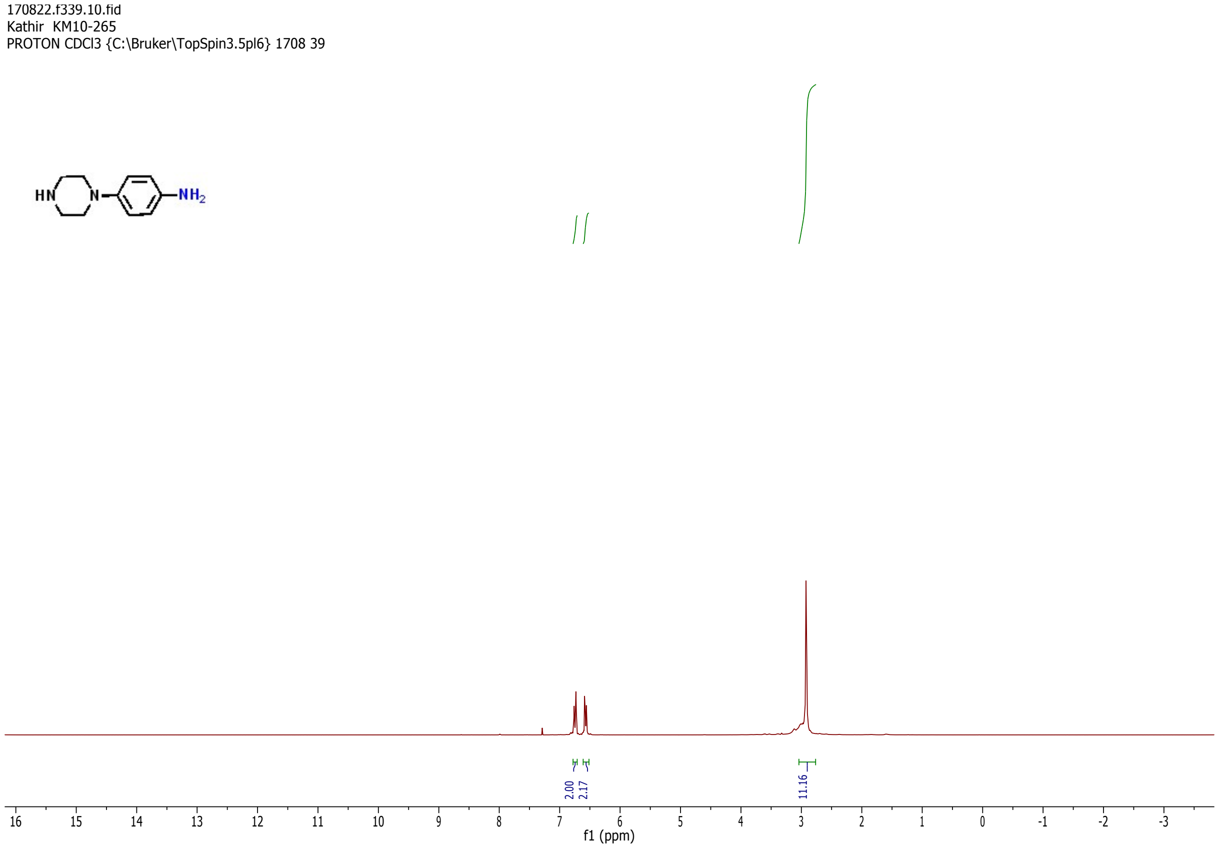
170822.f339.10.fid
Kathir KM10-265
PROTON CDCl3 {C:\Bruker\TopSpin3.5pl6} 1708 39
HN
N
NH2
2.00
2.17
11.16
16 15 14 13 12 11 10 9 8 7 6 5 4 3 2 1 0 -1 -2 -3
f1 (ppm)

170822.f339.11.fid
Kathir KM10-265
C13CPD CDCl3 {C:\Bruker\TopSpin3.5pl6} 1708 39

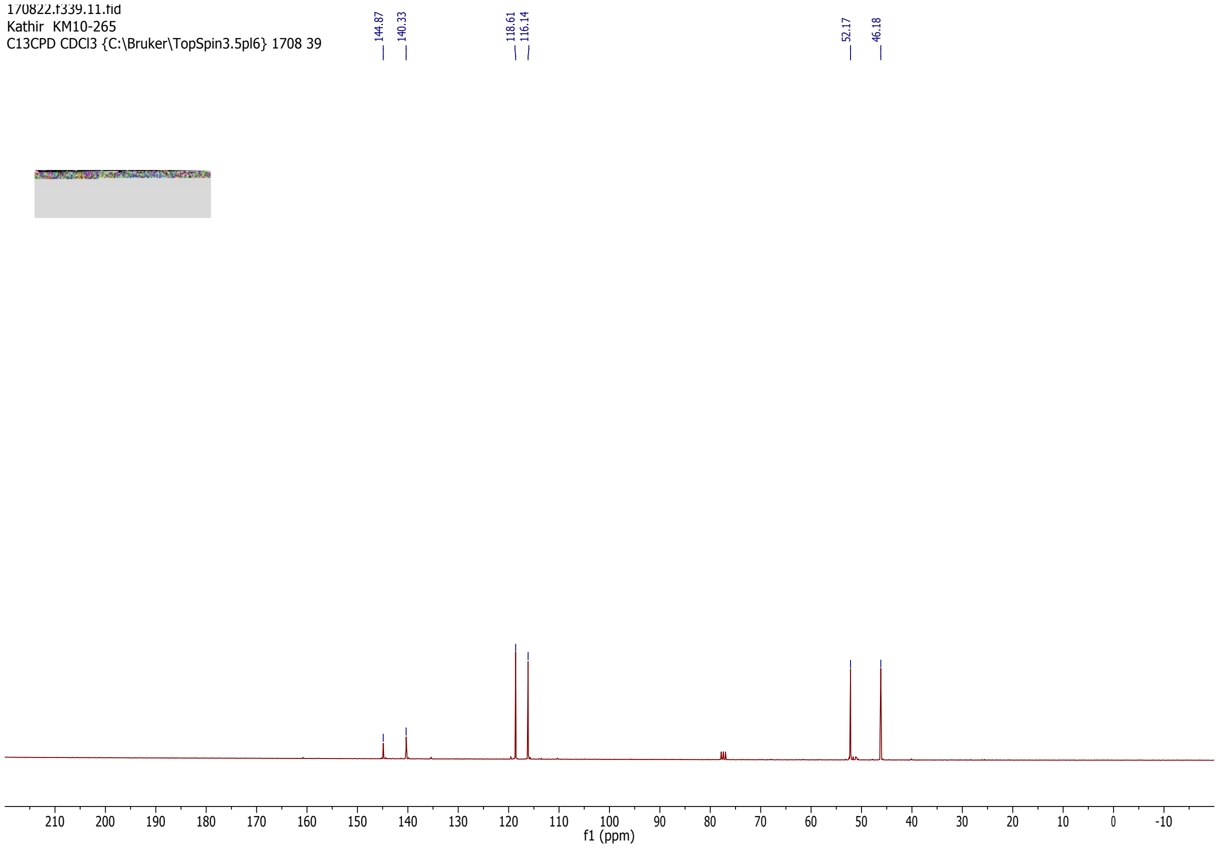

170822.f340.10.fid
Kathir KM10-268
PROTON CDCl3 {C:\Bruker\TopSpin3.5pl6} 1708 40

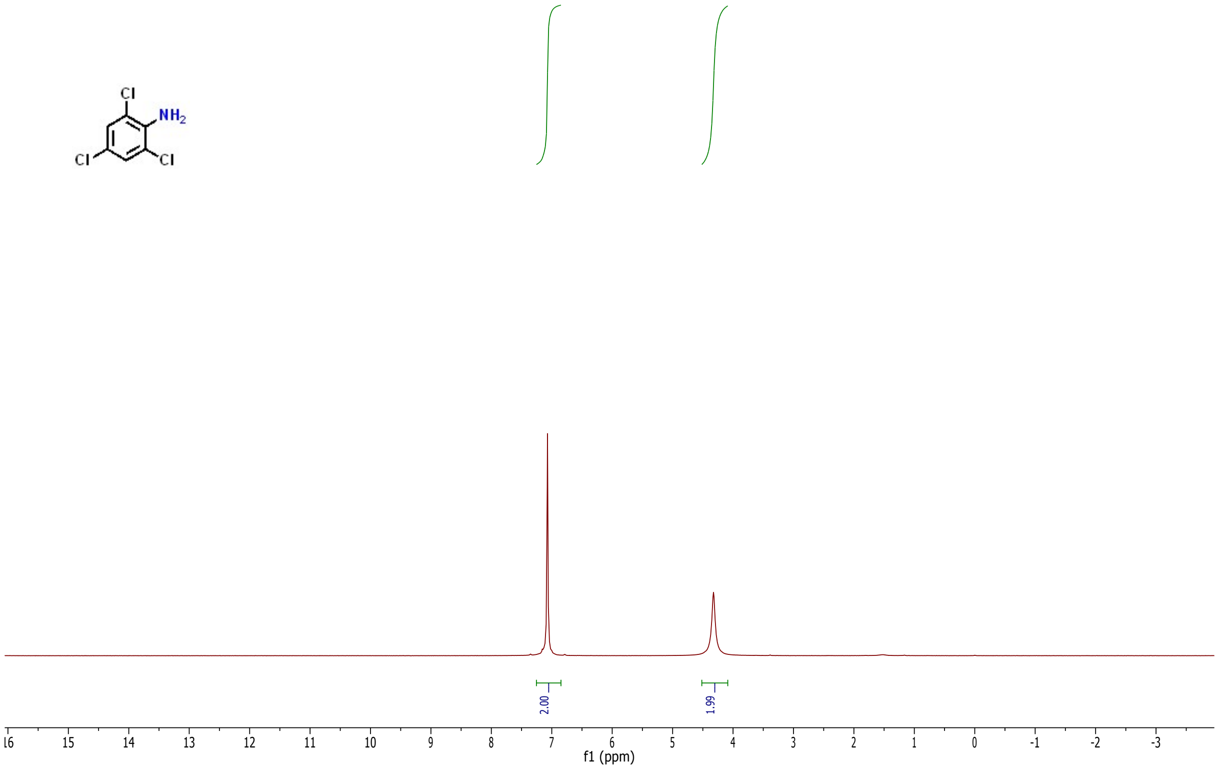

170822.f340.11.fid
Kathir KM10-268
C13CPD CDCl3 {C:\Bruker\TopSpin3.5pl6} 1708 40

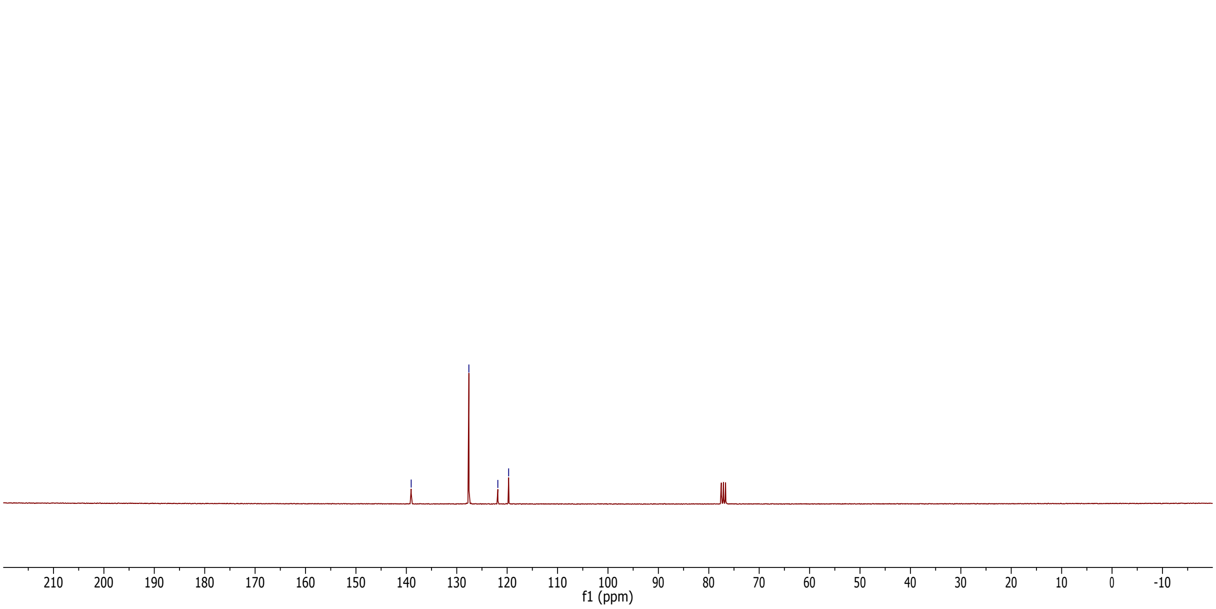

170823.f311.10.fid
Kathir KM10-272
PROTON CDCl3 {C:\Bruker\TopSpin3.5pl6} 1708 11

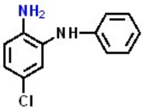

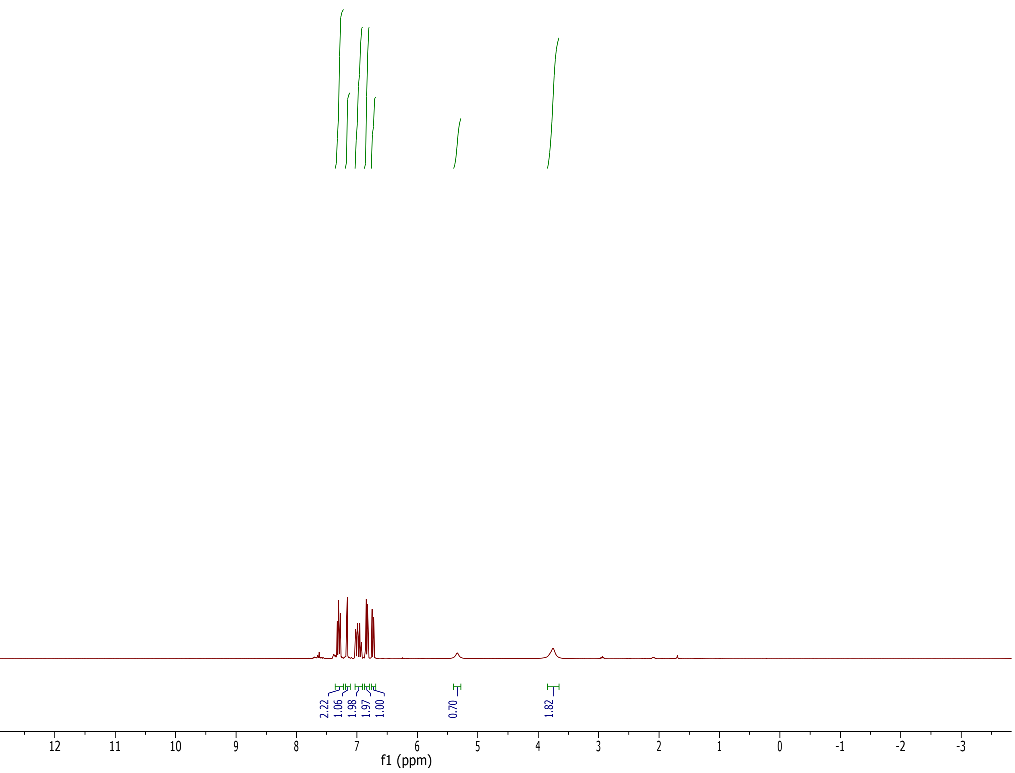

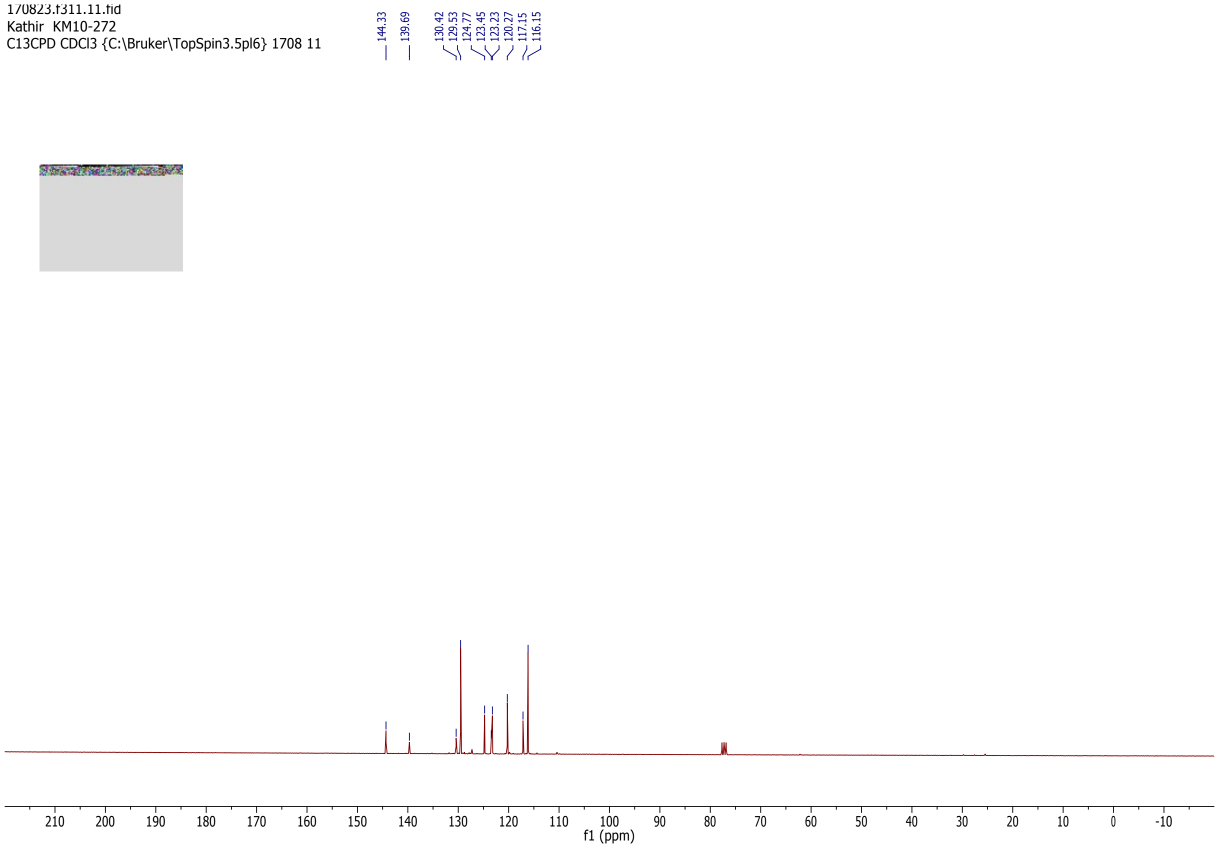
170823.f311.11.fid
Kathir KM10-272
C13CPD CDCl3 {C:\Bruker\TopSpin3.5pl6} 1708 11
144.33
139.69
130.42
129.53
124.77
123.45
123.23
120.27
117.15
116.15
210 200 190 180 170 160 150 140 130 120 110 100 90 80 70 60 50 40 30 20 10 0 -10
f1 (ppm)

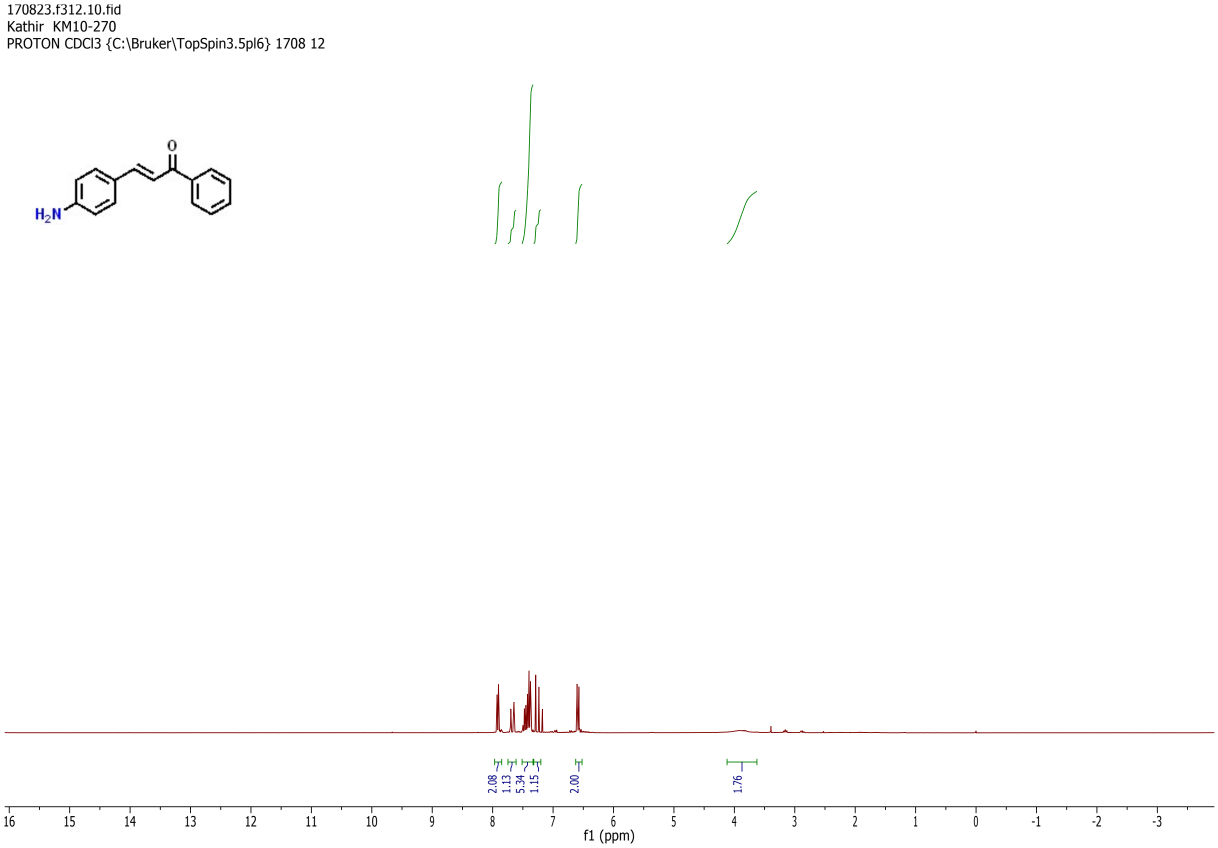
170823.f312.10.fid
Kathir KM10-270
PROTON CDCl3 {C:\Bruker\TopSpin3.5pl6} 1708 12
O
H2N
2.08
1.13
5.34
1.15
2.00
1.76
16 15 14 13 12 11 10 9 8 7 6 5 4 3 2 1 0 -1 -2 -3
f1 (ppm)

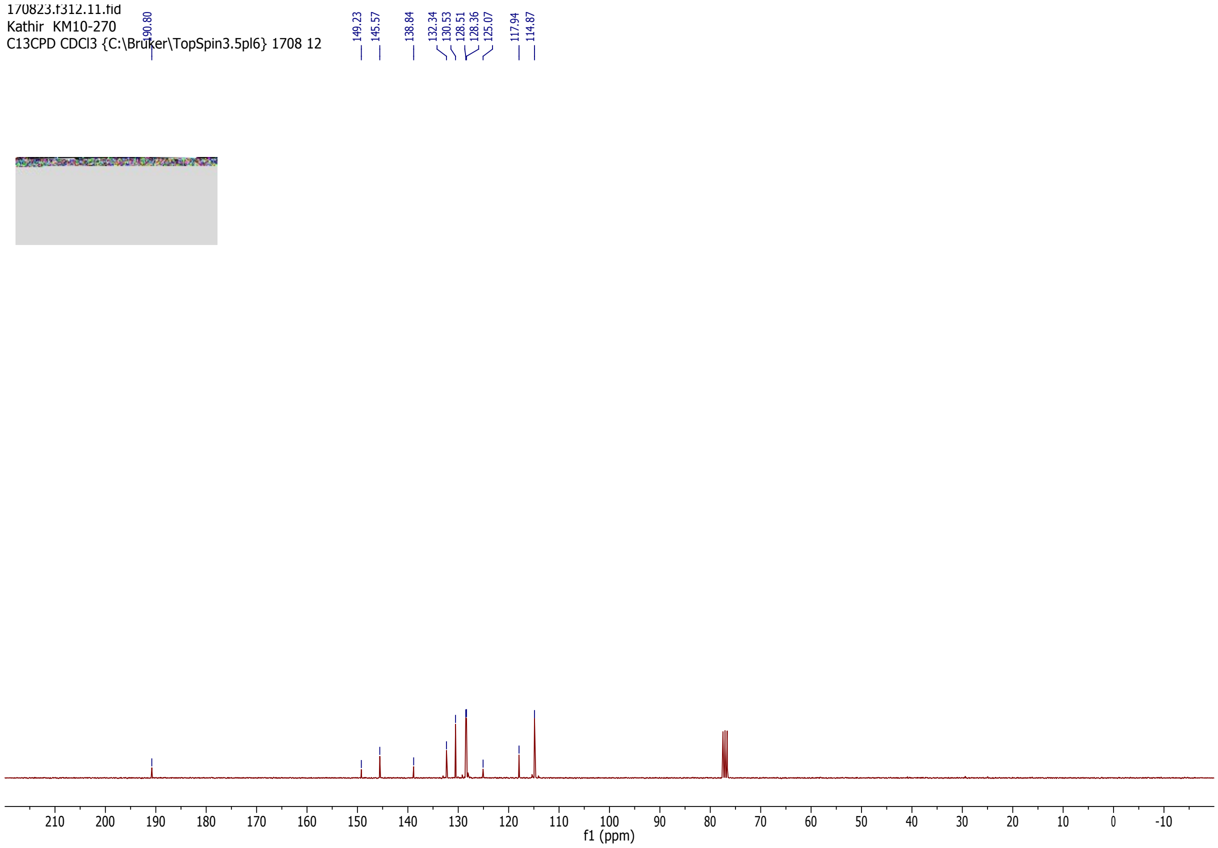
170823.f312.11.fid
Kathir KM10-270
C13CPD CDCl3 {C:\Bruker\TopSpin3.5pl6} 1708 12
190.80
149.23
145.57
138.84
132.34
130.53
128.51
128.36
125.07
117.94
114.87
f1 (ppm)

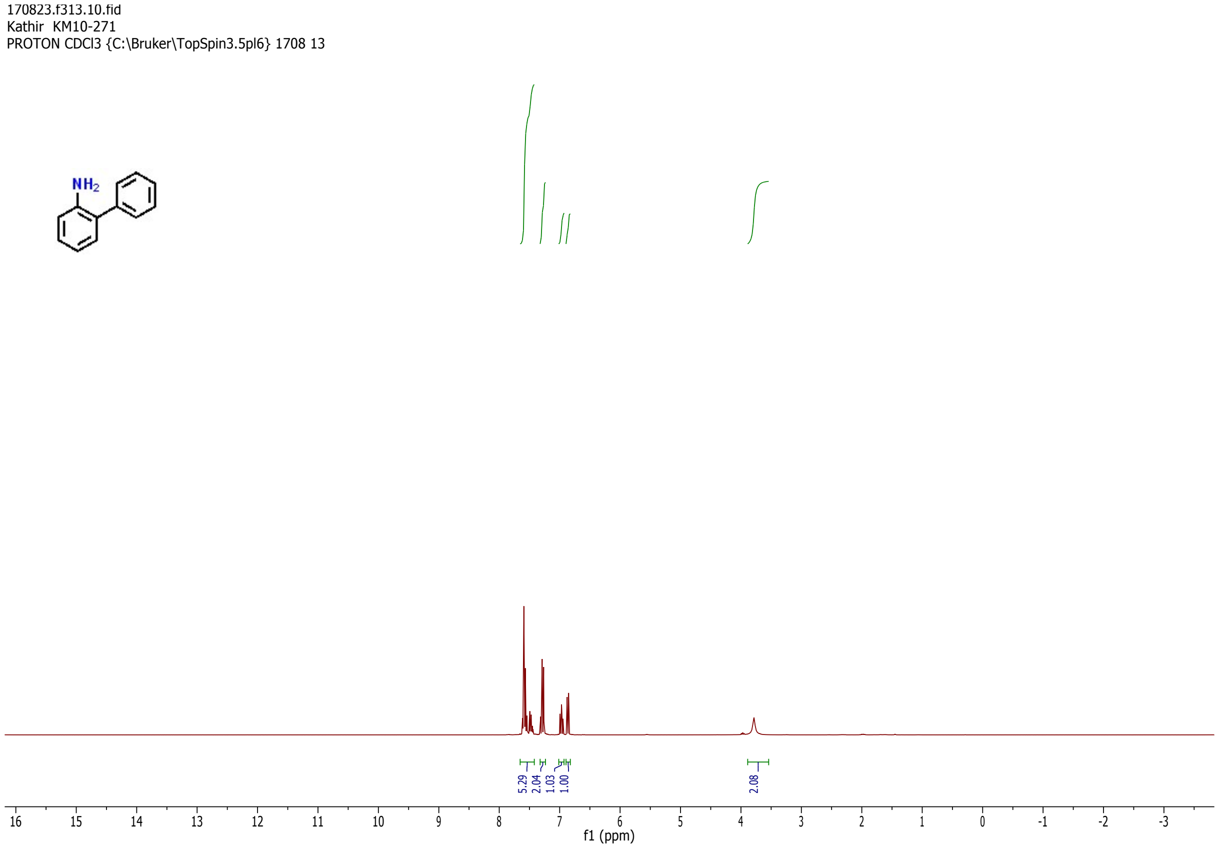
170823.f313.10.fid
Kathir KM10-271
PROTON CDCl3 {C:\Bruker\TopSpin3.5pl6} 1708 13
NH2
5.29
2.04
1.03
1.00
2.08
f1 (ppm)

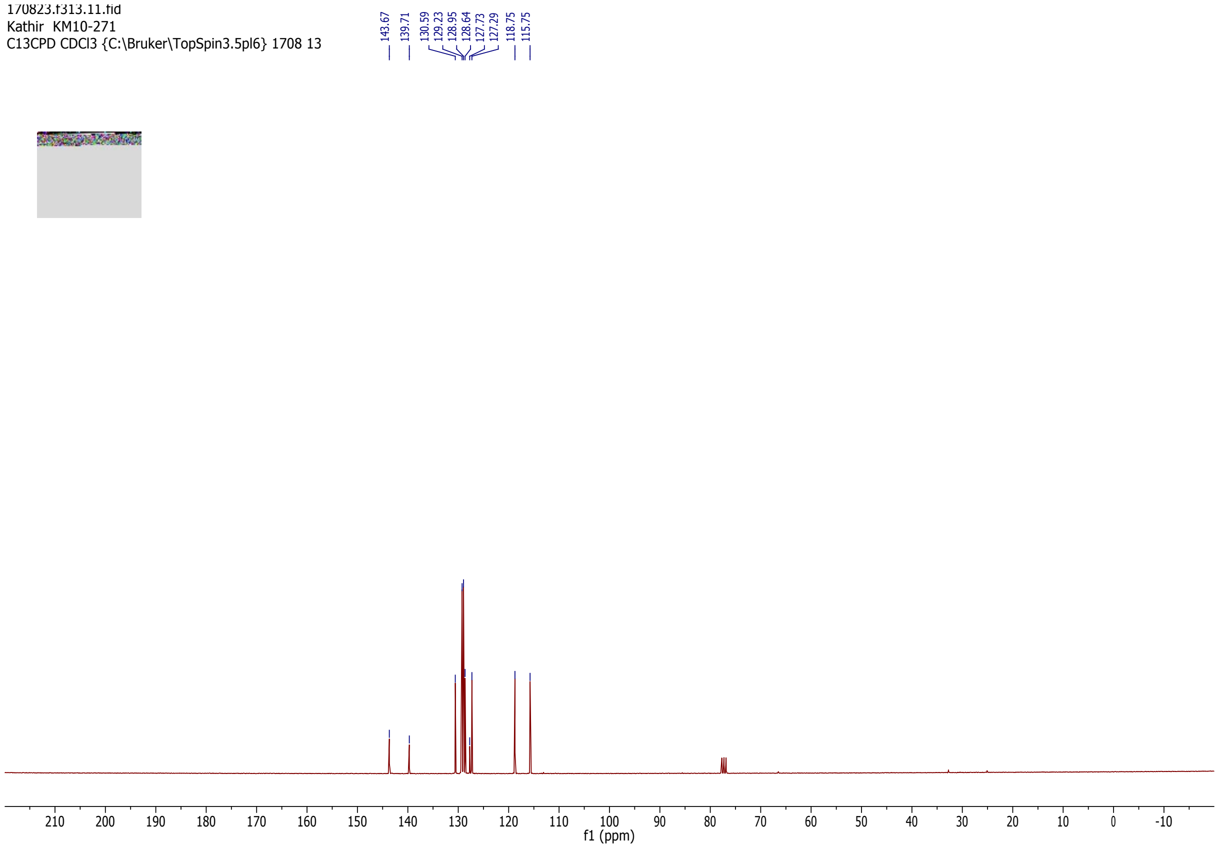
170823.f313.11.fid
Kathir KM10-271
C13CPD CDCl3 {C:\Bruker\TopSpin3.5pl6} 1708 13
143.67
139.71
130.59
129.23
128.95
128.64
127.73
127.29
118.75
115.75
f1 (ppm)

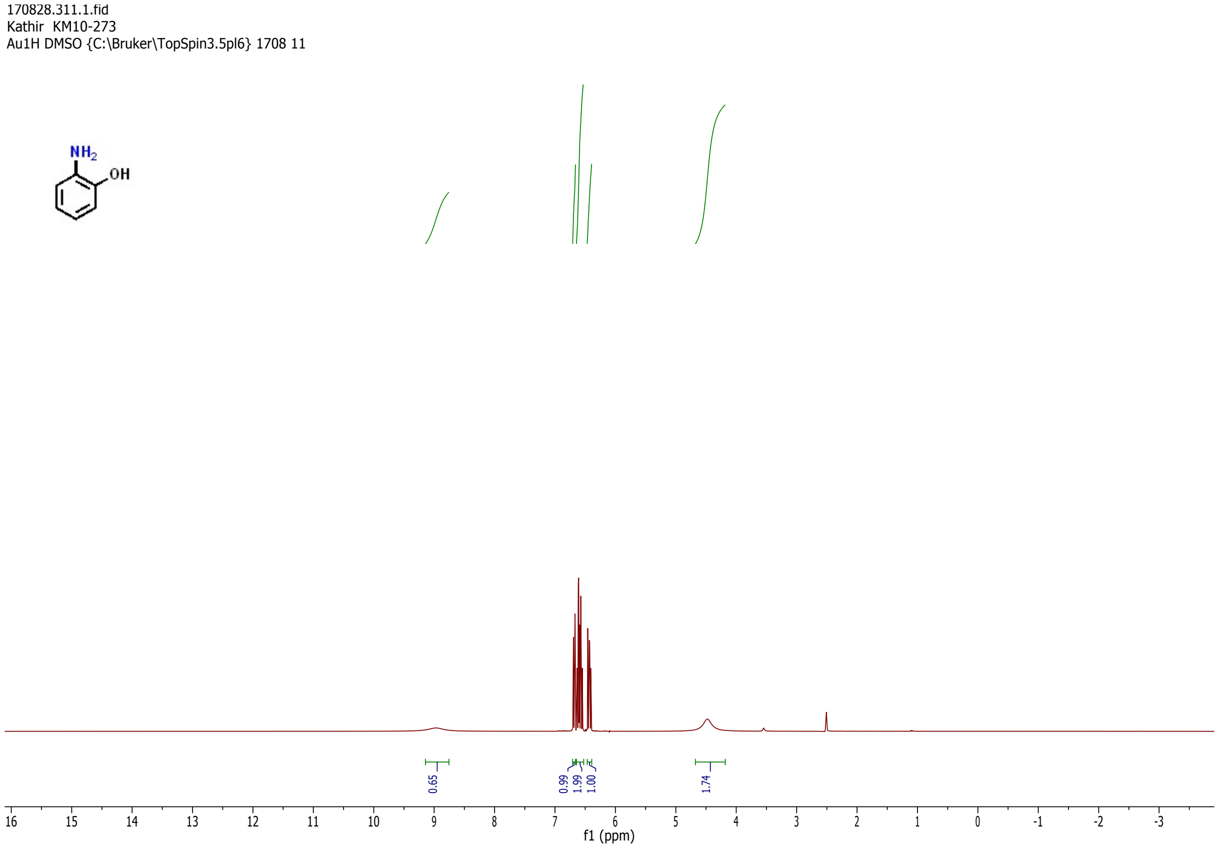
170828.311.1.fid
Kathir KM10-273
Au1H DMSO {C:\Bruker\TopSpin3.5pl6} 1708 11
NH2
OH
0.65
0.99
1.99
1.00
1.74
f1 (ppm)

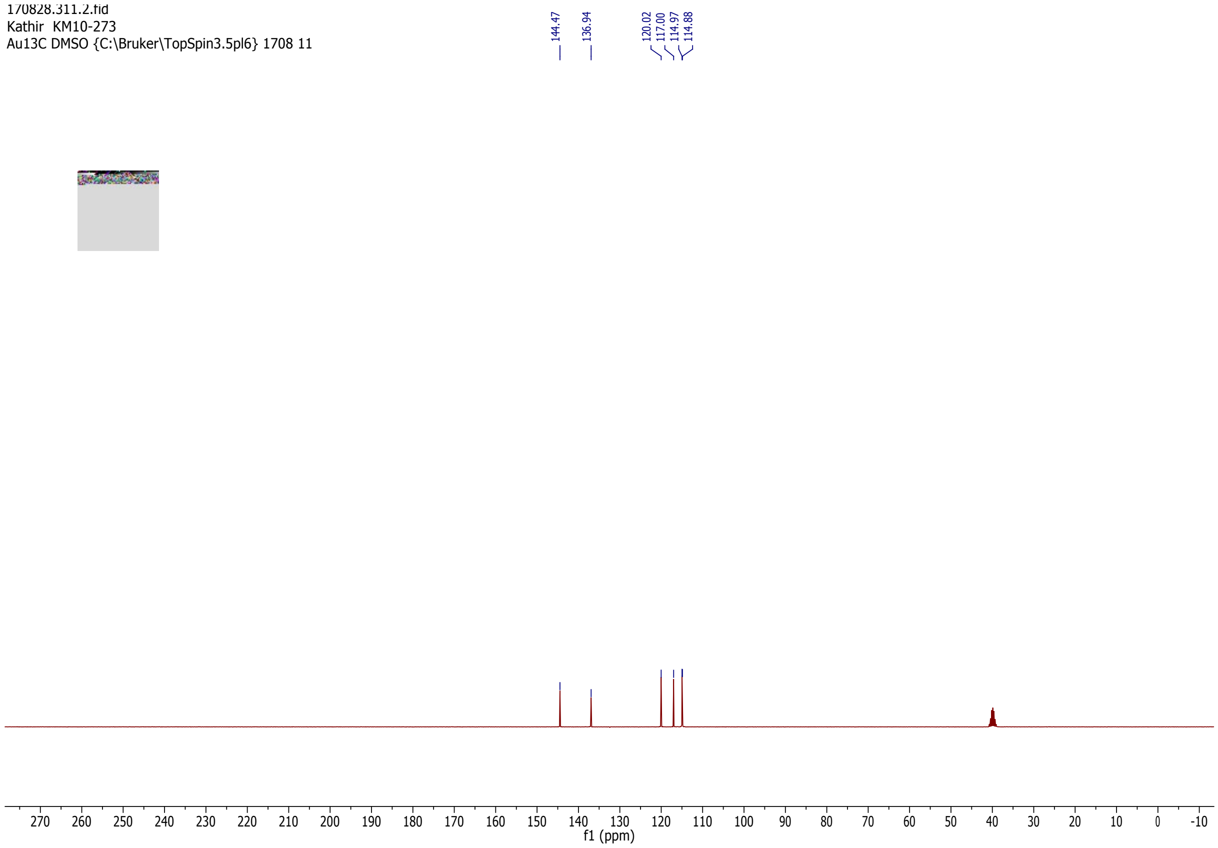
170828.311.2.fid
Kathir KM10-273
Au13C DMSO {C:\Bruker\TopSpin3.5pl6} 1708 11
144.47
136.94
120.02
117.00
114.97
114.88
270 260 250 240 230 220 210 200 190 180 170 160 150 140 130 120 110 100 90 80 70 60 50 40 30 20 10 0 -10
f1 (ppm)

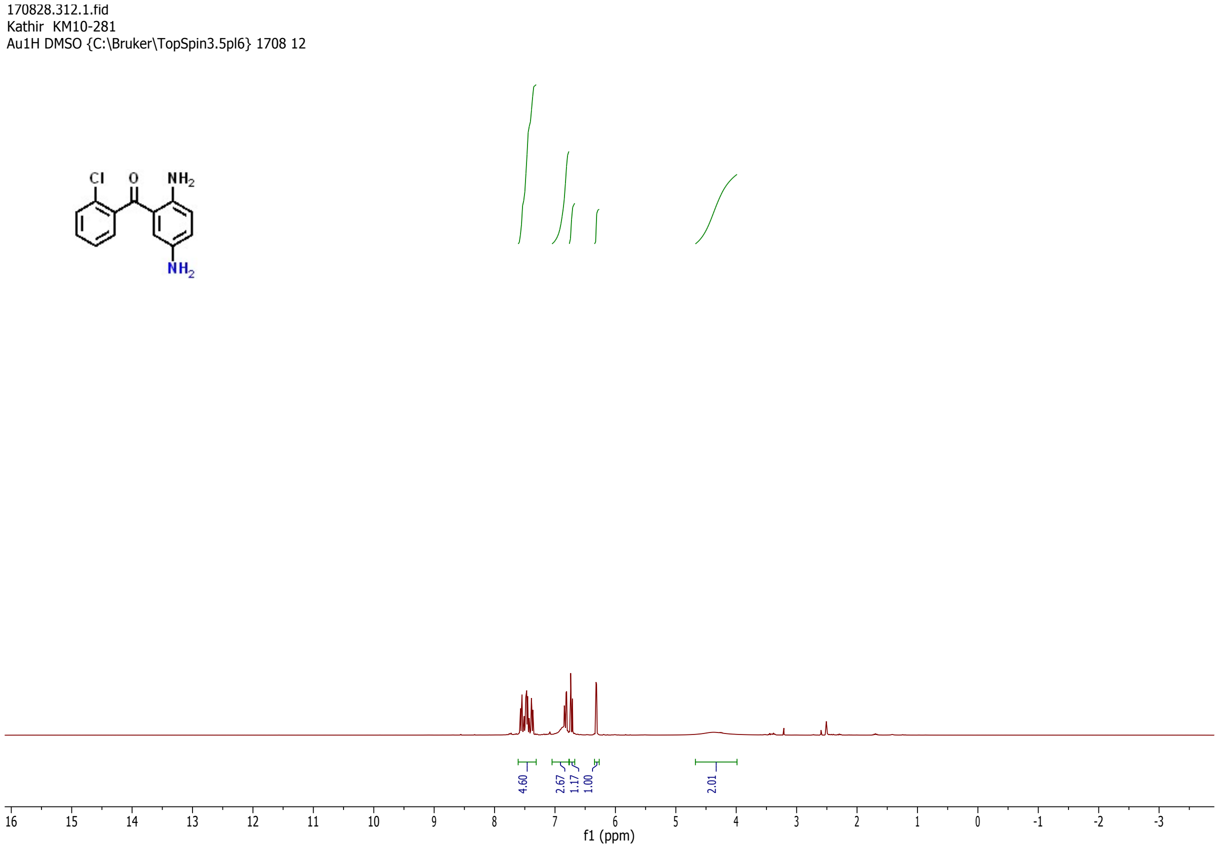
170828.312.1.fid
Kathir KM10-281
Au1H DMSO {C:\Bruker\TopSpin3.5pl6} 1708 12
Cl
O
NH2
NH2
4.60
2.67
1.17
1.00
2.01
16 15 14 13 12 11 10 9 8 7 6 5 4 3 2 1 0 -1 -2 -3
f1 (ppm)

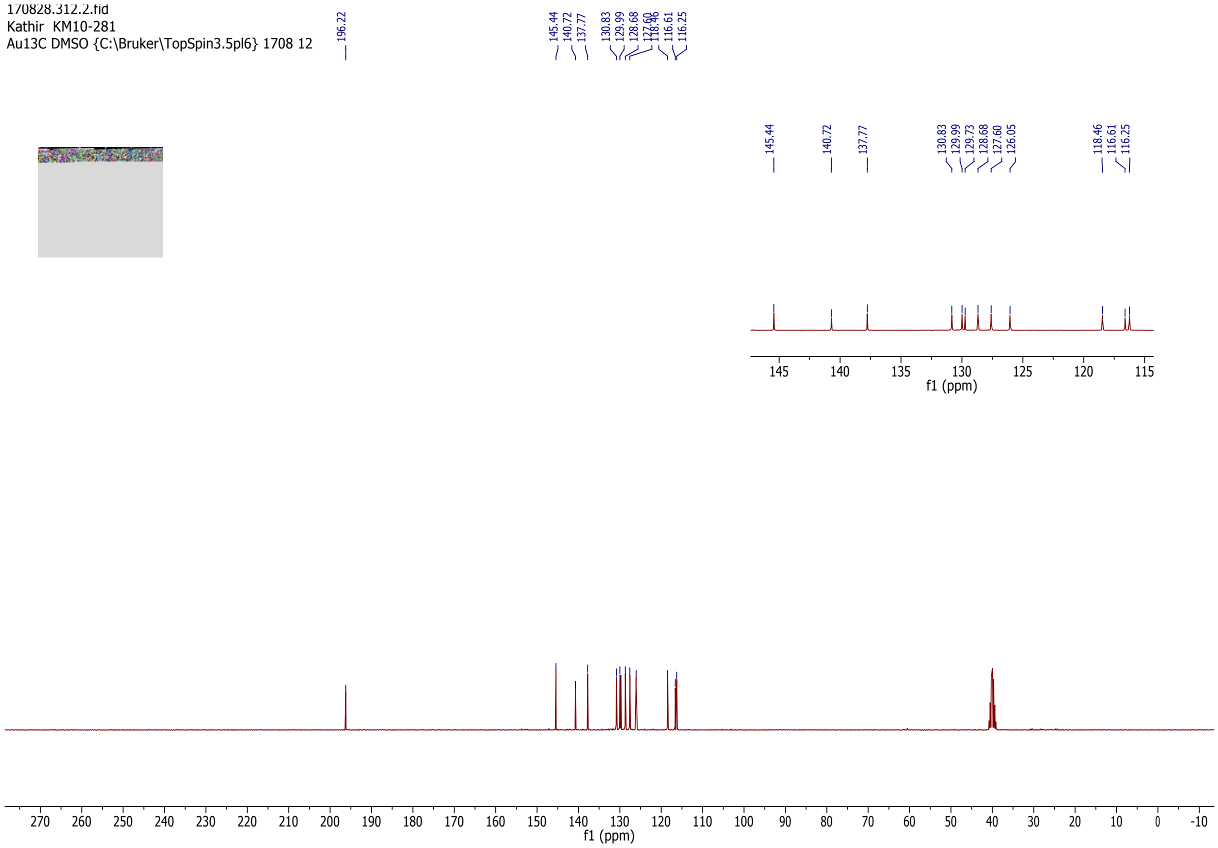
170828.312.2.fid
Kathir KM10-281
Au13C DMSO {C:\Bruker\TopSpin3.5pl6} 1708 12
196.22
145.44
140.72
137.77
130.83
129.99
129.73
128.68
127.60
126.05
118.46
116.61
116.25
f1 (ppm)

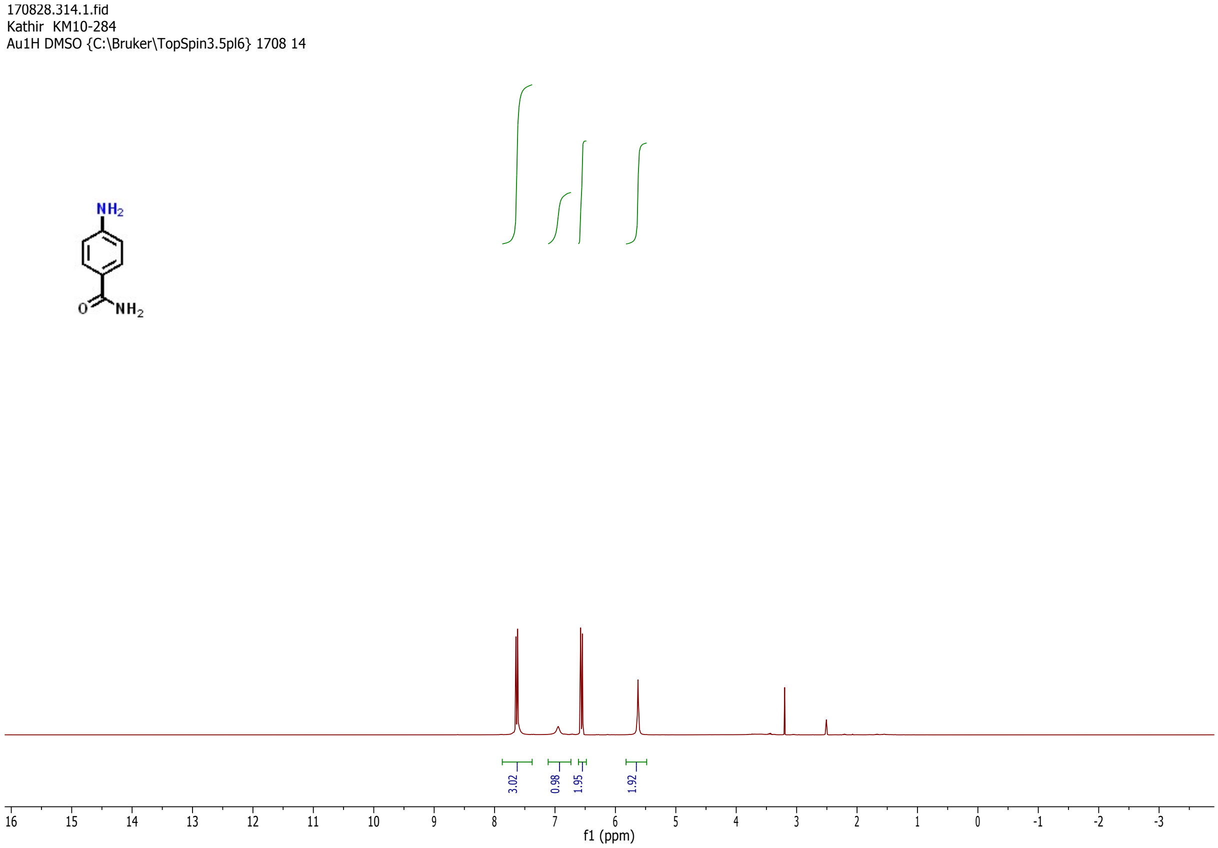
170828.314.1.fid
Kathir KM10-284
Au1H DMSO {C:\Bruker\TopSpin3.5pl6} 1708 14
NH2
O
NH2
3.02
0.98
1.95
1.92
f1 (ppm)

170828.314.2.fid
Kathir KM10-284
Au13C DMSO {C:\Bruker\TopSpin3.5pl6} 1708 14

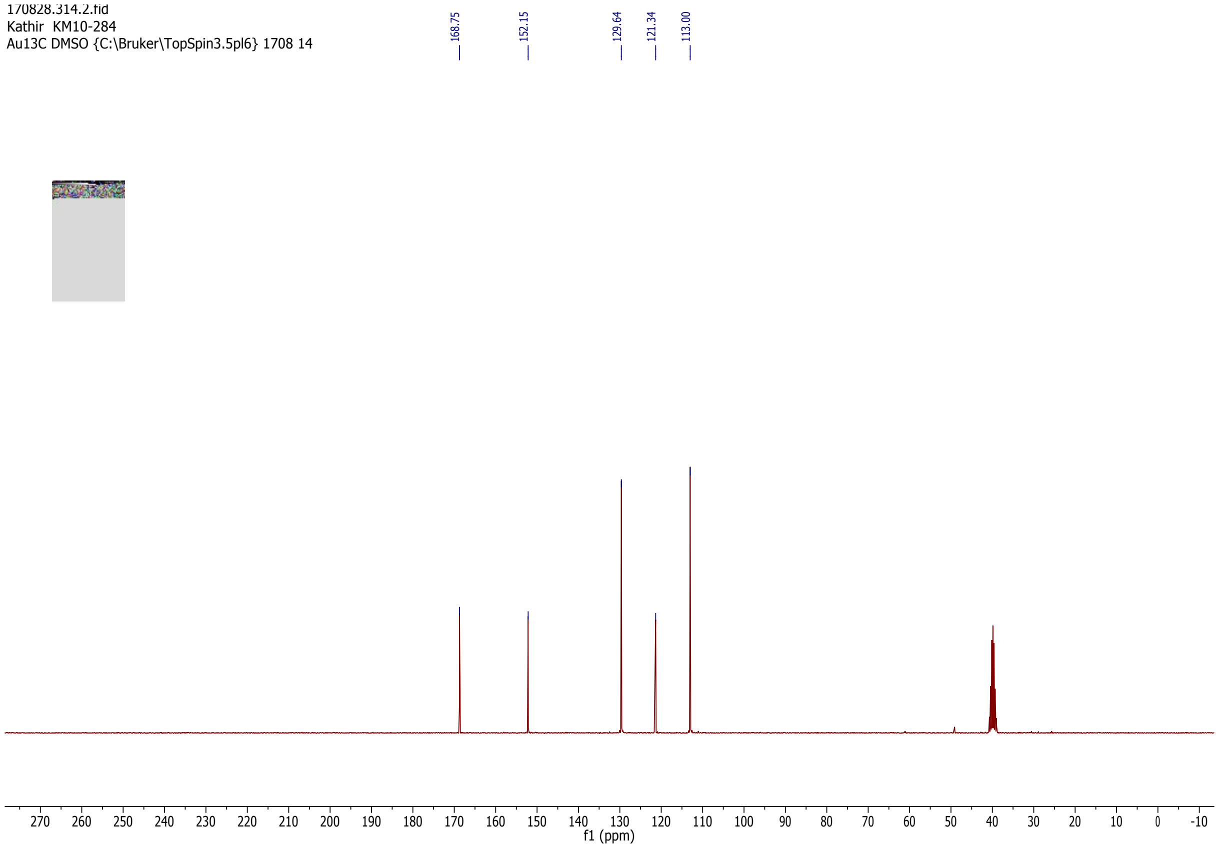

170828.315.1.fid
Kathir KM10-285
Au1H DMSO {C:\Bruker\TopSpin3.5pl6} 1708 15

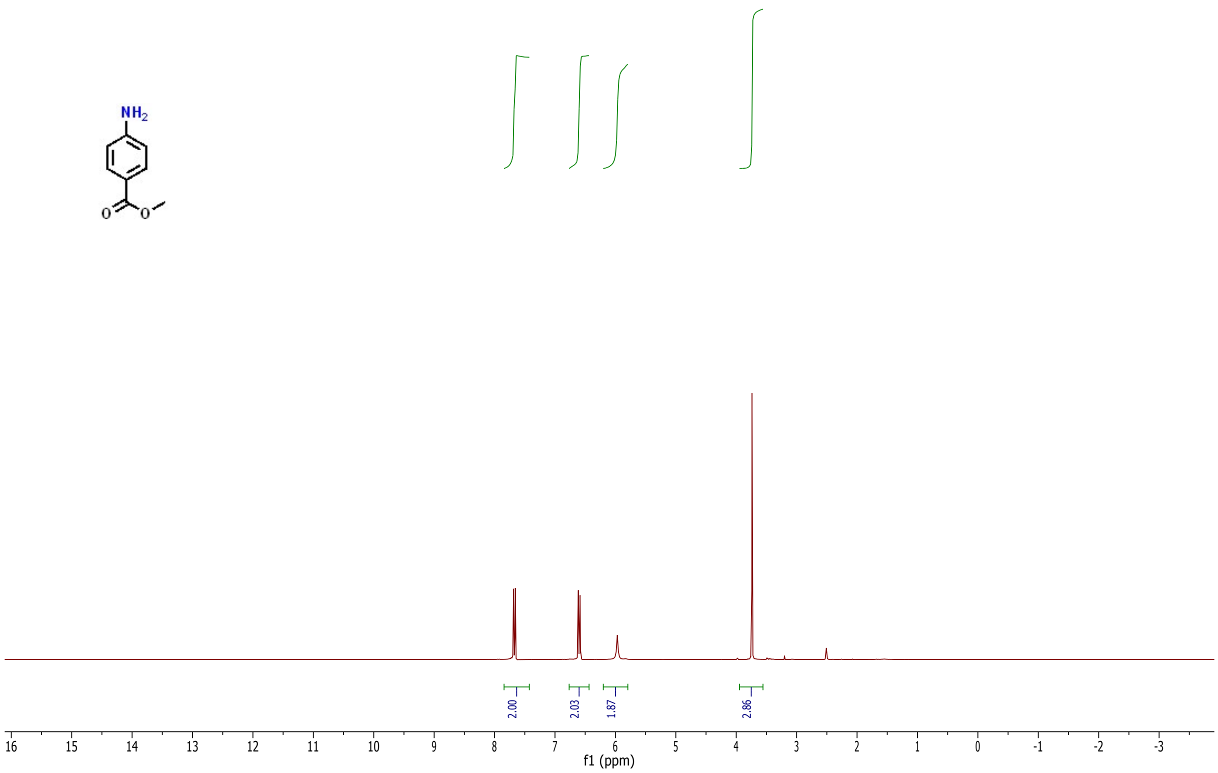

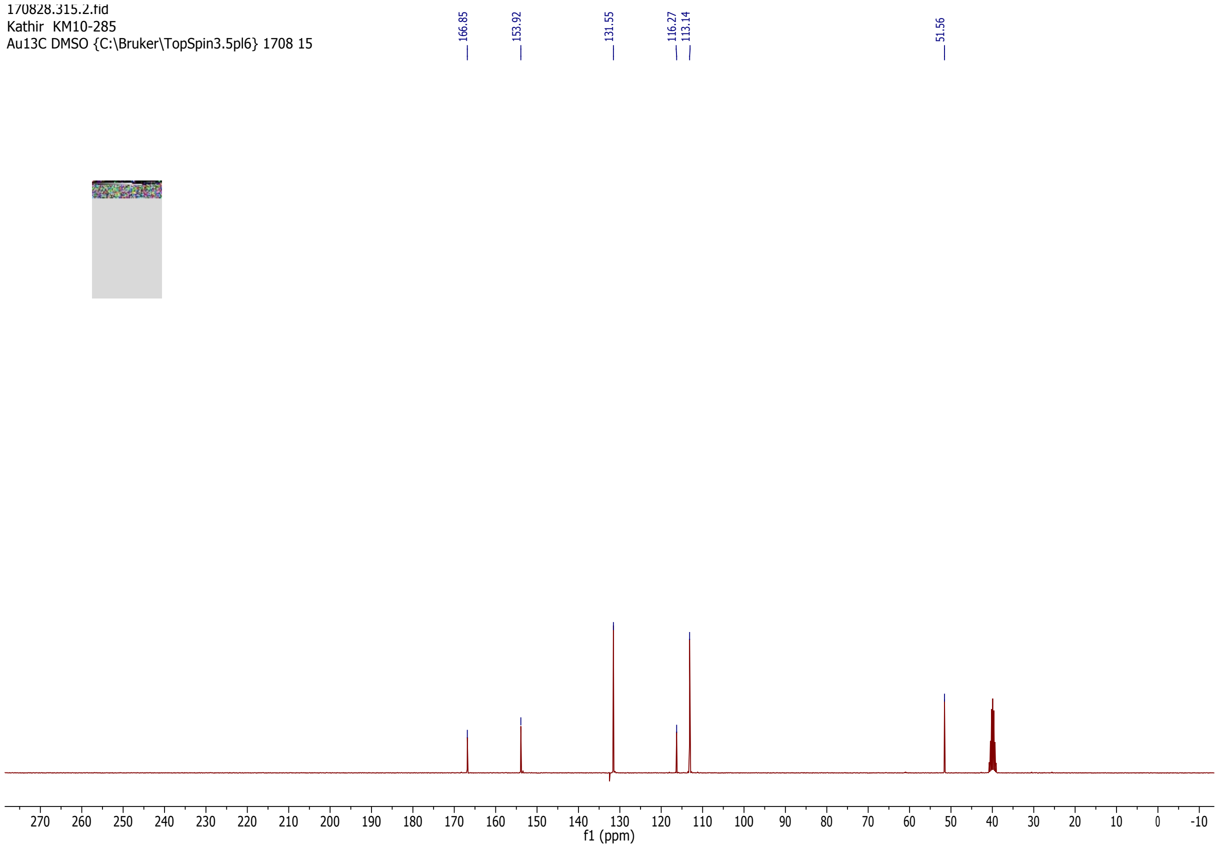
170828.315.2.fid
Kathir KM10-285
Au13C DMSO {C:\Bruker\TopSpin3.5pl6} 1708 15
166.85
153.92
131.55
116.27
113.14
51.56
f1 (ppm)

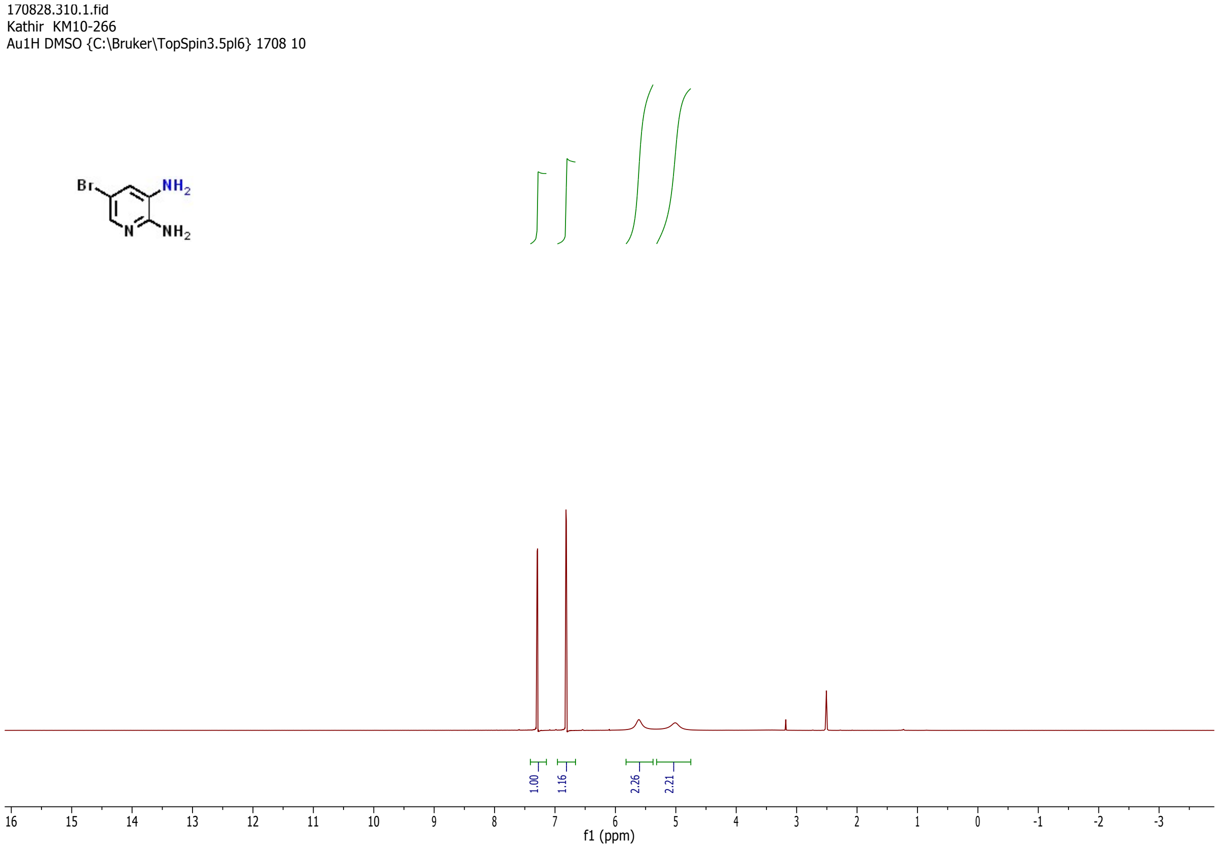
170828.310.1.fid
Kathir KM10-266
Au1H DMSO {C:\Bruker\TopSpin3.5pl6} 1708 10
Br
NH2
N
NH2
1.00
1.16
2.26
2.21
f1 (ppm)

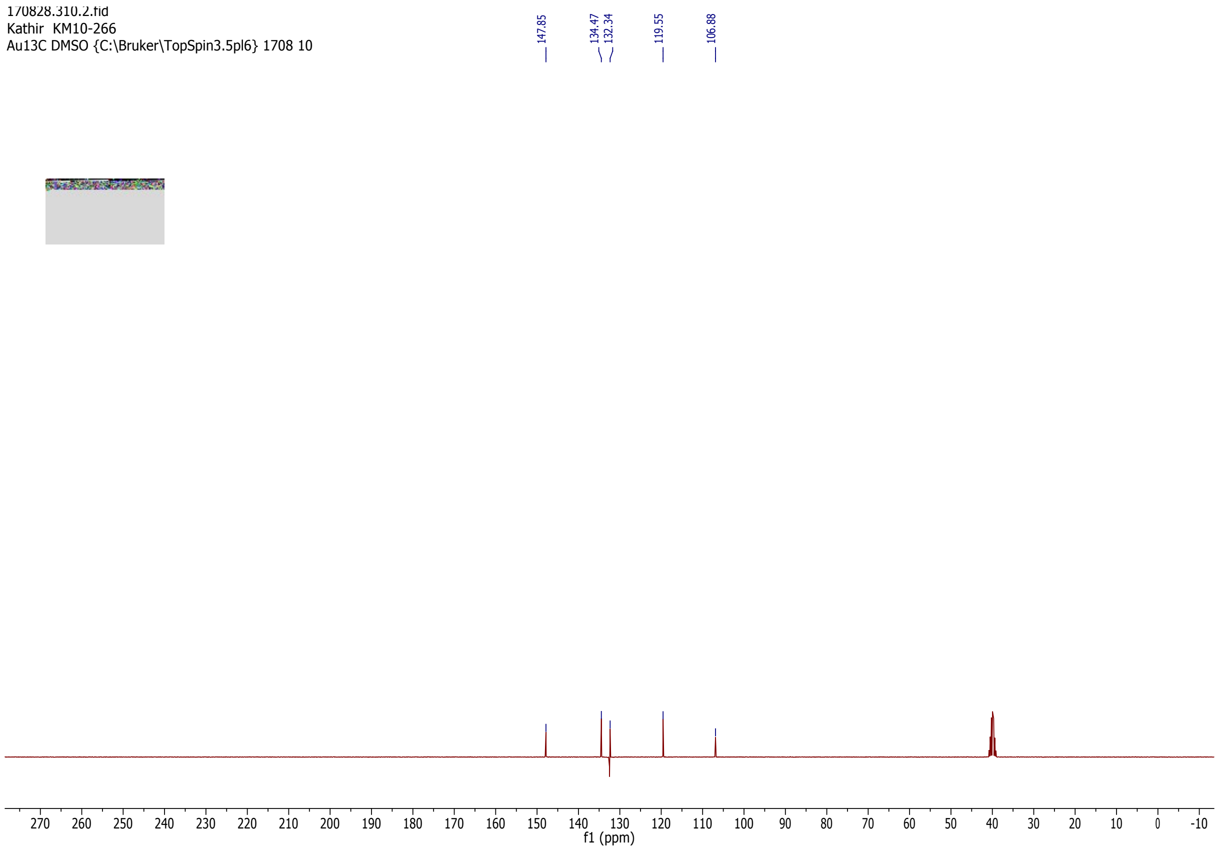
170828.310.2.fid
Kathir KM10-266
Au13C DMSO {C:\Bruker\TopSpin3.5pl6} 1708 10
147.85
134.47
132.34
119.55
106.88
270 260 250 240 230 220 210 200 190 180 170 160 150 140 130 120 110 100 90 80 70 60 50 40 30 20 10 0 -10
f1 (ppm)
